# clevRvis: visualization techniques for clonal evolution

**DOI:** 10.1093/gigascience/giad020

**Published:** 2023-04-11

**Authors:** Sarah Sandmann, Clara Inserte, Julian Varghese

**Affiliations:** Institute of Medical Informatics, University of Münster, Münster 48149, Germany; Institute of Medical Informatics, University of Münster, Münster 48149, Germany; Institute of Medical Informatics, University of Münster, Münster 48149, Germany

**Keywords:** clonal evolution, tumor development, visualization, cancer cell fraction, biallelic events, therapy effect

## Abstract

**Background:**

A thorough analysis of clonal evolution commonly requires integration of diverse sources of data (e.g., karyotyping, next-generation sequencing, and clinical information). Subsequent to actual reconstruction of clonal evolution, detailed analysis and interpretation of the results are essential. Often, however, only few tumor samples per patient are available. Thus, information on clonal development and therapy effect may be incomplete. Furthermore, analysis of biallelic events—considered of high relevance with respect to disease course—can commonly only be realized by time-consuming analysis of the raw results and even raw sequencing data.

**Results:**

We developed clevRvis, an R/Bioconductor package providing an extensive set of visualization techniques for clonal evolution. In addition to common approaches for visualization, clevRvis offers a unique option for allele-aware representation: plaice plots. Biallelic events may be visualized and inspected at a glance. Analyzing 4 public datasets, we show that plaice plots help to gain new insights into tumor development and investigate hypotheses on disease progression and therapy resistance. In addition to a graphical user interface, automatic phylogeny-aware color coding of the plots, and an approach to explore alternative trees, clevRvis provides 2 algorithms for fully automatic time point interpolation and therapy effect estimation. Analyzing 2 public datasets, we show that both approaches allow for valid approximation of a tumor’s development in between measured time points.

**Conclusions:**

clevRvis represents a novel option for user-friendly analysis of clonal evolution, contributing to gaining new insights into tumor development.

## Background

For many types of cancer, determining their mutational profile is a crucial step, having an impact on diagnosis as well as treatment. In multiple myeloma, for example, cytogenetic aberrations have been found to have a significant impact on prognosis and are thus considered in the Revised International Staging System for risk stratification [1,2]. Similarly, in myelodysplastic syndromes (MDS), patients are commonly stratified according to the Revised International Prognostic Scoring System, evaluating, among others, cytogenetic abnormalities [3]. Recently, it has been shown that the analysis of point mutations and small insertions/deletions (indels) even allows for identification of clinically relevant subgroups within low-risk MDS patients [4].

In addition to information on the bare presence or absence of variants, their development over time, the clonal evolution [5], is of major importance in several diseases, for example, MDS, acute myeloid leukemia, or Burkitt lymphoma (BL) [6–8]. For a thorough analysis of clonal evolution, all variants ranging from point mutations to aberrations affecting whole chromosomes should be taken into account. Commonly, various sources of data are considered to allow for valid detection of these variants, for example, karyotyping, fluorescence in situ hybridization, microarrays like single-nucleotide polymorphism (SNP) arrays or array-CGH (aCGH), next-generation sequencing, and Sanger sequencing. By integrating all of these data, clonal evolution may be reconstructed [7].

Studying clonal evolution in more detail, a diverse set of analyses can be performed: the model of clonal evolution (linear vs. branched dependent vs. branched independent; of note, neutral and punctuated evolution will be considered special cases of branched and linear evolution) [8,9] can be determined; correlation to blood parameters, therapy resistance, and disease progression investigated [10]; and patterns characterizing subgroups of patients explored. For example, by detailed analysis of clonal evolution, evidence was found that relapsing BL is associated with the presence of clones, featuring double-hit events in *TP53* [8].

However, these analyses may be hampered by different aspects: (i) variants are only detected at few time points, providing an incomplete representation of the disease course. (ii) No information on a therapy’s effect on clonal evolution is available. (iii) Evaluation of biallelic events requires tedious manual work, analyzing raw sequencing data and detailed variant calling information.

To overcome these obstacles, we developed clevRvis—an R/Bioconductor package for clonal evolution in R, providing innovative visualization techniques. In addition to common R functions, clevRvis offers a web-based graphical user interface, allowing usage not just by computer scientists but also by physicians and biologists. Our approach contains fully automatic algorithms for interpolating additional time points as well as estimating therapy effect. Evaluating 2 real, publicly available datasets from different disease entities, characterized by a high number of measured time points, we show that both estimation approaches generate valid results.

clevRvis generates 3 different types of plots: (i) shark plots (a graph-based representation of clonal evolution), (ii) dolphin plots (a fish plot-like representation, optionally also considering interpolated time points and estimated therapy effect), and (iii) plaice plots (a novel type of plots allowing for detection of biallelic events at a glance). All plots generated by clevRvis are highly customizable. Following recommendations for graphical strategies in clonal evolution [11], we implemented an algorithm for phylogeny-aware color coding of the clones to obtain optimal visualization. In addition, alternative phylogenetic trees can be determined and explored interactively. By analysis of 4 public datasets, we show that visualization with clevRvis outperforms common alternative approaches. Additionally, we show the added value of plaice plots.

## Methods

### Datasets analyzed

We analyze 4 real datasets, containing detailed information on the clonal evolution of *n* = 31 patients. Table 1 provides an overview of the datasets and their main characteristics.

**Table 1:** Overview of the datasets analyzed with clevRvis. BL, Burkitt lymphoma; CLL, chronic lymphatic leukemia; FISH, fluorescence in situ hybridization; MDS, myelodysplastic syndromes; MPN, myeloid neoplasia; SNP, single-nucleotide polymorphism; tNGS, targeted next-generation sequencing; WES, whole-exome sequencing.

Dataset	Disease	No. of patients	No. of time points	No. of clones	Clonal evolution	Data types
			(median [min–max])	(median [min–max])	models	
1	MDS	11	5 [5–30]	5 [1–9]	Linear, branched dependent,	Karyotyping, FISH,
					branched independent	SNP array, WES, tNGS
2	CLL	2	9.5 [4–15]	4 [3–5]	Linear, branched dependent	Karyotyping, FISH,
						WES, tNGS
3	MPN	8	2 [1–5]	5.5 [2–11]	Linear, branched dependent,	Karyotyping, FISH,
					branched independent	aCGH, tNGS
4	BL	10	1.5 [1–2]	9 [7–17]	Linear, branched dependent	FISH, SNP array
						WES, tNGS, Sanger

The first set covers data from 11 patients with MDS. Clonal evolution was reconstructed based on karyotyping, FISH, SNP array, whole-exome sequencing (WES), and ultra-deep targeted next-generation sequencing (tNGS) data [6]. The dataset is characterized by a high number of time points (up to 30) and a relatively low number of clones. All major models of clonal evolution are present. Six patients received supportive care only, while 5 additionally received lenalidomide, which is expected to impact clonal evolution. The dataset serves as a test case, exploring options for visualizing clonal evolution and validating our approaches for automatic time point interpolation and therapy effect estimation (information on how input data for the analysis with clevRvis were derived from the publication by da Silva-Coelho et al. [6] is available in Additional File 1, Section 1.1).

The second set covers data from 2 patients with chronic lymphatic leukemia. Clonal evolution was reconstructed based on FISH, WES, and tNGS data [12]. The dataset provides detailed information on follow-up—especially on therapies applied—for up to 12 years, as well as regular variant detection throughout the whole time of follow-up. Thereby, this dataset serves as a second test case for the analysis with clevRvis, considering time point interpolation and therapy effect estimation in a different disease entity and a different source of data (information on how input data for the analysis with clevRvis were derived from the publication by González-Rincón et al. [12] is available in Additional File 1, Section 1.2).

The third set covers data from 8 patients with myeloid neoplasia. Clonal evolution was reconstructed based on karyotyping, FISH, aCGH, and tNGS data [7]. Presence of 1 to 5 time points and an increased number of clones (1 case of branched dependent evolution can be specified more precisely as neutral evolution [9]) pose a challenge for visualization. Partly, no samples were collected toward the end of therapy, requiring interpolation of additional time points and estimating the effect of therapy (information on how input data for the analysis with clevRvis were derived from the publication by Sandmann et al. [7] is available in Additional File 1, Section 1.3).

The fourth set covers data from 10 patients with BL. Clonal evolution was reconstructed based on FISH, SNP array, WES, tNGS, and Sanger sequencing data [8]. The dataset is characterized by a low number of time points (1 for nonrelapsing patients, 2 for relapsing patients). While therapy is known to have been applied, no data showing its effect on clonal evolution are available. Thus, circumstances require interpolation of time points and estimation of therapy effect for all patients. Additionally, samples feature a high number of clones (up to 17), which is expected to be a major challenge for visualization (information on how input data for the analysis with clevRvis were derived from the publication by Reutter et al. [8] is available in Additional File 1, Section 1.4).

All datasets contain information on detected small variants (SNVs and indels) as well as large variants (structural variants and copy number variants [CNVs]) for every patient. Analysis of clonal evolution always involves integration of various data sources and partly overlapping variants. Thereby, the detection of biallelic variants and their evaluation by plaice plots is key for all datasets.

### clevRvis

clevRvis provides an extensive set of visualization techniques for clonal evolution. An overview of the analysis pipeline is provided in Fig. 1 (screenshots of the software are available in Additional File 1, Supplementary Figs. S1– S8).

**Figure 1:**
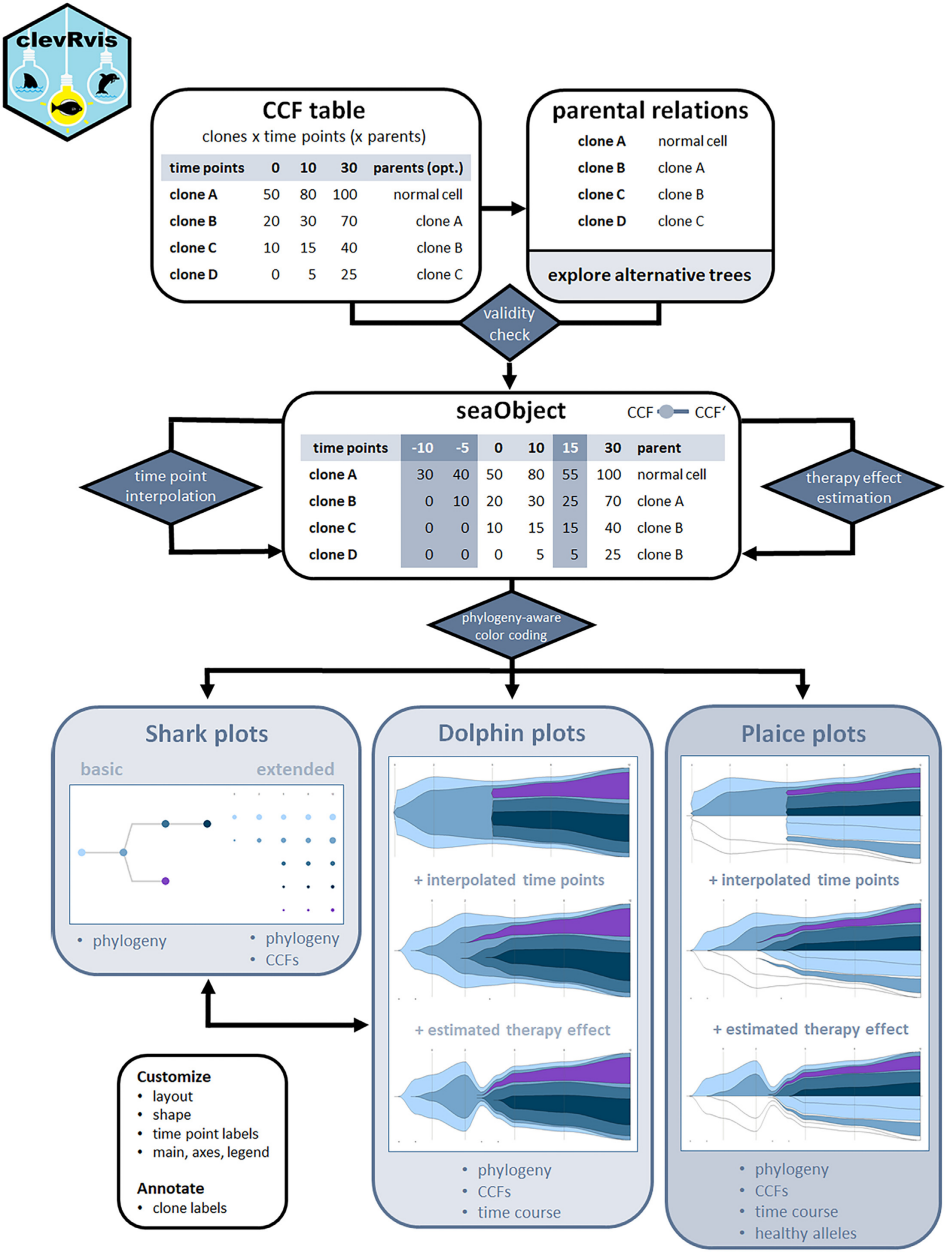
Overview of the analyses performed by clevRvis. Based on a CCF table and information on parental relations, a validity check is performed. For the resulting seaObject, additional time points may be interpolated and therapy effect may be estimated. Using phylogeny-aware color coding, shark plots, dolphin plots, and plaice plots can be generated.

For the subsequent description of the analyses performed by clevRvis, we use the following definitions: *ccf*(*c, t_i_*) is defined as the cancer cell fraction (CCF) of clone *c*, with *c* ∈ {1,..., *n*}, at time point *t_i_*, with *t_i_* ∈ {*t*_1_,..., *t_m_*}. *parent*(*c*) is defined as the parent-clone of clone *c* and *children*(*c*) as the children-clone(s) of clone *c*. For a clone *c* developing from normal cells, *parent*(*c*) = 0. All clones developing from normal cells are defined by *children*(0). *ccf*^′^(*c, t_i_*) is defined as the difference in CCFs of a clone *c* at time point *t_i_*. Thus, *ccf*^′^(*c, t_i_*) = *ccf*(*c, t_i_*) − ∑_*j* ∈ *children*(*c*)_*ccf*(*j, t_i_*).

#### Validity check

clevRvis requires the upload of a *CCF table*, containing information on the CCF of every clone at every time point, optionally also including information on parental relations. In an interactive dialogue, the user can subsequently—if this information has not been uploaded—define the parental relations for every clone listed in the CCF table. Normal cells as well as clones different from the considered one may be selected. Upon submitting this information to generate the initial *seaObject*, a validity check is performed, adapting and extending the check performed by fishplot [13] (see Algorithm 1).

**Table:** 

Algorithm 1 validity check
**for each** *t_i_* **in** {*t*_1_,..., *t_m_*} **do**
**for each** *c* **in** {1,..., *n*} **do**
**if** *ccf*(*c, t_i_*) < ∑_*j* ∈ *children*(*c*)_*ccf*(*j, t_i_*) **then**
**return** check.failed
**if** ∑_*j* ∈ *children*(0)_*ccf*(*j, t_i_*) > 100 **then**
**return** check.failed
**if** *ccf*(*c, t_i_*) > 0 **and** *ccf*(*c, t*_*i* + 1_) = 0 **and** *ccf*(*c, t*_*i* + 2_) > 0 **then**
**return** check.failed
**if** *ccf*^′^(*c, t_i_*) > 0 **and** *ccf*^′^(*c, t*_*i* + 1_) = 0 **and** *ccf*^′^(*c, t*_*i* + 2_) > 0 **then**
**return** check.failed
**return** check.passed

Summing up, (i) children-clones cannot exceed their parents, (ii) clones developing from normal cells cannot add up to more than 100%, (iii) a clone cannot reappear, and (iv) a parent-clone being thoroughly replaced by its children-clone(s) cannot reappear.

#### Exploring alternative trees

Alternative parental relations, resulting in alternative clonal evolution trees, can be explored interactively using clevRvis. Considering *n* clones, a parental relations vector of length *n* has to be defined. For every clone, 1 out of *n* options can be chosen (*n* − 1 different clones + normal cells), which results in *n*^*n*^ permutations. To optimize runtime, filtration of clearly invalid options is performed prior to the thorough validity check by clevRvis (see Algorithm 2).

**Table:** 

Algorithm 2 exploring alternative trees
**for each** *c* **in** {1,..., *n*} **do**
**for each** *t_i_* **in** {*t*_1_,..., *t_m_*} **do**
**if** *ccf*(*c, t_i_*) < any other *ccf*(*j, t_i_*) **then**
**return** *c*! = *parent*(*j*)
**for each remaining** *c* **in** {1,..., *n*} **do**
**for each remaining** *t_i_* **in** {*t*_1_,..., *t_m_*} **do**
**if** *parent*(*j*) can only be *c* **then**
**if** *ccf*(*c, t_i_*) − *ccf*(*j, t_i_*) < any other *ccf*(*k, t_i_*) **then**
**return** *c*! = *parent*(*k*)
**for each** remaining.parental.relations
apply **Algorithm 1** validity check
**return** valid.parental.relations

As a first filtration step, possible linear relations are investigated: if a clone *c* has, at any time point, a lower CCF compared to clone *j* at the same time point, *c* cannot be the parent of *j*. Subsequently, possible branched relations are taken into account: if a clone *j* clearly develops from clone *c*, an additional clone *k* can—as a branch—only develop from *c* if *ccf*(*c, t_i_*) − *ccf*(*j, t_i_*) ≥ *ccf*(*k, t_i_*) for every time point *t_i_*. On the basis of the remaining filtered options, permutations are determined and a thorough validity check (see Algorithm 1) is performed (maximum: 20,000 permutations). All valid parental relations are reported and alternative trees can be explored subsequently.

#### Time point interpolation

The initially generated seaObject may be extended by additional interpolated time points. The general idea of estimating auxiliary time points to improve visualization of clonal evolution was first outlined by Reutter et al. [8]. However, this initial approach mainly focused on improving figures generated by fishplot [13], considering only the difference in CCFs at a later measured time point. Furthermore, it did not contain any algorithm for automatic time point interpolation. Evaluating different scenarios of clonal evolution, we implemented an improved, fully automatic approach in clevRvis (see Algorithm 3).

**Table:** 

Algorithm 3 time point interpolation
**if** interpolate initial development **then**
*initial.clones* ← all clones *c* where *ccf*(*c, t*_1_) ≠ 0
*nested.levels* ← nested levels for all *initial.clones*
*new.time.points* ← *maximum*(*nested.levels*)
**for each** *new.time.point* **in** {1,..., *new.time.points*} **do**
// 1 refers to time point closest to *t*_1_
**for each** initial clone *c* with *maximum*(*nested.levels*) **do**
*ccf*(*c, new.time.point*) ← *ccf*^′^(*c, new.time.point*) ← 0
determine *ccf*(*remaining.initial.clones, new.time.point*) based on *ccf*^′^(*initial.clones, new.time.point*)
remove *maximum*(*nested.levels*)
**if** interpolate development between measured time points **then**
*old.clones* ← all clones *c* where *ccf*(*c, t_i_*) ≠ 0
*new.clones* ← all clones *c* where *ccf*(*c, t_i_*) = 0 **and** *ccf*(*c, t*_*i* + 1_) ≠ 0
*new.unique.nested.levels* ← unique nested levels for *new.clones*
*new.time.points* ← *new.unique.nested.levels* − 1
**for each** clone *c* **in** *old.clones* **do**
*ccf*(*c, new.time.points*) ← linear development from *ccf*(*c, t_i_*) to *ccf*(*c, t*_*i* + 1_)
**for each** clone *c* **in** *new.clones* **do**
*z* ← number of *new.unique.nested.levels* < *nested.level*(*c*)
**for each** *new.time.point* **in** {1,..., *z*} **do**
*ccf*(*c, new.time.point*) ← 0
*ccf*(*c, remaining.new.time.points*) ← linear development from *ccf*(*c, z*) to *ccf*(*c, t*_*i* + 1_)
**return** updated *ccf*

The number of time points interpolated by clevRvis depends on the clonal evolution being analyzed and cannot be defined by a user. We differentiate between interpolating development of a tumor prior to the first measured time point and interpolating development between 2 measured time points. By default, all interpolated time points are evenly distributed (a detailed description on how to implement skewed events is available in Additional File 1, Section 1.6).

We assume that clones of the same nested level developed at approximately the same time. A higher nested level indicates development at a later time. For example, in a linear evolution with clone B developing from clone A, it is sensible to assume that clone A developed first and expanded. Over time, a cell of clone A acquired additional mutations, finally resulting in the formation of clone B.

Interpolating time points prior to *t*_1_, we focus on the difference in CCFs *ccf*^′^(*c, t*_1_) for all clones present at *t*_1_ (*initial.clones*). The number of interpolated time points is defined by the maximum nested level of the initially present clones. As the clone(s) with the highest nested level are assumed to have developed last, *ccf*^′^ and *ccf* are set to zero for the first (and all subsequent) interpolated time points. The CCFs of the remaining initial clones are updated. The procedure is repeated with the second highest nested level, and so on, until only clones with nested level 0 remain.

Interpolating time points between *t_i_* and *t*_*i* + 1_, we assume linear development of CCF for all clones already present at *t_i_* (*old.clones*). For newly developing clones (*new.clones*), we determine their nested levels. The number of unique nested levels (*new.unique.nested.levels*) − 1 defines the number of interpolated time points. We stick to our assumption that clones with a higher nested level developed at a later time point. Thus, newly developing clone(s) with the lowest nested level are considered first. Linear development between *t_i_* and *t*_*i* + 1_ is assumed. For all clones with the second lowest nested level, CCF at the first interpolated time point is set to zero. Linear development is assumed for the remaining interpolated time points up to *t*_*i* + 1_. The procedure is repeated with the third lowest nested level, and so on, until only clones with the highest *new.unique.nested.levels* remain.

Of note, to generate smoother dolphin and plaice plots in R, for all clones, 0.1 is added to the (interpolated) time point prior to their first appearance.

#### Therapy effect estimation

If a therapy is applied, interpolating time points is assumed to be insufficient to approach the development of CCFs over time properly. In the absence of therapy, the overall tumor load is not expected to decrease. Any decrease in CCF differences over time is assumed to be caused by the expansion of superior children-clones. In the presence of therapy, however, we assume that any observed decrease in CCF differences is due to therapy. This general idea was first outlined by Reutter et al. [8] as well. In clevRvis, we implemented an updated, fully automatic approach for therapy effect estimation (see Algorithm 4).

**Table:** 

Algorithm 4 therapy effect estimation
*old.clones* ← all clones *c* where *ccf*(*c, t_i_*) ≠ 0
*new.clones* ← all clones *c* where *ccf*(*c, t_i_*) = 0 **and** *ccf*(*c, t*_*i* + 1_) ≠ 0
**for each** clone *c* **in** *old.clones* **do**
*ccf*^′^(*c, therapy.time.point*) ← *minimum*(*ccf*^′^(*c, t_i_*), *ccf*^′^(*c, t*_*i* + 1_))
determine *ccf*(*old.clones, therapy.time.point*) based on *ccf*^′^(*old.clones, therapy.time.point*)
*ccf*(*new.clones, therapy.time.point*) ← 0
**if** additional time point interpolation **then**
*therapy.clones* ← all clones *c* where *ccf*(*c, therapy.time.point*) ≠ 0
*final.clones* ← all clones *c* where *ccf*(*c, t*_*i* + 1_) ≠ 0
*new.final.clones* ← all clones *c* where *ccf*(*c, t*_*i* + 1_) ≠ 0 **and***ccf*(*c, therapy.time.point*) = 0
*nested.levels* ← nested level for all *new.final.clones*
recalculate *nested.levels* ignoring *therapy.clones*
*new.time.points* ← *maximum*(*recalculated.nested.levels*)
**for each** clone *c* **in** *new.final.clones* **do**
*z* ← *recalculated.nested.level*(*c*)
**for each** *new.time.point* **in** {1,..., *z*} **do**
// 1 refers to time point closest to *therapy.time.point*
*ccf*(*c, new.time.point*) ← *ccf*^′^(*c, new.time.point*) ← 0
**if** *c* has no children **then**
*ccf*(*c, remaining.new.time.points*) ← linear development from *ccf*(*c, z*) to *ccf*(*c, t*_*i* + 1_)
**if** *c* has children **then**
*ccf*^′^(*c, remaining.new.time.points*) ← *ccf*^′^(*c, t*_*i* + 1_)
determine *ccf*(*remaining.final.clones, new.time.points*) based on *ccf*^′^(*final.clones, new.time.points*)
**return** updated *ccf*

Estimating therapy effect, a single time point is added, corresponding to the clonal composition toward the end of therapy. The precise time point is user-definable (default: evenly distributed). In case new clones developed, enabling additional time point interpolation is recommended.

To estimate the effect of a therapy that has been applied between *t_i_* and *t*_*i* + 1_, we focus on the difference in CCFs *ccf*^′^(*c, t_i_*) for all clones present at *t_i_* (*old.clones*). Assuming that no increase in tumor load is observed during therapy, the minimum of *ccf*^′^(*c, t_i_*) and *ccf*^′^(*c, t*_*i* + 1_) is determined. For all newly developing clones (*new.clones*), we assume that they developed after the application of therapy. Thus, CCFs are set to 0 for the estimated time point (*therapy.time.point*).

Interpolating additional time points—located between *therapy.time.point* and *t*_*i* + 1_—we focus on newly developing clones. For all clones only present at *t*_*i* + 1_ (*new.final.clones*), we recalculated the nested levels, ignoring clones already present at *therapy.time.point*. As an example, we consider 3 clones A, B, and C that develop linearly. While A and B are already present at *t_i_*, clone C only emerges at *t*_*i* + 1_. Another clone D develops from normal cells and—just like clone C—only emerges at *t*_*i* + 1_. Nested levels of clones C and D are 2 and 0, respectively. However, development of both clones is just “one step” compared to the starting position at *t_i_*. Therefore, the recalculated nested levels, ignoring clones A and B, are 0 for both C and D.

Sticking to our main assumption that clones with a higher (recalculated) nested level develop at a later time, additional time points are interpolated. The number of time points added is defined by the maximum recalculated nested level. Newly developing clone(s) with the lowest recalculated nested level are considered first. If a clone *c* has no children, linear development between *therapy.time.point* and *t*_*i* + 1_ is assumed. Otherwise, we assume that the CCF for clone *c* at all interpolated time points is defined by *ccf*^′^(*c, t*_*i* + 1_). For all clones with the second lowest recalculated nested level, CCF at the first interpolated time point is set to 0. Subsequently, CCFs for the remaining time points are interpolated considering either linear development or *ccf*^′^(*c, t*_*i* + 1_). The procedure is repeated with the third lowest nested level, and so on, until only clones with the highest *recalculated.nested.level* remain.

#### Phylogeny-aware color coding

Based on an evaluation of graphical strategies for visualizing clonal evolution published by Krzywinski [11], we developed an approach for automatic phylogeny-aware color coding. Our approach sticks to the following rules:

1. The higher the nested level of a clone, the darker the hue.
2. Clones of the same branch are colored by a similar hue.
3. Clones on 2 (or more) branches with a common ancestor (branched dependent evolution) are colored by similar but diverging colors.
4. Clones on 2 (or more) branches without a common ancestor (branched independent evolution) are colored by colors of maximum difference.

The exact range of the color palette is dynamically determined based on the clonal evolution provided as input data (number of clones, maximum nested level). Thereby, options for differentiating between closely related clones are optimized. clevRvis supports a maximum of 25 independent clones, developing from normal cells, and an unlimited number of related clones.

#### Shark plots

Shark plots serve a basic, raw visualization of clonal evolution (see Fig. 1). Using a classical graph approach, clones are represented by nodes and parental relations by edges. Thus, the phylogeny can be directly deduced from shark plots.

Optionally, shark plots can be extended to provide information on CCFs as well. Clones are additionally visualized next to the actual shark plot. The size of each clone is correlated with its CCF. Time points are plotted next to each other.

If shark plots are chosen to be plotted along with dolphin plots, both plots are connected interactively. By hovering over one of the clones, it is automatically highlighted in both shark and dolphin plots.

#### Dolphin plots

An advanced visualization of clonal evolution is realized by dolphin plots, mainly corresponding to well-established fish plots. The development of each clone over time is displayed on the x-axis and the CCFs on the y-axis. Thereby, information on phylogeny, CCFs, and time course characterizing a clonal evolution is jointly visualized in a single plot. Several basic options for customizing dolphin plots are available (e.g., switching between spline and polygon shape or separating independent clones). Additionally, a user may choose between standard centered visualization of clonal evolution and bottom layout. This causes the clones to develop as “lying” on the x-axis (similar to visualization of clonal evolution by timescape [14]). Automatically, the longest branch is chosen to be plotted on the bottom, while the remaining branches are added on top.

If a seaObject has been extended by additional interpolated time points and/or estimated therapy effect, both can be visualized by dolphin plots. Customizable labels can be added to distinguish between measured and estimated time points.

#### Plaice plots

Plaice plots represent a derivative of dolphin plots (respectively, fish plots) developed to improve visualization of biallelic events. Instead of 1, we consider 2 “flatfish” (=plaice) that are mirrored above and below the y-axis.

Common clonal evolution is visualized only in the upper plot in the bottom layout. Similar to dolphin plots, a user may choose between spline and polygon shape and separating independent clones (recommended). The fraction of remaining healthy alleles is visualized in the lower plot. For this purpose, a mirrored presentation of clonal evolution in the bottom layout is plotted. By default, clones in this part of the plot are not colored, representing a starting position of 100% healthy alleles.

As an example, we consider linear clonal evolution of 2 clones—A and B. Clone A is characterized by a mutation in *TP53* and clone B by a deletion 17p. The 2 variants are overlapping. If they affect different alleles, no healthy allele of *TP53* remains in all cells belonging to clone B. Thus, a user may chose to color clone B in the lower plot to indicate a decrease in healthy alleles of *TP53* as the CCF of clone B increases. The clone should, however, be colored in the hue of clone A. Thereby, the biallelic event leading to *TP53* deficiency is linked to the clone that is originally characterized by a mutation in this gene. Instead, if both variants affect the same allele, 1 healthy copy of *TP53* remains—independent of the CCF of clones A and B. Thus, no clone should be colored in the lower plot. In addition to biallelic events, variants affecting the only available X- or Y-chromosome in male subjects can equally be visualized using plaice plots. Detailed information on the recommend color coding of plaice plots is provided in Additional File 1, Section 1.7.

Just like dolphin plots, plaice plots provide all options for visualizing data on additional interpolated time points (recommended) and estimated therapy effect.

### Comparison to common approaches

Several approaches exist for visualizing clonal evolution. A majority of tools performing clonal evolution tree reconstruction (e.g., PhyloWGS [15], Canopy [16], or TRaP [17]) provide a basic graph-based visualization that is automatically generated when performing the analysis. These plots, however, are commonly not customizable and will thus not be further considered in this work. Additionally, tools like BubbleTree [18] and AbsCN-seq [19], estimating and visualizing tumor purity, ploidy, and copy numbers, are not considered due to different scope. The tool MapScape [20] is not considered as visualization focuses on spatial clonal evolution, linking anatomical images to tumor samples.

A commonly used approach for visualizing clonal evolution by means of fish plots is the R package fishplot [13]. The R/Bioconductor package timescape [14] provides an alternative approach, visualizing clonal evolution by interactive fish plots linked to standard graphs. A detailed evaluation of both approaches in comparison to our novel approach clevRvis is performed.

We could not identify any tool visualizing biallelic events in clonal evolution for comparison with our plaice plot module. Therefore, representation by plaice plots is compared to dolphin/fish plots as well as manual evaluation of biallelic events.

## Results

We apply clevRvis to 4 real, publicly available datasets and compare performance of our approach to the commonly used R packages fishplot [13] and timescape [14]. Results for 3 exemplary samples are visualized in Fig. 2. Detailed results, considering all 31 samples, are available in Additional File 1 (dataset 1: Supplementary Supplementary Figs. S11–S21; dataset 2: Supplementary Supplementary Figs. S22, S23; dataset 3: Supplementary Supplementary Figs. S24–S34; dataset 4: Supplementary Supplementary Figs. S35–S45). Main analysis features of all 3 algorithms are summed up in Table 2.

**Figure 2:**
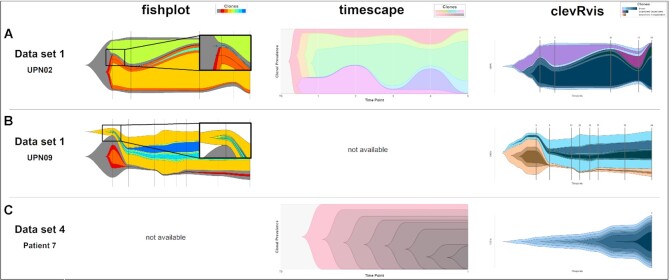
Visualization of clonal evolution using fish plots, comparing the approaches fishplot, timescape, and clevRvis. (A) Dataset 1, UPN02: branched dependent evolution can be visualized by all 3 tools. However, starting points of 2 clones are not correctly displayed using fishplot. (B) Dataset 1, UPN09: branched independent evolution can only be visualized by fishplot and clevRvis. The starting point of 1 clone is not correctly displayed using fishplot. (C) Dataset 4, patient 7: linear evolution based on a single time point can only be visualized by timescape and clevRvis.

**Table 2:** Overview of main analysis features, comparing the approaches fishplot [13], timescape [14], and clevRvis

	fishplot	timescape	clevRvis
**Visualization options**			
Graph		x	x
Fish plot	x	x	x
+ healthy alleles			x
**Clonal evolution models**			
Linear	x	x	x
Branched dependent	x	x	x
Branched independent	x		x
**Basic features**			
Graphical user interface			x
Validity check^1^	x		x
Interactive plots		x	x
Single time point		x	x
Single clone	x		x
**Advanced features**			
Exploring alternative trees			x
Time point interpolation			x
Therapy effect estimation			x
Phylogeny-aware color coding^2^			x

^1^We consider a validity check to be able to detect errors in the logic of clonal evolution (e.g., the CCF of a children-clone exceeding the CCF of its parent-clone or CCFs at 1 time point summing up to $> 100\%$).

^2^We consider phylogeny-aware color coding to indicate the degree of relatedness of 2 clones. This includes clones developing in a linear, branched dependent, and branched independent manner.

All 3 approaches are able to generate fish plots for visualization of clonal evolution (called “dolphin plots” in clevRvis). Additionally, timescape and clevRvis generate graphs (“shark plots” in clevRvis), representing the underlying phylogeny. However, clevRvis is the only approach providing an option to visualize information on healthy alleles and their development over time in terms of clonal evolution (“plaice plots”).

It can be observed that the 3 main models of clonal evolution—linear, branched dependent, and branched independent—can generally be considered by all 3 tools. The only exception is branched independent evolution, which cannot be visualized using timescape (Fig. 2B; dataset 1: patients UPN08, 09, 10, Supplementary Figs. S18–S20; dataset 3: patient 4, Supplementary Figs. S27–S29). The tool mandatorily requires all clones to be present in a single tree. Additionally, timescape is not capable of visualizing clonal evolution, if only a single clone is present (dataset 1: patient UPN04, Supplementary Fig. S14). Fishplot, on the contrary, struggles with visualizing data available at a single time point (Fig. 2C; dataset 3: patients 1–3, Supplementary Figs. S24–S26; dataset 4: patients 6–10, Supplementary Figs. S40–S45). Furthermore, the visualization with fishplot partly suggests a wrong starting point of the clone, despite correct definition of the input (Fig. 2A and B; dataset 1: patients UPN02, 03, 07, 09, 10, Supplementary Figs. S12, S13, S17, S19, S20; dataset 4: patients 2 and 5, Supplementary Figs. S36, S39).

clevRvis is the only approach providing a graphical user interface, allowing for user-friendly analysis of clonal evolution. Plots can be easily customized, for example, interactively moving labels of the clones along the x- and y-coordinates, or picking colors and transparency levels for the clones’ borders from a wide palette. By default, all plots are interactive. When hovering over a clone, its CCF is displayed and the clone is highlighted—in case of shark and dolphin plots, which are interactively connected, in both plots. Moreover, clevRvis is the only tool providing advanced features, exceeding the basic visualization of clonal evolution. These include fully automatic algorithms for time point interpolation and therapy effect estimation. Phylogeny-aware color coding is implemented as well as an algorithm for exploring alternative trees.

A detailed description on the usage of clevRvis, including exemplary input files and executable examples, is provided along with the package (manuals and vignette). A tutorial, including a complete walk-through, is additionally provided in the shiny-app.

### Time point interpolation and therapy effect estimation

**Figure 3:**
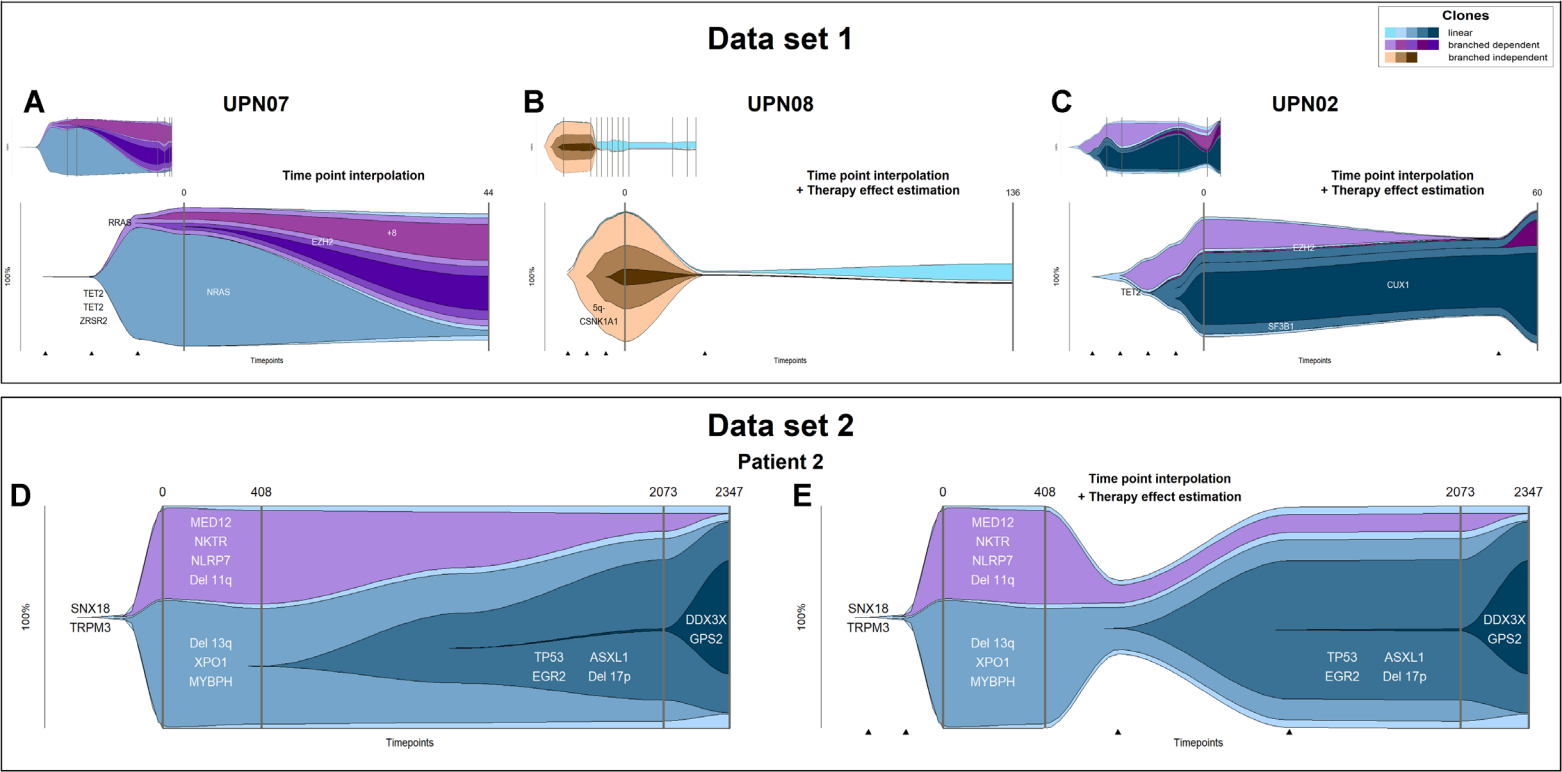
Visualization of clonal evolution using dolphin plots. (A) Dataset 1, UPN07: analysis of 6 time points vs. analysis of 2 time points and additional time point interpolation. (B) Dataset 1, UPN08: analysis of 12 measured time points vs. analysis of 2 measured time points, additional time point interpolation and therapy effect estimation. (C) Dataset 1, UPN02: analysis of 5 measured time points vs. analysis of 2 measured time points, additional time point interpolation and therapy effect estimation. (D) Dataset 2, patient 2: analysis of 4 measured time points. (E) Dataset 2, patient 2: analysis of 4 measured time points and additional therapy effect estimation. Note: for all plots, time point interpolation is enabled to improve visualization of newly developing clones prior to the first measured time point.

clevRvis provides algorithms for approximating the development of clonal evolution in between 2 measured time points, by interpolating additional time points as well as estimating the effect of a therapy applied. To investigate performance of our algorithms, we consider datasets 1 and 2. Results considering 4 exemplary patients are summed up in Fig. 3.

Patients in dataset 1 are characterized by a high number of measured time points (up to 30). Analysis with clevRvis is performed twice: (i) evaluating all measured time points and (ii) evaluating the first and last measured time points only. Six patients (UPN03, 04, 05, 06, 07, and 11) received supportive care only. Thus, development of clonal evolution is best approximated by time point interpolation. For patient UPN07 (Fig. 3A), it can be observed that despite a certain simplification in the development, clonal evolution estimated by clevRvis is highly comparable to the original course. Similar results can be observed for patient UPN08. As the patient was treated with lenalidomide, we compare clonal evolution based on 12 measured time points to the estimated development, based on only 2 measured time points + interpolated time points + estimated therapy effect (Fig. 3B).

For patient UPN02, also receiving treatment with lenalidomide, certain differences can be observed (Fig. 3C). The magenta clone can barely be observed in the development estimated by clevRvis (measured with $CCF=22\%$ at time point *t*_53_). However, as the clone is present with $CCF< 1\%$ at both *t*_0_ and *t*_60_, it appears basically impossible to predict its unexpected rise and subsequent fall at an intervening time point. Despite this apparent difference, the estimated clonal evolution reflects the main characteristics of the true clonal development of this patient (results for all patients available in Additional File 1, Supplementary Figs. S11–S21).

Dataset 2 contains information on 2 patients: patient 1 characterized by 15 and patient 2 characterized by 4 time points. Clonal evolution of patient 2, considering all available time points, is displayed in Fig. 3D. González-Rincón et al. [12] report that the sample at *t*_408_ was taken before treatment. Subsequently, the patient received treatment with FCR (fludarabin, cyclophosphamid, rituximab), followed by maintenance therapy with rituximab. The patient was reported to achieve complete response. However, clonal evolution only based on the measured time points does not reflect this response. From the results published, we estimate that FCR was given for roughly 1 year. Therefore, we estimate therapy effect for *t*_700_. The resulting plot in Fig. 3E shows a considerable effect of therapy on clonal evolution, matching the described course of disease (results for both patients available in Additional File 1, Supplementary Figs. S22, S23).

### Detecting biallelic events

**Figure 4:**
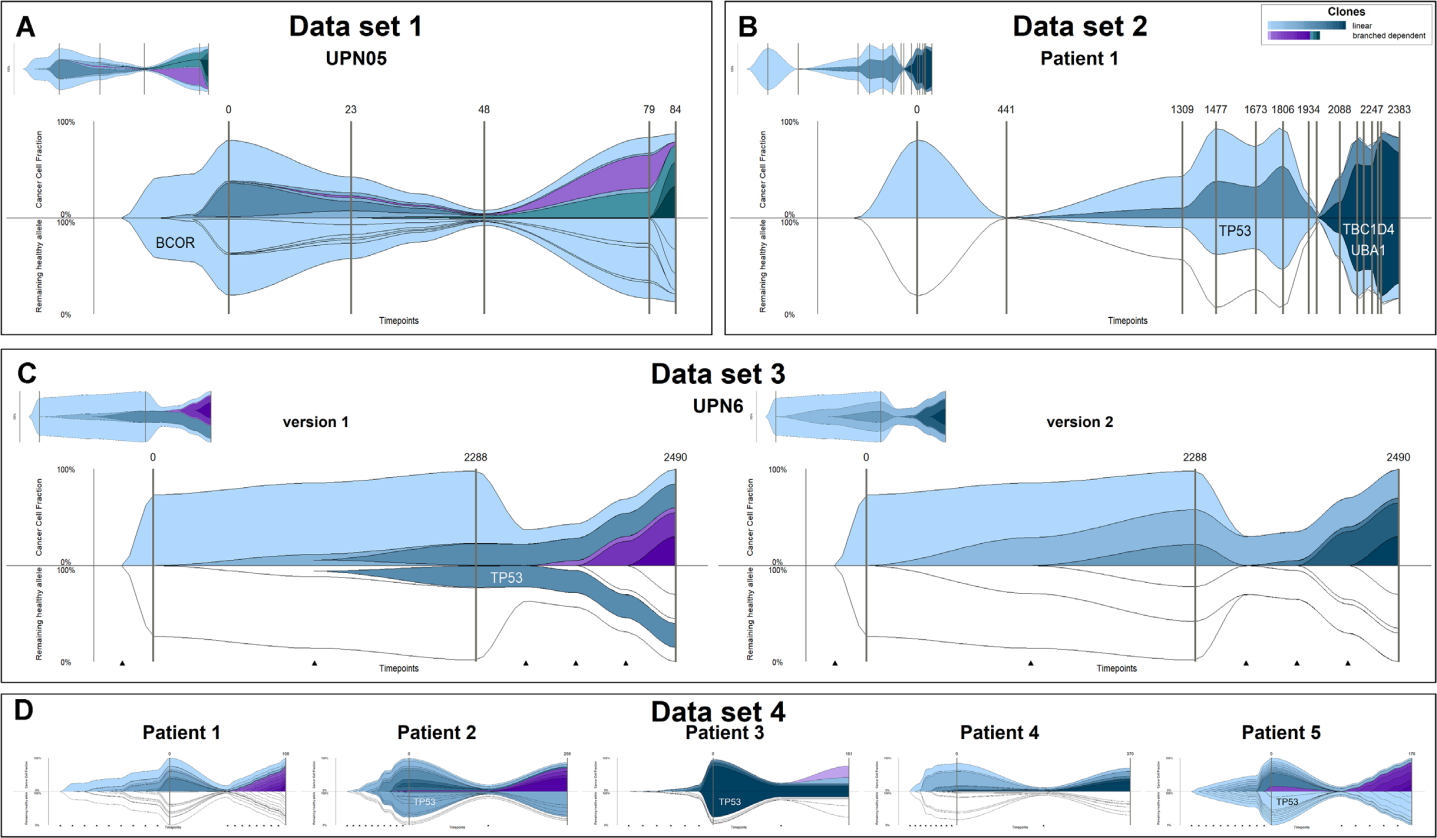
Visualization of clonal evolution using plaice plots vs. dolphin plots. (A) Dataset 1, UPN05: a hemizygous variant affects *BCOR*. (B) Dataset 2, patient 1: a biallelic event causes *TP53* deficiency in clone 2. Clone 3 is characterized by additional *TBC1D4* and *UBA1* deficiency. (C) Dataset 3, UPN06 (enabled therapy effect estimation): a double-hit event affects *TP53* (der(17)t(13;17)(q21;p12) + point mutation). Clonal evolution cannot be reconstructed uniquely. Version 1 assumes branched dependent evolution, leading to *TP53* deficiency in 1 clone. Version 2 assumes linear clonal evolution, leaving at least 1 healthy allele of *TP53* in each clone. (D) Dataset 4, patients 1 to 5: different biallelic events lead to *TP53* deficiency in patients 2, 3, and 5. Note: for all plots, time point interpolation is enabled to improve visualization of newly developing clones prior to the first measured time point.

clevRvis contains a novel plotting option for clonal evolution—plaice plots. These plots allow for identification of biallelic events. Considering datasets 1 to 4, we investigate applicability and added value of an analysis by plaice plots. Results considering 8 exemplary patients are summed up in Fig. 4.

Patient UPN05 in dataset 1 is characterized by branched dependent evolution, with a total of 7 clones. The first clone features, among others, a point mutation in *BCOR*. The gene is located on the X-chromosome. As the patient is male, the hemizygous variant leads to a loss of the only available copy of *BCOR*. In the plaice plot, the first and all subsequent clones are marked, indicating a missing healthy allele of *BCOR* (Fig. 4A) (plaice plots for all patients in dataset 1 available in Additional File 1, Supplementary Figs. S11–S21).

Patient 1 in dataset 2 features a splicing variant in *TP53* (Fig. 4B; light blue clone). Subsequently, the patient acquires a deletion in chromosome 17 (17p13.1 del), overlapping *TP53*. The CNV is clustered in clone 2 (intermediate blue). As this event leads to a loss of the only available allele of *TP53*, clone 2 is marked in the plaice plot. Light blue—the color of clone 1, characterized by the initial variant in *TP53*—is chosen for coloring. Additionally, clone 3 (dark blue) features deficient *TBC1D4* (13q14.3 del + point mutation) and *UBA1* (X-chromosomal variant in a male patient) (plaice plots for both patients in dataset 2 available in Additional File 1, Supplementary Figs. S22, S23).

For patient UPN06 in dataset 3, clonal evolution cannot be reconstructed uniquely (Fig. 4C). The patient features 2 variants affecting *TP53*: a point mutation (p.Val272Met) and a derivative chromosome 17 (der(17)t(13;17)(q21;p12)). Data do not allow for deciphering which of the 2 variants developed first. Branched dependent (version 1) and linear (version 2) evolution can be reconstructed. In addition to the difference in clonal evolution model, the effect on *TP53* differs considerably: in version 1, plaice plots show *TP53* deficiency in 22–25% of the cells. In version 2, on the contrary, all cells contain at least 1 healthy copy of *TP53* throughout the entire period of follow-up (plaice plots for all patients in dataset 3 available in Additional File 1, Supplementary Figs. S24–S34).

Relapsing patients in dataset 4 (patients 1 to 5) are—different from nonrelapse patients—characterized by ≥2 variants affecting *TP53*. Analysis and visualization with plaice plots show that patients 2, 3, and 5 are characterized by deficient *TP53* in a majority of cells (Fig. 4D). The stem line of patient 1 is characterized by a point mutation in *TP53* (p.Arg248Gln) and an overlapping CNV. The duplication affects the mutated allele of *TP53*, but a ratio of 1:2 for healthy/mutated remains. Thus, no clone is marked in the lower plaice plot. For patient 4, a point mutation in *TP53* (p.Arg248Gln) is detected in the second to last clone. A CNV below detection thresholds is assumed to be additionally present. However, data do not allow to decide on whether it is a deletion or duplication. Thus, it is unclear whether a healthy copy of *TP53* remains (plaice plots for all patients in dataset 4 available in Additional File 1, Supplementary Figs. S35–S45).

## Discussion

clevRvis is an R/Bioconductor package, providing innovative visualization techniques for clonal evolution. The optimized, highly customizable implementation of established visualization approaches (shark plots, dolphin plots) is complemented by a unique allele-aware representation of clonal evolution, allowing for analysis of biallelic events at a glance: plaice plots. In addition, the tool contains fully automatic algorithms for time point interpolation, therapy effect estimation, and phylogeny-aware color coding, exploring alternative trees as well as a graphical user interface for intuitive usage not just by computer scientists but also by biologists and physicians.

To our knowledge, only 2 alternative approaches for visualizing clonal evolution exist: fishplot [13] and timescape [14]. With respect to functionalities, our novel approach unites all options of currently available tools and provides a wide set of additional features. Analyzing 4 publicly available datasets, it can be observed that plots generated with clevRvis allow for an improved visualization of clonal evolution, outperforming both fishplot and timescape. Furthermore, new insights into disease course can be gained and reasons explored for therapy failure and relapse.

As regards suitable input data, the analysis of clonal evolution faces a major challenge: commonly, the number of tumor samples available heavily depends on the tumor itself. For nonsolid tumors, bone marrow or peripheral blood is commonly analyzed (e.g., [6, 8]). Taking into account a patient’s burden, performing regular bone marrow biopsies is ethically difficult to justify, especially toward the end of a therapy that is expected to lead to remission. For solid tumors (e.g., brain tumors), collecting samples after therapy is practically impossible as long as no relapse is observed. Thus, valid approaches estimating the development of a tumor and its response to therapy are of high relevance.

The 2 algorithms for time point interpolation and therapy effect estimation, inspired by the general idea outlined by Reutter et al. [8], mark a central element of clevRvis. However, the assumptions on which these algorithms are based can be discussed.

Interpolating initial development of a tumor toward the first measured time point *t*_1_, we focus on the difference in CCFs (*ccf*^′^). If *ccf*^′^ of clone A (=stemline) is 20% at *t*_1_, we assume that it is also 20% at every interpolated initial time point. It is, of course, possible that clone A temporarily reaches values $> 20\%$ and is—toward *t*_1_—pushed away by clone B, resulting in a decrease of *ccf*^′^. On the contrary, it is also possible that clone A expands only slowly ,and values $< 20\%$ are observed prior to *t*_1_. As we could not find evidence for 1 of the 2 scenarios being generally more likely, we decided—as a compromise—to focus only on *ccf*^′^ at *t*_1_, which may result in an underestimation in some cases and an overestimation in other cases.

In the absence of therapy, it appears sensible to assume that the overall tumor load never decreases. Interpolating development of a tumor between 2 measured time points with no therapy being applied, we therefore assume linear development of CCFs. While focusing on the difference in CCFs would be a valid alternative approach for clones showing an increase in *ccf*^′^, it can lead to a violation of our main assumption in case of decreasing *ccf*^′^ (new, quickly expanding clones pushing away existing clones). As the overall tumor load is not expected to decrease at any (interpolated) time point, we decided to stick to linear development for all clones. New clones are assumed to develop successively, based on their nested level.

In the presence of therapy, we assume that every observable decrease in CCF is related to therapy. Focusing again on the difference in CCFs, the minimum *ccf*^′^ of the measured time points prior to (*t_i_*) and after (*t*_*i* + 1_) estimated therapy effect is considered. If a decrease in *ccf*^′^ can be observed, we assume that it is caused by therapy. If an increase is observed, we assume that the clone is resistant to therapy and was able to expand after the end of therapy. Similar to interpolation of the initial development, it is possible that this approach overestimates therapy effect in some cases while underestimating it in others. We consider our approach a compromise, approximating true development in a majority of cases.

Due to limited data being available (e.g., dataset 4), we could not prove validity of our algorithms for all patients. However, evaluating 2 public datasets, we could show that our algorithms indeed provide valid solutions for interpolating time points and estimating therapy effect. Partly, they even revealed new insights into tumor development (dataset 2, patient 2; Fig. 3D vs. 3E). While our analysis also showed that the extreme development of a clone (e.g., a considerable increase followed by subsequent decrease) in between 2 measured time points cannot be approximated, it remains questionable whether any algorithm would be capable of predicting this unexpected behavior in the lack of sufficient data.

In addition to approximating a tumor’s development, the analysis of clonal evolution on the allele level represents another central aspect of clevRvis. While biallelic events are considered of high relevance, their analysis commonly requires tedious manual inspection of the variants characterizing each clone. Considering CNVs, genetic expert knowledge is often required to decipher partly complex karyotypes. Additionally, it may be necessary to consider raw sequencing data. While specific clones featuring biallelic events could also be highlighted in a common fish plot, our newly developed plaice plots provide a unique option to (i) easily convey information on biallelic events and (ii) link this information to characteristic clones by suitable color coding. As shown in Fig. 4B (dataset 2, patient 1), this does not necessarily refer to the same clone. It may of course be argued that it is still necessary to manually define the clones to color in the lower plaice plot once. Subsequently, however, this information can be evaluated at a glance by physicians, biologists, and even computer scientists.

Compared to dolphin plots, it may be considered a disadvantage that the actual clonal evolution is only displayed in the upper half of a plaice plot, making complex clonal evolution patterns potentially difficult to see. However, real examples of complex clonal evolution (e.g., dataset 1, UPN10: 8 clones, dependent and independent branches, Additional File 1, Supplementary Fig. S20C vs. S20D; dataset 4, patient 5: 17 clones, linear development, Additional File 1, Supplementary Fig. S39D vs. S39E) show that all clones can still be distinguished clearly. Simulated data, considering even more complex clonal evolution (100 clones and 100 time points; Additional File 1, Section 2.5), show that all plot types implemented in clevRvis—including plaice plots—allow for visualization of a high number of clones as well as a high number of time points.

## Conclusion

clevRvis provides an extensive set of visualization techniques for clonal evolution. Exceeding currently available approaches, clevRvis allows for approximating a tumor’s development in between measured time points as well as analyzing biallelic events. Our future work will include an extension of the clevRvis package, including approaches for considering multiple spatial locations per time point, as well as visualizing changes in gene expression and methylation along common clonal evolution, based on a tumor’s mutational profile.

## Availability of Source Code and Requirements

- Project name: clevRvis
- Project homepage: https://github.com/sandmanns/clevRvis
- Bioconductor: https://bioconductor.org/packages/clevRvis
- Operating system(s): Platform independent
- Programming language: R
- Other requirements: None
- License: LGPL-3.0
- bio.tools ID: biotools:clevRvis
- SciCrunch ID: RRID:SCR_023154

## Supplementary Material

giad020_GIGA-D-22-00248_Original_Submission

giad020_GIGA-D-22-00248_Revision_1

giad020_GIGA-D-22-00248_Revision_2

giad020_Response_to_Reviewer_Comments_Original_Submission

giad020_Response_to_Reviewer_Comments_Revision_1

giad020_Reviewer_1_Report_Original_Submission Christopher Miller -- 10/25/2022 Reviewed

giad020_Reviewer_2_Report_Original_Submission Zuguang Gu -- 11/3/2022 Reviewed

giad020_Reviewer_3_Report_Original_Submission Mohammed El-Kebir -- 11/8/2022 Reviewed

giad020_Reviewer_3_Report_Revision_1 Mohammed El-Kebir -- 2/19/2023 Reviewed

giad020_Supplemental_File

## Data Availability

An archival copy of the code and supporting data is available via the *GigaScience* repository, GigaDB [21]. Further material supporting the results of this article is available in Additional File 1.

## Additional Files

SupplementaryInformation.pdf: information on supplementary methods and results.

**Supplementary Fig. S1**. Start screen for the analysis with clevRvis.

**Supplementary Fig. S2**. Defining input for the analysis with clevRvis. A table containing the cancer cell fractions characterizing every clone at every time point may be uploaded. Subsequently, parental relations have to be defined interactively.

**Supplementary Fig. S3**. Defining input for the analysis with clevRvis. Alternatively, a table containing the cancer cell fractions characterizing every clone at every time point as well as parental information may be uploaded.

**Supplementary Fig. S4**. Defining the seaObject for the analysis with clevRvis. Optionally, additional time points may be interpolated, and therapy effect may be estimated. The seaObject table contains information on the cancer cell fractions of all clones, at all measured and estimated time points. Additionally, information on parental relations is included in the table.

**Supplementary Fig. S5**. Customized interactive shark plot generated with clevRvis. When hovering over a clone in the graph on the left side, the clone’s label is displayed. Additionally, all points on the right side, corresponding to the cancer cell fractions (measured and if selected also estimated), are highlighted. When hovering over a point on the right side, the precise cancer cell fraction is displayed. Additionally, the clone in the graph on the left side is highlighted. If additional dolphin plots are displayed, the corresponding clone is also highlighted in the dolphin plot.

**Supplementary Fig. S6**. Customized interactive dolphin plot generated with clevRvis. When hovering over a clone, the clone’s label is displayed and the CCF development highlighted. If additional shark plots are displayed, the corresponding clone is also highlighted in the shark plot.

**Supplementary Fig. S7**. Customized interactive plaice plot generated with clevRvis. When hovering over a clone, the clone’s label is displayed and the CCF development highlighted.

**Supplementary Fig. S8**. Tutorial describing the use of clevRvis.

**Supplementary Fig. S9**. Visualization of clonal evolution in an exemplary simulated patient. (A) clevRvis using dolphin plots, enabling time point interpolation. By default, interpolated time points at −50, −25, and 25 are added. (B) clevRvis using dolphin plots, enabling time point interpolation. Interpolated time point 25 was manually changed to 40 to visualize a skewed event (development of clone 6).

**Supplementary Fig. S10**. Recommended color coding for plaice plots considering chromosome 17 (red mark indicating mutation in TP53). (A) No clone in the lower plaice is colored since 1 copy of a healthy allele remains for all genes. (B) Clone 2 in the lower plaice is colored in the same hue as clone 1 in the upper plaice, which is characterized by mutated TP53. (C) Clone 2 in the lower plaice is colored in the same hue as clone 2 in the upper plaice, which is characterized by mutated TP53. (D) Clone 2 in the lower plaice is colored in the same hue as clone 2 in the upper plaice, which is characterized by 2 mutations in TP53.

**Supplementary Fig. S11**. Visualization of clonal evolution of UPN01 in dataset 1. (A) Fishplot. (B) Timescape. (C) clevRvis using dolphin plots and extended shark plots, enabling time point interpolation for improved visualization of newly developing clones. (D) clevRvis using dolphin plots and extended shark plots, enabling time point interpolation and therapy effect estimation, analyzing only the first and last measured time points. (E) clevRvis using plaice plots, enabling time point interpolation. A biallelic event affecting RAD50 (del(5q) + point mutation) is marked.

**Supplementary Fig. S12**. Visualization of clonal evolution of UPN02 in dataset 1. (A) Fishplot. (B) Timescape. (C) clevRvis using dolphin plots and extended shark plots, enabling time point interpolation for improved visualization of newly developing clones. (D) clevRvis using dolphin plots and extended shark plots, enabling time point interpolation and therapy effect estimation, analyzing only the first and last measured time points. (E) clevRvis using plaice plots, enabling time point interpolation. Two biallelic events affecting ODZ2 (2 point mutations) and TET2 (LOH(4q) + point mutation) are marked.

**Supplementary Fig. S13**. Visualization of clonal evolution of UPN03 in dataset 1. (A) Fishplot. (B) Timescape. (C) clevRvis using dolphin plots and extended shark plots, enabling time point interpolation for improved visualization of newly developing clones. (D) clevRvis using dolphin plots and extended shark plots, enabling time point interpolation, analyzing only the first and last measured time points. (E) clevRvis using plaice plots, enabling time point interpolation. A biallelic event affecting ZNF609 (2 point mutations) is marked.

**Supplementary Fig. S14**. Visualization of clonal evolution of UPN04 in dataset 1. (A) Fishplot. (B) clevRvis using dolphin plots and extended shark plots, enabling time point interpolation for improved visualization of newly developing clones. (C) clevRvis using dolphin plots and extended shark plots, enabling time point interpolation, analyzing only the first and last measured time points. (D) clevRvis using plaice plots, enabling time point interpolation. A biallelic event affecting TET2 (2 point mutations) and a hemizygous variant affecting ZRSR2 are marked. Note: Development of a single clone cannot be visualized using timescape.

**Supplementary Fig. S15**. Visualization of clonal evolution of UPN05 in dataset 1. (A) Fishplot. (B) Timescape. (C) clevRvis using dolphin plots and extended shark plots, enabling time point interpolation for improved visualization of newly developing clones. (D) clevRvis using dolphin plots and extended shark plots, enabling time point interpolation, analyzing only the first and last measured time points. (E) clevRvis using dolphin plots and extended shark plots, enabling time point interpolation and therapy effect estimation, analyzing only the first and last measured time points. (F) clevRvis using plaice plots, enabling time point interpolation and therapy effect estimation. A hemizygous variant affecting BCOR is marked. Note: Patient UPN05 is reported to have only received supportive care. The figure published by da Silva-Coelho [1] matches version (D). However, reconstructing the CCF of each clone based on the precise variant calling information published by da Silva-Coelho [1] results in version (C), indicating therapy effect.

**Supplementary Fig. S16**. Visualization of clonal evolution of UPN06 in dataset 1. (A) Fishplot. (B) Timescape. (C) clevRvis using dolphin plots and extended shark plots, enabling time point interpolation for improved visualization of newly developing clones. (D) clevRvis using dolphin plots and extended shark plots, enabling time point interpolation, analyzing only the first and last measured time points. (E) clevRvis using plaice plots, enabling time point interpolation. A point mutation in RUNX1 is overlapping trisomy 21, and a point mutation in UBR5 is overlapping trisomy 8. At least 1 healthy allele remains for both genes. Thus, no clone is marked.

**Supplementary Fig. S17**. Visualization of clonal evolution of UPN07 in dataset 1. (A) Fishplot. (B) Timescape. (C) clevRvis using dolphin plots and extended shark plots, enabling time point interpolation for improved visualization of newly developing clones. (D) clevRvis using dolphin plots and extended shark plots, enabling time point interpolation, analyzing only the first and last measured time points. (E) clevRvis using plaice plots, enabling time point interpolation. A biallelic event affecting TET2 (2 point mutations) and a hemizygous variant affecting ZRSR2 are marked.

**Supplementary Fig. S18**. Visualization of clonal evolution of UPN08 in dataset 1. (A) Fishplot. (B) clevRvis using dolphin plots and extended shark plots, enabling time point interpolation for improved visualization of newly developing clones. (C) clevRvis using dolphin plots and extended shark plots, enabling time point interpolation and therapy effect estimation, analyzing only the first and last measured time points. (D) clevRvis using plaice plots, enabling time point interpolation and therapy effect estimation. A biallelic event affecting CSNK1A1 (del(5q) + point mutation) is marked. Note: Independent evolution cannot be visualized using timescape.

**Supplementary Fig. S19**. Visualization of clonal evolution of UPN09 in dataset 1. (A) Fishplot. (B) clevRvis using dolphin plots and extended shark plots, enabling time point interpolation for improved visualization of newly developing clones. (C) clevRvis using dolphin plots and extended shark plots, enabling time point interpolation and therapy effect estimation, analyzing only the first and last measured time points. (D) clevRvis using plaice plots, enabling time point interpolation and therapy effect estimation. As no biallelic events are present, no clone is marked. Note: Independent evolution cannot be visualized using timescape.

**Supplementary Fig. S20**. Visualization of clonal evolution of UPN10 in dataset 1. (A) Fishplot. (B) clevRvis using dolphin plots and extended shark plots, enabling time point interpolation for improved visualization of newly developing clones. (C) clevRvis using dolphin plots and extended shark plots, enabling time point interpolation and therapy effect estimation, analyzing only the first and last measured time points. (D) clevRvis using plaice plots, enabling time point interpolation and therapy effect estimation. As no biallelic events are present, no clone is marked. Note: Independent evolution cannot be visualized using timescape.

**Supplementary Fig. S21**. Visualization of clonal evolution of UPN11 in dataset 1. (A) Fishplot. (B) Timescape. (C) clevRvis using dolphin plots and extended shark plots, enabling time point interpolation for improved visualization of newly developing clones. (D) clevRvis using dolphin plots and extended shark plots, enabling time point interpolation, analyzing only the first and last measured time points. (E) clevRvis using plaice plots, enabling time point interpolation. A point mutation in ARHGAP36 is overlapping dup(21q). As at least 1 healthy allele remains in each clone, no clone is marked.

**Supplementary Fig. S22**. Visualization of clonal evolution of patient 1 in dataset 2. (A) Fishplot. (B) Timescape. (C) clevRvis using dolphin plots and extended shark plots, enabling time point interpolation for improved visualization of newly developing clones. (D) clevRvis using dolphin plots and extended shark plots, enabling time point and first therapy effect estimation, analyzing only the first and last measured time points. (E) clevRvis using dolphin plots and extended shark plots, enabling time point and second therapy effect estimation, analyzing only the first and last measured time points. (F) clevRvis using plaice plots, enabling time point interpolation. Biallelic events affecting TP53 (17p13.1 del + point mutations; light blue), TBC1D4 (13q14.3 del + point mutation; dark blue), and a hemizygous variant on UBA1 (point mutation; dark blue) are marked.

**Supplementary Fig. S23**. Visualization of clonal evolution of patient 2 in dataset 2. (A) Fishplot. (B) Timescape. (C) clevRvis using dolphin plots and extended shark plots, enabling time point interpolation for improved visualization of newly developing clones. (D) clevRvis using dolphin plots and extended shark plots, enabling time point interpolation and therapy effect estimation, analyzing only the first and last measured time points. (E) clevRvis using plaice plots, enabling time point interpolation and therapy effect estimation. Biallelic events affecting TP53 (17p13.1 del + point mutations; intermediate blue) and GPS2 (17p13.1 del + point mutations; dark blue) are marked. Additionally, hemizygous variants on MED12 (purple), ZCCHC16 (intermediate blue), and DDX3X (dark blue) are marked.

**Supplementary Fig. S24**. Visualization of clonal evolution of UPN1 in dataset 3. (A) Timescape. (B) clevRvis using dolphin plots and extended shark plots, enabling time point interpolation for improved visualization of newly developing clones. (C) clevRvis using dolphin plots and extended shark plots, enabling time point interpolation and therapy effect estimation. (D) clevRvis using plaice plots, enabling time point interpolation and therapy effect estimation. A biallelic event affecting TP53 (deletion chromosome 17 + point mutation in TP53) is marked. Note: A single time point cannot be visualized using fishplot.

**Supplementary Fig. S25**. Visualization of clonal evolution of UPN2 in dataset 3. (A) Timescape. (B) clevRvis using dolphin plots and extended shark plots, enabling time point interpolation for improved visualization of newly developing clones. (C) clevRvis using dolphin plots and extended shark plots, enabling time point interpolation and therapy effect estimation. (D) clevRvis using plaice plots, enabling time point interpolation and therapy effect estimation. Hemizygous variants affecting BCOR and STAG2, leading to a loss of the only available healthy allele, are marked. Note: A single time point cannot be visualized using fishplot.

**Supplementary Fig. S26**. Visualization of clonal evolution of UPN3 in dataset 3. (A) Timescape. (B) clevRvis using dolphin plots and extended shark plots, enabling time point interpolation for improved visualization of newly developing clones. (C) clevRvis using dolphin plots and extended shark plots, enabling time point interpolation and therapy effect estimation. (D) clevRvis using plaice plots, enabling time point interpolation. As all clones feature at least 1 healthy allele of every gene, no clone is marked. Note: A single time point cannot be visualized using fishplot.

**Supplementary Fig. S27**. Visualization of clonal evolution of UPN4 (version 1) in dataset 3. (A) Fishplot. (B) clevRvis using dolphin plots and extended shark plots, enabling time point interpolation for improved visualization of newly developing clones. (C) clevRvis using dolphin plots and extended shark plots, enabling time point interpolation and therapy effect estimation. (D) clevRvis using plaice plots, enabling time point interpolation and therapy effect estimation. A biallelic event affecting TET2 (2 point mutations) is marked. Note: Independent evolution cannot be visualized using timescape.

**Supplementary Fig. S28**. Visualization of clonal evolution of UPN4 (version 2) in dataset 3. (A) Fishplot. (B) clevRvis using dolphin plots and extended shark plots, enabling time point interpolation for improved visualization of newly developing clones. (C) clevRvis using dolphin plots and extended shark plots, enabling time point interpolation and therapy effect estimation. (D) clevRvis using plaice plots, enabling time point interpolation and therapy effect estimation. A biallelic event affecting TET2 (2 point mutations) is marked. Note: Independent evolution cannot be visualized using timescape.

**Supplementary Fig. S29**. Visualization of clonal evolution of UPN4 (version 3) in dataset 3. (A) Fishplot. (B) clevRvis using dolphin plots and extended shark plots, enabling time point interpolation for improved visualization of newly developing clones. (C) clevRvis using dolphin plots and extended shark plots, enabling time point interpolation and therapy effect estimation. (D) clevRvis using plaice plots, enabling time point interpolation and therapy effect estimation. A biallelic event affecting TET2 (2 point mutations) is marked. Note: Independent evolution cannot be visualized using timescape.

**Supplementary Fig. S30**. Visualization of clonal evolution of UPN5 in dataset 3. (A) Fishplot. (B) Timescape. C) clevRvis using dolphin plots and extended shark plots, enabling time point interpolation for improved visualization of newly developing clones. (D) clevRvis using dolphin plots and extended shark plots, enabling time point interpolation and therapy effect estimation. (E) clevRvis using plaice plots, enabling time point interpolation and therapy effect estimation. As all clones feature at least 1 healthy allele of every gene, no clone is marked.

**Supplementary Fig. S31**. Visualization of clonal evolution of UPN6 (version 1) in dataset 3. (A) Fishplot. B) Timescape. (C) clevRvis using dolphin plots and extended shark plots, enabling time point interpolation for improved visualization of newly developing clones. (D) clevRvis using dolphin plots and extended shark plots, enabling time point interpolation and therapy effect estimation. (E) clevRvis using plaice plots, enabling time point interpolation and therapy effect estimation. A biallelic event affecting TP53 (derivative chromosome 17 der(17)t(13;17)(q21;p12) + point mutation in TP53) is marked.

**Supplementary Fig. S32**. Visualization of clonal evolution of UPN6 (version 2) in dataset 3. (A) Fishplot. (B) Timescape. (C) clevRvis using dolphin plots and extended shark plots, enabling time point interpolation for improved visualization of newly developing clones. (D) clevRvis using dolphin plots and extended shark plots, enabling time point interpolation and therapy effect estimation. (E) clevRvis using plaice plots, enabling time point interpolation and therapy effect estimation. It is assumed that der(17)t(13;17)(q21;p12) affects the allele already affected by a point mutation in TP53. Thus, a healthy copy of the gene remains, and no clone is marked.

**Supplementary Fig. S33**. Visualization of clonal evolution of UPN7 in dataset 3. (A) Fishplot. (B) Timescape. (C) clevRvis using dolphin plots and extended shark plots, enabling time point interpolation for improved visualization of newly developing clones. (D) clevRvis using plaice plots, enabling time point interpolation. As all clones feature at least 1 healthy allele of every gene, no clone is marked.

**Supplementary Fig. S34**. Visualization of clonal evolution of UPN8 in dataset 3. (A) Fishplot. (B) Timescape. (C) clevRvis using dolphin plots and extended shark plots, enabling time point interpolation for improved visualization of newly developing clones. (D) clevRvis using plaice plots, enabling time point interpolation and therapy effect estimation. A biallelic event affecting NRAS (2 point mutations) is marked.

**Supplementary Fig. S35**. Visualization of clonal evolution of patient 1 in dataset 4. (A) Fishplot. (B) Timescape. (C) clevRvis using dolphin plots and extended shark plots, enabling time point interpolation for improved visualization of newly developing clones. (D) clevRvis using dolphin plots and extended shark plots, enabling time point interpolation and therapy effect estimation. (E) clevRvis using plaice plots (only focusing on TP53), enabling time point interpolation and therapy effect estimation. Two variants affect TP53 (point mutation + duplication). However, 1 healthy copy of the gene remains. Thus, no clone is marked.

**Supplementary Fig. S36**. Visualization of clonal evolution of patient 2 in dataset 4. (A) Fishplot. (B) Timescape. (C) clevRvis using dolphin plots and extended shark plots, enabling time point interpolation for improved visualization of newly developing clones. (D) clevRvis using dolphin plots and extended shark plots, enabling time point interpolation and therapy effect estimation. (E) clevRvis using plaice plots (only focusing on TP53), enabling time point interpolation and therapy effect estimation. A biallelic event affecting TP53 (2 point mutations) is marked.

**Supplementary Fig. S37**. Visualization of clonal evolution of patient 3 in dataset 4. (A) Fishplot. (B) Timescape. (C) clevRvis using dolphin plots and extended shark plots, enabling time point interpolation for improved visualization of newly developing clones. (D) clevRvis using dolphin plots and extended shark plots, enabling time point interpolation and therapy effect estimation. (E) clevRvis using plaice plots (only focusing on TP53), enabling time point interpolation and therapy effect estimation. A biallelic event affecting TP53 (loss of heterozygosity + point mutation in TP53) is marked.

**Supplementary Fig. S38**. Visualization of clonal evolution of patient 4 in dataset 4. (A) Fishplot. (B) Timescape. (C) clevRvis using dolphin plots and extended shark plots, enabling time point interpolation for improved visualization of newly developing clones. (D) clevRvis using dolphin plots and extended shark plots, enabling time point interpolation and therapy effect estimation. (E) clevRvis using plaice plots (only focusing on TP53), enabling time point interpolation and therapy effect estimation. A point mutation affects TP53. Additionally, a CNV below detection threshold is assumed to be present. However, as it is unclear whether it is a deletion or a duplication, no clone is marked.

**Supplementary Fig. S39**. Visualization of clonal evolution of patient 5 in dataset 4. (A) Fishplot. (B) Timescape. (C) clevRvis using dolphin plots and extended shark plots, enabling time point interpolation for improved visualization of newly developing clones. (D) clevRvis using dolphin plots and extended shark plots, enabling time point interpolation and therapy effect estimation. (E) clevRvis using plaice plots (only focusing on TP53), enabling time point interpolation and therapy effect estimation. A biallelic event affecting TP53 (loss of heterozygosity + point mutation in TP53) is marked.

**Supplementary Fig. S40**. Visualization of clonal evolution of patient 6 in dataset 4. (A) Timescape. (B) clevRvis using dolphin plots and extended shark plots, enabling time point interpolation for improved visualization of newly developing clones. (C) clevRvis using dolphin plots and extended shark plots, enabling time point interpolation and therapy effect estimation. (D) clevRvis using plaice plots (only focusing on TP53), enabling time point interpolation and therapy effect estimation. As all clones feature at least 1 healthy allele of TP53, no clone is marked. Note: A single time point cannot be visualized using fishplot.

**Supplementary Fig. S41**. Visualization of clonal evolution of patient 7 in dataset 4. (A) Timescape. (B) clevRvis using dolphin plots and extended shark plots, enabling time point interpolation for improved visualization of newly developing clones. (C) clevRvis using dolphin plots and extended shark plots, enabling time point interpolation and therapy effect estimation. (D) clevRvis using plaice plots (only focusing on TP53), enabling time point interpolation and therapy effect estimation. As all clones feature at least 1 healthy allele of TP53, no clone is marked. Note: A single time point cannot be visualized using fishplot.

**Supplementary Fig. S42**. Visualization of clonal evolution of patient 8 (version 1) in dataset 4. (A) Timescape. (B) clevRvis using dolphin plots and extended shark plots, enabling time point interpolation for improved visualization of newly developing clones. (C) clevRvis using dolphin plots and extended shark plots, enabling time point interpolation and therapy effect estimation. (D) clevRvis using plaice plots (only focusing on TP53), enabling time point interpolation and therapy effect estimation. As all clones feature at least 1 healthy allele of TP53, no clone is marked. Note: A single time point cannot be visualized using fishplot.

**Supplementary Fig. S43**. Visualization of clonal evolution of patient 8 (version 2) in dataset 4. (A) Timescape. (B) clevRvis using dolphin plots and extended shark plots, enabling time point interpolation for improved visualization of newly developing clones. (C) clevRvis using dolphin plots and extended shark plots, enabling time point interpolation and therapy effect estimation. (D) clevRvis using plaice plots (only focusing on TP53), enabling time point interpolation and therapy effect estimation. As all clones feature at least 1 healthy allele of TP53, no clone is marked. Note: A single time point cannot be visualized using fishplot.

**Supplementary Fig. S44**. Visualization of clonal evolution of patient 9 in dataset 4. (A) Timescape. (B) clevRvis using dolphin plots and extended shark plots, enabling time point interpolation for improved visualization of newly developing clones. (C) clevRvis using dolphin plots and extended shark plots, enabling time point interpolation and therapy effect estimation. (D) clevRvis using plaice plots (only focusing on TP53), enabling time point interpolation and therapy effect estimation. As all clones feature at least 1 healthy allele of TP53, no clone is marked. Note: A single time point cannot be visualized using fishplot.

**Supplementary Fig. S45**. Visualization of clonal evolution of patient 10 in dataset 4. (A) Timescape. (B) clevRvis using dolphin plots and extended shark plots, enabling time point interpolation for improved visualization of newly developing clones. (C) clevRvis using dolphin plots and extended shark plots, enabling time point interpolation and therapy effect estimation. (D) clevRvis using plaice plots (only focusing on TP53), enabling time point interpolation and therapy effect estimation. As all clones feature at least 1 healthy allele of TP53, no clone is marked. Note: A single time point cannot be visualized using fishplot.

**Supplementary Fig. S46**. Visualization of clonal evolution of an exemplary case with 100 clones. (A) clevRvis using shark plots. (B) clevRvis using dolphin plots, enabling time point interpolation for improved visualization of newly developing clones. (C) clevRvis using plaice plots, enabling time point interpolation.

**Supplementary Fig. S47**. Visualization of clonal evolution of an exemplary case with 100 time points. (A) clevRvis using extended shark plots. (B) clevRvis using dolphin plots, enabling time point interpolation for improved visualization of newly developing clones. (C) clevRvis using plaice plots, enabling time point interpolation.

## Abbreviations

aCGH: array-CGH; BL: Burkitt lymphoma; CCF: cancer cell fraction; CNV: copy number variant; indels: insertions/deletions; FCR: fludarabin; cyclophosphamid; rituximab; FISH: fluorescence in situ hybridization; MDS: myelodysplastic syndromes; SNP: single-nucleotide polymorphism; tNGS: targeted next-generation sequencing; VAF: variant allele frequency; WES: whole-exome sequencing.
