# Supplementary material for: clevRvis: visualization techniques for clonal evolution
Source: Gigascience. 2023 Apr 11;12:giad020. doi: 10.1093/gigascience/giad020 (PMC10087014; doi:10.1093/gigascience/giad020)
Supplement: giad020_GIGA-D-22-00248_Revision_1 [file giad020_giga-d-22-00248_revision_1.pdf]

GigaScience
clevRvis: Visualization Techniques for Clonal Evolution
--Manuscript Draft--

**Manuscript Number:**	GIGA-D-22-00248R1
**Full Title:**	clevRvis: Visualization Techniques for Clonal Evolution
**Article Type:**	Technical Note
**Funding Information:**	Open Access Publication Fund — Not applicable
**Abstract:**	Background: A thorough analysis of clonal evolution commonly requires integration of diverse sources of data, e.g. karyotyping, next-generation sequencing and clinical information. Subsequent to actual reconstruction of clonal evolution, detailed analysis and interpretation of the results is essential. Often, however, only few tumor samples per patient are available. Thus, information on clonal development and therapy effect may be incomplete. Furthermore, analysis of bi-allelic events - considered of high relevance with respect to disease course - can commonly only be realized by time-consuming analysis of the raw results and even raw sequencing data. Results: We developed clevRvis, an R/Bioconductor package providing an extensive set of visualization techniques for clonal evolution. In addition to common approaches for visualization, clevRvis offers a unique option for allele-aware representation: plaice plots. Bi-allelic events may be visualized and inspected at a glance. Analyzing four public data sets, we show that plaice plots help to gain new insights into tumor development and investigate hypotheses on disease progression and therapy resistance. In addition to a graphical user interface, automatic phylogeny-aware color coding of the plots and an approach to explore alternative trees, clevRvis provides two algorithms for fully automatic time point interpolation and therapy effect estimation. Analyzing two public data sets, we show that both approaches allow for valid approximation of a tumor's development in between measured time points. Conclusions: clevRvis represents a novel option for user-friendly analysis of clonal evolution, contributing to gaining new insights into tumor development.
**Corresponding Author:**	Sarah Sandmann<br>Westfälische Wilhelms-Universität Münster<br>Münster, GERMANY
**Corresponding Author Secondary Information:**	
**Corresponding Author's Institution:**	Westfälische Wilhelms-Universität Münster
**Corresponding Author's Secondary Institution:**	
**First Author:**	Sarah Sandmann
**First Author Secondary Information:**	
**Order of Authors:**	Sarah Sandmann
	Clara Inserte
	Julian Varghese
**Order of Authors Secondary Information:**	
**Response to Reviewers:**	Editor:<br><br>1) Please include a link to an RCRAN/bioconductor version of the tool in the revised paper.<br><br>Changes: clevRvis was successfully submitted to Bioconductor and accepted as a package (https://bioconductor.org/packages/clevRvis). The clevRvis-package is currently available at Bioconductor's development-branch and is expected to be included in the Bioconductor 3.17 Release in April/May 2023. The link was included in the manuscript.

Powered by Editorial Manager® and ProduXion Manager® from Aries Systems Corporation

2) Reviewer #1 also has found some issues with the code which will need to be looked into carefully.

Changes: We carefully revised our code and examples. We added additional exemplary code. Furthermore, we extended our documentation on how to execute the examples, differentiating between examples for the “function-version” of clevRvis and the “shiny-version”.

3) In addition, please register any new software application in the bio.tools and SciCrunch.org databases to receive RRID (Research Resource Identification Initiative ID) and biotoolsID identifiers, and include these in your manuscript. Computational workflows should be registered in workflowhub.eu and the DOIs cited in the relevant places in the manuscript. These will facilitate tracking, reproducibility and re-use of your tool.

Changes: We registered clevRvis at bio.tools and SciCrunch.org, and added information on the biotools ID and the SciCrunch RRID to our manuscript.

We had a detailed look at workflowhub.eu and currently available workflows. To the best of our knowledge, only comprehensive workflows – partly using one or more R packages – are currently available at this website. R packages that stand alone, like clevRvis, we could not identify. Therefore, we opted against uploading clevRvis to workflowhub.eu for the moment.

Reviewer #1:

1) I do wonder if replacing "timepoint estimation" with "timepoint interpolation" might make things a little more clear. This tool isn't somehow gaining insight into non-represented intermediate timepoints, it's just evenly spacing events/changes throughout the missing time. That's certainly a fine (and very useful) approach for visualization purposes, but I initially assumed it was doing something more complicated! To be clear, I am not arguing that this has to be changed in the manuscript, but it's something to consider.

Changes: We changed “time point estimation” to “time point interpolation” – in the manuscript, the documentation of clevRvis and the functions.

2) I also think that at some future point (also not necessary for this manuscript), it might be nice to include options to the timepoint "estimation" that would allow events to be skewed left or right, if the user has belief that mutations were acquired early or late in the period between timepoints.

Changes: We added a detailed description and exemplary code to the supplement, describing how clevRvis' time point interpolation can be used to visualize skewed events (Additional File 1, section 1.6 “Using time point interpolation to implement skewed events”).

3) It took a while to wrap my head around the coloring of the plaice plots (e.g. Figure 3b). The match in color between the TP53 deletion (in clone 2) and the TP53 mutation (in clone 1) is a bit confusing at first. (why is clone 2's shape colored with clone 1's color?) While I understand now, and don't have a specific suggestion for how to improve it, it's something to ponder.

Changes: We added the section “Recommended color coding for plaice plots” to the supplement (Additional File 1, section 1.7), suggesting rules for coloring the “lower plaice” and providing a visual example with additional explanation.

Powered by Editorial Manager® and ProduXion Manager® from Aries Systems Corporation

4) With the version that I pulled (commit 83508c3 from Sep 14), man pages seemed to be missing, such that typing `?sharkPlot` or `?createSeaObject` didn't display the expected help text. That'd be a useful addition.

Changes: clevRvis is now available as a Bioconductor package (https://bioconductor.org/packages/clevRvis). Manual pages for all functions should now be accessible, typing e.g. `?sharkPlot` or `?createSeaObject'.

5) I tried to convert the example frac.table and parents info from github into an example csv and load it into the shiny app, but was unable to get it to run.

Changes: We carefully revised our code and examples. We added additional exemplary code. Furthermore, we extended our documentation on how to execute the examples, differentiating between examples for the "function-version" of clevRvis and the "shiny-version". Additionally, we added an error message if a user provides non-numeric time points as input.

We are very sorry for the problems and the confusion that arose from the exemplary data we provided. The exemplary code available in the vignette (parents, fracTable etc.) was intended exclusively for use of the "function-version" of clevRvis. Of course, the fracTable we provide may be converted to e.g. a CSV file and taken as input for clevRvis. However, the reason why the error message "NAs are not allowed in subscripted assignments" appeared is as follows: by default, clevRvis assums that the first row contains information on the time points (if this option is de-selected, the first row is assumed to contain CCFs of a clone). However, these time points are required to be numeric so that the subsequent algorithms for time point interpolation and therapy effect estimation do work. By changing the CSV file to

```
"","1","2","3"
"1",20,40,50
"2",10,20,25
"3",0,15,15
"4",0,0,10
"5",0,30,40
"6",0,10,20
"7",0,0,15
```

or – alternatively – leaving out the first line and selecting "Use default time points? (1,2…)", everything should work as expected.
We added a code example to our vignette (section clevRvisShiny() > Examples > Input – without parental relations and Input – with parental relations) on how to generate CSV files for use in clevRvisShiny() based on a fracTable matrix, a timepoints vector and optionally a parents vector.
We completely agree with the reviewer that this could have caused several support tickets in the future. Therefore, we added an automatic check to our "shiny-version" on whether the first row contains numeric values and – if this is not the case – a meaningful error message appears.

6) Outside of the GUI, when I ran `dolphinPlot(seaObject)` with the example data from github, I got output, but also a warning. These appear non-fatal, but it would be nice to suppress them or generate a more informative error if is something is actually wrong here.

Changes: The warnings are non-fatal. We had a look at all warnings reported by clevRvis and removed the reasons – wherever it was possible – or included suppressWarnings() otherwise.

Reviewer #2:

Powered by Editorial Manager® and ProduXion Manager® from Aries Systems Corporation

	1) I am wondering why authors did not format the tool as an R package, while a list of R script? For unexperienced users, it might be difficult to directly use the R scripts. I saw the documentation of functions is already in vignettes/clevR-vis.html, so with the package "devtools", I think it should be very easy to turn it into an R package.<br><br>Changes: clevRvis was successfully submitted to Bioconductor and accepted as a package (https://bioconductor.org/packages/clevRvis). The clevRvis-package is currently available at Bioconductor's development-branch and is expected to be included in the Bioconductor 3.17 Release in April/May 2023.<br><br><br>2) The shiny app looks great. I think it can also be integrated in the package. I tried to use the shiny app with the two example files provided (example1.xls and example2.xls). I can see the files are imported, but no plot is shown. I guess it is because the example files are too simple. I would suggest authors to provide a complete example file, also in the shiny app, to add a new button "load example" in the "Inputs" tab, so that users can know the format of the input file and fast try the shiny app.<br><br>Changes: We carefully revised our code and examples. Especially, we extended our documentation on how to execute the examples using the “shiny-version” of clevRvis. Furthermore, messages were added to the “seaObject table”-tab [Please upload a Cancer Cell Fractions table first ("Inputs" → "Submit inputs"). Then, configure the seaObject ("seaObject options" → "Submit") to visualize the seaObject Cancer Cell Fractions table.] and the “Plots”-tab [Please configure the seaObject first ("seaObject options" → "Submit"). Then, select the plots to visualize ("Plots" → switches for Shark, Dolphin and Plaice Plots)] if prerequisites for generating plots are not yet given.<br><br>We are very sorry for the inconveniences the reviewer experienced when testing clevRvis. Both files Example1.xlsx and Example2.xlsx should contain fully functional examples for the analysis with clevRvis. From what the reviewer describes, it could be that the exemplary files were successfully imported, but no seaObject was generated afterwards. When uploading an input file, it is automatically displayed as a datatable in the input tab on the right. Subsequently, a user has to define the parental relations (information available as a table at the end of the vignette; update now: parental relations can also be uploaded directly, using one of the new, exemplary files Example1_withParents.xlsx and Example2_withParents. xlsx that are provided along with the package). As soon as information on parental relations has been provided, a user has to click “Submit inputs” to execute the validity check. If a success message is displayed, one may move on to the “seaObject options”. Even if “Extra time point interpolation” and “Therapy effect estimation” are not enabled, a user has to click “Submit” to generate a seaObject table (shown in the tab “seaObject table” on the right). Then, finally, plots can be generated and inspected in the “Plots”-tab.<br><br><br>3) In the shiny app, I saw the relations of parent-child clones should be manually selected. Maybe authors can allow to upload the relations as a second table file?<br><br>Changes: The upload options have been extended. Optionally, a user may now upload a CCF table (clones x time points) additionally containing a “parents” column with information on the parental relations. Information is automatically imported. Still, a CCF table not containing any parent information can be uploaded and the relations defined manually.<br><br><br>4) If authors plan to submit the R package to CRAN or Bioconductor, "clevR-vis" might need to be changed to e.g. "clevRvis" or maybe "clever"? Hyphen is not allowed in the package name on these two repositories.<br><br>Changes: We changed the name of our algorithm and the R package to clevRvis.<br><br><br>5) Last, in Figure 2 and 3, the legends are missing. Maybe add a global legend for all panels?

Changes: We added global legends to Figures 2 and 3 (now: Figures 3 and 4).

Reviewer #3:

1) Comparison against timescape and fishplot: Differences between clevR-vis and the two previous methods (timescape and fishplot) should be illustrated using real data. Also, the differences between a fishplot and dolphin plot are not clear to me. As far as I can tell, the visualization is identical, barring small diferences in how clones are ordered and the use of colormaps. clevR-vis does include interpolation, which might lead to smoother fishplots. I encourage the authors to better illustrate the differences.

Changes: We revised our manuscript and added more information on the comparison of clevRvis vs fishplot and timescape, especially focusing on additional features only provided by clevRvis. We added Figure 2 to the Results section, highlighting differences in the fish plots generated by the three approaches, considering 3 exemplary patients (real data). Evaluation of real data from all 31 patients is available in Additional File 1 (Figures S11-S45).

For the visualization of clonal evolution, we consider fish plots being the most established solution, as they unite information on phylogeny, CCFs and time course in a single elegant plot. By our approach, clevRvis, we aimed at providing an extensive set of visualization techniques for clonal evolution. Therefore, clevRvis also contains an implementation of fish plots. We decided to call them “dolphin plots” as we liked to stick to the “sea”-naming and – in comparison to the rather raw graph (=shark) plots – they appeared to us a more “friendly” visualization, which lead us to the name “dolphin plots”.
The reviewer is thoroughly right in noticing that fish plots, as implemented in the fishplot or timescape package, and dolphin plots contain the same basis. However, we aimed at optimizing the visualization (e.g. by automatic phylogeny-aware color coding, showing the correct starting point for all clones, single time point and single clone visualization being supported, providing an option to switch between centered and bottom layout, providing interactive plots and interactive annotation). Furthermore, clevRvis contains several unique features, e.g. shark plots and extended shark plots, plaice plots, algorithms for time point interpolation and therapy effect estimation, a graphical user interface and – now added – an option to explore alternative trees.
We hope to show that clevRvis offers an optimized version of the established visualization of clonal evolution, as well as several novel visualization techniques and features for advanced analysis of clonal evolution.

2) Better description of methods: There are three steps that need to be described more carefully in the Methods part of the paper: (i) estimation of initial CCFs, (ii) estimation of additional timepoints and CCF interpolation, (iii) estimation of tumor load. For step (i) no description in Methods is given. It is also not clear how many additional timepoints are added. For (iii), it is not clear how tumor load is calculated for previous and subsequent timepoints prior and after treatment. Pseudocode is not an alternative to a precise textual description.

Changes: We revised the description of our Algorithm 3 “time point interpolation”, adding further details. Especially, information was added on how the number of interpolated time points is defined. Furthermore, we added a detailed description of Algorithm 4 “therapy effect estimation”. Of note, as the functionality of clevRvis was extended, Algorithm 2 “exploring alternative trees” was added to the Methods section (pseudocode + detailed description).

3) Usefulness of plaice plots: The paper motivates the use of plaice plots to visualize bi-allelic events. An example of such an event is two hits of TP53, where one allelic copy is deleted and the other copy undergoes a truncating mutation. I am not convinced that such events warrant a new type of visualization. A disadvantage of plaice plots is that one loses half the visualization real estate due to mirroring along the

Powered by Editorial Manager® and ProduXion Manager® from Aries Systems Corporation

x-axis. The more general feature seems to be highlighting/emphasizing clones that contain a pre-specified set of mutations. This can be accomplished in the original fishplot using for instance edge color.

Changes: We carefully revised our manuscript and added a discussion on the potential difficulty to see complex clonal evolution patterns in plaice plots vs dolphin plots. Furthermore, we outline the added value of plaice plots compared to marking clones affected by bi-allelic events in common fish plots. Plaice plots provide the unique additional option to link bi-allelic events to characteristic clones by suitable color-coding. We added the section “Recommended color coding for plaice plots” to the supplement (Additional File 1, section 1.7), suggesting rules for coloring the “lower plaice” and providing a visual example with additional explanation.

4) Lack of interactivity: A missed opportunity is that the visualizations are mostly static. Interactivity would greatly improve the tool. For instance, filtering based on mutations, listing clone proportions (ccf(parent) - sum ccfs of children), exploring alternative tree solutions.

Changes: Interactivity of clevRvis was extended: For the input, now, a user has the option to explore alternative tree solutions (available in the shiny-app + as individual new function exploreTrees()). For the seaObject, now, a user may easily switch between visualization of “CCF_clone” vs “CCF_clone – sum(CCF_children)”. For the plots, clevRvis still offers the option of interactive plots (available in the shiny-app + in the individual plot functions).

We would like to thank the reviewer for his very valuable ideas of increasing clevRvis' level of interactivity! For now, we opted against including an option to filter based on mutations. clevRvis does – in its current version – solely work on information characterizing clones. We assume that a user initially performs clustering of mutations to determine clones and their CCFs using other established approaches. However, we hope to extend our “clevR” software suite in near future, developing e.g. “clevRclust” for this analysis step. For the commonly subsequent analysis step – reconstructing clonal evolution trees, which also includes exploring alternative trees – we extended our clevRvis package. Taking information on the available time points and CCFs of each clone at each time point as input, our novel exploreTrees()-function investigates possible, alternative parental relations and reports all valid solutions, passing clevRvis' validity check. Results are available in a drop-down menu (shiny-version) or as a list (function-version). Subsequently, a user may select any of these parental relations for visualization with clevRvis.

5) Examples in R package, bioconductor/cran: To facilitate reproduction and also ease of use, I encourage the authors to save the seaObjects corresponding to the real data figures as .Rdata files. Moreover, it might be good to include one or two examples in the library. Finally, I recommend publishing this on bioconductor/cran.

Changes: seaObjects for all real data figures are now available as file “data.Rdata” at Giga DB. clevRvis was successfully submitted to Bioconductor and accepted as a package (https://bioconductor.org/packages/clevRvis). The clevRvis-package is currently available at Bioconductor's development-branch and is expected to be included in the Bioconductor 3.17 Release in April/May 2023. Examples are included in the functions' manual pages as well as in the vignette. For the ”shiny-version” of clevRvis, we provide 4 exemplary xlsx-files (2 exemplary patients, each with and without information on parental relations included in the file). Furthermore, the vignette contains exemplary code to generate a CSV file for the analysis with clevRvisShiny(), based the fracTable matrix, a time points vector and optionally a parents vector.

6) Another quality of life improvement is that the tree itself should be taken as input. Setting edges in the UI is cumbersome.

Changes: The upload options have been extended. Optionally, a user may now upload a CCF table (clones x time points) additionally containing a “parents” column with

	information on the parental relations. Information is automatically imported. Still, a CCF table not containing any parent information can be uploaded and the relations defined manually.<br><br>7) List limitations: Finally, I'd like to see a discussion of limitations. For instance, how many clones do you support? How many timepoints? How realistic is the timepoint estimation? What about multiple spatial locations per timepoint?<br><br>Changes: We added section 2.5 “Visualizing complex cases of clonal evolution” to Additional file 1, considering visualization of a simulated data set with 100 clones, and another set with 100 time points using clevRvis. The results are considered in our Discussion. Additionally, we revised our Discussion adding the limitation that, due to limited data being available, we could not check validity of our algorithms interpolating time points and estimating therapy effect for all patients. However, analysis of the two real data sets 1 and 2 showed that our algorithms generate realistic results. Considering multiple spatial locations per time point was added as future work to our Conclusions.
Additional Information:	
Question	Response
Are you submitting this manuscript to a special series or article collection?	No
**Experimental design and statistics**<br><br>Full details of the experimental design and statistical methods used should be given in the Methods section, as detailed in our Minimum Standards Reporting Checklist. Information essential to interpreting the data presented should be made available in the figure legends.<br><br>Have you included all the information requested in your manuscript?	No
If not, please give reasons for any omissions below.<br><br>as follow-up to "**Experimental design and statistics**<br><br>Full details of the experimental design and statistical methods used should be given in the Methods section, as detailed in our Minimum Standards Reporting Checklist. Information essential to interpreting the data presented should be made available in the figure legends.	Our methods section contains a detailed description of all analyses we performed and all necessary information to reproduce our analyses. However, as we only analyzed public data sets, no information on e.g. power calculation or how samples were collected is included.

Have you included all the information requested in your manuscript?<br><br>"	
**Resources**<br><br>A description of all resources used, including antibodies, cell lines, animals and software tools, with enough information to allow them to be uniquely identified, should be included in the Methods section. Authors are strongly encouraged to cite Research Resource Identifiers (RRIDs) for antibodies, model organisms and tools, where possible.<br><br>Have you included the information requested as detailed in our Minimum Standards Reporting Checklist?	Yes
**Availability of data and materials**<br><br>All datasets and code on which the conclusions of the paper rely must be either included in your submission or deposited in publicly available repositories (where available and ethically appropriate), referencing such data using a unique identifier in the references and in the "Availability of Data and Materials" section of your manuscript.<br><br>Have you have met the above requirement as detailed in our Minimum Standards Reporting Checklist?	Yes

 Click here to access/download;LaTeX - Main Document (TeX file);main.tex

```
This is pdfTeX, Version 3.141592653-2.6-1.40.24 (TeX Live 2022)
(preloaded format=pdflatex 2022.10.28)  23 JAN 2023 06:05
entering extended mode
 restricted \write18 enabled.
 %&-line parsing enabled.
**main.tex
(./main.tex
LaTeX2e <2022-06-01> patch level 5
L3 programming layer <2022-10-26> (./oup-contemporary.cls
Document Class: oup-contemporary 2017/06/28, v1.1
(c:/TeXLive/2022/texmf-dist/tex/latex/base/article.cls
Document Class: article 2022/07/02 v1.4n Standard LaTeX document class
(c:/TeXLive/2022/texmf-dist/tex/latex/base/size10.clo
File: size10.clo 2022/07/02 v1.4n Standard LaTeX file (size option)
)
\c@part=\count185
\c@section=\count186
\c@subsection=\count187
\c@subsubsection=\count188
\c@paragraph=\count189
\c@subparagraph=\count190
\c@figure=\count191
\c@table=\count192
\abovecaptionskip=\skip47
\belowcaptionskip=\skip48
\bibindent=\dimen138
) (c:/TeXLive/2022/texmf-dist/tex/latex/base/inputenc.sty
Package: inputenc 2021/02/14 v1.3d Input encoding file
\inpenc@prehook=\toks16
\inpenc@posthook=\toks17
) (c:/TeXLive/2022/texmf-dist/tex/latex/base/fontenc.sty
Package: fontenc 2021/04/29 v2.0v Standard LaTeX package
) (c:/TeXLive/2022/texmf-dist/tex/generic/iftex/ifpdf.sty
Package: ifpdf 2019/10/25 v3.4 ifpdf legacy package. Use iftex instead.
(c:/TeXLive/2022/texmf-dist/tex/generic/iftex/iftex.sty
Package: iftex 2022/02/03 v1.0f TeX engine tests
)) (c:/TeXLive/2022/texmf-dist/tex/latex/microtype/microtype.sty
Package: microtype 2022/06/23 v3.0f Micro-typographical refinements (RS)
(c:/TeXLive/2022/texmf-dist/tex/latex/graphics/keyval.sty
Package: keyval 2022/05/29 v1.15 key=value parser (DPC)
\KV@toks@=\toks18
) (c:/TeXLive/2022/texmf-dist/tex/latex/etoolbox/etoolbox.sty
Package: etoolbox 2020/10/05 v2.5k e-TeX tools for LaTeX (JAW)
\etb@tempcnta=\count193
)
\MT@toks=\toks19
\MT@tempbox=\box51
\MT@count=\count194
LaTeX Info: Redefining \noprotrusionifhmode on input line 1045.
LaTeX Info: Redefining \leftprotrusion on input line 1046.
LaTeX Info: Redefining \rightprotrusion on input line 1056.
LaTeX Info: Redefining \textls on input line 1234.
\MT@outer@kern=\dimen139
LaTeX Info: Redefining \textmicrotypecontext on input line 1858.
```

```
\MT@listname@count=\count195
(c:/TeXLive/2022/texmf-dist/tex/latex/microtype/microtype-pdftex.def
File: microtype-pdftex.def 2022/06/23 v3.0f Definitions specific to
pdftex (RS)

LaTeX Info: Redefining \lsstyle on input line 900.
LaTeX Info: Redefining \lslig on input line 900.
\MT@outer@space=\skip49
)
Package microtype Info: Loading configuration file microtype.cfg.
(c:/TeXLive/2022/texmf-dist/tex/latex/microtype/microtype.cfg
File: microtype.cfg 2022/06/23 v3.0f microtype main configuration file
(RS)
)) (c:/TeXLive/2022/texmf-dist/tex/latex/euler/euler.sty
Package: euler 1995/03/05 v2.5
Package: `euler' v2.5 <1995/03/05> (FJ and FMi)
LaTeX Font Info:    Redeclaring symbol font `letters' on input line 35.
LaTeX Font Info:    Encoding `OML' has changed to `U' for symbol font
(Font)              `letters' in the math version `normal' on input line
35.
LaTeX Font Info:    Overwriting symbol font `letters' in version `normal'
(Font)                  OML/cmm/m/it --> U/eur/m/n on input line 35.
LaTeX Font Info:    Encoding `OML' has changed to `U' for symbol font
(Font)              `letters' in the math version `bold' on input line
35.
LaTeX Font Info:    Overwriting symbol font `letters' in version `bold'
(Font)                  OML/cmm/b/it --> U/eur/m/n on input line 35.
LaTeX Font Info:    Overwriting symbol font `letters' in version `bold'
(Font)                  U/eur/m/n --> U/eur/b/n on input line 36.
LaTeX Font Info:    Redeclaring math symbol \Gamma on input line 47.
LaTeX Font Info:    Redeclaring math symbol \Delta on input line 48.
LaTeX Font Info:    Redeclaring math symbol \Theta on input line 49.
LaTeX Font Info:    Redeclaring math symbol \Lambda on input line 50.
LaTeX Font Info:    Redeclaring math symbol \Xi on input line 51.
LaTeX Font Info:    Redeclaring math symbol \Pi on input line 52.
LaTeX Font Info:    Redeclaring math symbol \Sigma on input line 53.
LaTeX Font Info:    Redeclaring math symbol \Upsilon on input line 54.
LaTeX Font Info:    Redeclaring math symbol \Phi on input line 55.
LaTeX Font Info:    Redeclaring math symbol \Psi on input line 56.
LaTeX Font Info:    Redeclaring math symbol \Omega on input line 57.
\symEulerFraktur=\mathgroup4
LaTeX Font Info:    Overwriting symbol font `EulerFraktur' in version
`bold'
(Font)                  U/euf/m/n --> U/euf/b/n on input line 63.
LaTeX Info: Redefining \oldstylenums on input line 85.
\symEulerScript=\mathgroup5
LaTeX Font Info:    Overwriting symbol font `EulerScript' in version
`bold'
(Font)                  U/eus/m/n --> U/eus/b/n on input line 93.
LaTeX Font Info:    Redeclaring math symbol \aleph on input line 97.
LaTeX Font Info:    Redeclaring math symbol \Re on input line 98.
LaTeX Font Info:    Redeclaring math symbol \Im on input line 99.
LaTeX Font Info:    Redeclaring math delimiter \vert on input line 101.
```

```
LaTeX Font Info:    Redeclaring math delimiter \backslash on input line
103.
LaTeX Font Info:    Redeclaring math symbol \neg on input line 106.
LaTeX Font Info:    Redeclaring math symbol \wedge on input line 108.
LaTeX Font Info:    Redeclaring math symbol \vee on input line 110.
LaTeX Font Info:    Redeclaring math symbol \setminus on input line 112.
LaTeX Font Info:    Redeclaring math symbol \sim on input line 113.
LaTeX Font Info:    Redeclaring math symbol \mid on input line 114.
LaTeX Font Info:    Redeclaring math delimiter \arrowvert on input line
116.
LaTeX Font Info:    Redeclaring math symbol \mathsection on input line
117.
\symEulerExtension=\mathgroup6
LaTeX Font Info:    Redeclaring math symbol \coprod on input line 125.
LaTeX Font Info:    Redeclaring math symbol \prod on input line 125.
LaTeX Font Info:    Redeclaring math symbol \sum on input line 125.
LaTeX Font Info:    Redeclaring math symbol \intop on input line 130.
LaTeX Font Info:    Redeclaring math symbol \ointop on input line 131.
LaTeX Font Info:    Redeclaring math symbol \braceld on input line 132.
LaTeX Font Info:    Redeclaring math symbol \bracerd on input line 133.
LaTeX Font Info:    Redeclaring math symbol \bracelu on input line 134.
LaTeX Font Info:    Redeclaring math symbol \braceru on input line 135.
LaTeX Font Info:    Redeclaring math symbol \infty on input line 136.
LaTeX Font Info:    Redeclaring math symbol \nearrow on input line 153.
LaTeX Font Info:    Redeclaring math symbol \searrow on input line 154.
LaTeX Font Info:    Redeclaring math symbol \nwarrow on input line 155.
LaTeX Font Info:    Redeclaring math symbol \swarrow on input line 156.
LaTeX Font Info:    Redeclaring math symbol \Leftrightarrow on input line
157.
LaTeX Font Info:    Redeclaring math symbol \Leftarrow on input line 158.
LaTeX Font Info:    Redeclaring math symbol \Rightarrow on input line
159.
LaTeX Font Info:    Redeclaring math symbol \leftrightarrow on input line
160.
LaTeX Font Info:    Redeclaring math symbol \leftarrow on input line 161.
LaTeX Font Info:    Redeclaring math symbol \rightarrow on input line
163.
LaTeX Font Info:    Redeclaring math delimiter \uparrow on input line
166.
LaTeX Font Info:    Redeclaring math delimiter \downarrow on input line
168.
LaTeX Font Info:    Redeclaring math delimiter \updownarrow on input line
170.
LaTeX Font Info:    Redeclaring math delimiter \Uparrow on input line
172.
LaTeX Font Info:    Redeclaring math delimiter \Downarrow on input line
174.
LaTeX Font Info:    Redeclaring math delimiter \Updownarrow on input line
176.
LaTeX Font Info:    Redeclaring math symbol \leftharpoonup on input line
177.
LaTeX Font Info:    Redeclaring math symbol \leftharpoondown on input
line 178.
```

```
LaTeX Font Info:    Redeclaring math symbol \rightharpoonup on input line
179.
LaTeX Font Info:    Redeclaring math symbol \rightharpoondown on input
line 180
.
LaTeX Font Info:    Redeclaring math delimiter \lbrace on input line 182.
LaTeX Font Info:    Redeclaring math delimiter \rbrace on input line 184.
\symcmmigroup=\mathgroup7
LaTeX Font Info:    Overwriting symbol font `cmmigroup' in version `bold'
(Font)                  OML/cmm/m/it --> OML/cmm/b/it on input line 200.
LaTeX Font Info:    Redeclaring math accent \vec on input line 201.
LaTeX Font Info:    Redeclaring math symbol \triangleleft on input line
202.
LaTeX Font Info:    Redeclaring math symbol \triangleright on input line
203.
LaTeX Font Info:    Redeclaring math symbol \star on input line 204.
LaTeX Font Info:    Redeclaring math symbol \lhook on input line 205.
LaTeX Font Info:    Redeclaring math symbol \rhook on input line 206.
LaTeX Font Info:    Redeclaring math symbol \flat on input line 207.
LaTeX Font Info:    Redeclaring math symbol \natural on input line 208.
LaTeX Font Info:    Redeclaring math symbol \sharp on input line 209.
LaTeX Font Info:    Redeclaring math symbol \smile on input line 210.
LaTeX Font Info:    Redeclaring math symbol \frown on input line 211.
LaTeX Font Info:    Redeclaring math accent \grave on input line 245.
LaTeX Font Info:    Redeclaring math accent \acute on input line 246.
LaTeX Font Info:    Redeclaring math accent \tilde on input line 247.
LaTeX Font Info:    Redeclaring math accent \ddot on input line 248.
LaTeX Font Info:    Redeclaring math accent \check on input line 249.
LaTeX Font Info:    Redeclaring math accent \breve on input line 250.
LaTeX Font Info:    Redeclaring math accent \bar on input line 251.
LaTeX Font Info:    Redeclaring math accent \dot on input line 252.
LaTeX Font Info:    Redeclaring math accent \hat on input line 254.
) (c:/TeXLive/2022/texmf-dist/tex/latex/merriweather/merriweather.sty
Package: merriweather 2022/09/20 (Bob Tennent) Supports
Merriweather(Sans) font
s for all LaTeX engines.
(c:/TeXLive/2022/texmf-dist/tex/generic/iftex/ifxetex.sty
Package: ifxetex 2019/10/25 v0.7 ifxetex legacy package. Use iftex
instead.
) (c:/TeXLive/2022/texmf-dist/tex/generic/iftex/ifluatex.sty
Package: ifluatex 2019/10/25 v1.5 ifluatex legacy package. Use iftex
instead.
) (c:/TeXLive/2022/texmf-dist/tex/latex/base/textcomp.sty
Package: textcomp 2020/02/02 v2.0n Standard LaTeX package
) (c:/TeXLive/2022/texmf-dist/tex/latex/xkeyval/xkeyval.sty
Package: xkeyval 2022/06/16 v2.9 package option processing (HA)
(c:/TeXLive/2022/texmf-dist/tex/generic/xkeyval/xkeyval.tex
(c:/TeXLive/2022/te
xmf-dist/tex/generic/xkeyval/xkvutils.tex
\XKV@toks=\toks20
\XKV@tempa@toks=\toks21
)
\XKV@depth=\count196
File: xkeyval.tex 2014/12/03 v2.7a key=value parser (HA)
```

```
)) (c:/TeXLive/2022/texmf-dist/tex/latex/base/fontenc.sty
Package: fontenc 2021/04/29 v2.0v Standard LaTeX package
) (c:/TeXLive/2022/texmf-dist/tex/latex/fontaxes/fontaxes.sty
Package: fontaxes 2020/07/21 v1.0e Font selection axes
LaTeX Info: Redefining \upshape on input line 29.
LaTeX Info: Redefining \itshape on input line 31.
LaTeX Info: Redefining \slshape on input line 33.
LaTeX Info: Redefining \swshape on input line 35.
LaTeX Info: Redefining \scshape on input line 37.
LaTeX Info: Redefining \sscshape on input line 39.
LaTeX Info: Redefining \ulcshape on input line 41.
LaTeX Info: Redefining \textsw on input line 47.
LaTeX Info: Redefining \textssc on input line 48.
LaTeX Info: Redefining \textulc on input line 49.
)) (c:/TeXLive/2022/texmf-dist/tex/latex/mathastext/mathastext.sty
Package: mathastext 2022/11/04 v1.3y Use the text font in math mode (JFB)
\mst@exists@muskip=\muskip16
\mst@forall@muskip=\muskip17
\mst@prime@muskip=\muskip18
\mst@do@nonletters=\toks22
\mst@do@easynonletters=\toks23
\mst@do@az=\toks24
\mst@do@AZ=\toks25
\symmtoperatorfont=\mathgroup8
\symmtletterfont=\mathgroup9
** ! and ?
** punctuation: , . : ; and \colon
LaTeX Info: Redefining \relbar on input line 844.
LaTeX Info: Redefining \rightarrowfill on input line 847.
LaTeX Info: Redefining \leftarrowfill on input line 852.
** + and =
LaTeX Info: Redefining \Relbar on input line 943.
** adding = ; and + to \nfss@catcodes
** parentheses ( ) [ ] and slash /
** alldelims: < > \backslash \setminus | \vert \mid \{ and \}
LaTeX Font Info:    Redeclaring math delimiter \backslash on input line
989.
LaTeX Font Info:    Redeclaring math symbol \setminus on input line 1001.
LaTeX Info: Redefining \models on input line 1010.
** \# \mathdollar \% \&
** \imath and \jmath
LaTeX Font Info:    Overwriting math alphabet `\mathnormalbold' in
version `nor
mal'
(Font)                  T1/Merriwthr-OsF/b/it --> T1/Merriwthr-OsF/b/it
on inpu
t line 2370.
LaTeX Font Info:    Overwriting math alphabet `\mathnormalbold' in
version `bol
d'
(Font)                  T1/Merriwthr-OsF/b/it --> T1/Merriwthr-OsF/b/it
on inpu
t line 2370.
```

```
LaTeX Font Info:    Overwriting symbol font `mtletterfont' in version
`normal'
(Font)                  T1/Merriwthr-OsF/m/it --> T1/Merriwthr-OsF/m/it
on inpu
t line 2370.
LaTeX Font Info:    Overwriting symbol font `mtletterfont' in version
`bold'
(Font)                  T1/Merriwthr-OsF/m/it --> T1/Merriwthr-OsF/b/it
on inpu
t line 2370.
LaTeX Font Info:    Overwriting symbol font `mtoperatorfont' in version
`normal
'
(Font)                  T1/Merriwthr-OsF/m/n --> T1/Merriwthr-OsF/m/n on
input
line 2370.
LaTeX Font Info:    Overwriting symbol font `mtoperatorfont' in version
`bold'
(Font)                  T1/Merriwthr-OsF/m/n --> T1/Merriwthr-OsF/b/n on
input
line 2370.
LaTeX Font Info:    Overwriting math alphabet `\Mathbf' in version
`normal'
(Font)                  T1/Merriwthr-OsF/b/n --> T1/Merriwthr-OsF/b/n on
input
line 2370.
LaTeX Font Info:    Overwriting math alphabet `\Mathbf' in version `bold'
(Font)                  T1/Merriwthr-OsF/b/n --> T1/Merriwthr-OsF/b/n on
input
line 2370.
LaTeX Font Info:    Overwriting math alphabet `\Mathit' in version
`normal'
(Font)                  T1/Merriwthr-OsF/m/it --> T1/Merriwthr-OsF/m/it
on inpu
t line 2370.
LaTeX Font Info:    Overwriting math alphabet `\Mathit' in version `bold'
(Font)                  T1/Merriwthr-OsF/m/it --> T1/Merriwthr-OsF/b/it
on inpu
t line 2370.
LaTeX Font Info:    Overwriting math alphabet `\Mathsf' in version
`normal'
(Font)                  T1/MerriwthrSans-OsF/m/n --> T1/MerriwthrSans-
OsF/m/n o
n input line 2370.
LaTeX Font Info:    Overwriting math alphabet `\Mathsf' in version `bold'
(Font)                  T1/MerriwthrSans-OsF/m/n --> T1/MerriwthrSans-
OsF/b/n o
n input line 2370.
LaTeX Font Info:    Overwriting math alphabet `\Mathtt' in version
`normal'
(Font)                  T1/lmtt/m/n --> T1/lmtt/m/n on input line 2370.
LaTeX Font Info:    Overwriting math alphabet `\Mathtt' in version `bold'
(Font)                  T1/lmtt/m/n --> T1/lmtt/b/n on input line 2370.
** Latin letters in the `normal' (resp. `bold') math versions are now
```

```
** set up to use the fonts T1/Merriwthr-OsF/m(b)/it
** Other characters (digits, ...) and \log-like names will be
** typeset with the n shape.
** \hbar
** minus as endash
** \HUGE has been (re)-defined.
** mathastext has declared larger sizes for subscripts.
** To keep LaTeX defaults, use option `defaultmathsizes'.
) (c:/TeXLive/2022/texmf-dist/tex/latex/relsize/relsize.sty
Package: relsize 2013/03/29 ver 4.1
) (c:/TeXLive/2022/texmf-dist/tex/latex/ragged2e/ragged2e.sty
Package: ragged2e 2022/11/13 v3.2 ragged2e Package
\CenteringLeftskip=\skip50
\RaggedLeftLeftskip=\skip51
\RaggedRightLeftskip=\skip52
\CenteringRightskip=\skip53
\RaggedLeftRightskip=\skip54
\RaggedRightRightskip=\skip55
\CenteringParfillskip=\skip56
\RaggedLeftParfillskip=\skip57
\RaggedRightParfillskip=\skip58
\JustifyingParfillskip=\skip59
\CenteringParindent=\skip60
\RaggedLeftParindent=\skip61
\RaggedRightParindent=\skip62
\JustifyingParindent=\skip63
) (c:/TeXLive/2022/texmf-dist/tex/latex/xcolor/xcolor.sty
Package: xcolor 2022/06/12 v2.14 LaTeX color extensions (UK)
(c:/TeXLive/2022/texmf-dist/tex/latex/graphics-cfg/color.cfg
File: color.cfg 2016/01/02 v1.6 sample color configuration
)
Package xcolor Info: Driver file: pdftex.def on input line 227.
(c:/TeXLive/2022/texmf-dist/tex/latex/graphics-def/pdftex.def
File: pdftex.def 2022/09/22 v1.2b Graphics/color driver for pdftex
\stockwidth=\dimen140
\stockheight=\dimen141
) (c:/TeXLive/2022/texmf-dist/tex/latex/graphics/mathcolor.ltx)
Package xcolor Info: Model `cmy' substituted by `cmy0' on input line
1353.
Package xcolor Info: Model `hsb' substituted by `rgb' on input line 1357.
Package xcolor Info: Model `RGB' extended on input line 1369.
Package xcolor Info: Model `HTML' substituted by `rgb' on input line
1371.
Package xcolor Info: Model `Hsb' substituted by `hsb' on input line 1372.
Package xcolor Info: Model `tHsb' substituted by `hsb' on input line
1373.
Package xcolor Info: Model `HSB' substituted by `hsb' on input line 1374.
Package xcolor Info: Model `Gray' substituted by `gray' on input line
1375.
Package xcolor Info: Model `wave' substituted by `hsb' on input line
1376.
) (c:/TeXLive/2022/texmf-dist/tex/latex/colortbl/colortbl.sty
Package: colortbl 2022/06/20 v1.0f Color table columns (DPC)
(c:/TeXLive/2022/texmf-dist/tex/latex/tools/array.sty
```

```
Package: array 2022/09/04 v2.5g Tabular extension package (FMi)
\col@sep=\dimen142
\ar@mcellbox=\box52
\extrarowheight=\dimen143
\NC@list=\toks26
\extratabsurround=\skip64
\backup@length=\skip65
\ar@cellbox=\box53
)
\everycr=\toks27
\minrowclearance=\skip66
\rownum=\count197
) (c:/TeXLive/2022/texmf-dist/tex/latex/graphics/graphicx.sty
Package: graphicx 2021/09/16 v1.2d Enhanced LaTeX Graphics (DPC,SPQR)
(c:/TeXLive/2022/texmf-dist/tex/latex/graphics/graphics.sty
Package: graphics 2022/03/10 v1.4e Standard LaTeX Graphics (DPC,SPQR)
(c:/TeXLive/2022/texmf-dist/tex/latex/graphics/trig.sty
Package: trig 2021/08/11 v1.11 sin cos tan (DPC)
) (c:/TeXLive/2022/texmf-dist/tex/latex/graphics-cfg/graphics.cfg
File: graphics.cfg 2016/06/04 v1.11 sample graphics configuration
)
Package graphics Info: Driver file: pdftex.def on input line 107.
)
\Gin@req@height=\dimen144
\Gin@req@width=\dimen145
) (c:/TeXLive/2022/texmf-dist/tex/latex/xpatch/xpatch.sty
(c:/TeXLive/2022/texm
f-dist/tex/latex/l3kernel/expl3.sty
Package: expl3 2022-12-17 L3 programming layer (loader)

! LaTeX Error: Mismatched LaTeX support files detected.
(LaTeX)        Loading 'expl3.sty' aborted!
(LaTeX)
(LaTeX)        The L3 programming layer in the LaTeX format
(LaTeX)        is dated 2022-10-26, but in your TeX tree the files
require
(LaTeX)        at least 2022-12-17.

For immediate help type H <return>.
 ...

l.77      \ExplLoaderFileDate{expl3.sty}}
                                          %

The most likely causes are:
 - A recent format generation failed;
 - A stray format file in the user tree which needs to be removed or
rebuilt;
 - You are running a manually installed version of expl3.sty
   which is incompatible with the version in LaTeX.

LaTeX will abort loading the incompatible support files but this may lead
to
```

```
later errors. Please ensure that your LaTeX format is correctly
regenerated.


)
Package: xpatch 2020/03/25 v0.3a Extending etoolbox patching commands
(c:/TeXLive/2022/texmf-dist/tex/latex/l3packages/xparse/xparse.sty
Package: xparse 2022-12-17 L3 Experimental document command parser
)) (c:/TeXLive/2022/texmf-dist/tex/latex/environ/environ.sty
Package: environ 2014/05/04 v0.3 A new way to define environments
(c:/TeXLive/2022/texmf-dist/tex/latex/trimspaces/trimspaces.sty
Package: trimspaces 2009/09/17 v1.1 Trim spaces around a token list
)
\@envbody=\toks28
) (c:/TeXLive/2022/texmf-dist/tex/latex/lastpage/lastpage.sty
Package: lastpage 2021/09/03 v1.2n Refers to last page's name (HMM; JPG)
) (c:/TeXLive/2022/texmf-dist/tex/latex/graphics/rotating.sty
Package: rotating 2016/08/11 v2.16d rotated objects in LaTeX
(c:/TeXLive/2022/texmf-dist/tex/latex/base/ifthen.sty
Package: ifthen 2022/04/13 v1.1d Standard LaTeX ifthen package (DPC)
)
\c@r@tfl@t=\count198
\rotFPtop=\skip67
\rotFPbot=\skip68
\rot@float@box=\box54
\rot@mess@toks=\toks29
) (c:/TeXLive/2022/texmf-dist/tex/latex/graphics/lscape.sty
Package: lscape 2020/05/28 v3.02 Landscape Pages (DPC)
) (c:/TeXLive/2022/texmf-dist/tex/latex/tools/afterpage.sty
Package: afterpage 2014/10/28 v1.08 After-Page Package (DPC)
\AP@output=\toks30
\AP@partial=\box55
\AP@footins=\box56
) (c:/TeXLive/2022/texmf-dist/tex/latex/textpos/textpos.sty
Package: textpos 2022/07/23 v1.10.1
Package textpos Info: choosing support for LaTeX3 on input line 60.
\TP@textbox=\box57
\TP@holdbox=\box58
\TPHorizModule=\dimen146
\TPVertModule=\dimen147
\TP@margin=\dimen148
\TP@absmargin=\dimen149
Grid set 16 x 16 = 37.34424pt x 52.81541pt
\TPboxrulesize=\dimen150
\TP@ox=\dimen151
\TP@oy=\dimen152
\TP@tbargs=\toks31
TextBlockOrigin set to 0pt x 0pt
) (c:/TeXLive/2022/texmf-dist/tex/latex/url/url.sty
\Urlmuskip=\muskip19
Package: url 2013/09/16  ver 3.4  Verb mode for urls, etc.
) (c:/TeXLive/2022/texmf-dist/tex/latex/newfloat/newfloat.sty
Package: newfloat 2019/09/02 v1.1l Defining new floating environments
(AR)
```

```
Package newfloat Info: `rotating' package detected.
) (c:/TeXLive/2022/texmf-dist/tex/latex/mdframed/mdframed.sty
Package: mdframed 2013/07/01 1.9b: mdframed
(c:/TeXLive/2022/texmf-dist/tex/latex/kvoptions/kvoptions.sty
Package: kvoptions 2022-06-15 v3.15 Key value format for package options
(HO)
(c:/TeXLive/2022/texmf-dist/tex/generic/ltxcmds/ltxcmds.sty
Package: ltxcmds 2020-05-10 v1.25 LaTeX kernel commands for general use
(HO)
) (c:/TeXLive/2022/texmf-dist/tex/latex/kvsetkeys/kvsetkeys.sty
Package: kvsetkeys 2022-10-05 v1.19 Key value parser (HO)
)) (c:/TeXLive/2022/texmf-dist/tex/latex/zref/zref-abspage.sty
Package: zref-abspage 2022-04-07 v2.34 Module abspage for zref (HO)
(c:/TeXLive/2022/texmf-dist/tex/latex/zref/zref-base.sty
Package: zref-base 2022-04-07 v2.34 Module base for zref (HO)
(c:/TeXLive/2022/texmf-dist/tex/generic/infwarerr/infwarerr.sty
Package: infwarerr 2019/12/03 v1.5 Providing info/warning/error messages
(HO)
) (c:/TeXLive/2022/texmf-dist/tex/generic/kvdefinekeys/kvdefinekeys.sty
Package: kvdefinekeys 2019-12-19 v1.6 Define keys (HO)
) (c:/TeXLive/2022/texmf-dist/tex/generic/pdftexcmds/pdftexcmds.sty
Package: pdftexcmds 2020-06-27 v0.33 Utility functions of pdfTeX for
LuaTeX (HO
)
Package pdftexcmds Info: \pdf@primitive is available.
Package pdftexcmds Info: \pdf@ifprimitive is available.
Package pdftexcmds Info: \pdfdraftmode found.
) (c:/TeXLive/2022/texmf-dist/tex/generic/etexcmds/etexcmds.sty
Package: etexcmds 2019/12/15 v1.7 Avoid name clashes with e-TeX commands
(HO)
) (c:/TeXLive/2022/texmf-dist/tex/latex/auxhook/auxhook.sty
Package: auxhook 2019-12-17 v1.6 Hooks for auxiliary files (HO)
)
Package zref Info: New property list: main on input line 767.
Package zref Info: New property: default on input line 768.
Package zref Info: New property: page on input line 769.
) (c:/TeXLive/2022/texmf-dist/tex/latex/base/atbegshi-ltx.sty
Package: atbegshi-ltx 2021/01/10 v1.0c Emulation of the original atbegshi
package with kernel methods
)
\c@abspage=\count199
Package zref Info: New property: abspage on input line 65.
) (c:/TeXLive/2022/texmf-dist/tex/latex/needspace/needspace.sty
Package: needspace 2010/09/12 v1.3d reserve vertical space
)
\mdf@templength=\skip69
\c@mdf@globalstyle@cnt=\count266
\mdf@skipabove@length=\skip70
\mdf@skipbelow@length=\skip71
\mdf@leftmargin@length=\skip72
\mdf@rightmargin@length=\skip73
\mdf@innerleftmargin@length=\skip74
\mdf@innerrightmargin@length=\skip75
\mdf@innertopmargin@length=\skip76
```

```
\mdf@innerbottommargin@length=\skip77
\mdf@splittopskip@length=\skip78
\mdf@splitbottomskip@length=\skip79
\mdf@outermargin@length=\skip80
\mdf@innermargin@length=\skip81
\mdf@linewidth@length=\skip82
\mdf@innerlinewidth@length=\skip83
\mdf@middlelinewidth@length=\skip84
\mdf@outerlinewidth@length=\skip85
\mdf@roundcorner@length=\skip86
\mdf@footenotedistance@length=\skip87
\mdf@userdefinedwidth@length=\skip88
\mdf@needspace@length=\skip89
\mdf@frametitleaboveskip@length=\skip90
\mdf@frametitlebelowskip@length=\skip91
\mdf@frametitlerulewidth@length=\skip92
\mdf@frametitleleftmargin@length=\skip93
\mdf@frametitlerightmargin@length=\skip94
\mdf@shadowsize@length=\skip95
\mdf@extratopheight@length=\skip96
\mdf@subtitleabovelinewidth@length=\skip97
\mdf@subtitlebelowlinewidth@length=\skip98
\mdf@subtitleaboveskip@length=\skip99
\mdf@subtitlebelowskip@length=\skip100
\mdf@subtitleinneraboveskip@length=\skip101
\mdf@subtitleinnerbelowskip@length=\skip102
\mdf@subsubtitleabovelinewidth@length=\skip103
\mdf@subsubtitlebelowlinewidth@length=\skip104
\mdf@subsubtitleaboveskip@length=\skip105
\mdf@subsubtitlebelowskip@length=\skip106
\mdf@subsubtitleinneraboveskip@length=\skip107
\mdf@subsubtitleinnerbelowskip@length=\skip108
(c:/TeXLive/2022/texmf-dist/tex/latex/mdframed/md-frame-0.mdf
File: md-frame-0.mdf 2013/07/01\ 1.9b: md-frame-0
)
\mdf@frametitlebox=\box59
\mdf@footnotebox=\box60
\mdf@splitbox@one=\box61
\mdf@splitbox@two=\box62
\mdf@splitbox@save=\box63
\mdfsplitboxwidth=\skip109
\mdfsplitboxtotalwidth=\skip110
\mdfsplitboxheight=\skip111
\mdfsplitboxdepth=\skip112
\mdfsplitboxtotalheight=\skip113
\mdfframetitleboxwidth=\skip114
\mdfframetitleboxtotalwidth=\skip115
\mdfframetitleboxheight=\skip116
\mdfframetitleboxdepth=\skip117
\mdfframetitleboxtotalheight=\skip118
\mdffootnoteboxwidth=\skip119
\mdffootnoteboxtotalwidth=\skip120
\mdffootnoteboxheight=\skip121
\mdffootnoteboxdepth=\skip122
```

```
\mdffootnoteboxtotalheight=\skip123
\mdftotallinewidth=\skip124
\mdfboundingboxwidth=\skip125
\mdfboundingboxtotalwidth=\skip126
\mdfboundingboxheight=\skip127
\mdfboundingboxdepth=\skip128
\mdfboundingboxtotalheight=\skip129
\mdf@freevspace@length=\skip130
\mdf@horizontalwidthofbox@length=\skip131
\mdf@verticalmarginwhole@length=\skip132
\mdf@horizontalspaceofbox=\skip133
\mdfsubtitleheight=\skip134
\mdfsubsubtitleheight=\skip135
\c@mdfcountframes=\count267

****** mdframed patching \endmdf@trivlist

****** -- success******

\mdf@envdepth=\count268
\c@mdf@env@i=\count269
\c@mdf@env@ii=\count270
\c@mdf@zref@counter=\count271
Package zref Info: New property: mdf@pagevalue on input line 895.
) (c:/TeXLive/2022/texmf-dist/tex/latex/titlesec/titlesec.sty
Package: titlesec 2021/07/05 v2.14 Sectioning titles
\ttl@box=\box64
\beforetitleunit=\skip136
\aftertitleunit=\skip137
\ttl@plus=\dimen153
\ttl@minus=\dimen154
\ttl@toksa=\toks32
\titlewidth=\dimen155
\titlewidthlast=\dimen156
\titlewidthfirst=\dimen157
) (c:/TeXLive/2022/texmf-dist/tex/latex/koma-script/scrextend.sty
Package: scrextend 2022/10/12 v3.38 KOMA-Script package (extend other
classes w
ith features of KOMA-Script classes)
(c:/TeXLive/2022/texmf-dist/tex/latex/koma-script/scrkbase.sty
Package: scrkbase 2022/10/12 v3.38 KOMA-Script package (KOMA-Script-
dependent b
asics and keyval usage)
(c:/TeXLive/2022/texmf-dist/tex/latex/koma-script/scrbase.sty
Package: scrbase 2022/10/12 v3.38 KOMA-Script package (KOMA-Script-
independent
basics and keyval usage)
(c:/TeXLive/2022/texmf-dist/tex/latex/koma-script/scrlfile.sty
Package: scrlfile 2022/10/12 v3.38 KOMA-Script package (file load hooks)
(c:/TeXLive/2022/texmf-dist/tex/latex/koma-script/scrlfile-hook.sty
Package: scrlfile-hook 2022/10/12 v3.38 KOMA-Script package (using LaTeX
hooks)

(c:/TeXLive/2022/texmf-dist/tex/latex/koma-script/scrlogo.sty
```

```
Package: scrlogo 2022/10/12 v3.38 KOMA-Script package (logo)
)))
Applying: [2021/05/01] Usage of raw or classic option list on input line
252.
Already applied: [0000/00/00] Usage of raw or classic option list on
input line
 368.
))
Package scrextend Info: unexpected definition of `\@makefnmark'.
(scrextend)             Trying to patch it on input line 1709.
Package scrextend Info: patch seems to be successfull on input line 1709.
)

LaTeX Font Warning: Font shape `T1/cmr/m/n' in size <7.5> not available
(Font)              size <7> substituted on input line 65.

(c:/TeXLive/2022/texmf-dist/tex/latex/tools/calc.sty
Package: calc 2017/05/25 v4.3 Infix arithmetic (KKT,FJ)
\calc@Acount=\count272
\calc@Bcount=\count273
\calc@Adimen=\dimen158
\calc@Bdimen=\dimen159
\calc@Askip=\skip138
\calc@Bskip=\skip139
LaTeX Info: Redefining \setlength on input line 80.
LaTeX Info: Redefining \addtolength on input line 81.
\calc@Ccount=\count274
\calc@Cskip=\skip140
) (c:/TeXLive/2022/texmf-dist/tex/latex/geometry/geometry.sty
Package: geometry 2020/01/02 v5.9 Page Geometry
(c:/TeXLive/2022/texmf-dist/tex/generic/iftex/ifvtex.sty
Package: ifvtex 2019/10/25 v1.7 ifvtex legacy package. Use iftex instead.
)
\Gm@cnth=\count275
\Gm@cntv=\count276
\c@Gm@tempcnt=\count277
\Gm@bindingoffset=\dimen160
\Gm@wd@mp=\dimen161
\Gm@odd@mp=\dimen162
\Gm@even@mp=\dimen163
\Gm@layoutwidth=\dimen164
\Gm@layoutheight=\dimen165
\Gm@layouthoffset=\dimen166
\Gm@layoutvoffset=\dimen167
\Gm@dimlist=\toks33
) (c:/TeXLive/2022/texmf-dist/tex/latex/hyperref/hyperref.sty
Package: hyperref 2022-11-13 v7.00u Hypertext links for LaTeX
(c:/TeXLive/2022/texmf-dist/tex/generic/pdfescape/pdfescape.sty
Package: pdfescape 2019/12/09 v1.15 Implements pdfTeX's escape features
(HO)
) (c:/TeXLive/2022/texmf-dist/tex/latex/hycolor/hycolor.sty
Package: hycolor 2020-01-27 v1.10 Color options for hyperref/bookmark
(HO)
) (c:/TeXLive/2022/texmf-dist/tex/latex/letltxmacro/letltxmacro.sty
```

```
Package: letltxmacro 2019/12/03 v1.6 Let assignment for LaTeX macros (HO)
) (c:/TeXLive/2022/texmf-dist/tex/latex/hyperref/nameref.sty
Package: nameref 2022-05-17 v2.50 Cross-referencing by name of section
(c:/TeXLive/2022/texmf-dist/tex/latex/refcount/refcount.sty
Package: refcount 2019/12/15 v3.6 Data extraction from label references
(HO)
) (c:/TeXLive/2022/texmf-
dist/tex/generic/gettitlestring/gettitlestring.sty
Package: gettitlestring 2019/12/15 v1.6 Cleanup title references (HO)
)
\c@section@level=\count278
)
\@linkdim=\dimen168
\Hy@linkcounter=\count279
\Hy@pagecounter=\count280
(c:/TeXLive/2022/texmf-dist/tex/latex/hyperref/pd1enc.def
File: pd1enc.def 2022-11-13 v7.00u Hyperref: PDFDocEncoding definition
(HO)
Now handling font encoding PD1 ...
... no UTF-8 mapping file for font encoding PD1
) (c:/TeXLive/2022/texmf-dist/tex/generic/intcalc/intcalc.sty
Package: intcalc 2019/12/15 v1.3 Expandable calculations with integers
(HO)
)
\Hy@SavedSpaceFactor=\count281
(c:/TeXLive/2022/texmf-dist/tex/latex/hyperref/puenc.def
File: puenc.def 2022-11-13 v7.00u Hyperref: PDF Unicode definition (HO)
Now handling font encoding PU ...
... no UTF-8 mapping file for font encoding PU
)
Package hyperref Info: Option `colorlinks' set `true' on input line 4045.
Package hyperref Info: Hyper figures OFF on input line 4162.
Package hyperref Info: Link nesting OFF on input line 4167.
Package hyperref Info: Hyper index ON on input line 4170.
Package hyperref Info: Plain pages OFF on input line 4177.
Package hyperref Info: Backreferencing OFF on input line 4182.
Package hyperref Info: Implicit mode ON; LaTeX internals redefined.
Package hyperref Info: Bookmarks ON on input line 4410.
\c@Hy@tempcnt=\count282
LaTeX Info: Redefining \url on input line 4748.
\XeTeXLinkMargin=\dimen169
(c:/TeXLive/2022/texmf-dist/tex/generic/bitset/bitset.sty
Package: bitset 2019/12/09 v1.3 Handle bit-vector datatype (HO)
(c:/TeXLive/2022/texmf-dist/tex/generic/bigintcalc/bigintcalc.sty
Package: bigintcalc 2019/12/15 v1.5 Expandable calculations on big
integers (HO
)
))
\Fld@menulength=\count283
\Field@Width=\dimen170
\Fld@charsize=\dimen171
Package hyperref Info: Hyper figures OFF on input line 6027.
Package hyperref Info: Link nesting OFF on input line 6032.
Package hyperref Info: Hyper index ON on input line 6035.
```

```
Package hyperref Info: backreferencing OFF on input line 6042.
Package hyperref Info: Link coloring ON on input line 6045.
Package hyperref Info: Link coloring with OCG OFF on input line 6052.
Package hyperref Info: PDF/A mode OFF on input line 6057.
\Hy@abspage=\count284
\c@Item=\count285
\c@Hfootnote=\count286
)
Package hyperref Info: Driver (autodetected): hpdftex.
(c:/TeXLive/2022/texmf-dist/tex/latex/hyperref/hpdftex.def
File: hpdftex.def 2022-11-13 v7.00u Hyperref driver for pdfTeX
(c:/TeXLive/2022/texmf-dist/tex/latex/base/atveryend-ltx.sty
Package: atveryend-ltx 2020/08/19 v1.0a Emulation of the original
atveryend pac
kage
with kernel methods
)
\HyAnn@Count=\count287
\Fld@listcount=\count288
\c@bookmark@seq@number=\count289
(c:/TeXLive/2022/texmf-dist/tex/latex/rerunfilecheck/rerunfilecheck.sty
Package: rerunfilecheck 2022-07-10 v1.10 Rerun checks for auxiliary files
(HO)
(c:/TeXLive/2022/texmf-dist/tex/generic/uniquecounter/uniquecounter.sty
Package: uniquecounter 2019/12/15 v1.4 Provide unlimited unique counter
(HO)
)
Package uniquecounter Info: New unique counter `rerunfilecheck' on input
line 2
85.
)
\Hy@SectionHShift=\skip141
) (c:/TeXLive/2022/texmf-dist/tex/latex/preprint/authblk.sty
Package: authblk 2001/02/27 1.3 (PWD)
\affilsep=\skip142
\@affilsep=\skip143
\c@Maxaffil=\count290
\c@authors=\count291
\c@affil=\count292
) (c:/TeXLive/2022/texmf-dist/tex/latex/footmisc/footmisc.sty
Package: footmisc 2022/03/08 v6.0d a miscellany of footnote facilities
\FN@temptoken=\toks34
\footnotemargin=\dimen172
\@outputbox@depth=\dimen173
Package footmisc Info: Declaring symbol style bringhurst on input line
695.
Package footmisc Info: Declaring symbol style chicago on input line 703.
Package footmisc Info: Declaring symbol style wiley on input line 712.
Package footmisc Info: Declaring symbol style lamport-robust on input
line 723.

Package footmisc Info: Declaring symbol style lamport* on input line 743.
Package footmisc Info: Declaring symbol style lamport*-robust on input
line 764
```

```
.
) (c:/TeXLive/2022/texmf-dist/tex/latex/fancyhdr/fancyhdr.sty
Package: fancyhdr 2022/11/09 v4.1 Extensive control of page headers and
footers

\f@nch@headwidth=\skip144
\f@nch@O@elh=\skip145
\f@nch@O@erh=\skip146
\f@nch@O@olh=\skip147
\f@nch@O@orh=\skip148
\f@nch@O@elf=\skip149
\f@nch@O@erf=\skip150
\f@nch@O@olf=\skip151
\f@nch@O@orf=\skip152
) (c:/TeXLive/2022/texmf-dist/tex/generic/alphalph/alphalph.sty
Package: alphalph 2019/12/09 v2.6 Convert numbers to letters (HO)
)
\c@authorfn=\count293
(c:/TeXLive/2022/texmf-dist/tex/latex/abstract/abstract.sty
Package: abstract 2009/06/08 v1.2a configurable abstracts
\abstitleskip=\skip153
\absleftindent=\skip154
\absrightindent=\skip155
\absparindent=\skip156
\absparsep=\skip157
)
Package newfloat Info: New float `keypoints' with options
`placement=t!,name=kp
t' on input line 286.
\c@keypoints=\count294
\newfloat@ftype=\count295
Package newfloat Info: float type `keypoints'=8 on input line 286.
(c:/TeXLive/2022/texmf-dist/tex/latex/enumitem/enumitem.sty
Package: enumitem 2019/06/20 v3.9 Customized lists
\labelindent=\skip158
\enit@outerparindent=\dimen174
\enit@toks=\toks35
\enit@inbox=\box65
\enit@count@id=\count296
\enitdp@description=\count297
) (c:/TeXLive/2022/texmf-dist/tex/latex/quoting/quoting.sty
Package: quoting 2014/01/28 v0.1c Consolidated environment for displayed
text
\quo@toppartop=\skip159
) (c:/TeXLive/2022/texmf-dist/tex/latex/sttools/stfloats.sty
Package: stfloats 2017/03/27 v3.3 Improve float mechanism and
baselineskip sett
ings
\@dblbotnum=\count298
\c@dblbotnumber=\count299
) (c:/TeXLive/2022/texmf-dist/tex/latex/booktabs/booktabs.sty
Package: booktabs 2020/01/12 v1.61803398 Publication quality tables
\heavyrulewidth=\dimen175
\lightrulewidth=\dimen176
```

```
\cmidrulewidth=\dimen177
\belowrulesep=\dimen178
\belowbottomsep=\dimen179
\aboverulesep=\dimen180
\abovetopsep=\dimen181
\cmidrulesep=\dimen182
\cmidrulekern=\dimen183
\defaultaddspace=\dimen184
\@cmidla=\count300
\@cmidlb=\count301
\@aboverulesep=\dimen185
\@belowrulesep=\dimen186
\@thisruleclass=\count302
\@lastruleclass=\count303
\@thisrulewidth=\dimen187
) (c:/TeXLive/2022/texmf-dist/tex/latex/tools/tabularx.sty
Package: tabularx 2020/01/15 v2.11c `tabularx' package (DPC)
\TX@col@width=\dimen188
\TX@old@table=\dimen189
\TX@old@col=\dimen190
\TX@target=\dimen191
\TX@delta=\dimen192
\TX@cols=\count304
\TX@ftn=\toks36
)
\enitdp@tablenotes=\count305
(c:/TeXLive/2022/texmf-dist/tex/latex/caption/caption.sty
Package: caption 2022/03/01 v3.6b Customizing captions (AR)
(c:/TeXLive/2022/texmf-dist/tex/latex/caption/caption3.sty
Package: caption3 2022/03/17 v2.3b caption3 kernel (AR)
\caption@tempdima=\dimen193
\captionmargin=\dimen194
\caption@leftmargin=\dimen195
\caption@rightmargin=\dimen196
\caption@width=\dimen197
\caption@indent=\dimen198
\caption@parindent=\dimen199
\caption@hangindent=\dimen256
Package caption Info: Standard document class detected.
)
\c@caption@flags=\count306
\c@continuedfloat=\count307
Package caption Info: hyperref package is loaded.
Package caption Info: rotating package is loaded.
) (c:/TeXLive/2022/texmf-dist/tex/latex/natbib/natbib.sty
Package: natbib 2010/09/13 8.31b (PWD, AO)
\bibhang=\skip160
\bibsep=\skip161
LaTeX Info: Redefining \cite on input line 694.
\c@NAT@ctr=\count308
)) (c:/TeXLive/2022/texmf-dist/tex/latex/siunitx/siunitx.sty
Package: siunitx 2023-01-03 v3.2.0 A comprehensive (SI) units package
\l__siunitx_angle_tmp_dim=\dimen257
\l__siunitx_angle_marker_box=\box66
```

```
\l__siunitx_angle_unit_box=\box67
\l__siunitx_compound_count_int=\count309
(c:/TeXLive/2022/texmf-dist/tex/latex/translations/translations.sty
Package: translations 2022/02/05 v1.12 internationalization of LaTeX2e
packages
 (CN)
)
\l__siunitx_number_exponent_fixed_int=\count310
\l__siunitx_number_min_decimal_int=\count311
\l__siunitx_number_min_integer_int=\count312
\l__siunitx_number_round_precision_int=\count313
\l__siunitx_number_lower_threshold_int=\count314
\l__siunitx_number_upper_threshold_int=\count315
\l__siunitx_number_group_first_int=\count316
\l__siunitx_number_group_size_int=\count317
\l__siunitx_number_group_minimum_int=\count318
(c:/TeXLive/2022/texmf-dist/tex/latex/amsmath/amstext.sty
Package: amstext 2021/08/26 v2.01 AMS text
(c:/TeXLive/2022/texmf-dist/tex/latex/amsmath/amsgen.sty
File: amsgen.sty 1999/11/30 v2.0 generic functions
\@emptytoks=\toks37
\ex@=\dimen258
))
\l__siunitx_table_tmp_box=\box68
\l__siunitx_table_tmp_dim=\dimen259
\l__siunitx_table_column_width_dim=\dimen260
\l__siunitx_table_integer_box=\box69
\l__siunitx_table_decimal_box=\box70
\l__siunitx_table_uncert_box=\box71
\l__siunitx_table_before_box=\box72
\l__siunitx_table_after_box=\box73
\l__siunitx_table_before_dim=\dimen261
\l__siunitx_table_carry_dim=\dimen262
\l__siunitx_unit_tmp_int=\count319
\l__siunitx_unit_position_int=\count320
\l__siunitx_unit_total_int=\count321
! Undefined control sequence.
\__siunitx_unit_non_latin:n ...epoint_generate:nn

{#1}{\char_value_catcode:n...
l.6908 ... \__siunitx_unit_non_latin:n { "03BC } }

The control sequence at the end of the top line
of your error message was never \def'ed. If you have
misspelled it (e.g., `\hobx'), type `I' and the correct
spelling (e.g., `I\hbox'). Otherwise just continue,
and I'll forget about whatever was undefined.

! Bad character code (956).
\char_value_catcode:n ..._eval:n {#1}\exp_stop_f:

l.6908 ... \__siunitx_unit_non_latin:n { "03BC } }

A character number must be between 0 and 255.
```

```
I changed this one to zero.

! Undefined control sequence.
\__siunitx_unit_non_latin:n ...epoint_generate:nn

{#1}{\char_value_catcode:n...
l.6926 ...__siunitx_unit_non_latin:n { "00B0 } C }

The control sequence at the end of the top line
of your error message was never \def'ed. If you have
misspelled it (e.g., `\hobx'), type `I' and the correct
spelling (e.g., `I\hbox'). Otherwise just continue,
and I'll forget about whatever was undefined.

! Undefined control sequence.
\__siunitx_unit_non_latin:n ...epoint_generate:nn

{#1}{\char_value_catcode:n...
l.6937 ... \__siunitx_unit_non_latin:n { "2126 } }

The control sequence at the end of the top line
of your error message was never \def'ed. If you have
misspelled it (e.g., `\hobx'), type `I' and the correct
spelling (e.g., `I\hbox'). Otherwise just continue,
and I'll forget about whatever was undefined.

! Bad character code (8486).
\char_value_catcode:n ..._eval:n {#1}\exp_stop_f:

l.6937 ... \__siunitx_unit_non_latin:n { "2126 } }

A character number must be between 0 and 255.
I changed this one to zero.

! Undefined control sequence.
\__siunitx_unit_non_latin:n ...epoint_generate:nn

{#1}{\char_value_catcode:n...
l.6960 ... \__siunitx_unit_non_latin:n { "02B9 } }

The control sequence at the end of the top line
of your error message was never \def'ed. If you have
misspelled it (e.g., `\hobx'), type `I' and the correct
spelling (e.g., `I\hbox'). Otherwise just continue,
and I'll forget about whatever was undefined.

! Bad character code (697).
\char_value_catcode:n ..._eval:n {#1}\exp_stop_f:

l.6960 ... \__siunitx_unit_non_latin:n { "02B9 } }

A character number must be between 0 and 255.
I changed this one to zero.
```

```
! Undefined control sequence.
\__siunitx_unit_non_latin:n ...epoint_generate:nn

{#1}{\char_value_catcode:n...
l.6961 ... \__siunitx_unit_non_latin:n { "02BA } }

The control sequence at the end of the top line
of your error message was never \def'ed. If you have
misspelled it (e.g., `\hobx'), type `I' and the correct
spelling (e.g., `I\hbox'). Otherwise just continue,
and I'll forget about whatever was undefined.

! Bad character code (698).
\char_value_catcode:n ..._eval:n {#1}\exp_stop_f:

l.6961 ... \__siunitx_unit_non_latin:n { "02BA } }

A character number must be between 0 and 255.
I changed this one to zero.

! Undefined control sequence.
\__siunitx_unit_non_latin:n ...epoint_generate:nn

{#1}{\char_value_catcode:n...
l.6962 ... \__siunitx_unit_non_latin:n { "00B0 } }

The control sequence at the end of the top line
of your error message was never \def'ed. If you have
misspelled it (e.g., `\hobx'), type `I' and the correct
spelling (e.g., `I\hbox'). Otherwise just continue,
and I'll forget about whatever was undefined.

! Undefined control sequence.
\__siunitx_quantity_non_latin:n ...nt_generate:nn

{#1}{\char_value_catcode:n...
l.7220 ...siunitx_quantity_non_latin:n { "02B9 } }

The control sequence at the end of the top line
of your error message was never \def'ed. If you have
misspelled it (e.g., `\hobx'), type `I' and the correct
spelling (e.g., `I\hbox'). Otherwise just continue,
and I'll forget about whatever was undefined.

! Bad character code (697).
\char_value_catcode:n ..._eval:n {#1}\exp_stop_f:

l.7220 ...siunitx_quantity_non_latin:n { "02B9 } }

A character number must be between 0 and 255.
I changed this one to zero.

! Undefined control sequence.
\__siunitx_quantity_non_latin:n ...nt_generate:nn
```

```
{#1}{\char_value_catcode:n...
l.7223 ...siunitx_quantity_non_latin:n { "02BA } }

The control sequence at the end of the top line
of your error message was never \def'ed. If you have
misspelled it (e.g., `\hobx'), type `I' and the correct
spelling (e.g., `I\hbox'). Otherwise just continue,
and I'll forget about whatever was undefined.

! Bad character code (698).
\char_value_catcode:n ..._eval:n {#1}\exp_stop_f:

l.7223 ...siunitx_quantity_non_latin:n { "02BA } }

A character number must be between 0 and 255.
I changed this one to zero.

! Undefined control sequence.
\__siunitx_quantity_non_latin:n ...nt_generate:nn

{#1}{\char_value_catcode:n...
l.7226 ...siunitx_quantity_non_latin:n { "00B0 } }

The control sequence at the end of the top line
of your error message was never \def'ed. If you have
misspelled it (e.g., `\hobx'), type `I' and the correct
spelling (e.g., `I\hbox'). Otherwise just continue,
and I'll forget about whatever was undefined.

! Undefined control sequence.
\__siunitx_emulation_non_latin:n ...t_generate:nn

{#1}{\char_value_catcode:n...
l.8845   }

The control sequence at the end of the top line
of your error message was never \def'ed. If you have
misspelled it (e.g., `\hobx'), type `I' and the correct
spelling (e.g., `I\hbox'). Otherwise just continue,
and I'll forget about whatever was undefined.

! Undefined control sequence.
\__siunitx_emulation_non_latin:n ...t_generate:nn

{#1}{\char_value_catcode:n...
l.8845   }

The control sequence at the end of the top line
of your error message was never \def'ed. If you have
misspelled it (e.g., `\hobx'), type `I' and the correct
spelling (e.g., `I\hbox'). Otherwise just continue,
and I'll forget about whatever was undefined.
```

```
) (c:/TeXLive/2022/texmf-dist/tex/latex/sttools/flushend.sty
Package: flushend 2021/10/04 v4.0 Balancing columns in twocolumn mode
\flushend@@lastskip@a=\skip162
\flushend@@lastskip@b=\skip163
\flushend@@lastnode=\count322
\var@@loop@iter=\count323
\var@@temp@spread=\dimen263
\var@@temp@a=\dimen264
\var@@temp@loop=\dimen265
\flushend@@page@rule=\dimen266
\flushend@@varbox@lastpage=\box74
\flushend@@varbox@a=\box75
\flushend@@varbox@c=\box76
\flushend@@tempbox@a=\box77
\flushend@@tempbox@c=\box78
\flushend@@floatbox=\box79
\@viper=\box80
\hold@viper=\box81
\atColsBreak=\toks38
\atColsEnd=\toks39
\oldbreak@skip=\dimen267
)
Package translations Info: No language package found. I am going to use
`englis
h' as default language. on input line 37.
LaTeX Font Info:    Trying to load font information for T1+Merriwthr-OsF
on inp
ut line 37.
(c:/TeXLive/2022/texmf-dist/tex/latex/merriweather/T1Merriwthr-OsF.fd
File: T1Merriwthr-OsF.fd 2020/08/30 (autoinst) Font definitions for
T1/Merriwth
r-OsF.
)
LaTeX Font Info:    Font shape `T1/Merriwthr-OsF/m/n' will be
(Font)              scaled to size 7.5pt on input line 37.
(c:/TeXLive/2022/texmf-dist/tex/latex/l3backend/l3backend-pdftex.def
File: l3backend-pdftex.def 2022-10-26 L3 backend support: PDF output
(pdfTeX)
\l__color_backend_stack_int=\count324
\l__pdf_internal_box=\box82
) (./main.aux)
\openout1 = `main.aux'.

LaTeX Font Info:    Checking defaults for OML/cmm/m/it on input line 37.
LaTeX Font Info:    ... okay on input line 37.
LaTeX Font Info:    Checking defaults for OMS/cmsy/m/n on input line 37.
LaTeX Font Info:    ... okay on input line 37.
LaTeX Font Info:    Checking defaults for OT1/cmr/m/n on input line 37.
LaTeX Font Info:    ... okay on input line 37.
LaTeX Font Info:    Checking defaults for T1/cmr/m/n on input line 37.
LaTeX Font Info:    ... okay on input line 37.
LaTeX Font Info:    Checking defaults for TS1/cmr/m/n on input line 37.
LaTeX Font Info:    ... okay on input line 37.
LaTeX Font Info:    Checking defaults for OMX/cmex/m/n on input line 37.
```

```
LaTeX Font Info:    ... okay on input line 37.
LaTeX Font Info:    Checking defaults for U/cmr/m/n on input line 37.
LaTeX Font Info:    ... okay on input line 37.
LaTeX Font Info:    Checking defaults for PD1/pdf/m/n on input line 37.
LaTeX Font Info:    ... okay on input line 37.
LaTeX Font Info:    Checking defaults for PU/pdf/m/n on input line 37.
LaTeX Font Info:    ... okay on input line 37.
LaTeX Info: Redefining \microtypecontext on input line 37.
Package microtype Info: Applying patch `item' on input line 37.
Package microtype Info: Applying patch `toc' on input line 37.
Package microtype Info: Applying patch `eqnum' on input line 37.

Package microtype Warning: Unable to apply patch `footnote' on input line
37.

Package microtype Info: Generating PDF output.
Package microtype Info: Character protrusion enabled (level 2).
Package microtype Info: Using default protrusion set `alltext'.
Package microtype Info: Automatic font expansion enabled (level 2),
(microtype)             stretch: 20, shrink: 20, step: 1, non-selected.
Package microtype Info: Using default expansion set `alltext-nott'.
LaTeX Info: Redefining \showhyphens on input line 37.
Package microtype Info: No adjustment of tracking.
Package microtype Info: No adjustment of interword spacing.
Package microtype Info: No adjustment of character kerning.
Package microtype Info: Loading generic protrusion settings for font
family
(microtype)             `Merriwthr-OsF' (encoding: T1).
(microtype)             For optimal results, create family-specific
settings.
(microtype)             See the microtype manual for details.
LaTeX Font Info:    Redeclaring symbol font `operators' on input line 37.
LaTeX Font Info:    Encoding `OT1' has changed to `T1' for symbol font
(Font)              `operators' in the math version `normal' on input
line 37.
LaTeX Font Info:    Overwriting symbol font `operators' in version
`normal'
(Font)                  OT1/cmr/m/n --> T1/Merriwthr-OsF/m/up on input
line 37.

LaTeX Font Info:    Encoding `OT1' has changed to `T1' for symbol font
(Font)              `operators' in the math version `bold' on input line
37.
LaTeX Font Info:    Overwriting symbol font `operators' in version `bold'
(Font)                  OT1/cmr/bx/n --> T1/Merriwthr-OsF/m/up on input
line 37
.
LaTeX Font Info:    Overwriting symbol font `operators' in version `bold'
(Font)                  T1/Merriwthr-OsF/m/up --> T1/Merriwthr-OsF/b/up
on inpu
t line 37.
LaTeX Font Info:    Redeclaring math alphabet \mathbf on input line 37.
LaTeX Font Info:    Overwriting math alphabet `\mathbf' in version
`normal'
```

```
(Font)                  OT1/cmr/bx/n --> T1/Merriwthr-OsF/b/up on input
line 37
.
LaTeX Font Info:    Overwriting math alphabet `\mathbf' in version `bold'
(Font)                  OT1/cmr/bx/n --> T1/Merriwthr-OsF/b/up on input
line 37
.
LaTeX Font Info:    Redeclaring math alphabet \mathsf on input line 37.
LaTeX Font Info:    Overwriting math alphabet `\mathsf' in version
`normal'
(Font)                  OT1/cmss/m/n --> T1/MerriwthrSans-OsF/m/up on
input lin
e 37.
LaTeX Font Info:    Overwriting math alphabet `\mathsf' in version `bold'
(Font)                  OT1/cmss/bx/n --> T1/MerriwthrSans-OsF/m/up on
input li
ne 37.
LaTeX Font Info:    Redeclaring math alphabet \mathit on input line 37.
LaTeX Font Info:    Overwriting math alphabet `\mathit' in version
`normal'
(Font)                  OT1/cmr/m/it --> T1/Merriwthr-OsF/m/it on input
line 37
.
LaTeX Font Info:    Overwriting math alphabet `\mathit' in version `bold'
(Font)                  OT1/cmr/bx/it --> T1/Merriwthr-OsF/m/it on input
line 3
7.
LaTeX Font Info:    Redeclaring math alphabet \mathtt on input line 37.
LaTeX Font Info:    Overwriting math alphabet `\mathtt' in version
`normal'
(Font)                  OT1/cmtt/m/n --> T1/lmtt/m/up on input line 37.
LaTeX Font Info:    Overwriting math alphabet `\mathtt' in version `bold'
(Font)                  OT1/cmtt/m/n --> T1/lmtt/m/up on input line 37.
LaTeX Font Info:    Overwriting math alphabet `\mathsf' in version `bold'
(Font)                  T1/MerriwthrSans-OsF/m/up --> T1/MerriwthrSans-
OsF/b/up
 on input line 37.
LaTeX Font Info:    Overwriting math alphabet `\mathit' in version `bold'
(Font)                  T1/Merriwthr-OsF/m/it --> T1/Merriwthr-OsF/b/it
on inpu
t line 37.
\c@mv@tabular=\count325
\c@mv@boldtabular=\count326
(c:/TeXLive/2022/texmf-dist/tex/context/base/mkii/supp-pdf.mkii
[Loading MPS to PDF converter (version 2006.09.02).]
\scratchcounter=\count327
\scratchdimen=\dimen268
\scratchbox=\box83
\nofMPsegments=\count328
\nofMParguments=\count329
\everyMPshowfont=\toks40
\MPscratchCnt=\count330
\MPscratchDim=\dimen269
\MPnumerator=\count331
```

```
\makeMPintoPDFobject=\count332
\everyMPtoPDFconversion=\toks41
) (c:/TeXLive/2022/texmf-dist/tex/latex/epstopdf-pkg/epstopdf-base.sty
Package: epstopdf-base 2020-01-24 v2.11 Base part for package epstopdf
Package epstopdf-base Info: Redefining graphics rule for `.eps' on input
line 4
85.
(c:/TeXLive/2022/texmf-dist/tex/latex/latexconfig/epstopdf-sys.cfg
File: epstopdf-sys.cfg 2010/07/13 v1.3 Configuration of (r)epstopdf for
TeX Liv
e
))
Package lastpage Info: Please have a look at the pageslts package at
(lastpage)             https://www.ctan.org/pkg/pageslts
(lastpage)             ! on input line 37.
*geometry* driver: auto-detecting
*geometry* detected driver: pdftex
*geometry* verbose mode - [ preamble ] result:
* driver: pdftex
* paper: a4paper
* layout: <same size as paper>
* layoutoffset:(h,v)=(0.0pt,0.0pt)
* modes: includefoot twoside
* h-part:(L,W,R)=(54.64pt, 488.22787pt, 54.64pt)
* v-part:(T,H,B)=(66.0pt, 745.04684pt, 34.0pt)
* \paperwidth=597.50787pt
* \paperheight=845.04684pt
* \textwidth=488.22787pt
* \textheight=715.04684pt
* \oddsidemargin=-17.62999pt
* \evensidemargin=-17.62999pt
* \topmargin=-47.76999pt
* \headheight=17.5pt
* \headsep=24.0pt
* \topskip=10.0pt
* \footskip=30.0pt
* \marginparwidth=48.0pt
* \marginparsep=10.0pt
* \columnsep=18.0pt
* \skip\footins=22.0pt plus 2.0pt
* \hoffset=0.0pt
* \voffset=0.0pt
* \mag=1000
* \@twocolumntrue
* \@twosidetrue
* \@mparswitchtrue
* \@reversemarginfalse
* (1in=72.27pt=25.4mm, 1cm=28.453pt)

Package hyperref Info: Link coloring ON on input line 37.
(./main.out) (./main.out)
\@outlinefile=\write3
\openout3 = `main.out'.
```

```
\@gscitedetails=\box84
\@gscitedetailsheight=\skip164
\@gsheadbox=\box85
\@gsheadboxheight=\skip165
LaTeX Font Info:    Font shape `T1/Merriwthr-OsF/b/n' will be
(Font)              scaled to size 6.5pt on input line 37.
LaTeX Font Info:    Calculating math sizes for size <7.5> on input line
37.

LaTeX Font Warning: Font shape `T1/Merriwthr-OsF/m/up' undefined
(Font)              using `T1/Merriwthr-OsF/m/n' instead on input line
37.

LaTeX Font Info:    Font shape `T1/Merriwthr-OsF/m/up' will be
(Font)              scaled to size 6.24973pt on input line 37.
LaTeX Font Info:    Font shape `T1/Merriwthr-OsF/m/up' will be
(Font)              scaled to size 5.24997pt on input line 37.
LaTeX Font Info:    Trying to load font information for U+eur on input
line 37.

(c:/TeXLive/2022/texmf-dist/tex/latex/amsfonts/ueur.fd
File: ueur.fd 2013/01/14 v3.01 Euler Roman
) (c:/TeXLive/2022/texmf-dist/tex/latex/microtype/mt-eur.cfg
File: mt-eur.cfg 2006/07/31 v1.1 microtype config. file: AMS Euler Roman
(RS)
)

LaTeX Font Warning: Font shape `OMS/cmsy/m/n' in size <7.5> not available
(Font)              size <7> substituted on input line 37.

LaTeX Font Info:    External font `cmex10' loaded for size
(Font)              <7.5> on input line 37.
LaTeX Font Info:    External font `cmex10' loaded for size
(Font)              <6.24973> on input line 37.
LaTeX Font Info:    External font `cmex10' loaded for size
(Font)              <5.24997> on input line 37.
LaTeX Font Info:    Trying to load font information for U+euf on input
line 37.

(c:/TeXLive/2022/texmf-dist/tex/latex/amsfonts/ueuf.fd
File: ueuf.fd 2013/01/14 v3.01 Euler Fraktur
) (c:/TeXLive/2022/texmf-dist/tex/latex/microtype/mt-euf.cfg
File: mt-euf.cfg 2006/07/03 v1.1 microtype config. file: AMS Euler
Fraktur (RS)

)
LaTeX Font Info:    Trying to load font information for U+eus on input
line 37.

(c:/TeXLive/2022/texmf-dist/tex/latex/amsfonts/ueus.fd
File: ueus.fd 2013/01/14 v3.01 Euler Script
) (c:/TeXLive/2022/texmf-dist/tex/latex/microtype/mt-eus.cfg
File: mt-eus.cfg 2006/07/28 v1.2 microtype config. file: AMS Euler Script
(RS)
```

```
)
LaTeX Font Info:    Trying to load font information for U+euex on input
line 37
.
(c:/TeXLive/2022/texmf-dist/tex/latex/amsfonts/ueuex.fd
File: ueuex.fd 2013/01/14 v3.01 Euler extra symbols
)

LaTeX Font Warning: Font shape `OML/cmm/m/it' in size <7.5> not available
(Font)              size <7> substituted on input line 37.

LaTeX Font Info:    Font shape `T1/Merriwthr-OsF/m/n' will be
(Font)              scaled to size 6.24973pt on input line 37.
LaTeX Font Info:    Font shape `T1/Merriwthr-OsF/m/n' will be
(Font)              scaled to size 5.24997pt on input line 37.
LaTeX Font Info:    Font shape `T1/Merriwthr-OsF/m/it' will be
(Font)              scaled to size 7.5pt on input line 37.
LaTeX Font Info:    Font shape `T1/Merriwthr-OsF/m/it' will be
(Font)              scaled to size 6.24973pt on input line 37.
LaTeX Font Info:    Font shape `T1/Merriwthr-OsF/m/it' will be
(Font)              scaled to size 5.24997pt on input line 37.
LaTeX Font Info:    Font shape `T1/Merriwthr-OsF/m/n' will be
(Font)              scaled to size 8.0pt on input line 37.
LaTeX Font Info:    Font shape `T1/Merriwthr-OsF/m/it' will be
(Font)              scaled to size 8.0pt on input line 37.
LaTeX Font Info:    Font shape `T1/Merriwthr-OsF/b/it' will be
(Font)              scaled to size 8.0pt on input line 37.
Package caption Info: Begin \AtBeginDocument code.
Package caption Info: End \AtBeginDocument code.
! Undefined control sequence.
\__siunitx_symbol_non_latin:n ...oint_generate:nn

{#1}{\char_value_catcode:n...
l.37 \begin{document}

The control sequence at the end of the top line
of your error message was never \def'ed. If you have
misspelled it (e.g., `\hobx'), type `I' and the correct
spelling (e.g., `I\hbox'). Otherwise just continue,
and I'll forget about whatever was undefined.

! Bad character code (697).
\char_value_catcode:n ..._eval:n {#1}\exp_stop_f:

l.37 \begin{document}

A character number must be between 0 and 255.
I changed this one to zero.

! Undefined control sequence.
\__siunitx_symbol_non_latin:n ...oint_generate:nn

{#1}{\char_value_catcode:n...
l.37 \begin{document}
```

```
The control sequence at the end of the top line
of your error message was never \def'ed. If you have
misspelled it (e.g., `\hobx'), type `I' and the correct
spelling (e.g., `I\hbox'). Otherwise just continue,
and I'll forget about whatever was undefined.

! Bad character code (698).
\char_value_catcode:n ..._eval:n {#1}\exp_stop_f:

l.37 \begin{document}

A character number must be between 0 and 255.
I changed this one to zero.

! Undefined control sequence.
\__siunitx_symbol_non_latin:n ...oint_generate:nn

{#1}{\char_value_catcode:n...
l.37 \begin{document}

The control sequence at the end of the top line
of your error message was never \def'ed. If you have
misspelled it (e.g., `\hobx'), type `I' and the correct
spelling (e.g., `I\hbox'). Otherwise just continue,
and I'll forget about whatever was undefined.

! Undefined control sequence.
\__siunitx_symbol_non_latin:n ...oint_generate:nn

{#1}{\char_value_catcode:n...
l.37 \begin{document}

The control sequence at the end of the top line
of your error message was never \def'ed. If you have
misspelled it (e.g., `\hobx'), type `I' and the correct
spelling (e.g., `I\hbox'). Otherwise just continue,
and I'll forget about whatever was undefined.

! Undefined control sequence.
\__siunitx_symbol_non_latin:n ...oint_generate:nn

{#1}{\char_value_catcode:n...
l.37 \begin{document}

The control sequence at the end of the top line
of your error message was never \def'ed. If you have
misspelled it (e.g., `\hobx'), type `I' and the correct
spelling (e.g., `I\hbox'). Otherwise just continue,
and I'll forget about whatever was undefined.

! Bad character code (8486).
\char_value_catcode:n ..._eval:n {#1}\exp_stop_f:
```

```
l.37 \begin{document}

A character number must be between 0 and 255.
I changed this one to zero.

! Undefined control sequence.
\__siunitx_symbol_non_latin:n ...oint_generate:nn

{#1}{\char_value_catcode:n...
l.37 \begin{document}

The control sequence at the end of the top line
of your error message was never \def'ed. If you have
misspelled it (e.g., `\hobx'), type `I' and the correct
spelling (e.g., `I\hbox'). Otherwise just continue,
and I'll forget about whatever was undefined.

! Bad character code (956).
\char_value_catcode:n ..._eval:n {#1}\exp_stop_f:

l.37 \begin{document}

A character number must be between 0 and 255.
I changed this one to zero.

! Undefined control sequence.
\__siunitx_symbol_non_latin:n ...oint_generate:nn

{#1}{\char_value_catcode:n...
l.37 \begin{document}

The control sequence at the end of the top line
of your error message was never \def'ed. If you have
misspelled it (e.g., `\hobx'), type `I' and the correct
spelling (e.g., `I\hbox'). Otherwise just continue,
and I'll forget about whatever was undefined.

! Bad character code (937).
\char_value_catcode:n ..._eval:n {#1}\exp_stop_f:

l.37 \begin{document}

A character number must be between 0 and 255.
I changed this one to zero.

! Undefined control sequence.
\__siunitx_emulation_non_latin:n ...t_generate:nn

{#1}{\char_value_catcode:n...
l.37 \begin{document}

The control sequence at the end of the top line
of your error message was never \def'ed. If you have
misspelled it (e.g., `\hobx'), type `I' and the correct
```

```
spelling (e.g., `I\hbox'). Otherwise just continue,
and I'll forget about whatever was undefined.

! Undefined control sequence.
\__siunitx_emulation_non_latin:n ...t_generate:nn

{#1}{\char_value_catcode:n...
l.37 \begin{document}

The control sequence at the end of the top line
of your error message was never \def'ed. If you have
misspelled it (e.g., `\hobx'), type `I' and the correct
spelling (e.g., `I\hbox'). Otherwise just continue,
and I'll forget about whatever was undefined.

! Undefined control sequence.
\__siunitx_emulation_non_latin:n ...t_generate:nn

{#1}{\char_value_catcode:n...
l.37 \begin{document}

The control sequence at the end of the top line
of your error message was never \def'ed. If you have
misspelled it (e.g., `\hobx'), type `I' and the correct
spelling (e.g., `I\hbox'). Otherwise just continue,
and I'll forget about whatever was undefined.


(c:/TeXLive/2022/texmf-dist/tex/latex/translations/translations-basic-
dictionar
y-english.trsl
File: translations-basic-dictionary-english.trsl (english translation
file `tra
nslations-basic-dictionary')
)
Package translations Info: loading dictionary `translations-basic-
dictionary' f
or `english'. on input line 37.
TextBlockOrigin set to 4pc+6.64pt x 4pc+6pt
<oup.pdf, id=137, 49.18375pt x 48.18pt>
File: oup.pdf Graphic file (type pdf)
<use oup.pdf>
Package pdftex.def Info: oup.pdf  used on input line 48.
(pdftex.def)             Requested size: 59.24683pt x 58.038pt.
<gigascience-logo.pdf, id=138, 99.37125pt x 33.12375pt>
File: gigascience-logo.pdf Graphic file (type pdf)
<use gigascience-logo.pdf>
Package pdftex.def Info: gigascience-logo.pdf  used on input line 48.
(pdftex.def)             Requested size: 126.00902pt x 42.0pt.

Overfull \hbox (54.64pt too wide) in paragraph at lines 48--48
[][]
 []
```

```
LaTeX Font Info:    Font shape `T1/Merriwthr-OsF/m/n' will be
(Font)              scaled to size 14.0pt on input line 48.
LaTeX Font Info:    Font shape `T1/Merriwthr-OsF/m/n' will be
(Font)              scaled to size 8.99997pt on input line 48.
LaTeX Font Info:    Calculating math sizes for size <14> on input line
48.
LaTeX Font Info:    Font shape `T1/Merriwthr-OsF/m/up' will be
(Font)              scaled to size 14.0pt on input line 48.
LaTeX Font Info:    Font shape `T1/Merriwthr-OsF/m/up' will be
(Font)              scaled to size 11.66617pt on input line 48.
LaTeX Font Info:    Font shape `T1/Merriwthr-OsF/m/up' will be
(Font)              scaled to size 9.79996pt on input line 48.
LaTeX Font Info:    External font `cmex10' loaded for size
(Font)              <14> on input line 48.
LaTeX Font Info:    External font `cmex10' loaded for size
(Font)              <11.66617> on input line 48.
LaTeX Font Info:    External font `cmex10' loaded for size
(Font)              <9.79996> on input line 48.
LaTeX Font Info:    Font shape `T1/Merriwthr-OsF/m/n' will be
(Font)              scaled to size 11.66617pt on input line 48.
LaTeX Font Info:    Font shape `T1/Merriwthr-OsF/m/n' will be
(Font)              scaled to size 9.79996pt on input line 48.
LaTeX Font Info:    Font shape `T1/Merriwthr-OsF/m/it' will be
(Font)              scaled to size 14.0pt on input line 48.
LaTeX Font Info:    Font shape `T1/Merriwthr-OsF/m/it' will be
(Font)              scaled to size 11.66617pt on input line 48.
LaTeX Font Info:    Font shape `T1/Merriwthr-OsF/m/it' will be
(Font)              scaled to size 9.79996pt on input line 48.
LaTeX Font Info:    Font shape `T1/Merriwthr-OsF/b/n' will be
(Font)              scaled to size 18.0pt on input line 48.
LaTeX Font Info:    Font shape `T1/Merriwthr-OsF/m/n' will be
(Font)              scaled to size 13.0pt on input line 48.
LaTeX Font Info:    Calculating math sizes for size <13> on input line
48.
LaTeX Font Info:    Font shape `T1/Merriwthr-OsF/m/up' will be
(Font)              scaled to size 13.0pt on input line 48.
LaTeX Font Info:    Font shape `T1/Merriwthr-OsF/m/up' will be
(Font)              scaled to size 10.83287pt on input line 48.
LaTeX Font Info:    Font shape `T1/Merriwthr-OsF/m/up' will be
(Font)              scaled to size 9.09996pt on input line 48.

LaTeX Font Warning: Font shape `OMS/cmsy/m/n' in size <13> not available
(Font)              size <12> substituted on input line 48.

LaTeX Font Info:    External font `cmex10' loaded for size
(Font)              <13> on input line 48.
LaTeX Font Info:    External font `cmex10' loaded for size
(Font)              <10.83287> on input line 48.
LaTeX Font Info:    External font `cmex10' loaded for size
(Font)              <9.09996> on input line 48.

LaTeX Font Warning: Font shape `OML/cmm/m/it' in size <13> not available
(Font)              size <12> substituted on input line 48.
```

```
LaTeX Font Info:    Font shape `T1/Merriwthr-OsF/m/n' will be
(Font)              scaled to size 10.83287pt on input line 48.
LaTeX Font Info:    Font shape `T1/Merriwthr-OsF/m/n' will be
(Font)              scaled to size 9.09996pt on input line 48.
LaTeX Font Info:    Font shape `T1/Merriwthr-OsF/m/it' will be
(Font)              scaled to size 13.0pt on input line 48.
LaTeX Font Info:    Font shape `T1/Merriwthr-OsF/m/it' will be
(Font)              scaled to size 10.83287pt on input line 48.
LaTeX Font Info:    Font shape `T1/Merriwthr-OsF/m/it' will be
(Font)              scaled to size 9.09996pt on input line 48.
LaTeX Font Info:    Trying to load font information for TS1+Merriwthr-OsF
on in
put line 48.
(c:/TeXLive/2022/texmf-dist/tex/latex/merriweather/TS1Merriwthr-OsF.fd
File: TS1Merriwthr-OsF.fd 2020/08/30 (autoinst) Font definitions for
TS1/Merriw
thr-OsF.
)
LaTeX Font Info:    Font shape `TS1/Merriwthr-OsF/m/n' will be
(Font)              scaled to size 10.83287pt on input line 48.
Package microtype Info: Loading generic protrusion settings for font
family
(microtype)             `Merriwthr-OsF' (encoding: TS1).
(microtype)             For optimal results, create family-specific
settings.
(microtype)             See the microtype manual for details.
LaTeX Font Info:    Font shape `T1/Merriwthr-OsF/m/n' will be
(Font)              scaled to size 9.0pt on input line 48.
LaTeX Font Info:    Font shape `T1/Merriwthr-OsF/m/up' will be
(Font)              scaled to size 9.0pt on input line 48.
LaTeX Font Info:    Font shape `T1/Merriwthr-OsF/m/up' will be
(Font)              scaled to size 7.0pt on input line 48.
LaTeX Font Info:    Font shape `T1/Merriwthr-OsF/m/up' will be
(Font)              scaled to size 5.0pt on input line 48.
LaTeX Font Info:    External font `cmex10' loaded for size
(Font)              <9> on input line 48.
LaTeX Font Info:    External font `cmex10' loaded for size
(Font)              <7> on input line 48.
LaTeX Font Info:    External font `cmex10' loaded for size
(Font)              <5> on input line 48.
LaTeX Font Info:    Font shape `T1/Merriwthr-OsF/m/n' will be
(Font)              scaled to size 7.0pt on input line 48.
LaTeX Font Info:    Font shape `T1/Merriwthr-OsF/m/n' will be
(Font)              scaled to size 5.0pt on input line 48.
LaTeX Font Info:    Font shape `T1/Merriwthr-OsF/m/it' will be
(Font)              scaled to size 9.0pt on input line 48.
LaTeX Font Info:    Font shape `T1/Merriwthr-OsF/m/it' will be
(Font)              scaled to size 7.0pt on input line 48.
LaTeX Font Info:    Font shape `T1/Merriwthr-OsF/m/it' will be
(Font)              scaled to size 5.0pt on input line 48.
LaTeX Font Info:    Font shape `T1/Merriwthr-OsF/m/n' will be
(Font)              scaled to size 6.5pt on input line 48.
LaTeX Font Info:    Calculating math sizes for size <6.5> on input line
48.
```

```
LaTeX Font Info:    Font shape `T1/Merriwthr-OsF/m/up' will be
(Font)              scaled to size 6.5pt on input line 48.
LaTeX Font Info:    Font shape `T1/Merriwthr-OsF/m/up' will be
(Font)              scaled to size 5.41643pt on input line 48.
LaTeX Font Info:    Font shape `T1/Merriwthr-OsF/m/up' will be
(Font)              scaled to size 4.54997pt on input line 48.

LaTeX Font Warning: Font shape `OMS/cmsy/m/n' in size <6.5> not available
(Font)              size <6> substituted on input line 48.


LaTeX Font Warning: Font shape `OMS/cmsy/m/n' in size <5.41643> not
available
(Font)              size <5> substituted on input line 48.


LaTeX Font Warning: Font shape `OMS/cmsy/m/n' in size <4.54997> not
available
(Font)              size <5> substituted on input line 48.

LaTeX Font Info:    External font `cmex10' loaded for size
(Font)              <6.5> on input line 48.
LaTeX Font Info:    External font `cmex10' loaded for size
(Font)              <5.41643> on input line 48.
LaTeX Font Info:    External font `cmex10' loaded for size
(Font)              <4.54997> on input line 48.

LaTeX Font Warning: Font shape `OML/cmm/m/it' in size <6.5> not available
(Font)              size <6> substituted on input line 48.


LaTeX Font Warning: Font shape `OML/cmm/m/it' in size <5.41643> not
available
(Font)              size <5> substituted on input line 48.


LaTeX Font Warning: Font shape `OML/cmm/m/it' in size <4.54997> not
available
(Font)              size <5> substituted on input line 48.

LaTeX Font Info:    Font shape `T1/Merriwthr-OsF/m/n' will be
(Font)              scaled to size 5.41643pt on input line 48.
LaTeX Font Info:    Font shape `T1/Merriwthr-OsF/m/n' will be
(Font)              scaled to size 4.54997pt on input line 48.
LaTeX Font Info:    Font shape `T1/Merriwthr-OsF/m/it' will be
(Font)              scaled to size 6.5pt on input line 48.
LaTeX Font Info:    Font shape `T1/Merriwthr-OsF/m/it' will be
(Font)              scaled to size 5.41643pt on input line 48.
LaTeX Font Info:    Font shape `T1/Merriwthr-OsF/m/it' will be
(Font)              scaled to size 4.54997pt on input line 48.
LaTeX Font Info:    Font shape `TS1/Merriwthr-OsF/m/n' will be
(Font)              scaled to size 5.41643pt on input line 48.

Overfull \hbox (54.64pt too wide) in paragraph at lines 48--48
```

```
[][][]
 []

LaTeX Font Info:    Font shape `T1/Merriwthr-OsF/b/n' will be
(Font)              scaled to size 10.0pt on input line 48.
LaTeX Font Info:    Font shape `T1/Merriwthr-OsF/b/n' will be
(Font)              scaled to size 8.0pt on input line 48.

Overfull \hbox (54.64pt too wide) in paragraph at lines 48--48
[][][]
 []

LaTeX Font Info:    Font shape `T1/Merriwthr-OsF/b/n' will be
(Font)              scaled to size 7.5pt on input line 61.

Package natbib Warning: Citation `palumbo2015' on page 1 undefined on
input lin
e 61.


Package natbib Warning: Citation `rajkumar2020' on page 1 undefined on
input li
ne 61.


Package natbib Warning: Citation `ipssr' on page 1 undefined on input
line 61.


Package natbib Warning: Citation `malcovati2020' on page 1 undefined on
input l
ine 61.


Package natbib Warning: Citation `nowell1976' on page 1 undefined on
input line
 63.


Package natbib Warning: Citation `dasilva2017' on page 1 undefined on
input lin
e 63.


Package natbib Warning: Citation `sandmann2022a' on page 1 undefined on
input l
ine 63.


Package natbib Warning: Citation `reutter2021' on page 1 undefined on
input lin
e 63.
```

```
Package natbib Warning: Citation `sandmann2022a' on page 1 undefined on input l
ine 63.


Underfull \vbox (badness 2150) has occurred while \output is active []


Package natbib Warning: Citation `reutter2021' on page 1 undefined on input lin
e 65.


Package natbib Warning: Citation `davies2017' on page 1 undefined on input line
 65.


Package natbib Warning: Citation `sandmann2022MM' on page 1 undefined on input
line 65.


Package natbib Warning: Citation `reutter2021' on page 1 undefined on input lin
e 65.

LaTeX Font Info:    Font shape `T1/Merriwthr-OsF/m/n' will be
(Font)              scaled to size 7.8pt on input line 68.
LaTeX Font Info:    Font shape `T1/Merriwthr-OsF/b/n' will be
(Font)              scaled to size 7.8pt on input line 68.
[1{c:/TeXLive/2022/texmf-var/fonts/map/pdftex/updmap/pdftex.map}


 <./oup.pdf> <./gigascience-logo.pdf>]
LaTeX Font Info:    Font shape `T1/Merriwthr-OsF/m/up' will be
(Font)              scaled to size 7.5pt on input line 69.

Package natbib Warning: Citation `CEcolor2016' on page 2 undefined on input lin
e 71.

LaTeX Font Info:    Font shape `T1/Merriwthr-OsF/b/n' will be
(Font)              scaled to size 8.5pt on input line 75.
LaTeX Font Info:    Font shape `T1/Merriwthr-OsF/b/n' will be
(Font)              scaled to size 7.0pt on input line 79.

Package natbib Warning: Citation `dasilva2017' on page 2 undefined on input lin
e 97.


Package natbib Warning: Citation `dasilva2017' on page 2 undefined on input lin
```

```
e 97.


Package natbib Warning: Citation `rincon2019' on page 2 undefined on
input line
 99.


Package natbib Warning: Citation `rincon2019' on page 2 undefined on
input line
 99.


Package natbib Warning: Citation `sandmann2022a' on page 2 undefined on
input l
ine 101.


Package natbib Warning: Citation `davies2017' on page 2 undefined on
input line
 101.


Package natbib Warning: Citation `sandmann2022a' on page 2 undefined on
input l
ine 101.


Package natbib Warning: Citation `reutter2021' on page 2 undefined on
input lin
e 103.


Package natbib Warning: Citation `reutter2021' on page 2 undefined on
input lin
e 103.

<Figure1.png, id=161, 272.6988pt x 360.8682pt>
File: Figure1.png Graphic file (type png)
<use Figure1.png>
Package pdftex.def Info: Figure1.png  used on input line 113.
(pdftex.def)             Requested size: 488.22787pt x 646.10423pt.
LaTeX Font Info:    Font shape `T1/Merriwthr-OsF/m/n' will be
(Font)              scaled to size 6.0pt on input line 114.
LaTeX Font Info:    Font shape `T1/Merriwthr-OsF/b/n' will be
(Font)              scaled to size 6.0pt on input line 114.
LaTeX Font Info:    Font shape `T1/Merriwthr-OsF/m/it' will be
(Font)              scaled to size 7.8pt on input line 119.
[2] [3 <./Figure1.png>]
LaTeX Font Info:    Font shape `T1/Merriwthr-OsF/b/sl' in size <7.5> not
availa
ble
(Font)              Font shape `T1/Merriwthr-OsF/b/it' tried instead on
input l
```

```
ine 121.
LaTeX Font Info:    Font shape `T1/Merriwthr-OsF/b/it' will be
(Font)              scaled to size 7.5pt on input line 121.

Package natbib Warning: Citation `miller2016' on page 4 undefined on
input line
 122.


Package natbib Warning: Citation `reutter2021' on page 4 undefined on
input lin
e 177.


Package natbib Warning: Citation `miller2016' on page 4 undefined on
input line
 177.

[4]
Underfull \hbox (badness 1478) in paragraph at lines 223--224
[]\T1/Merriwthr-OsF/m/up/7.5 (+20) Interpolating time points be-tween
$\T1/Merr
iwthr-OsF/m/it/7.5 (+20) t[]$ \T1/Merriwthr-OsF/m/up/7.5 (+20) and
$\T1/Merriwt
hr-OsF/m/it/7.5 (+20) t[]$\T1/Merriwthr-OsF/m/up/7.5 (+20) , we as-sume
 []


Package natbib Warning: Citation `reutter2021' on page 5 undefined on
input lin
e 229.


Overfull \hbox (1.01892pt too wide) in paragraph at lines 233--267
[][]
 []


Underfull \vbox (badness 10000) has occurred while \output is active []


Underfull \hbox (badness 10000) in paragraph at lines 273--274
[]\T1/Merriwthr-OsF/m/up/7.5 (+20) Interpolating ad-di-tional time points
-- lo
-cated be-tween
 []


Underfull \hbox (badness 1990) in paragraph at lines 273--274
\T1/Merriwthr-OsF/m/it/7.5 (+20) therapy\T1/Merriwthr-OsF/m/up/7.5 (+20)
.\T1/M
erriwthr-OsF/m/it/7.5 (+20) time\T1/Merriwthr-OsF/m/up/7.5 (+20)
.\T1/Merriwthr
```

```
-OsF/m/it/7.5 (+20) point$ \T1/Merriwthr-OsF/m/up/7.5 (+20) and
$\T1/Merriwthr-
OsF/m/it/7.5 (+20) t[]$ \T1/Merriwthr-OsF/m/up/7.5 (+20) --, we fo-cus on
newly
 de-vel-op-ing
 []


Package natbib Warning: Citation `CEcolor2016' on page 5 undefined on
input lin
e 279.

[5]

Package natbib Warning: Citation `smith2022' on page 6 undefined on input
line
299.


Package natbib Warning: Citation `phylowgs' on page 6 undefined on input
line 3
14.


Package natbib Warning: Citation `canopy' on page 6 undefined on input
line 314
.


Package natbib Warning: Citation `trap' on page 6 undefined on input line
314.


Package natbib Warning: Citation `bubbletree' on page 6 undefined on
input line
 314.


Package natbib Warning: Citation `abscn' on page 6 undefined on input
line 314.



Package natbib Warning: Citation `mapscape' on page 6 undefined on input
line 3
14.


Package natbib Warning: Citation `miller2016' on page 6 undefined on
input line
 316.
```

```
Package natbib Warning: Citation `smith2022' on page 6 undefined on input
line
316.


Underfull \hbox (badness 1194) in paragraph at lines 316--317
\T1/Merriwthr-OsF/m/up/7.5 (+20) by means of fish plots is the R pack-age
fish-
plot [\T1/Merriwthr-OsF/b/n/7.5 (+20) ? \T1/Merriwthr-OsF/m/up/7.5 (+20)
]. The

 []


Package natbib Warning: Citation `miller2016' on page 6 undefined on
input line
 322.


Package natbib Warning: Citation `smith2022' on page 6 undefined on input
line
322.

<Figure2.png, id=199, 1445.4pt x 602.25pt>
File: Figure2.png Graphic file (type png)
<use Figure2.png>
Package pdftex.def Info: Figure2.png  used on input line 326.
(pdftex.def)             Requested size: 488.22787pt x 203.4298pt.

Package natbib Warning: Citation `miller2016' on page 6 undefined on
input line
 332.


Package natbib Warning: Citation `smith2022' on page 6 undefined on input
line
332.


Overfull \hbox (6.15709pt too wide) in paragraph at lines 333--361
[][]
 []

LaTeX Font Info:    Font shape `T1/Merriwthr-OsF/m/up' will be
(Font)              scaled to size 6.0pt on input line 362.
LaTeX Font Info:    External font `cmex10' loaded for size
(Font)              <6> on input line 362.
LaTeX Font Info:    Font shape `T1/Merriwthr-OsF/m/it' will be
(Font)              scaled to size 6.0pt on input line 362.
[6]
<Figure3.png, id=211, 1445.4pt x 722.7pt>
File: Figure3.png Graphic file (type png)
<use Figure3.png>
Package pdftex.def Info: Figure3.png  used on input line 381.
```

```
(pdftex.def)             Requested size: 488.22787pt x 244.11575pt.

Package natbib Warning: Citation `rincon2019' on page 7 undefined on
input line
 390.


Underfull \vbox (badness 10000) has occurred while \output is active []

[7 <./Figure2.png>]
<Figure4.png, id=224, 1445.4pt x 843.15pt>
File: Figure4.png Graphic file (type png)
<use Figure4.png>
Package pdftex.def Info: Figure4.png  used on input line 398.
(pdftex.def)             Requested size: 488.22787pt x 284.80171pt.

Package natbib Warning: Citation `miller2016' on page 8 undefined on
input line
 415.


Package natbib Warning: Citation `smith2022' on page 8 undefined on input
line
415.


Package natbib Warning: Citation `dasilva2017' on page 8 undefined on
input lin
e 417.


Package natbib Warning: Citation `reutter2021' on page 8 undefined on
input lin
e 417.

[8 <./Figure3.png>]

Package natbib Warning: Citation `reutter2021' on page 9 undefined on
input lin
e 419.

[9 <./Figure4.png>]
LaTeX Font Info:    Font shape `TS1/Merriwthr-OsF/m/n' will be
(Font)              scaled to size 7.5pt on input line 442.
LaTeX Font Info:    Trying to load font information for T1+lmtt on input
line 4
43.
(c:/TeXLive/2022/texmf-dist/tex/latex/lm/t1lmtt.fd
File: t1lmtt.fd 2015/05/01 v1.6.1 Font defs for Latin Modern
)
Package microtype Info: Loading generic protrusion settings for font
family
(microtype)             `lmtt' (encoding: T1).
```

```
(microtype)             For optimal results, create family-specific
settings.
(microtype)             See the microtype manual for details.
No file main.bbl.

AED: lastpage setting LastPage
[leftcolumn badness]: 1000000 on 0.90814pt
[rightcolumn badness]: 1000000 on 0.90814pt
[leftcolumn badness]: 1000000 on 0.90814pt
[rightcolumn badness]: 1000000 on 0.90814pt
[leftcolumn badness]: 1000000 on 0.90814pt
[rightcolumn badness]: 1000000 on 0.90814pt
[leftcolumn badness]: 1000000 on 0.90814pt
[rightcolumn badness]: 1000000 on 0.90814pt
[leftcolumn badness]: 1000000 on 0.90814pt
[rightcolumn badness]: 1000000 on 0.90814pt
[leftcolumn badness]: 1000000 on 0.90814pt
[rightcolumn badness]: 1000000 on 0.90814pt
[leftcolumn badness]: 1000000 on 0.90814pt
[rightcolumn badness]: 1000000 on 0.90814pt
[leftcolumn badness]: 1000000 on 0.90814pt
[rightcolumn badness]: 1000000 on 0.90814pt
[leftcolumn badness]: 1000000 on 0.90814pt
[rightcolumn badness]: 1000000 on 0.90814pt
[leftcolumn badness]: 1000000 on 0.90814pt
[rightcolumn badness]: 1000000 on 0.90814pt
[leftcolumn badness]: 1000000 on 0.90814pt
[rightcolumn badness]: 1000000 on 0.90814pt
[leftcolumn badness]: 1000000 on 0.90814pt
[rightcolumn badness]: 1000000 on 0.90814pt
[leftcolumn badness]: 1000000 on 0.90814pt
[rightcolumn badness]: 1000000 on 0.90814pt
[leftcolumn badness]: 1000000 on 0.90814pt
[rightcolumn badness]: 1000000 on 0.90814pt
[leftcolumn badness]: 1000000 on 0.90814pt
[rightcolumn badness]: 1000000 on 0.90814pt
[leftcolumn badness]: 1000000 on 0.90814pt
[rightcolumn badness]: 1000000 on 0.90814pt
[leftcolumn badness]: 1000000 on 0.90814pt
[rightcolumn badness]: 1000000 on 0.90814pt
[leftcolumn badness]: 1000000 on 0.90814pt
[rightcolumn badness]: 1000000 on 0.90814pt
[leftcolumn badness]: 1000000 on 0.90814pt
[rightcolumn badness]: 1000000 on 0.90814pt
- LAST -
Extra skip:0.0pt
Left:693.39186pt/0.0pt
Right:694.3pt/0.0pt
Split: 694.95407pt
Output:676.56221pt
Pageshrink: 0.0pt
Pagestretch: 20.0pt
@colht:715.04684pt
FLUSHEND [output]: 694.95407pt
```

```
FLUSHEND [leftcolumn badness]: 0
FUSHEND [rightcolumn badness]: 0
[10]

Package natbib Warning: There were undefined citations.

(./main.aux)

LaTeX Font Warning: Size substitutions with differences
(Font)              up to 1.0pt have occurred.


LaTeX Font Warning: Some font shapes were not available, defaults
substituted.

Package rerunfilecheck Info: File `main.out' has not changed.
(rerunfilecheck)             Checksum:
6CC38BF4963A267920C9A4578E993736;5263.
 )
Here is how much of TeX's memory you used:
 23935 strings out of 475066
 468610 string characters out of 5782772
 877861 words of memory out of 5000000
 44218 multiletter control sequences out of 15000+600000
 1765130 words of font info for 584 fonts, out of 8000000 for 9000
 1141 hyphenation exceptions out of 8191
 123i,13n,131p,1767b,958s stack positions out of
10000i,1000n,20000p,200000b,200000s
{c:/TeXLive/2022/texmf-dist/fonts/enc/dvips/lm/lm-
ec.enc}{c:/TeXLive/2022/tex
mf-
dist/fonts/enc/dvips/merriweather/merriwthr_posqbl.enc}{c:/TeXLive/2022/t
exm
f-
dist/fonts/enc/dvips/merriweather/merriwthr_owzwzj.enc}<c:/TeXLive/2022/t
exmf
-dist/fonts/type1/sorkin/merriweather/Merriwthr-
Bold.pfb><c:/TeXLive/2022/texmf
-dist/fonts/type1/sorkin/merriweather/Merriwthr-
BoldItalic.pfb><c:/TeXLive/2022
/texmf-dist/fonts/type1/sorkin/merriweather/Merriwthr-
Italic.pfb><c:/TeXLive/20
22/texmf-dist/fonts/type1/sorkin/merriweather/Merriwthr-
Regular.pfb><c:/TeXLive
/2022/texmf-
dist/fonts/type1/public/amsfonts/cm/cmsy5.pfb><c:/TeXLive/2022/texm
f-dist/fonts/type1/public/amsfonts/cm/cmsy6.pfb><c:/TeXLive/2022/texmf-
dist/fon
ts/type1/public/amsfonts/cm/cmsy7.pfb><c:/TeXLive/2022/texmf-
dist/fonts/type1/p
ublic/amsfonts/euler/euex7.pfb><c:/TeXLive/2022/texmf-
dist/fonts/type1/public/a
msfonts/euler/euex8.pfb><c:/TeXLive/2022/texmf-
dist/fonts/type1/public/lm/lmtt8
```

```
.pfb>
Output written on main.pdf (10 pages, 3189398 bytes).
PDF statistics:
 311 PDF objects out of 1000 (max. 8388607)
 266 compressed objects within 3 object streams
 59 named destinations out of 1000 (max. 500000)
 207655 words of extra memory for PDF output out of 221844 (max.
10000000)
```

Manuscript
Click here to access/download;Manuscript;clevRvis_main.pdf

GigaScience, 2017, 1–11

doi: xx.xxxx/xxxx
Manuscript in Preparation
Technical Note

TECHNICAL NOTE

clevRvis: Visualization Techniques for Clonal Evolution

Sarah Sandmann[1,*], Clara Inserte[1] and Julian Varghese[1]

[1]Institute of Medical Informatics, University of Münster, Münster, Germany

[*]sarah.sandmann@uni-muenster.de

Abstract

Background: A thorough analysis of clonal evolution commonly requires integration of diverse sources of data, e.g. karyotyping, next-generation sequencing and clinical information. Subsequent to actual reconstruction of clonal evolution, detailed analysis and interpretation of the results is essential. Often, however, only few tumor samples per patient are available. Thus, information on clonal development and therapy effect may be incomplete. Furthermore, analysis of bi-allelic events – considered of high relevance with respect to disease course – can commonly only be realized by time-consuming analysis of the raw results and even raw sequencing data. **Results:** We developed clevRvis, an R/Bioconductor package providing an extensive set of visualization techniques for clonal evolution. In addition to common approaches for visualization, clevRvis offers a unique option for allele-aware representation: plaice plots. Bi-allelic events may be visualized and inspected at a glance. Analyzing four public data sets, we show that plaice plots help to gain new insights into tumor development and investigate hypotheses on disease progression and therapy resistance. In addition to a graphical user interface, automatic phylogeny-aware color coding of the plots and an approach to explore alternative trees, clevRvis provides two algorithms for fully automatic time point interpolation and therapy effect estimation. Analyzing two public data sets, we show that both approaches allow for valid approximation of a tumor's development in between measured time points. **Conclusions:** clevRvis represents a novel option for user-friendly analysis of clonal evolution, contributing to gaining new insights into tumor development.

Key words: Clonal evolution; tumor development; visualization; cancer cell fraction; bi-allelic events; therapy effect

Background

For many types of cancer, determining their mutational profile is a crucial step, having an impact on diagnosis as well as treatment. In multiple myeloma (MM), for example, cytogenetic aberrations have been found to have significant impact on prognosis and are thus considered in the Revised International Staging System (R-ISS) for risk stratification [1, 2]. Similarly, in myelodysplastic syndromes (MDS), patients are commonly stratified according to the Revised International Prognostic Scoring System (IPSS-R), evaluating – among others – cytogenetic abnormalities [3]. Recently, it has been shown that the analysis of point mutations and small insertions/deletions (indels) even allows for identification of clinically relevant subgroups within low-risk MDS patients [4].

In addition to information on the bare presence or absence of variants, their development over time, the clonal evolution [5], is of major importance in several diseases, e.g. MDS, acute myeloid leukemia (AML) or Burkitt lymphoma (BL) [6, 7, 8]. For a thorough analysis of clonal evolution, all variants ranging from point mutations to aberrations affecting whole chromosomes should be taken into account. Commonly, various sources of data are considered to allow for valid detection of these variants, e.g. karyotyping, fluorescence *in sito* hybridization, microarrays like SNP-arrays or array-CGH (aCGH), next-generation sequencing (NGS) and Sanger sequencing. Integrating all of these data, clonal evolution may be reconstructed [7].

Studying clonal evolution in more detail, a diverse set of analyses can be performed: The model of clonal evolution (linear vs branched dependent vs branched independent; of note,

Compiled on: January 23, 2023.
Draft manuscript prepared by the author.

Table 1. Overview of the data sets analyzed with clevRvis. BL – Burkitt lymphoma; CLL – chronic lymphatic leukemia; FISH – fluorescence *in situ* hybridization; MDS – myelodysplastic syndromes; MPN – myeloid neoplasia; tNGS – targeted next-generation sequencing; WES – whole-exome sequencing.

Data set	Disease	Patients	#Time points (median [min-max])	#Clones (median [min-max])	Clonal evolution models	Data types
1	MDS	11	5 [5-30]	5 [1-9]	linear, branched dependent, branched independent	karyotyping, FISH, SNP-array, WES, tNGS
2	CLL	2	9.5 [4-15]	4 [3-5]	linear, branched dependent	karyotyping, FISH, WES, tNGS
3	MPN	8	2 [1-5]	5.5 [2-11]	linear, branched dependent, branched independent	karyotyping, FISH, aCGH, tNGS
4	BL	10	1.5 [1-2]	9 [7-17]	linear, branched dependent	FISH, SNP-array WES, tNGS, Sanger

neutral and punctuated evolution will be considered special cases of branched and linear evolution) [8, 9] can be determined, correlation to blood parameters, therapy resistance and disease progression investigated [10] and patterns characterizing subgroups of patients explored. For example, by detailed analysis of clonal evolution, evidence was found that relapsing BL is associated with the presence of clones, featuring double-hit events in *TP53* [8].

However, these analyses may be hampered by different aspects: 1) Variants are only detected at few time points, providing an incomplete representation of the disease course. 2) No information on a therapy's effect on clonal evolution is available. 3) Evaluation of bi-allelic events requires tedious manual work, analyzing raw sequencing data and detailed variant calling information.

To overcome these obstacles, we developed clevRvis – an R/Bioconductor package for clonal evolution in R, providing innovative visualization techniques. In addition to common R functions, clevRvis offers a web-based graphical user interface, allowing usage not just by computer scientists, but also physicians and biologists. Our approach contains fully automatic algorithms for interpolating additional time points as well as estimating therapy effect. Evaluating two real, publically available data sets from different disease entities, characterized by a high number of measured time points, we show that both estimation approaches generate valid results.

clevRvis generates three different types of plots: 1) shark plots (a graph-based representation of clonal evolution), 2) dolphin plots (a fish plot-like representation, optionally also considering interpolated time points and estimated therapy effect), 3) plaice plots (a novel type of plots allowing for detection of bi-allelic events at a glance). All plots generated by clevRvis are highly customizable. Following recommendations for graphical strategies in clonal evolution [11], we implemented an algorithm for phylogeny-aware color coding of the clones to obtain optimal visualization. In addition, alternative phylogenetic trees can be determined and explored interactively. By analysis of four public data sets, we show that visualization with clevRvis outperforms common alternative approaches. Additionally, we show the added value of plaice plots.

Methods

Data sets analyzed

We analyze four real data sets, containing detailed information on the clonal evolution of $n = 31$ patients. Table 1 provides an overview of the data sets and their main characteristics.

The first set covers data from 11 patients with MDS. Clonal evolution was reconstructed based on karyotyping, FISH, SNP-array, whole-exome sequencing (WES) and ultra-deep targeted next-generation sequencing (tNGS) data [6]. The data set is characterized by a high number of time points (up to 30) and a relatively low number of clones. All major models of clonal evolution are present. Six patients received supportive care only, while five additionally received lenalidomide, which is expected to impact clonal evolution. The data set serves a test case, exploring options for visualizing clonal evolution and validating our approaches for automatic time point interpolation and therapy effect estimation (information on how input data for the analysis with clevRvis was derived from the publication by da Silva-Coelho et al. [6] is available in Additional file 1, section 1.1, input data itself is available in Additional file 2).

The second set covers data from 2 patients with chronic lymphatic leukemia (CLL). Clonal evolution was reconstructed based on FISH, WES and tNGS data [12]. The data set provides detailed information on follow-up – especially on therapies applied – for up to 12 years, as well as regular variant detection throughout the whole time of follow-up. Thereby, this data set serves a second test case for the analysis with clevRvis, considering time point interpolation and therapy effect estimation in a different disease entity and a different source of data (information on how input data for the analysis with clevRvis was derived from the publication by González-Rincón et al. [12] is available in Additional file 1, section 1.2, input data itself is available in Additional file 3).

The third set covers data from 8 patients with myeloid neoplasia (MPN). Clonal evolution was reconstructed based on karyotyping, FISH, aCGH and tNGS data [7]. Presence of 1 to 5 time points and an increased number of clones (one case of branched dependent evolution can be specified more precisely as neutral evolution [9]), pose a challenge for visualization. Partly, no samples were collected towards the end of therapy, requiring interpolation of additional time points and estimating the effect of therapy (information on how input data for the analysis with clevRvis was derived from the publication by Sandmann et al. [7] is available in Additional file 1, section 1.3, input data itself is available in Additional file 4).

The fourth set covers data from 10 patients with BL. Clonal evolution was reconstructed based on FISH, SNP-array, WES, tNGS and Sanger sequencing data [8]. The data set is characterized by a low number of time points (1 for non-relapsing patients, 2 for relapsing patients). While therapy is known to have been applied, no data showing its effect on clonal evolution is available. Thus, circumstances require interpolation of time points and estimation of therapy effect for all patients. Additionally, samples feature a high number of clones (up to 17), which is expected to be a major challenge for visualization (information on how input data for the analysis with clevRvis was derived from the publication by Reutter et al. [8] is available in Additional file 1, section 1.4, input data itself is available in Additional file 5).

All data sets contain information on detected small variants

(SNVs and indels) as well as large variants (structural variants SVs and copy number variants CNVs) for every patient. Analysis of clonal evolution always involves integration of various data sources and partly overlapping variants. Thereby, the detection of bi-allelic variants and their evaluation by plaice plots is key for all data sets.

clevRvis

clevRvis provides an extensive set of visualization techniques for clonal evolution. An overview of the analysis pipeline is provided in Figure 1 (screenshots of the software are available in Additional file 1, Figures S1-S8).

For the subsequent description of the analyses performed by clevRvis, we use the following definitions: $ccf(c, t_i)$ is defined as the cancer cell fraction (CCF) of clone c, with $c \in \{1, \ldots, n\}$, at time point t_i, with $t_i \in \{t_1, \ldots, t_m\}$. $parent(c)$ is defined as the parent-clone of clone c, $children(c)$ as the children-clone(s) of clone c. For a clone c developing from normal cells, $parent(c) = 0$. All clones developing from normal cells are defined by $children(0)$. $ccf'(c, t_i)$ is defined as the difference in CCFs of a clone c at time point t_i. Thus, $ccf'(c, t_i) = ccf(c, t_i) - \sum_{j \in children(c)} ccf(j, t_i)$.

Validity check

clevRvis requires the upload of a *CCF table*, containing information on the CCF of every clone at every time point, optionally also including information on parental relations. In an interactive dialogue, the user can subsequently – if this information has not been uploaded – define the parental relations for every clone listed in the CCF table. Normal cells as well as clones different from the considered one may be selected. Upon submitting this information to generate the initial *seaObject*, a validity check is performed, adapting and extending the check performed by fishplot [13] (see Algorithm 1).

Algorithm 1 validity check

```
for each t_i in {t_1, ..., t_m} do
    for each c in {1, ..., n} do
        if ccf(c, t_i) < ∑_{j∈children(c)} ccf(j, t_i) then
            return check.failed
        if ∑_{j∈children(0)} ccf(j, t_i) > 100 then
            return check.failed
        if ccf(c, t_i) > 0 and ccf(c, t_{i+1}) = 0 and ccf(c, t_{i+2}) > 0 then
            return check.failed
        if ccf'(c, t_i) > 0 and ccf'(c, t_{i+1}) = 0 and ccf'(c, t_{i+2}) > 0 then
            return check.failed
return check.passed
```

Summing up, 1) children-clones cannot exceed their parents, 2) clones developing from normal cells cannot add up to more than 100%, 3) a clone cannot reappear, 4) a parent-clone being thoroughly replaced by its children-clone(s) cannot reappear.

Exploring alternative trees

Alternative parental relations, resulting in alternative clonal evolution trees, can be explored interactively using clevRvis. Considering n clones, a parental relations vector of length n has to be defined. For every clone, 1 out of n options can be chosen ($n - 1$ different clones + normal cells), which results in n^n permutations. To optimize run-time, filtration of clearly invalid options is performed prior to clevRvis' thorough validity check (see Algorithm 2).

Algorithm 2 exploring alternative trees

```
for each c in {1, ..., n} do
    for each t_i in {t_1, ..., t_m} do
        if ccf(c, t_i) < any other ccf(j, t_i) then
            return c! = parent(j)
for each remaining c in {1, ..., n} do
    for each remaining t_i in {t_1, ..., t_m} do
        if parent(j) can only be c then
            if ccf(c, t_i) − ccf(j, t_i) < any other ccf(k, t_i) then
                return c! = parent(k)
for each remaining.parental.relations
    apply Algorithm 1 validity check
return valid.parental.relations
```

As a first filtration step, possible linear relations are investigated: If a clone c has, at any time point, a lower CCF compared to clone j at the same time point, c cannot be the parent of j. Subsequently, possible branched relations are taken into account: If a clone j clearly develops from clone c, an additional clone k can – as a branch – only develop from c if $ccf(c, t_i) - ccf(j, t_i) \geq ccf(k, t_i)$ for every time point t_i. On the basis of the remaining filtered options, permutations are determined and a thorough validity check (see Algorithm 1) is performed (maximum: 20,000 permutations). All valid parental relations are reported and alternative trees can be explored subsequently.

Time point interpolation

The initially generated seaObject may be extended by additional interpolated time points. The general idea of estimating auxiliary time points to improve visualization of clonal evolution was first outlined by Reutter et al. [8]. However, this initial approach mainly focused on improving figures generated by fishplot [13], considering only the difference in CCFs at a later measured time point. Furthermore, it did not contain any algorithm for automatic time point interpolation. Evaluating different scenarios of clonal evolution, we implemented an improved, fully automatic approach in clevRvis (see Algorithm 3).

The number of time points interpolated by clevRvis depends on the clonal evolution being analyzed and cannot be defined by a user. We differentiate between interpolating development of a tumor prior to the first measured time point, and interpolating development between two measured time points. By default, all interpolated time points are evenly distributed (a detailed description on how to implement skewed events is available in Additional file 1, section 1.6).

We assume that clones of the same nested level developed at approximately the same time. A higher nested level indicates development at a later time. For example, in a linear evolution with clone B developing from clone A, it is sensible to assume that clone A developed first and expanded. Over time, a cell of clone A acquired additional mutations, finally resulting in the formation of clone B.

Interpolating time points prior to t_1, we focus on the difference in CCFs $ccf'(c, t_1)$ for all clones present at t_1 (*initial.clones*). The number of interpolated time points is defined by the maximum nested level of the initially present clones. As the clone(s) with the highest nested level are assumed to have developed last, ccf' as well as ccf are set to zero for the first (and all subsequent) interpolated time points. The CCFs of the remaining initial clones are updated. The procedure is repeated with the second highest nested level etc. until only clones with nested level 0 remain.

Interpolating time points between t_i and t_{i+1}, we assume linear development of CCF for all clones already present at t_i (*old.clones*). For newly developing clones (*new.clones*), we determine their nested levels. The number of unique nested lev-

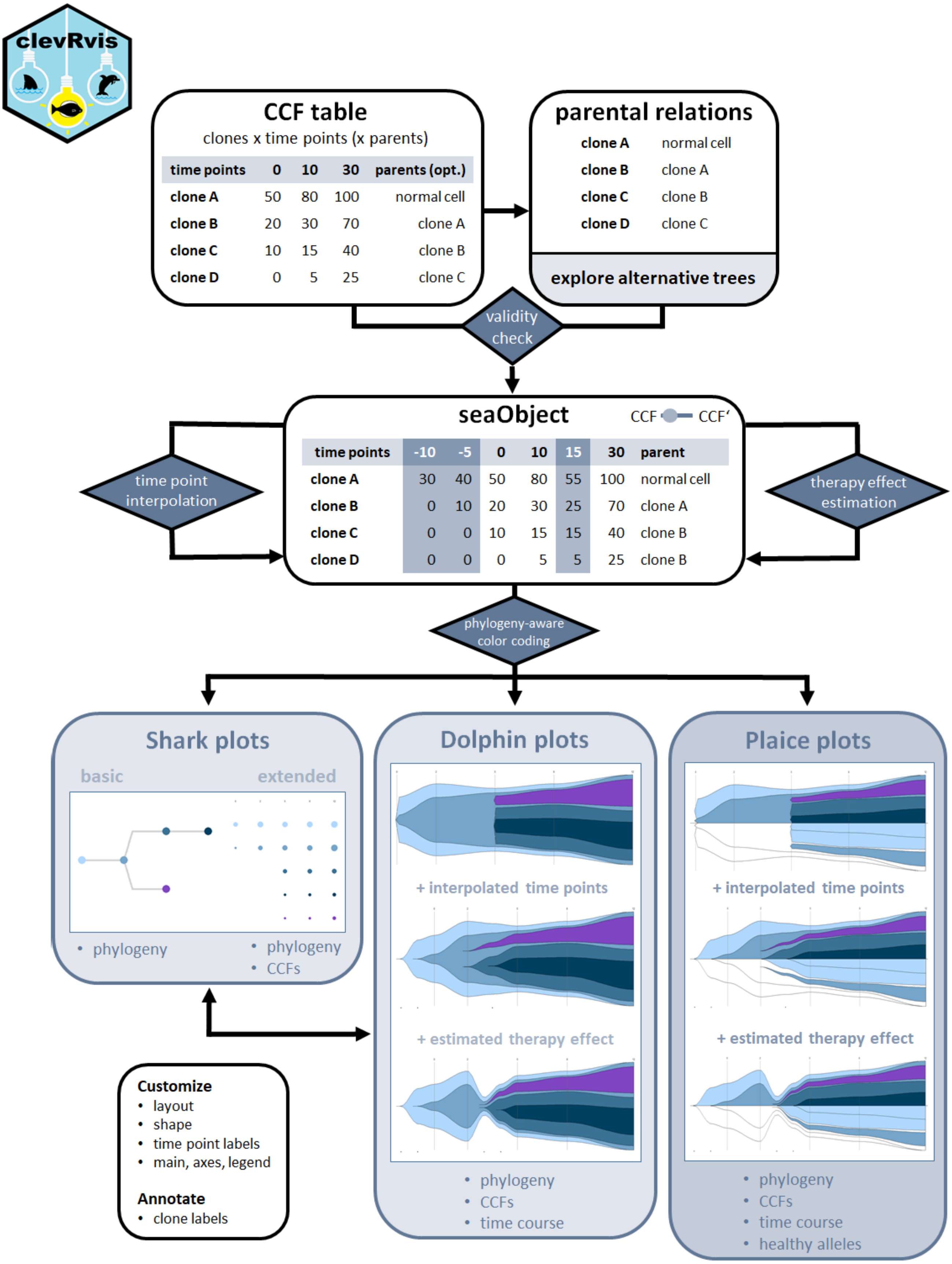

Figure 1. Overview of the analyses performed by clevRvis. Based on a CCF table and information on parental relations, a validity check is performed. For the resulting seaObject, additional time points may be interpolated and therapy effect may be estimated. Using phylogeny-aware color coding, shark plots, dolphin plots and plaice plots can be generated.

Algorithm 3 time point interpolation

if interpolate initial development **then**
  initial.clones ← all clones *c* where $ccf(c, t_1) \neq 0$
  nested.levels ← nested levels for all *initial.clones*
  new.time.points ← *maximum*(*nested.levels*)
  for each *new.time.point* **in** {1, ..., *new.time.points*} **do**
  // 1 refers to time point closest to t_1
    for each initial clone *c* with *maximum*(*nested.levels*) **do**
      $ccf(c, new.time.point) \leftarrow ccf'(c, new.time.point) \leftarrow 0$
    determine *ccf*(*remaining.initial.clones*, *new.time.point*) based on *ccf′*(*initial.clones*, *new.time.point*)
    remove *maximum*(*nested.levels*)

if interpolate development between measured time points **then**
  old.clones ← all clones *c* where $ccf(c, t_i) \neq 0$
  new.clones ← all clones *c* where $ccf(c, t_i) = 0$ **and** $ccf(c, t_{i+1}) \neq 0$
  new.unique.nested.levels ← unique nested levels for *new.clones*
  new.time.points ← *new.unique.nested.levels* − 1
    for each clone *c* **in** *old.clones* **do**
      ccf(*c*, *new.time.points*) ← linear development from $ccf(c, t_i)$ to $ccf(c, t_{i+1})$
    for each clone *c* **in** *new.clones* **do**
      z ← number of *new.unique.nested.levels* < *nested.level*(*c*)
      for each *new.time.point* **in** {1, ..., *z*} **do**
        ccf(*c*, *new.time.point*) ← 0
      ccf(*c*, *remaining.new.time.points*) ← linear development from *ccf*(*c*, *z*) to $ccf(c, t_{i+1})$

return updated *ccf*

Algorithm 4 therapy effect estimation

old.clones ← all clones *c* where $ccf(c, t_i) \neq 0$
new.clones ← all clones *c* where $ccf(c, t_i) = 0$ **and** $ccf(c, t_{i+1}) \neq 0$
for each clone *c* **in** *old.clones* **do**
  ccf′(*c*, *therapy.time.point*) ← *minimum*($ccf'(c, t_i), ccf'(c, t_{i+1})$)
determine *ccf*(*old.clones*, *therapy.time.point*) based on *ccf′*(*old.clones*, *therapy.time.point*)
ccf(*new.clones*, *therapy.time.point*) ← 0

if additional time point interpolation **then**
  therapy.clones ← all clones *c* where *ccf*(*c*, *therapy.time.point*) ≠ 0
  final.clones ← all clones *c* where $ccf(c, t_{i+1}) \neq 0$
  new.final.clones ← all clones *c* where $ccf(c, t_{i+1}) \neq 0$ **and** *ccf*(*c*, *therapy.time.point*) = 0
  nested.levels ← nested level for all *new.final.clones*
  recalculate *nested.levels* ignoring *therapy.clones*
  new.time.points ← *maximum*(*recalculated.nested.levels*)
  for each clone *c* **in** *new.final.clones* **do**
    z ← *recalculated.nested.level*(*c*)
    for each *new.time.point* **in** {1, ..., *z*} **do**
    // 1 refers to time point closest to *therapy.time.point*
      ccf(*c*, *new.time.point*) ← *ccf′*(*c*, *new.time.point*) ← 0
    if *c* has no children **then**
      ccf(*c*, *remaining.new.time.points*) ← linear development from *ccf*(*c*, *z*) to $ccf(c, t_{i+1})$
    if *c* has children **then**
      ccf′(*c*, *remaining.new.time.points*) ← $ccf'(c, t_{i+1})$
  determine *ccf*(*remaining.final.clones*, *new.time.points*) based on *ccf′*(*final.clones*, *new.time.points*)
return updated *ccf*

els (*new.unique.nested.levels*) - 1 defines the number of interpolated time points. We stick to our assumption that clones with a higher nested level developed at a later time point. Thus, newly developing clone(s) with the lowest nested level are considered first. Linear development between t_i and t_{i+1} is assumed. For all clones with the second lowest nested level, CCF at the first interpolated time point is set to zero. Linear development is assumed for the remaining interpolated time points up to t_{i+1}. The procedure is repeated with the third lowest nested level etc. until only clones with the highest *new.unique.nested.levels* remain.

Of note, to generate smoother dolphin- and plaice plots in R, for all clones 0.1 is added to the (interpolated) time point prior to their first appearance.

Therapy effect estimation

If a therapy is applied, interpolating time points is assumed to be insufficient to approach the development of CCFs over time properly. In the absence of therapy, the overall tumor load is not expected to decrease. Any decrease in CCF differences over time is assumed to be caused by the expansion of superior children-clones. In the presence of therapy, however, we assume that any observed decrease in CCF differences is due to therapy. This general idea was first outlined by Reutter et al. [8] as well. In clevRvis, we implemented an updated, fully automatic approach for therapy effect estimation (see Algorithm 4).

Estimating therapy effect, a single time point is added, corresponding to the clonal composition towards the end of therapy. The precise time point is user-definable (default: evenly distributed). In case new clones developed, enabling additional time point interpolation is recommended.

To estimate the effect of a therapy that has been applied between t_i and t_{i+1}, we focus on the difference in CCFs $ccf'(c, t_i)$ for all clones present at t_i (*old.clones*). Assuming that no increase in tumor load is observed during therapy, the minimum of $ccf'(c, t_i)$ and $ccf'(c, t_{i+1})$ is determined. For all newly developing clones (*new.clones*), we assume that they developed after the application of therapy. Thus, CCFs are set to zero for the estimated time point (*therapy.time.point*).

Interpolating additional time points – located between *therapy.time.point* and t_{i+1} –, we focus on newly developing clones. For all clones only present at t_{i+1} (*new.final.clones*), we recalculated the nested levels, ignoring clones already present at *therapy.time.point*. As an example, we consider three clones A, B and C that develop linearly. While A and B are already present at t_i, clone C only emerges at t_{i+1}. Another clone D develops from normal cells and – just like clone C – only emerges at t_{i+1}. Nested levels of clones C and D are two and zero respectively. However, development of both clones is just 'one step' compared to the starting position at t_i. Therefore, the recalculated nested levels, ignoring clones A and B, are zero for both C and D.

Sticking to our main assumption that clones with a higher (recalculated) nested level develop at a later time, additional time points are interpolated. The number of time points added is defined by the maximum recalculated nested level. Newly developing clone(s) with the lowest recalculated nested level are considered first. If a clone *c* has no children, linear development between *therapy.time.point* and t_{i+1} is assumed. Otherwise, we assume that the CCF for clone *c* at all interpolated time points is defined by $ccf'(c, t_{i+1})$. For all clones with the second lowest recalculated nested level, CCF at the first interpolated time point is set to zero. Subsequently, CCFs for the remaining time points are interpolated considering either linear development or $ccf'(c, t_{i+1})$. The procedure is repeated with the third lowest nested level etc. until only clones with the highest *recalculated.nested.level* remain.

Phylogeny-aware color coding

Based on an evaluation of graphical strategies for visualizing clonal evolution published by Krzywinski [11], we developed an approach for automatic phylogeny-aware color coding. Our

approach sticks to the following rules:

i. The higher the nested level of a clone, the darker the hue.
ii. Clones of the same branch are colored by a similar hue.
iii. Clones on two (or more) branches with a common ancestor (branched dependent evolution) are colored by similar, but diverging colors.
iv. Clones on two (or more) branches without a common ancestor (branched independent evolution) are colored by colors of maximum difference.

The exact range of the color palette is dynamically determined based on the clonal evolution provided as input data (number of clones, maximum nested level). Thereby, options for differentiating between closely related clones are optimized. clevRvis supports a maximum of 25 independent clones, developing from normal cells, and an unlimited number of related clones.

Shark plots

Shark plots serve a basic, raw visualization of clonal evolution (see Figure 1). Using a classical graph approach, clones are represented by nodes, parental relations by edges. Thus, the phylogeny can be directly deduced from shark plots.

Optionally, shark plots can be extended to provide information on CCFs as well. Clones are additionally visualized next to the actual shark plot. The size of each clone is correlated with its CCF. Time points are plotted next to each other.

If shark plots are chosen to be plotted along with dolphin plots, both plots are connected interactively. By hovering over one of the clones, it is automatically highlighted in both, shark and dolphin plot.

Dolphin plots

An advanced visualization of clonal evolution is realized by dolphin plots, mainly corresponding to well-established fish plots. The development of each clone over time is displayed on the x-axis, the CCFs on the y-axis. Thereby, information on phylogeny, CCFs and time course characterizing a clonal evolution are jointly visualized in a single plot. Several basic options for customizing dolphin plots are available, e.g. switching between spline and polygon shape or separating independent clones. Additionally, a user may choose between standard centered visualization of clonal evolution and bottom layout. This causes the clones to develop as “lying” on the x-axis (similar to visualization of clonal evolution by timescape [14]). Automatically, the longest branch is chosen to be plotted on the bottom, while the remaining branches are added on top.

If a seaObject has been extended by additional interpolated time points and/or estimated therapy effect, both can be visualized by dolphin plots. Customizable labels can be added to distinguish between measured vs estimated time points.

Plaice plots

Plaice plots represent a derivative of dolphin plots, resp. fish plots, developed to improve visualization of bi-allelic events. Instead of one, we consider two “flatfish” (=plaice) that are mirrored above and below the y-axis.

Common clonal evolution is visualized only in the upper plot in bottom layout. Similar to dolphin plots, a user may choose between spline vs polygon shape and separating independent clones (recommended). The fraction of remaining healthy alleles is visualized in the lower plot. For this purpose, a mirrored presentation of clonal evolution in bottom layout is plotted. By default, clones in this part of the plot are not colored, representing a starting position of 100% healthy alleles.

As an example, we consider linear clonal evolution of 2 clones – A and B. Clone A is characterized by a mutation in *TP53*, clone B by a deletion 17p. The two variants are overlapping. If they affect different alleles, no healthy allele of *TP53* remains in all cells belonging to clone B. Thus, a user may chose to color clone B in the lower plot to indicate a decrease in healthy alleles of *TP53* as the CCF of clone B increases. The clone should, however, be colored in the hue of clone A. Thereby, the bi-allelic event leading to *TP53* deficiency is linked to the clone that is originally characterized by a mutation in this gene. Instead, if both variants affect the same allele, one healthy copy of *TP53* remains – independent of the CCF of clones A and B. Thus, no clone should be colored in the lower plot. In addition to bi-allelic events, variants affecting the only available X- or Y-chromosome in male subjects can equally be visualized using plaice plots. Detailed information on the recommend color coding of plaice plots is provided in Additional file 1, section 1.7.

Just like dolphin plots, plaice plots provide all options for visualizing data on additional interpolated time points (recommended) and estimated therapy effect.

Comparison to common approaches

Several approaches exist for visualizing clonal evolution. A majority of tools performing clonal evolution tree reconstruction, e.g. PhyloWGS [15], Canopy [16] or TRaP [17], provide a basic graph-based visualization that is automatically generated when performing the analysis. These plots, however, are commonly not customizable and will, thus, not be further considered in this work. Additionally, tools like BubbleTree[18] and AbsCN-seq [19], estimating and visualizing tumor purity, ploidy and copy numbers, are not considered due to different scope. The tool MapScape [20] is not considered as visualization focuses on spatial clonal evolution, linking anatomical images to tumor samples

A commonly used approach for visualizing clonal evolution by means of fish plots is the R package fishplot [13]. The R/Bioconductor package timescape [14] provides an alternative approach, visualizing clonal evolution by interactive fish plots linked to standard graphs. A detailed evaluation of both approaches in comparison to our novel approach clevRvis is performed.

We could not identify any tool visualizing bi-allelic events in clonal evolution for comparison with our plaice plot module. Therefore, representation by plaice plots is compared to dolphin/fish plots as well as manual evaluation of bi-allelic events.

Results

We apply clevRvis to four real, publically available data sets and compare performance of our approach to the commonly used R packages fishplot [13] and timescape [14]. Results for three exemplary samples are visualized in Figure 2. Detailed results, considering all 31 samples, are available in Additional file 1 (data set 1: Figures S11-S21; data set 2: Figures S22, S23; data set 3: Figures S24-S34; data set 4: Figures S35-S45). Main analysis features of all three algorithms are summed up in Table 2.

All three approaches are able to generate fish plots for visualization of clonal evolution (called “dolphin plots” in clevRvis). Additionally, timescape and clevRvis generate graphs (“shark plots” in clevRvis), representing the underlying phylogeny. However, clevRvis is the only approach providing an option to visualize information on healthy alleles and their development over time in terms of clonal evolution (“plaice plots”).

It can be observed that the three main models of clonal evo-

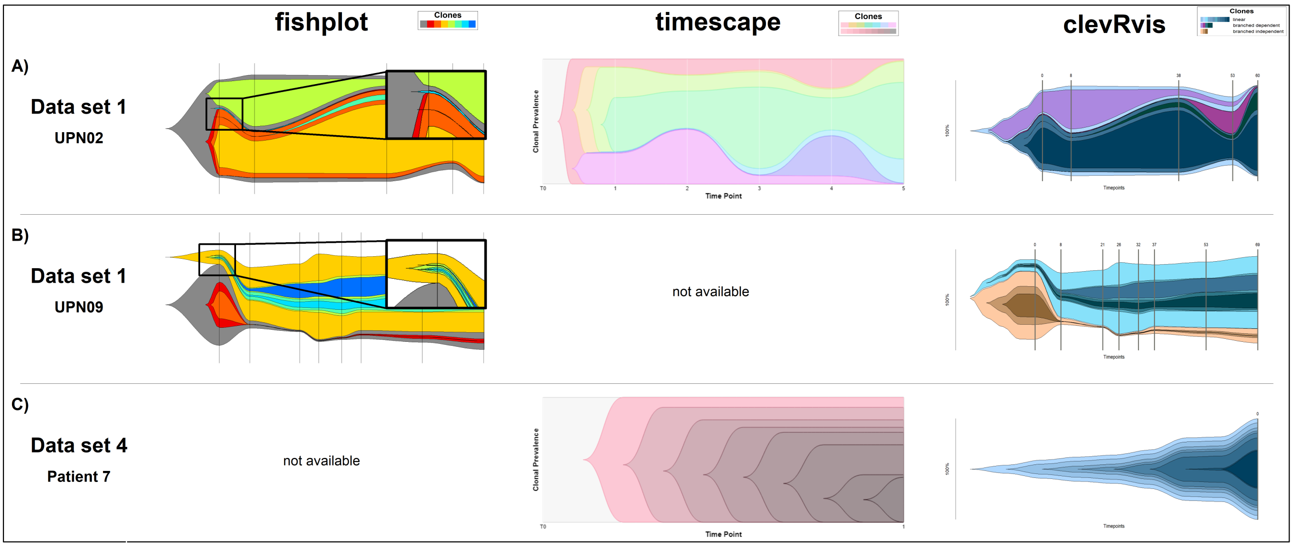

Figure 2. Visualization of clonal evolution using fish plots, comparing the approaches fishplot, timescape and clevRvis. A) Data set 1, UPN02: branched dependent evolution can be visualized by all three tools. However, starting points of two clones are not correctly displayed using fishplot. B) Data set 1, UPN09: branched independent evolution can only be visualized by fishplot and clevRvis. The starting point of one clone is not correctly displayed using fishplot. C) Data set 4, Patient 7: linear evolution based on a single time point can only be visualized by timescape and clevRvis.

Table 2. Overview of main analysis features, comparing the approaches fishplot [13], timescape [14] and clevRvis.

	fishplot	timescape	clevRvis
**Visualization options**			
Graph		x	x
Fish plot	x	x	x
+ healthy alleles			x
**Clonal evolution models**			
Linear	x	x	x
Branched dependent	x	x	x
Branched independent	x		x
**Basic features**			
Graphical user interface			x
Validity check[1]	x		x
Interactive plots		x	x
Single time point		x	x
Single clone	x		x
**Advanced features**			
Exploring alternative trees			x
Time point interpolation			x
Therapy effect estimation			x
Phylogeny-aware color coding[2]			x

[1]We consider a validity check to be able to detect errors in the logic of clonal evolution, e.g. the CCF of a children-clone exceeding the CCF of its parent-clone, or CCFs at one time point summing up to > 100%.
[2]We consider phylogeny-aware color coding to indicate the degree of relatedness of two clones. This includes clones developing in a linear, branched dependent and branched independent manner.

lution – linear, branched dependent and branched independent – can generally be considered by all three tools. The only exception is branched independent evolution, which cannot be visualized using timescape (Figure 2B; data set 1: patients UPN08, 09, 10, Figures S18-S20; data set 3: patient 4, Figures S27-S29). The tool mandatorily requires all clones to be present in a single tree. Additionally, timescape is not capable of visualizing clonal evolution, if only a single clone is present (data set 1: patient UPN04, Figure S14). Fishplot, on the contrary, struggles with visualizing data available at a single time point (Figure 2C; data set 3: patients 1-3, Figures S24-S26; data set 4: patients 6-10, Figures S40-S45). Furthermore, the visualization with fishplot partly suggests a wrong starting point of the clone, despite correct definition of the input (Figure 2A and 2B; data set 1: patients UPN02, 03, 07, 09, 10, Figures S12, S13, S17, S19, S20; data set 4: patients 2 and 5, Figures S36, S39).

clevRvis is the only approach providing a graphical user interface, allowing for user-friendly analysis of clonal evolution. Plots can be easily customized, e.g. interactively moving labels of the clones along the x- and y-coordinates, or picking colors and transparency levels for the clones' borders from a wide palette. By default, all plots are interactive. When hovering over a clone, its CCF is displayed and the clone is highlighted – in case of shark and dolphin plots, which are interactively connected, in both plots. Moreover, clevRvis is the only tool providing advanced features, exceeding the basic visualization of clonal evolution. These include fully automatic algorithms for time point interpolation and therapy effect estimation. Phylogeny-aware color coding is implemented as well as an algorithm for exploring alternative trees.

A detailed description on the usage of clevRvis, including exemplary input files and executable examples, is provided along with the package (manuals and vignette). A tutorial, including a complete walk-through, is additionally provided in the shiny-app.

Time point interpolation and therapy effect estimation

clevRvis provides algorithms for approximating the development of clonal evolution in between two measured time points, by interpolating additional time points as well as estimating the effect of a therapy applied. To investigate performance of our algorithms, we consider data sets 1 and 2. Results considering 4 exemplary patients are summed up in Figure 3.

Patients in data set 1 are characterized by a high number of measured time points (up to 30). Analysis with clevRvis is performed twice: 1) evaluating all measured time points; 2) evaluating the first and last measured time point only. Six patients (UPN03, 04, 05, 06, 07 and 11) received supportive care only. Thus, development of clonal evolution is best approximated by time point interpolation. For patient UPN07 (Figure 3A) it can be observed that despite a certain simplification in the develop-

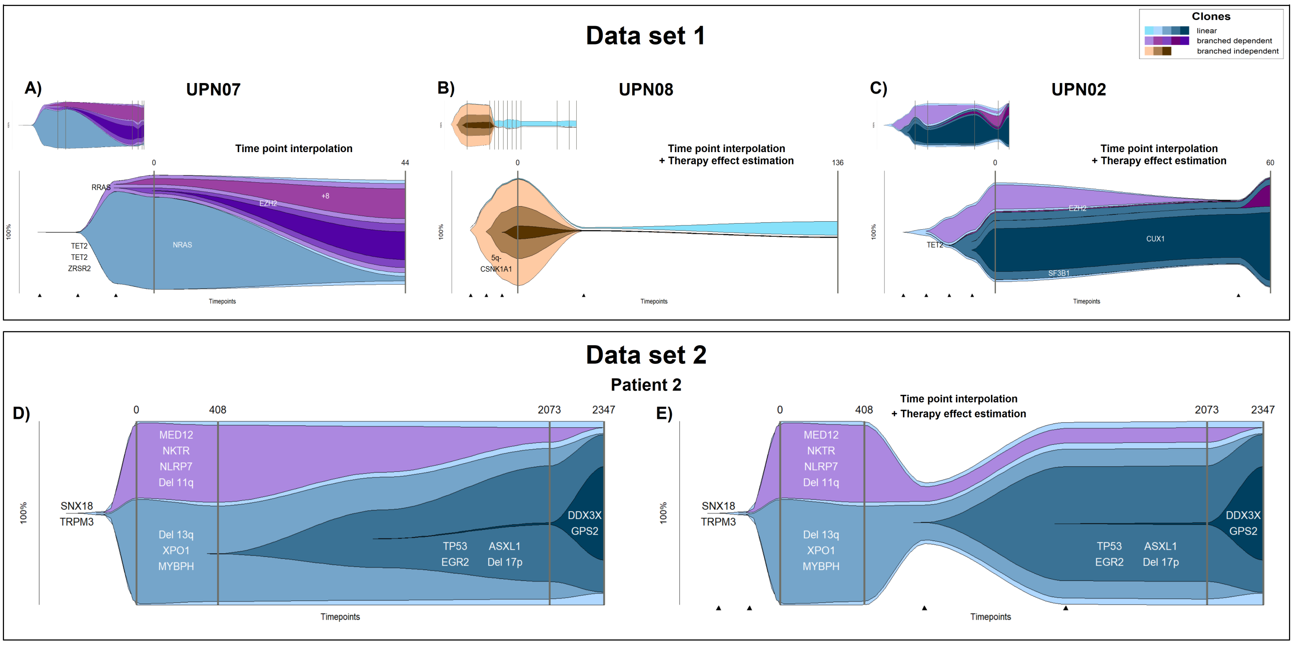

Figure 3. Visualization of clonal evolution using dolphin plots. A) Data set 1, UPN07: analysis of 6 time points vs analysis of 2 time points and additional time point interpolation. B) Data set 1, UPN08: analysis of 12 measured time points vs analysis of 2 measured time points, additional time point interpolation and therapy effect estimation. C) Data set 1, UPN02: analysis of 5 measured time points vs analysis of 2 measured time points, additional time point interpolation and therapy effect estimation. D) Data set 2, patient 2: analysis of 4 measured time points. E) Data set 2, patient 2: analysis of 4 measured time points and additional therapy effect estimation. Note: for all plots, time point interpolation is enabled to improve visualization of newly developing clones prior to the first measured time point.

ment, clonal evolution estimated by clevRvis is highly comparable to the original course. Similar results can be observed for patient UPN08. As the patient was treated with lenalidomide, we compare clonal evolution based on 12 measured time points to the estimated development, based on only 2 measured time points + interpolated time points + estimated therapy effect (Figure 3B).

For patient UPN02, also receiving treatment with lenalidomide, certain differences can be observed (Figure 3C). The magenta clone can barely be observed in the development estimated by clevRvis (measured with CCF = 22% at time point t_{53}). However, as the clone is present with CCF < 1% at both t_0 and t_{60}, it appears basically impossible to predict its unexpected rise and subsequent fall at an intervening time point. Despite this apparent difference, the estimated clonal evolution reflects the main characteristics of the true clonal development of this patient (results for all patients available in Additional file 1, Figures S11–S21).

Data set 2 contains information on 2 patients: patient 1 characterized by 15 and patient 2 characterized by 4 time points. Clonal evolution of patient 2, considering all available time points, is displayed in Figure 3D. González-Rincón et al. [12] report that the sample at t_{408} was taken before treatment. Subsequently, the patient received treatment with FCR (fludarabin, cyclophosphamid, rituximab) followed by maintenance therapy with rituximab. The patient was reported to achieve complete response. However, clonal evolution only based on the measured time points does not reflect this response. From the results published, we estimate that FCR was given for roughly one year. Therefore, we estimate therapy effect for t_{700}. The resulting plot in Figure 3E shows a considerable effect of therapy on clonal evolution, matching the described course of disease (results for both patients available in Additional file 1, Figures S22, S23).

Detecting bi-allelic events

clevRvis contains a novel plotting option for clonal evolution – plaice plots. These plots allow for identification of bi-allelic events. Considering data sets 1 to 4, we investigate applicability and added value of an analysis by plaice plots. Results considering 8 exemplary patients are summed up in Figure 4.

Patient UPN05 in data set 1 is characterized by branched dependent evolution, with a total of 7 clones. The first clone features, among others, a point mutation in *BCOR*. The gene is located on the X-chromosome. As the patient is male, the hemizygous variant leads to a loss of the only available copy of *BCOR*. In the plaice plot, the first and all subsequent clones are marked, indicating a missing healthy allele of *BCOR* (Figure 4A) (plaice plots for all patients in data set 1 available in Additional file 1, Figures S11–S21).

Patient 1 in data set 2 features a splicing variant in *TP53* (Figure 4B; light blue clone). Subsequently, the patient acquires a deletion in chromosome 17 (17p13.1 del), overlapping *TP53*. The CNV is clustered in clone 2 (intermediate blue). As this event leads to a loss of the only available allele of *TP53*, clone 2 is marked in the plaice plot. Light blue – the color of clone 1, characterized by the initial variant in *TP53* – is chosen for coloring. Additionally, clone 3 (dark blue) features deficient *TBC1D4* (13q14.3 del + point mutation) and *UBA1* (X-chromosomal variant in a male patient) (plaice plots for both patients in data set 2 available in Additional file 1, Figures S22, S23).

For patient UPN06 in data set 3, clonal evolution cannot be reconstructed uniquely (Figure 4C). The patient features two variants affecting *TP53*: a point mutation (p.Val272Met) and a derivative chromosome 17 (der(17)t(13;17)(q21;p12)). Data does not allow for deciphering, which of the two variants developed first. Branched dependent (version 1) as well as linear (version 2) evolution can be reconstructed. In addition to the difference in clonal evolution model, the effect on *TP53* differs considerably: in version 1, plaice plots show *TP53* deficiency in 22–25% of the cells. In version 2, on the contrary, all cells contain at least one healthy copy of *TP53* throughout the entire period of

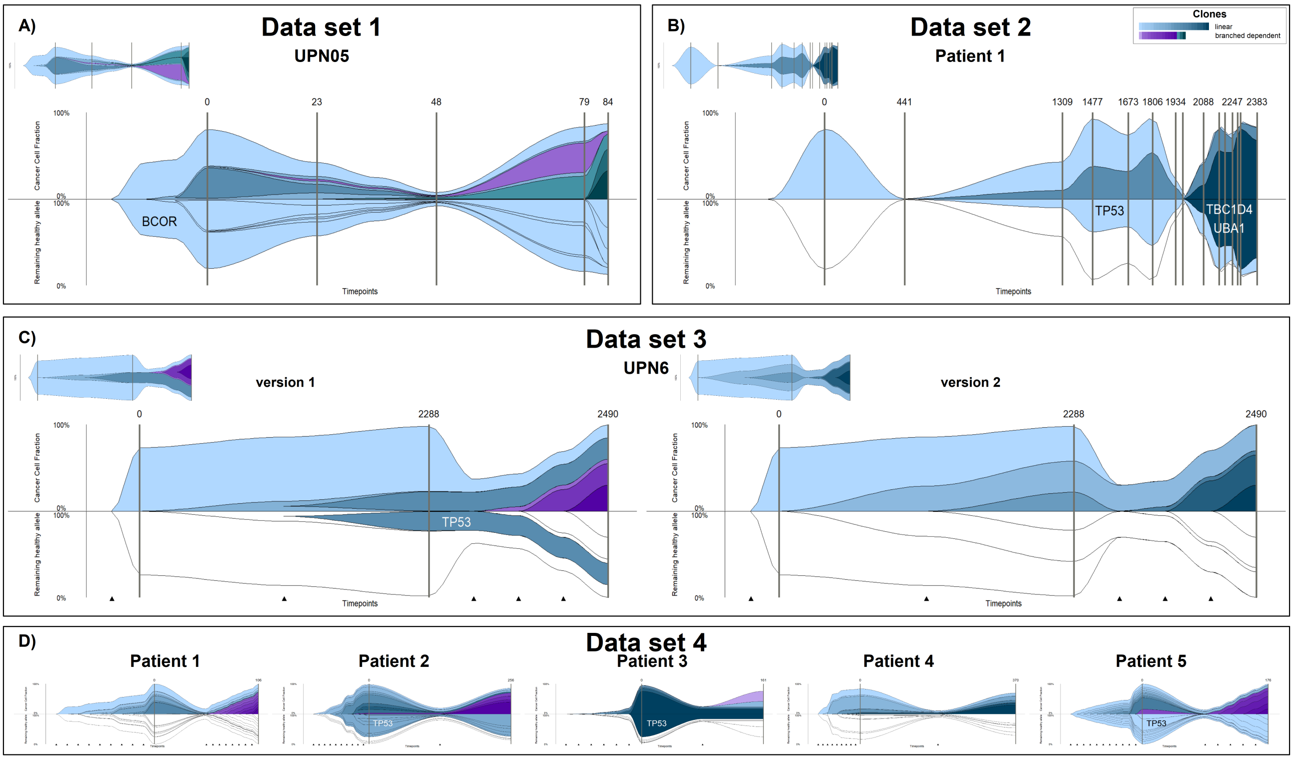

Figure 4. Visualization of clonal evolution using plaice plots vs dolphin plots. A) Data set 1, UPN05: A hemizygous variant affects *BCOR*. B) Data set 2, patient 1: A bi-allelic event causes *TP53* deficiency in clone 2. Clone 3 is characterized by additional *TBC1D4* and *UBA1* deficiency. C) Data set 3, UPN06 (enabled therapy effect estimation): A double-hit event affects *TP53* (der(17)t(13;17)(q21;p12) + point mutation). Clonal evolution cannot be reconstructed uniquely. Version 1 assumes branched dependent evolution, leading to *TP53* deficiency in one clone. Version 2 two assumes linear clonal evolution, leaving at least one healthy allele of *TP53* in each clone. D) Data set 4, patients 1 to 5: Different bi-allelic events lead to *TP53* deficiency in patients 2, 3 and 5. Note: for all plots, time point interpolation is enabled to improve visualization of newly developing clones prior to the first measured time point.

follow-up (plaice plots for all patients in data set 3 available in Additional file 1, Figures S24-S34).

Relapsing patients in data set 4 (patients 1 to 5) are – different from non-relapse patients – characterized by $\geq$ 2 variants affecting *TP53*. Analysis and visualization with plaice plots shows that patients 2, 3 and 5 are characterized by deficient *TP53* in a majority of cells (Figure 4D). The stem line of patient 1 is characterized by a point mutation in *TP53* (p.Arg248Gln) and an overlapping CNV. The duplication affects the mutated allele of *TP53*, however, a ratio of 1:2 for healthy:mutated remains. Thus, no clone is marked in the lower plaice plot. For patient 4, a point mutation in *TP53* (p.Arg248Gln) is detected in the second to last clone. A CNV below detection thresholds is assumed to be additionally present. However, data does not allow to decide on whether it is a deletion or duplication. Thus, it is unclear whether a healthy copy of *TP53* remains (plaice plots for all patients in data set 4 available in Additional file 1, Figures S35-S45).

Discussion

clevRvis is an R/Bioconductor package, providing innovative visualization techniques for clonal evolution. The optimized, highly customizable implementation of established visualization approaches (shark plots, dolphin plots) is complemented by a unique allele-aware representation of clonal evolution, allowing for analysis of bi-allelic events at a glance: plaice plots. In addition, the tool contains fully automatic algorithms for time point interpolation and therapy effect estimation, phylogeny-aware color-coding, exploring alternative trees as well as a graphical user interface for intuitive usage not just by computer scientists, but also biologists and physicians.

To our knowledge, only two alternative approaches for visualizing clonal evolution exist: fishplot [13] and timescape [14]. With respect to functionalities, our novel approach unites all options of currently available tools and provides a wide set of additional features. Analyzing four publically available data sets, it can be observed that plots generated with clevRvis allow for an improved visualization of clonal evolution, outperforming both fishplot and timescape. Furthermore, new insights into disease course can be gained and reasons explored for therapy failure and relapse.

As regards suitable input data, the analysis of clonal evolution faces a major challenge: commonly, the number of tumor samples available heavily depends on the tumor itself. For non-solid tumors, bone marrow or peripheral blood are commonly analyzed (e.g. [6], [8]). Taking into account a patient's burden, performing regular bone marrow biopsies is ethically difficult to justify, especially towards the end of a therapy that is expected to lead to remission. For solid tumors, e.g. brain tumors, collecting samples after therapy is practically impossible as long as no relapse is observed. Thus, valid approaches estimating the development of a tumor and its response to therapy are of high relevance.

The two algorithms for time point interpolation and therapy effect estimation, inspired by the general idea outlined by Reutter et al. [8], mark a central element of clevRvis. However, the assumptions, on which these algorithms are based, can be discussed.

Interpolating initial development of a tumor towards the first measured time point t_1, we focus on the difference in CCFs (ccf'). If ccf' of clone A (=stemline) is 20% at t_1, we assume that it is also 20% at every interpolated initial time point. It is, of course, possible that clone A temporarily reaches values > 20% and is – towards t_1 – pushed away by clone B, resulting in a

decrease of ccf'. On the contrary, it is also possible that clone A expands only slowly and values < 20% are observed prior to t_1. As we could not find evidence for one of the two scenarios being generally more likely, we decided – as a compromise – to focus only on ccf' at t_1, which may result in an underestimation in some cases and an overestimation in other cases.

In the absence of therapy, it appears sensible to assume that the overall tumor load never decreases. Interpolating development of a tumor between two measured time points with no therapy being applied, we therefore assume linear development of CCFs. While focusing on the difference in CCFs would be a valid alternative approach for clones showing an increase in ccf', it can lead to a violation of our main assumption in case of decreasing ccf' (new, quickly expanding clones pushing away existing clones). As the overall tumor load is not expected to decrease at any (interpolated) time point, we decided to stick to linear development for all clones. New clones are assumed to develop successively, based on their nested level.

In the presence of therapy, we assume that every observable decrease in CCF is related to therapy. Focusing again on the difference in CCFs, the minimum ccf' of the measured time points prior (t_i) and after (t_{i+1}) estimated therapy effect is considered. If a decrease in ccf' can be observed, we assume that it is caused by therapy. If an increase is observed, we assume that the clone is resistant to therapy and was able to expand after the end of therapy. Similar to interpolation of the initial development, it is possible that this approach overestimates therapy effect in some cases, while underestimating it in others. We consider our approach a compromise, approximating true development in a majority of cases.

Due to limited data being available, e.g. data set 4, we could not proof validity of our algorithms for all patients. However, evaluating two public data sets, we could show that our algorithms indeed provide valid solutions for interpolating time points and estimating therapy effect. Partly, they even revealed new insights into tumor development (data set 2, patient 2; Figure 3D vs 3E). While our analysis also showed that the extreme development of a clone, e.g. a considerable increase followed by subsequent decrease, in between two measured time points cannot be approximated, it remains questionable whether any algorithm would be capable of predicting this unexpected behaviour in the lack of sufficient data.

In addition to approximating a tumor's development, the analysis of clonal evolution on allele-level represents another central aspect of clevRvis. While bi-allelic events are considered of high relevance, their analysis commonly requires tedious manual inspection of the variants characterizing each clone. Considering CNVs, genetic expert knowledge is often required to decipher partly complex karyotypes. Additionally, it may be necessary to consider raw sequencing data. While specific clones featuring bi-allelic events could also be highlighted in a common fish plot, our newly developed plaice plots provide a unique option to 1) easily convey information on bi-allelic events and 2) link this information to characteristic clones by suitable color-coding. As shown in Figure 4B (data set 2, patient 1), this does not necessarily refer to the same clone. It may of course be argued that it is still necessary to manually define the clones to color in the lower plaice plot once. Subsequently, however, this information can be evaluated at a glance by physicians, biologists and even computer scientists.

Compared to dolphin plots, it may be considered a disadvantage that the actual clonal evolution is only displayed in the upper half of a plaice plot, making complex clonal evolution patterns potentially difficult to see. However, real examples of complex clonal evolution (e.g. data set 1, UPN10: 8 clones, dependent and independent branches, Additional file 1, Figure S20C vs S20D; data set 4, patient 5: 17 clones, linear development, Additional file 1, Figure S39D vs S39E) show that all clones can still be distinguished clearly. Simulated data, considering even more complex clonal evolution (100 clones and 100 time points; Additional file 1, section 2.5), show that all plot types implemented in clevRvis – including plaice plots – allow for visualization of a high number of clones as well as a high number of time points.

Conclusion

clevRvis provides an extensive set of visualization techniques for clonal evolution. Exceeding currently available approaches, clevRvis allows for approximating of a tumor's development in between measured time points as well as analyzing bi-allelic events. Our future work will include an extension of the clevRvis package, including approaches for considering multiple spatial locations per time point, as well as visualizing changes in gene expression and methlyation along common clonal evolution, based on a tumor's mutational profile.

Availability of source code and requirements

- Project name: clevRvis
- Project home page: https://github.com/sandmanns/clevRvis
- Bioconductor: https://bioconductor.org/packages/clevRvis
- Operating system(s): Platform independent
- Programming language: R
- Other requirements: none
- License: LGPL-3.0
- bio.tools ID: biotools:clevRvis
- SciCrunch ID: RRID:SCR_023154

Availability of supporting data and materials

Data and materials supporting the results of this article are available in Additional files 1-5.

Additional file 1

SupplementaryInformation.pdf: information on supplementary methods and results.

Additional file 2

Table_S1.tsv: input for the analysis of data set 1 with clevRvis.

Additional file 3

Table_S2.tsv: input for the analysis of data set 2 with clevRvis.

Additional file 4

Table_S3.tsv: input for the analysis of data set 3 with clevRvis.

Additional file 5

Table_S4.tsv: input for the analysis of data set 4 with clevRvis.

Declarations

List of abbreviations

aCGH: array-CGH; AML: acute myeloid leukemia; BL: Burkitt lymphoma; CCF: cancer cell fraction; CLL: chronic lymphatic leukemia; CNV: copy number variant; indels: insertions/deletions; FCR: fludarabin, cyclophosphamid, rituximab; FISH: fluorescence *in situ* hybridization; IPSS-R: Revised International Prognostic Scoring System; MDS: myelodysplastic syndromes; MM: multiple myeloma; MPN: myeloid neoplasia; NGS: next-generation sequencing; R-ISS: Revised International Staging System; SV: structural variant; tNGS: targeted next-generation sequencing; VAF: variant allele frequency; WES: whole-exome sequencing.

Ethical Approval

All patient material was collected and analyzed in accordance with the relevant ethical guidelines and regulations. Informed consent was obtained from all subjects.

Consent for publication

Not applicable

Competing Interests

The authors declare that they have no competing interests.

Funding

This research was supported by the Open Access Publication Fund of the University of Muenster. There was no additional external funding received. The funding body had no role in the design of the study, collection, analysis, and interpretation of data, decision to publish, or in writing the manuscript.

Author's Contributions

S.S. conceptualized the project, developed the algorithm, supervised and supported implementation of the algorithm, performed data analyses, and wrote the manuscript. C.I. implemented the algorithm. J.V. supervised the project and reviewed the manuscript. All authors read, revised, and approved the final version of the manuscript.

Acknowledgements

Not applicable

References

1. Palumbo A, Avet-Loiseau H, Oliva S, Lokhorst HM, Goldschmidt H, Rosinol L, et al. Revised International Staging System for Multiple Myeloma: A Report From International Myeloma Working Group. J Clin Oncol 2015;33:2863–2869.
3. Greenberg PL, Tuechler H, Schanz J, Sanz G, Garcia-Manero G, Solé F, et al. Revised international prognostic scoring system for myelodysplastic syndromes. Blood 2012;120:2454–2465.
2. Rajkumar SV. Multiple Myeloma: 2020 Update on Diagnosis, Risk-Stratification and Management. Am J Hematol 2020;95:548–567.
4. Malcovati L, Crouch S, de Graaf AO, Sandmann S, Tobiasson M, Kosmider O, et al. Mutation Profiles Identify Distinct Clusters of Lower Risk Myelodysplastic Syndromes with Unique Clinical and Biological Features and Clinical Endpoints. Blood 136(Supplement 1) 2020;p. 2454–2465.
5. Nowell PC. The Clonal Evolution of Tumor Cell Populations. Science 1976;194:23–28.
6. da Silva-Coelho P, Kroeze LI, Yoshida K, Koorenhof-Scheele TN, Knops R, van de Locht LT, et al. Clonal evolution in myelodysplastic syndromes. Nat Commun 2017;8:15099.
7. Sandmann S, Behrens YL, Davenport C, Thol F, Heuser M, Dörfel D, et al. Clonal Evolution at First Sight: A Combined Visualization of Diverse Diagnostic Methods Improves Understanding of Leukemic Progression. Front Oncol 2022;12:888114.
8. Reutter K, Sandmann S, Rohde J, Müller S, Wöste M, Khanam T, et al. Reconstructing clonal evolution in relapsed and non-relapsed Burkitt lymphoma. Leukemia 2021;35:639–643.
9. Davies A, Gao R, Navin N. Tumor evolution: Linear, branching, neutral or punctuated? Biochim Biophys Acta Rev Cancer 2017;1867:151–161.
10. Sandmann S, Karsch K, Bartel P, Exeler R, Brix TJ, Mai EK, et al. The Role of Clonal Evolution on Progression, Blood Parameters, and Response to Therapy in Multiple Myeloma. Front Oncol 2022;12:919278.
11. Krzywinski M. Visualizing Clonal Evolution in Cancer. Mol Cell 2016;62:652–656.
12. González-Rincón J, Gómez S, Martinez N, Troulé K, Perales-Patón J, Derdak S, et al. Clonal dynamics monitoring during clinical evolution in chronic lymphocytic leukaemia. Sci Rep 2019;9:975.
13. Miller CA, McMichael J, Dang HX, Maher CA, Ding L, Ley TJ, et al. Visualizing tumor evolution with the fishplot package for R. BMC Genomics 2016;17:880.
14. Smith M. timescape: Patient Clonal Timescapes. R package version 1.20.0; 2022.
15. Deshwar AG, Vembu S, Yung CK, Jang GH, Stein L, Morris Q. PhyloWGS: reconstructing subclonal composition and evolution from whole-genome sequencing of tumors. Genome Biol 2015;16:35.
16. Jiang Y, Qiu Y, Minn AJ, Zhang NR. Assessing intratumor heterogeneity and tracking longitudinal and spatial clonal evolutionary history by next-generation sequencing. Proc Natl Acad Sci U S A 2016;113:E5528–E5537.
17. Strino F, Parisi F, Micsinai M, Kluger Y. TrAp: a tree approach for fingerprinting subclonal tumor composition. Nucleic Acids Res 2013;41:e165.
18. Zhu W, Kuziora M, Creasy T, Lai Z, Morehouse C, Guo X, et al. BubbleTree: an intuitive visualization to elucidate tumoral aneuploidy and clonality using next generation sequencing data. Nucleic Acids Res 2016;44:e38.
19. Bao L, Pu M, Messer K. AbsCN-seq: a statistical method to estimate tumor purity, ploidy and absolute copy numbers from next-generation sequencing data. Bioinformatics 2014;30:1056–1063.
20. Smith M. mapscape: mapscape. R package version 1.20.0; 2022.

Click here to access/download
Supplementary Material
Additional File 1.pdf
