# Supplementary material for: clevRvis: visualization techniques for clonal evolution
Source: Gigascience. 2023 Apr 11;12:giad020. doi: 10.1093/gigascience/giad020 (PMC10087014; doi:10.1093/gigascience/giad020)
Supplement: giad020_GIGA-D-22-00248_Revision_2 [file giad020_giga-d-22-00248_revision_2.pdf]

GigaScience
clevRvis: Visualization Techniques for Clonal Evolution
--Manuscript Draft--

**Manuscript Number:**	GIGA-D-22-00248R2
**Full Title:**	clevRvis: Visualization Techniques for Clonal Evolution
**Article Type:**	Technical Note
**Funding Information:**	Open Access Publication Fund / Not applicable
**Abstract:**	Background: A thorough analysis of clonal evolution commonly requires integration of diverse sources of data, e.g. karyotyping, next-generation sequencing and clinical information. Subsequent to actual reconstruction of clonal evolution, detailed analysis and interpretation of the results is essential. Often, however, only few tumor samples per patient are available. Thus, information on clonal development and therapy effect may be incomplete. Furthermore, analysis of bi-allelic events - considered of high relevance with respect to disease course - can commonly only be realized by time-consuming analysis of the raw results and even raw sequencing data. Results: We developed clevRvis, an R/Bioconductor package providing an extensive set of visualization techniques for clonal evolution. In addition to common approaches for visualization, clevRvis offers a unique option for allele-aware representation: plaice plots. Bi-allelic events may be visualized and inspected at a glance. Analyzing four public data sets, we show that plaice plots help to gain new insights into tumor development and investigate hypotheses on disease progression and therapy resistance. In addition to a graphical user interface, automatic phylogeny-aware color coding of the plots and an approach to explore alternative trees, clevRvis provides two algorithms for fully automatic time point interpolation and therapy effect estimation. Analyzing two public data sets, we show that both approaches allow for valid approximation of a tumor's development in between measured time points. Conclusions: clevRvis represents a novel option for user-friendly analysis of clonal evolution, contributing to gaining new insights into tumor development.
**Corresponding Author:**	Sarah Sandmann<br>Westfälische Wilhelms-Universität Münster<br>Münster, GERMANY
**Corresponding Author Secondary Information:**	
**Corresponding Author's Institution:**	Westfälische Wilhelms-Universität Münster
**Corresponding Author's Secondary Institution:**	
**First Author:**	Sarah Sandmann
**First Author Secondary Information:**	
**Order of Authors:**	Sarah Sandmann
	Clara Inserte
	Julian Varghese
**Order of Authors Secondary Information:**	
**Response to Reviewers:**	We added our ORCID iDs to the title page. The data availability section was modified as suggested, now citing our archived code and data available at GigaDB.<br><br>As pdf building from our tex-file went wrong in the EM, we uploaded the manuscript as pdf and added all files (incl figures, bibliography and style files) to re-build our manuscript as "supplemental material".
**Additional Information:**	
**Question**	**Response**

Powered by Editorial Manager® and ProduXion Manager® from Aries Systems Corporation

Are you submitting this manuscript to a special series or article collection?	No
**Experimental design and statistics**<br><br>Full details of the experimental design and statistical methods used should be given in the Methods section, as detailed in our Minimum Standards Reporting Checklist. Information essential to interpreting the data presented should be made available in the figure legends.<br><br>Have you included all the information requested in your manuscript?	No
If not, please give reasons for any omissions below.<br><br>as follow-up to "**Experimental design and statistics**<br><br>Full details of the experimental design and statistical methods used should be given in the Methods section, as detailed in our Minimum Standards Reporting Checklist. Information essential to interpreting the data presented should be made available in the figure legends.<br><br>Have you included all the information requested in your manuscript?<br><br>"	Our methods section contains a detailed description of all analyses we performed and all necessary information to reproduce our analyses. However, as we only analyzed public data sets, no information on e.g. power calculation or how samples were collected is included.
**Resources**<br><br>A description of all resources used, including antibodies, cell lines, animals and software tools, with enough information to allow them to be uniquely identified, should be included in the Methods section. Authors are strongly encouraged to cite Research Resource Identifiers (RRIDs) for antibodies, model organisms and tools, where possible.	Yes

Powered by Editorial Manager® and ProduXion Manager® from Aries Systems Corporation

Have you included the information requested as detailed in our Minimum Standards Reporting Checklist?	
**Availability of data and materials**<br><br>All datasets and code on which the conclusions of the paper rely must be either included in your submission or deposited in publicly available repositories (where available and ethically appropriate), referencing such data using a unique identifier in the references and in the "Availability of Data and Materials" section of your manuscript.<br><br>Have you have met the above requirement as detailed in our Minimum Standards Reporting Checklist?	Yes

Powered by Editorial Manager® and ProduXion Manager® from Aries Systems Corporation

Manuscript - corresponds to Latex Main file Click here to access/download;Manuscript;main.pdf

OXFORD (GIGA)^n SCIENCE

GigaScience, 2017, 1–11

doi: xx.xxxx/xxxx
Manuscript in Preparation
Technical Note

TECHNICAL NOTE

clevRvis: Visualization Techniques for Clonal Evolution

Sarah Sandmann[1,*], Clara Inserte[1] and Julian Varghese[1]

[1]Institute of Medical Informatics, University of Münster, Münster, Germany

*sarah.sandmann@uni-muenster.de
ORCID iD: Sarah Sandmann (0000-0002-5011-0641), Clara Inserte (0000-0001-7371-8839), Julian Varghese (0000-0002-7206-3719)

Abstract

Background: A thorough analysis of clonal evolution commonly requires integration of diverse sources of data, e.g. karyotyping, next-generation sequencing and clinical information. Subsequent to actual reconstruction of clonal evolution, detailed analysis and interpretation of the results is essential. Often, however, only few tumor samples per patient are available. Thus, information on clonal development and therapy effect may be incomplete. Furthermore, analysis of bi-allelic events – considered of high relevance with respect to disease course – can commonly only be realized by time-consuming analysis of the raw results and even raw sequencing data. **Results:** We developed clevRvis, an R/Bioconductor package providing an extensive set of visualization techniques for clonal evolution. In addition to common approaches for visualization, clevRvis offers a unique option for allele-aware representation: plaice plots. Bi-allelic events may be visualized and inspected at a glance. Analyzing four public data sets, we show that plaice plots help to gain new insights into tumor development and investigate hypotheses on disease progression and therapy resistance. In addition to a graphical user interface, automatic phylogeny-aware color coding of the plots and an approach to explore alternative trees, clevRvis provides two algorithms for fully automatic time point interpolation and therapy effect estimation. Analyzing two public data sets, we show that both approaches allow for valid approximation of a tumor's development in between measured time points. **Conclusions:** clevRvis represents a novel option for user-friendly analysis of clonal evolution, contributing to gaining new insights into tumor development.

Key words: Clonal evolution; tumor development; visualization; cancer cell fraction; bi-allelic events; therapy effect

Background

For many types of cancer, determining their mutational profile is a crucial step, having an impact on diagnosis as well as treatment. In multiple myeloma (MM), for example, cytogenetic aberrations have been found to have significant impact on prognosis and are thus considered in the Revised International Staging System (R-ISS) for risk stratification [1, 2]. Similarly, in myelodysplastic syndromes (MDS), patients are commonly stratified according to the Revised International Prognostic Scoring System (IPSS-R), evaluating – among others – cytogenetic abnormalities [3]. Recently, it has been shown that the analysis of point mutations and small insertions/deletions (indels) even allows for identification of clinically relevant subgroups within low-risk MDS patients [4].

In addition to information on the bare presence or absence of variants, their development over time, the clonal evolution [5], is of major importance in several diseases, e.g. MDS, acute myeloid leukemia (AML) or Burkitt lymphoma (BL) [6, 7, 8]. For a thorough analysis of clonal evolution, all variants ranging from point mutations to aberrations affecting whole chromosomes should be taken into account. Commonly, various sources of data are considered to allow for valid detection of these variants, e.g. karyotyping, fluorescence *in sito* hybridization, microarrays like SNP-arrays or array-CGH (aCGH), next-generation sequencing (NGS) and Sanger sequencing. Integrating all of these data, clonal evolution may be reconstructed [7].

Studying clonal evolution in more detail, a diverse set of analyses can be performed: The model of clonal evolution (linear vs branched dependent vs branched independent; of note, neutral and punctuated evolution will be considered special cases of branched and linear evolution) [8, 9] can be determined, correlation to blood parameters, therapy resistance and disease progression investigated [10] and patterns characterizing subgroups of patients explored. For example, by detailed analysis of clonal evolution, evidence was found that relapsing BL is associated with the presence of clones, featuring double-hit events in *TP53* [8].

Compiled on: March 7, 2023.
Draft manuscript prepared by the author.

Table 1. Overview of the data sets analyzed with clevRvis. BL – Burkitt lymphoma; CLL – chronic lymphatic leukemia; FISH – fluorescence *in situ* hybridization; MDS – myelodysplastic syndromes; MPN – myeloid neoplasia; tNGS – targeted next-generation sequencing; WES – whole-exome sequencing.

Data set	Disease	Patients	#Time points (median [min-max])	#Clones (median [min-max])	Clonal evolution models	Data types
1	MDS	11	5 [5-30]	5 [1-9]	linear, branched dependent, branched independent	karyotyping, FISH, SNP-array, WES, tNGS
2	CLL	2	9.5 [4-15]	4 [3-5]	linear, branched dependent	karyotyping, FISH, WES, tNGS
3	MPN	8	2 [1-5]	5.5 [2-11]	linear, branched dependent, branched independent	karyotyping, FISH, aCGH, tNGS
4	BL	10	1.5 [1-2]	9 [7-17]	linear, branched dependent	FISH, SNP-array WES, tNGS, Sanger

However, these analyses may be hampered by different aspects: 1) Variants are only detected at few time points, providing an incomplete representation of the disease course. 2) No information on a therapy's effect on clonal evolution is available. 3) Evaluation of bi-allelic events requires tedious manual work, analyzing raw sequencing data and detailed variant calling information.

To overcome these obstacles, we developed clevRvis – an R/Bioconductor package for clonal evolution in R, providing innovative visualization techniques. In addition to common R functions, clevRvis offers a web-based graphical user interface, allowing usage not just by computer scientists, but also physicians and biologists. Our approach contains fully automatic algorithms for interpolating additional time points as well as estimating therapy effect. Evaluating two real, publically available data sets from different disease entities, characterized by a high number of measured time points, we show that both estimation approaches generate valid results.

clevRvis generates three different types of plots: 1) shark plots (a graph-based representation of clonal evolution), 2) dolphin plots (a fish plot-like representation, optionally also considering interpolated time points and estimated therapy effect), 3) plaice plots (a novel type of plots allowing for detection of bi-allelic events at a glance). All plots generated by clevRvis are highly customizable. Following recommendations for graphical strategies in clonal evolution [11], we implemented an algorithm for phylogeny-aware color coding of the clones to obtain optimal visualization. In addition, alternative phylogenetic trees can be determined and explored interactively. By analysis of four public data sets, we show that visualization with clevRvis outperforms common alternative approaches. Additionally, we show the added value of plaice plots.

Methods

Data sets analyzed

We analyze four real data sets, containing detailed information on the clonal evolution of $n = 31$ patients. Table 1 provides an overview of the data sets and their main characteristics.

The first set covers data from 11 patients with MDS. Clonal evolution was reconstructed based on karyotyping, FISH, SNP-array, whole-exome sequencing (WES) and ultra-deep targeted next-generation sequencing (tNGS) data [6]. The data set is characterized by a high number of time points (up to 30) and a relatively low number of clones. All major models of clonal evolution are present. Six patients received supportive care only, while five additionally received lenalidomide, which is expected to impact clonal evolution. The data set serves a test case, exploring options for visualizing clonal evolution and validating our approaches for automatic time point interpolation and therapy effect estimation (information on how input data for the analysis with clevRvis was derived from the publication by da Silva-Coelho et al. [6] is available in Additional file 1, section 1.1).

The second set covers data from 2 patients with chronic lymphatic leukemia (CLL). Clonal evolution was reconstructed based on FISH, WES and tNGS data [12]. The data set provides detailed information on follow-up – especially on therapies applied – for up to 12 years, as well as regular variant detection throughout the whole time of follow-up. Thereby, this data set serves a second test case for the analysis with clevRvis, considering time point interpolation and therapy effect estimation in a different disease entity and a different source of data (information on how input data for the analysis with clevRvis was derived from the publication by González-Rincón et al. [12] is available in Additional file 1, section 1.2).

The third set covers data from 8 patients with myeloid neoplasia (MPN). Clonal evolution was reconstructed based on karyotyping, FISH, aCGH and tNGS data [7]. Presence of 1 to 5 time points and an increased number of clones (one case of branched dependent evolution can be specified more precisely as neutral evolution [9]), pose a challenge for visualization. Partly, no samples were collected towards the end of therapy, requiring interpolation of additional time points and estimating the effect of therapy (information on how input data for the analysis with clevRvis was derived from the publication by Sandmann et al. [7] is available in Additional file 1, section 1.3).

The fourth set covers data from 10 patients with BL. Clonal evolution was reconstructed based on FISH, SNP-array, WES, tNGS and Sanger sequencing data [8]. The data set is characterized by a low number of time points (1 for non-relapsing patients, 2 for relapsing patients). While therapy is known to have been applied, no data showing its effect on clonal evolution is available. Thus, circumstances require interpolation of time points and estimation of therapy effect for all patients. Additionally, samples feature a high number of clones (up to 17), which is expected to be a major challenge for visualization (information on how input data for the analysis with clevRvis was derived from the publication by Reutter et al. [8] is available in Additional file 1, section 1.4).

All data sets contain information on detected small variants (SNVs and indels) as well as large variants (structural variants SVs and copy number variants CNVs) for every patient. Analysis of clonal evolution always involves integration of various data sources and partly overlapping variants. Thereby, the detection of bi-allelic variants and their evaluation by plaice plots is key for all data sets.

clevRvis

clevRvis provides an extensive set of visualization techniques for clonal evolution. An overview of the analysis pipeline is provided in Figure 1 (screenshots of the software are available in Additional file 1, Figures S1-S8).

For the subsequent description of the analyses performed by clevRvis, we use the following definitions: $ccf(c, t_i)$ is defined as the cancer cell fraction (CCF) of clone c, with $c \in \{1, ..., n\}$, at time point t_i, with $t_i \in \{t_1, ..., t_m\}$. $parent(c)$ is defined as the parent-clone of clone c, $children(c)$ as the children-clone(s) of clone c. For a clone c developing from normal cells, $parent(c) = 0$. All clones

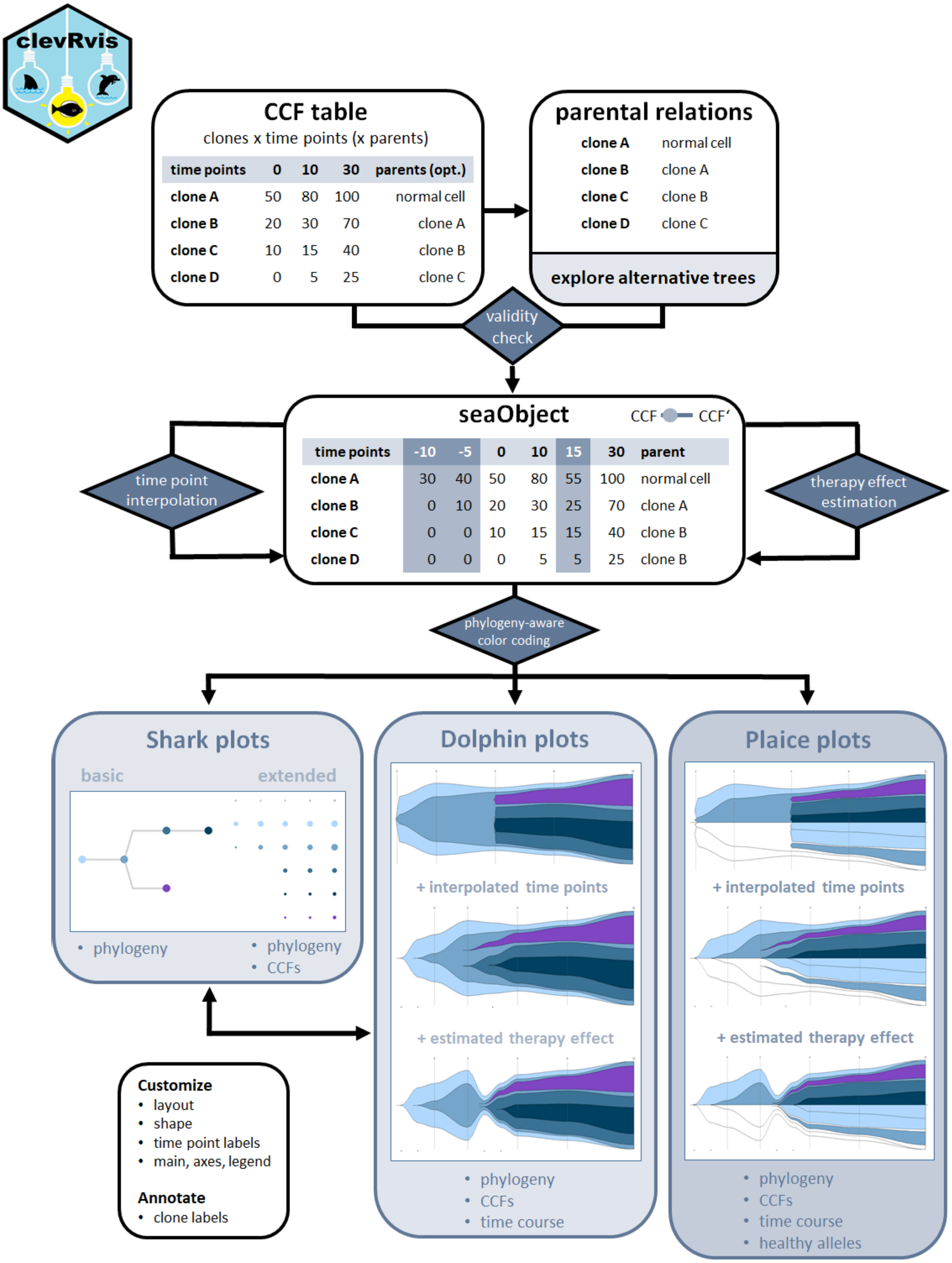

Figure 1. Overview of the analyses performed by clevRvis. Based on a CCF table and information on parental relations, a validity check is performed. For the resulting seaObject, additional time points may be interpolated and therapy effect may be estimated. Using phylogeny-aware color coding, shark plots, dolphin plots and plaice plots can be generated.

developing from normal cells are defined by $children(0)$. $ccf'(c, t_i)$ is defined as the difference in CCFs of a clone c at time point t_i. Thus, $ccf'(c, t_i) = ccf(c, t_i) - \sum_{j \in children(c)} ccf(j, t_i)$.

Validity check

clevRvis requires the upload of a *CCF table*, containing information on the CCF of every clone at every time point, optionally also including information on parental relations. In an interactive dialogue, the user can subsequently – if this information has not been uploaded – define the parental relations for every clone listed in the CCF table. Normal cells as well as clones different from the considered one may be selected. Upon submitting this information to generate the initial *seaObject*, a validity check is performed, adapting and extending the check performed by fishplot [13] (see Algorithm 1).

Algorithm 1 validity check

```
for each t_i in {t_1, ..., t_m} do
    for each c in {1, ..., n} do
        if ccf(c, t_i) < Σ_{j∈children(c)} ccf(j, t_i) then
            return check.failed
        if Σ_{j∈children(0)} ccf(j, t_i) > 100 then
            return check.failed
        if ccf(c, t_i) > 0 and ccf(c, t_{i+1}) = 0 and ccf(c, t_{i+2}) > 0 then
            return check.failed
        if ccf'(c, t_i) > 0 and ccf'(c, t_{i+1}) = 0 and ccf'(c, t_{i+2}) > 0 then
            return check.failed
return check.passed
```

Summing up, 1) children-clones cannot exceed their parents, 2) clones developing from normal cells cannot add up to more than 100%, 3) a clone cannot reappear, 4) a parent-clone being thoroughly replaced by its children-clone(s) cannot reappear.

Exploring alternative trees

Alternative parental relations, resulting in alternative clonal evolution trees, can be explored interactively using clevRvis. Considering n clones, a parental relations vector of length n has to be defined. For every clone, 1 out of n options can be chosen ($n - 1$ different clones + normal cells), which results in n^n permutations. To optimize run-time, filtration of clearly invalid options is performed prior to clevRvis' thorough validity check (see Algorithm 2).

Algorithm 2 exploring alternative trees

```
for each c in {1, ..., n} do
    for each t_i in {t_1, ..., t_m} do
        if ccf(c, t_i) < any other ccf(j, t_i) then
            return c! = parent(j)
for each remaining c in {1, ..., n} do
    for each remaining t_i in {t_1, ..., t_m} do
        if parent(j) can only be c then
            if ccf(c, t_i) − ccf(j, t_i) < any other ccf(k, t_i) then
                return c! = parent(k)
for each remaining.parental.relations
    apply Algorithm 1 validity check
return valid.parental.relations
```

As a first filtration step, possible linear relations are investigated: If a clone c has, at any time point, a lower CCF compared to clone j at the same time point, c cannot be the parent of j. Subsequently, possible branched relations are taken into account: If a clone j clearly develops from clone c, an additional clone k can – as a branch – only develop from c if $ccf(c, t_i) - ccf(j, t_i) \geq ccf(k, t_i)$ for every time point t_i. On the basis of the remaining filtered options, permutations are determined and a thorough validity check (see Algorithm 1) is performed (maximum: 20,000 permutations). All valid parental relations are reported and alternative trees can be explored subsequently.

Time point interpolation

The initially generated seaObject may be extended by additional interpolated time points. The general idea of estimating auxiliary time points to improve visualization of clonal evolution was first outlined by Reutter et al. [8]. However, this initial approach mainly focused on improving figures generated by fishplot [13], considering only the difference in CCFs at a later measured time point. Furthermore, it did not contain any algorithm for automatic time point interpolation. Evaluating different scenarios of clonal evolution, we implemented an improved, fully automatic approach in clevRvis (see Algorithm 3).

Algorithm 3 time point interpolation

```
if interpolate initial development then
    initial.clones ← all clones c where ccf(c, t_1) ≠ 0
    nested.levels ← nested levels for all initial.clones
    new.time.points ← maximum(nested.levels)
    for each new.time.point in {1, ..., new.time.points} do
    // 1 refers to time point closest to t_1
        for each initial clone c with maximum(nested.levels) do
            ccf(c, new.time.point) ← ccf'(c, new.time.point) ← 0
        determine ccf(remaining.initial.clones, new.time.point) based
        on ccf'(initial.clones, new.time.point)
        remove maximum(nested.levels)

if interpolate development between measured time points then
    old.clones ← all clones c where ccf(c, t_i) ≠ 0
    new.clones ← all clones c where ccf(c, t_i) = 0 and ccf(c, t_{i+1}) ≠ 0
    new.unique.nested.levels ← unique nested levels for new.clones
    new.time.points ← new.unique.nested.levels − 1
        for each clone c in old.clones do
            ccf(c, new.time.points) ← linear development from
                                      ccf(c, t_i) to ccf(c, t_{i+1})
        for each clone c in new.clones do
            z ← number of new.unique.nested.levels < nested.level(c)
            for each new.time.point in {1, ..., z} do
                ccf(c, new.time.point) ← 0
            ccf(c, remaining.new.time.points) ← linear development
                                      from ccf(c, z) to ccf(c, t_{i+1})

return updated ccf
```

The number of time points interpolated by clevRvis depends on the clonal evolution being analyzed and cannot be defined by a user. We differentiate between interpolating development of a tumor prior to the first measured time point, and interpolating development between two measured time points. By default, all interpolated time points are evenly distributed (a detailed description on how to implement skewed events is available in Additional file 1, section 1.6).

We assume that clones of the same nested level developed at approximately the same time. A higher nested level indicates development at a later time. For example, in a linear evolution with clone B developing from clone A, it is sensible to assume that clone A developed first and expanded. Over time, a cell of clone A acquired additional mutations, finally resulting in the formation of clone B.

Interpolating time points prior to t_1, we focus on the difference in CCFs $ccf'(c, t_1)$ for all clones present at t_1 (*initial.clones*). The number of interpolated time points is defined by the maximum nested level of the initially present clones. As the clone(s) with the

highest nested level are assumed to have developed last, ccf' as well as ccf are set to zero for the first (and all subsequent) interpolated time points. The CCFs of the remaining initial clones are updated. The procedure is repeated with the second highest nested level etc. until only clones with nested level 0 remain.

Interpolating time points between t_i and t_{i+1}, we assume linear development of CCF for all clones already present at t_i (*old.clones*). For newly developing clones (*new.clones*), we determine their nested levels. The number of unique nested levels (*new.unique.nested.levels*) - 1 defines the number of interpolated time points. We stick to our assumption that clones with a higher nested level developed at a later time point. Thus, newly developing clone(s) with the lowest nested level are considered first. Linear development between t_i and t_{i+1} is assumed. For all clones with the second lowest nested level, CCF at the first interpolated time point is set to zero. Linear development is assumed for the remaining interpolated time points up to t_{i+1}. The procedure is repeated with the third lowest nested level etc. until only clones with the highest *new.unique.nested.levels* remain.

Of note, to generate smoother dolphin- and plaice plots in R, for all clones 0.1 is added to the (interpolated) time point prior to their first appearance.

Therapy effect estimation

If a therapy is applied, interpolating time points is assumed to be insufficient to approach the development of CCFs over time properly. In the absence of therapy, the overall tumor load is not expected to decrease. Any decrease in CCF differences over time is assumed to be caused by the expansion of superior children-clones. In the presence of therapy, however, we assume that any observed decrease in CCF differences is due to therapy. This general idea was first outlined by Reutter et al. [8] as well. In clevRvis, we implemented an updated, fully automatic approach for therapy effect estimation (see Algorithm 4).

Algorithm 4 therapy effect estimation

```
old.clones ← all clones c where ccf(c, t_i) ≠ 0
new.clones ← all clones c where ccf(c, t_i) = 0 and ccf(c, t_i+1) ≠ 0
for each clone c in old.clones do
    ccf'(c, therapy.time.point) ← minimum(ccf'(c, t_i), ccf'(c, t_i+1))
determine ccf(old.clones, therapy.time.point) based on
ccf'(old.clones, therapy.time.point)
ccf(new.clones, therapy.time.point) ← 0

if additional time point interpolation then
    therapy.clones ← all clones c where ccf(c, therapy.time.point) ≠ 0
    final.clones ← all clones c where ccf(c, t_i+1) ≠ 0
    new.final.clones ← all clones c where ccf(c, t_i+1) ≠ 0 and
                                          ccf(c, therapy.time.point) = 0
    nested.levels ← nested level for all new.final.clones
    recalculate nested.levels ignoring therapy.clones
    new.time.points ← maximum(recalculated.nested.levels)
    for each clone c in new.final.clones do
      z ← recalculated.nested.level(c)
      for each new.time.point in {1, ..., z} do
      // 1 refers to time point closest to therapy.time.point
        ccf(c, new.time.point) ← ccf'(c, new.time.point) ← 0
      if c has no children then
        ccf(c, remaining.new.time.points) ← linear development
                                    from ccf(c, z) to ccf(c, t_i+1)
      if c has children then
        ccf'(c, remaining.new.time.points) ← ccf'(c, t_i+1)
    determine ccf(remaining.final.clones, new.time.points) based
    on ccf'(final.clones, new.time.points)
return updated ccf
```

Estimating therapy effect, a single time point is added, corresponding to the clonal composition towards the end of therapy. The precise time point is user-definable (default: evenly distributed). In case new clones developed, enabling additional time point interpolation is recommended.

To estimate the effect of a therapy that has been applied between t_i and t_{i+1}, we focus on the difference in CCFs $ccf'(c, t_i)$ for all clones present at t_i (*old.clones*). Assuming that no increase in tumor load is observed during therapy, the minimum of $ccf'(c, t_i)$ and $ccf'(c, t_{i+1})$ is determined. For all newly developing clones (*new.clones*), we assume that they developed after the application of therapy. Thus, CCFs are set to zero for the estimated time point (*therapy.time.point*).

Interpolating additional time points – located between *therapy.time.point* and t_{i+1} –, we focus on newly developing clones. For all clones only present at t_{i+1} (*new.final.clones*), we recalculated the nested levels, ignoring clones already present at *therapy.time.point*. As an example, we consider three clones A, B and C that develop linearly. While A and B are already present at t_i, clone C only emerges at t_{i+1}. Another clone D develops from normal cells and – just like clone C – only emerges at t_{i+1}. Nested levels of clones C and D are two and zero respectively. However, development of both clones is just 'one step' compared to the starting position at t_i. Therefore, the recalculated nested levels, ignoring clones A and B, are zero for both C and D.

Sticking to our main assumption that clones with a higher (recalculated) nested level develop at a later time, additional time points are interpolated. The number of time points added is defined by the maximum recalculated nested level. Newly developing clone(s) with the lowest recalculated nested level are considered first. If a clone c has no children, linear development between *therapy.time.point* and t_{i+1} is assumed. Otherwise, we assume that the CCF for clone c at all interpolated time points is defined by $ccf'(c, t_{i+1})$. For all clones with the second lowest recalculated nested level, CCF at the first interpolated time point is set to zero. Subsequently, CCFs for the remaining time points are interpolated considering either linear development or $ccf'(c, t_{i+1})$. The procedure is repeated with the third lowest nested level etc. until only clones with the highest *recalculated.nested.level* remain.

Phylogeny-aware color coding

Based on an evaluation of graphical strategies for visualizing clonal evolution published by Krzywinski [11], we developed an approach for automatic phylogeny-aware color coding. Our approach sticks to the following rules:

i. The higher the nested level of a clone, the darker the hue.
ii. Clones of the same branch are colored by a similar hue.
iii. Clones on two (or more) branches with a common ancestor (branched dependent evolution) are colored by similar, but diverging colors.
iv. Clones on two (or more) branches without a common ancestor (branched independent evolution) are colored by colors of maximum difference.

The exact range of the color palette is dynamically determined based on the clonal evolution provided as input data (number of clones, maximum nested level). Thereby, options for differentiating between closely related clones are optimized. clevRvis supports a maximum of 25 independent clones, developing from normal cells, and an unlimited number of related clones.

Shark plots

Shark plots serve a basic, raw visualization of clonal evolution (see Figure 1). Using a classical graph approach, clones are represented by nodes, parental relations by edges. Thus, the phylogeny can be directly deduced from shark plots.

Optionally, shark plots can be extended to provide information on CCFs as well. Clones are additionally visualized next to the actual shark plot. The size of each clone is correlated with its CCF. Time points are plotted next to each other.

If shark plots are chosen to be plotted along with dolphin plots, both plots are connected interactively. By hovering over one of the clones, it is automatically highlighted in both, shark and dolphin plot.

Dolphin plots

An advanced visualization of clonal evolution is realized by dolphin plots, mainly corresponding to well-established fish plots. The development of each clone over time is displayed on the x-axis, the CCFs on the y-axis. Thereby, information on phylogeny, CCFs and time course characterizing a clonal evolution are jointly visualized in a single plot. Several basic options for customizing dolphin plots are available, e.g. switching between spline and polygon shape or separating independent clones. Additionally, a user may choose between standard centered visualization of clonal evolution and bottom layout. This causes the clones to develop as "lying" on the x-axis (similar to visualization of clonal evolution by timescape [14]). Automatically, the longest branch is chosen to be plotted on the bottom, while the remaining branches are added on top.

If a seaObject has been extended by additional interpolated time points and/or estimated therapy effect, both can be visualized by dolphin plots. Customizable labels can be added to distinguish between measured vs estimated time points.

Plaice plots

Plaice plots represent a derivative of dolphin plots, resp. fish plots, developed to improve visualization of bi-allelic events. Instead of one, we consider two "flatfish" (=plaice) that are mirrored above and below the y-axis.

Common clonal evolution is visualized only in the upper plot in bottom layout. Similar to dolphin plots, a user may choose between spline vs polygon shape and separating independent clones (recommended). The fraction of remaining healthy alleles is visualized in the lower plot. For this purpose, a mirrored presentation of clonal evolution in bottom layout is plotted. By default, clones in this part of the plot are not colored, representing a starting position of 100% healthy alleles.

As an example, we consider linear clonal evolution of 2 clones – A and B. Clone A is characterized by a mutation in *TP53*, clone B by a deletion 17p. The two variants are overlapping. If they affect different alleles, no healthy allele of *TP53* remains in all cells belonging to clone B. Thus, a user may chose to color clone B in the lower plot to indicate a decrease in healthy alleles of *TP53* as the CCF of clone B increases. The clone should, however, be colored in the hue of clone A. Thereby, the bi-allelic event leading to *TP53* deficiency is linked to the clone that is originally characterized by a mutation in this gene. Instead, if both variants affect the same allele, one healthy copy of *TP53* remains – independent of the CCF of clones A and B. Thus, no clone should be colored in the lower plot. In addition to bi-allelic events, variants affecting the only available X- or Y-chromosome in male subjects can equally be visualized using plaice plots. Detailed information on the recommend color coding of plaice plots is provided in Additional file 1, section 1.7.

Just like dolphin plots, plaice plots provide all options for visualizing data on additional interpolated time points (recommended) and estimated therapy effect.

Comparison to common approaches

Several approaches exist for visualizing clonal evolution. A majority of tools performing clonal evolution tree reconstruction, e.g. PhyloWGS [15], Canopy [16] or TRaP [17], provide a basic graph-based visualization that is automatically generated when performing the analysis. These plots, however, are commonly not customizable and will, thus, not be further considered in this work. Additionally, tools like BubbleTree[18] and AbsCN-seq [19], estimating and visualizing tumor purity, ploidy and copy numbers, are not considered due to different scope. The tool MapScape [20] is not considered as visualization focuses on spatial clonal evolution, linking anatomical images to tumor samples

A commonly used approach for visualizing clonal evolution by means of fish plots is the R package fishplot [13]. The R/Bioconductor package timescape [14] provides an alternative approach, visualizing clonal evolution by interactive fish plots linked to standard graphs. A detailed evaluation of both approaches in comparison to our novel approach clevRvis is performed.

We could not identify any tool visualizing bi-allelic events in clonal evolution for comparison with our plaice plot module. Therefore, representation by plaice plots is compared to dolphin/fish plots as well as manual evaluation of bi-allelic events.

Table 2. Overview of main analysis features, comparing the approaches fishplot [13], timescape [14] and clevRvis.

	fishplot	timescape	clevRvis
**Visualization options**			
Graph		x	x
Fish plot	x	x	x
+ healthy alleles			x
**Clonal evolution models**			
Linear	x	x	x
Branched dependent	x	x	x
Branched independent	x		x
**Basic features**			
Graphical user interface			x
Validity check[1]	x		x
Interactive plots		x	x
Single time point		x	x
Single clone	x		x
**Advanced features**			
Exploring alternative trees			x
Time point interpolation			x
Therapy effect estimation			x
Phylogeny-aware color coding[2]			x

[1]We consider a validity check to be able to detect errors in the logic of clonal evolution, e.g. the CCF of a children-clone exceeding the CCF of its parent-clone, or CCFs at one time point summing up to > 100%.
[2]We consider phylogeny-aware color coding to indicate the degree of relatedness of two clones. This includes clones developing in a linear, branched dependent and branched independent manner.

Results

We apply clevRvis to four real, publically available data sets and compare performance of our approach to the commonly used R packages fishplot [13] and timescape [14]. Results for three exemplary samples are visualized in Figure 2. Detailed results, considering all 31 samples, are available in Additional file 1 (data set 1: Figures S11-S21; data set 2: Figures S22, S23; data set 3: Figures S24-S34; data set 4: Figures S35-S45). Main analysis features of all three algorithms are summed up in Table 2.

All three approaches are able to generate fish plots for visualization of clonal evolution (called "dolphin plots" in clevRvis). Additionally, timescape and clevRvis generate graphs ("shark plots" in clevRvis), representing the underlying phylogeny. However, clevRvis is the only approach providing an option to visualize information on healthy alleles and their development over time in terms of clonal evolution ("plaice plots").

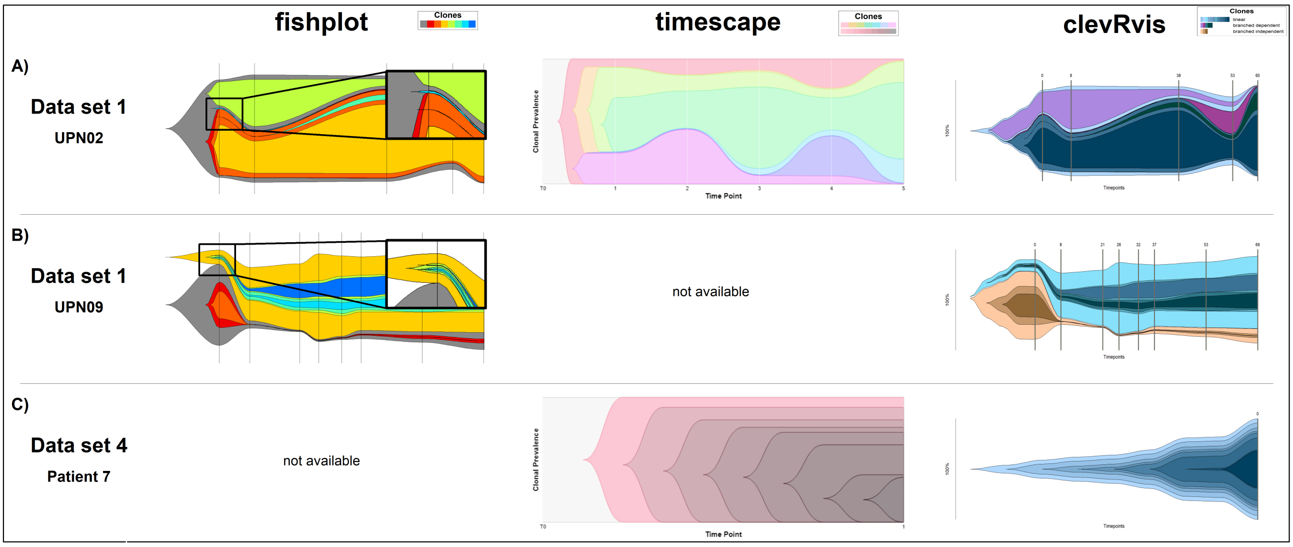

Figure 2. Visualization of clonal evolution using fish plots, comparing the approaches fishplot, timescape and clevRvis. A) Data set 1, UPN02: branched dependent evolution can be visualized by all three tools. However, starting points of two clones are not correctly displayed using fishplot. B) Data set 1, UPN09: branched independent evolution can only be visualized by fishplot and clevRvis. The starting point of one clone is not correctly displayed using fishplot. C) Data set 4, Patient 7: linear evolution based on a single time point can only be visualized by timescape and clevRvis.

It can be observed that the three main models of clonal evolution – linear, branched dependent and branched independent – can generally be considered by all three tools. The only exception is branched independent evolution, which cannot be visualized using timescape (Figure 2B; data set 1: patients UPN08, 09, 10, Figures S18-S20; data set 3: patient 4, Figures S27-S29). The tool mandatorily requires all clones to be present in a single tree. Additionally, timescape is not capable of visualizing clonal evolution, if only a single clone is present (data set 1: patient UPN04, Figure S14). Fishplot, on the contrary, struggles with visualizing data available at a single time point (Figure 2C; data set 3: patients 1-3, Figures S24-S26; data set 4: patients 6-10, Figures S40-S45). Furthermore, the visualization with fishplot partly suggests a wrong starting point of the clone, despite correct definition of the input (Figure 2A and 2B; data set 1: patients UPN02, 03, 07, 09, 10, Figures S12, S13, S17, S19, S20; data set 4: patients 2 and 5, Figures S36, S39).

clevRvis is the only approach providing a graphical user interface, allowing for user-friendly analysis of clonal evolution. Plots can be easily customized, e.g. interactively moving labels of the clones along the x- and y-coordinates, or picking colors and transparency levels for the clones' borders from a wide palette. By default, all plots are interactive. When hovering over a clone, its CCF is displayed and the clone is highlighted – in case of shark and dolphin plots, which are interactively connected, in both plots. Moreover, clevRvis is the only tool providing advanced features, exceeding the basic visualization of clonal evolution. These include fully automatic algorithms for time point interpolation and therapy effect estimation. Phylogeny-aware color coding is implemented as well as an algorithm for exploring alternative trees.

A detailed description on the usage of clevRvis, including exemplary input files and executable examples, is provided along with the package (manuals and vignette). A tutorial, including a complete walk-through, is additionally provided in the shiny-app.

Time point interpolation and therapy effect estimation

clevRvis provides algorithms for approximating the development of clonal evolution in between two measured time points, by interpolating additional time points as well as estimating the effect of a therapy applied. To investigate performance of our algorithms, we consider data sets 1 and 2. Results considering 4 exemplary patients are summed up in Figure 3.

Patients in data set 1 are characterized by a high number of measured time points (up to 30). Analysis with clevRvis is performed twice: 1) evaluating all measured time points; 2) evaluating the first and last measured time point only. Six patients (UPN03, 04, 05, 06, 07 and 11) received supportive care only. Thus, development of clonal evolution is best approximated by time point interpolation. For patient UPN07 (Figure 3A) it can be observed that despite a certain simplification in the development, clonal evolution estimated by clevRvis is highly comparable to the original course. Similar results can be observed for patient UPN08. As the patient was treated with lenalidomide, we compare clonal evolution based on 12 measured time points to the estimated development, based on only 2 measured time points + interpolated time points + estimated therapy effect (Figure 3B).

For patient UPN02, also receiving treatment with lenalidomide, certain differences can be observed (Figure 3C). The magenta clone can barely be observed in the development estimated by clevRvis (measured with $CCF = 22\%$ at time point t_{53}). However, as the clone is present with $CCF < 1\%$ at both t_0 and t_{60}, it appears basically impossible to predict its unexpected rise and subsequent fall at an intervening time point. Despite this apparent difference, the estimated clonal evolution reflects the main characteristics of the true clonal development of this patient (results for all patients available in Additional file 1, Figures S11-S21).

Data set 2 contains information on 2 patients: patient 1 characterized by 15 and patient 2 characterized by 4 time points. Clonal evolution of patient 2, considering all available time points, is displayed in Figure 3D. González-Rincón et al. [12] report that the sample at t_{408} was taken before treatment. Subsequently, the patient received treatment with FCR (fludarabin, cyclophosphamid, rituximab) followed by maintenance therapy with rituximab. The patient was reported to achieve complete response. However, clonal evolution only based on the measured time points does not reflect this response. From the results published, we estimate that FCR was given for roughly one year. Therefore, we estimate therapy effect for t_{700}. The resulting plot in Figure 3E shows a considerable effect of therapy on clonal evolution, matching the described course of disease (results for both patients available in Additional file 1, Figures S22, S23).

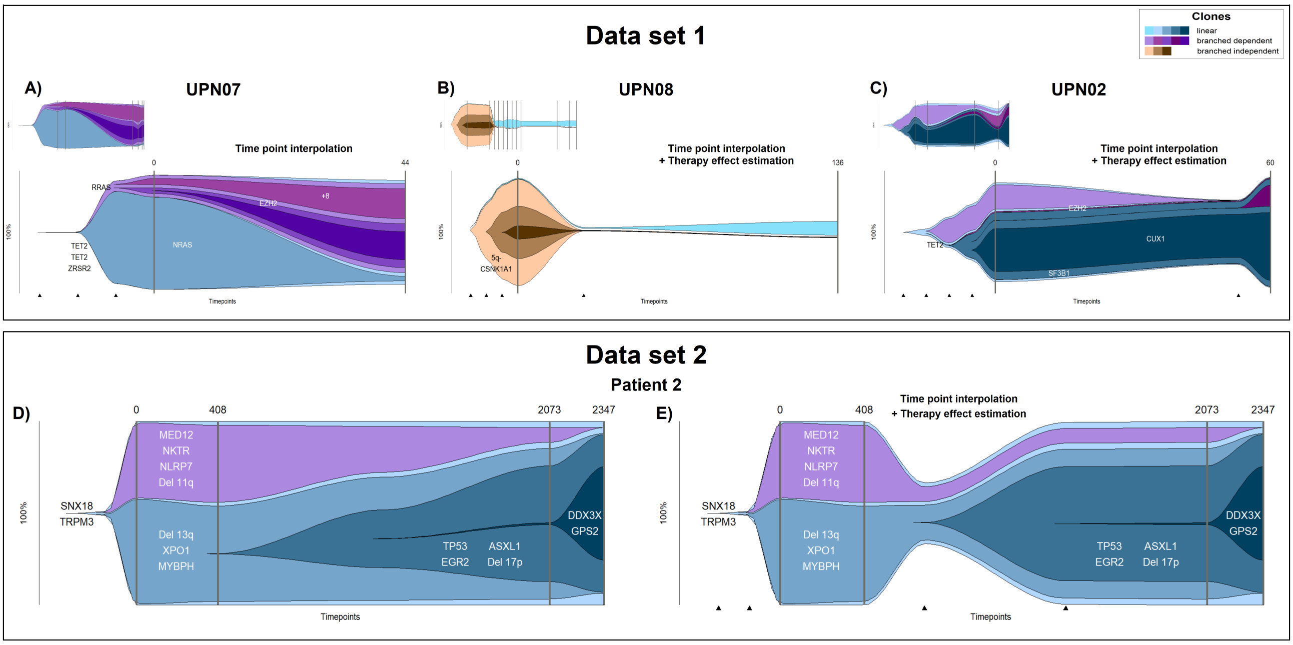

Figure 3. Visualization of clonal evolution using dolphin plots. A) Data set 1, UPN07: analysis of 6 time points vs analysis of 2 time points and additional time point interpolation. B) Data set 1, UPN08: analysis of 12 measured time points vs analysis of 2 measured time points, additional time point interpolation and therapy effect estimation. C) Data set 1, UPN02: analysis of 5 measured time points vs analysis of 2 measured time points, additional time point interpolation and therapy effect estimation. D) Data set 2, patient 2: analysis of 4 measured time points. E) Data set 2, patient 2: analysis of 4 measured time points and additional therapy effect estimation. Note: for all plots, time point interpolation is enabled to improve visualization of newly developing clones prior to the first measured time point.

Detecting bi-allelic events

clevRvis contains a novel plotting option for clonal evolution – plaice plots. These plots allow for identification of bi-allelic events. Considering data sets 1 to 4, we investigate applicability and added value of an analysis by plaice plots. Results considering 8 exemplary patients are summed up in Figure 4.

Patient UPN05 in data set 1 is characterized by branched dependent evolution, with a total of 7 clones. The first clone features, among others, a point mutation in *BCOR*. The gene is located on the X-chromosome. As the patient is male, the hemizygous variant leads to a loss of the only available copy of *BCOR*. In the plaice plot, the first and all subsequent clones are marked, indicating a missing healthy allele of *BCOR* (Figure 4A) (plaice plots for all patients in data set 1 available in Additional file 1, Figures S11-S21).

Patient 1 in data set 2 features a splicing variant in *TP53* (Figure 4B; light blue clone). Subsequently, the patient acquires a deletion in chromosome 17 (17p13.1 del), overlapping *TP53*. The CNV is clustered in clone 2 (intermediate blue). As this event leads to a loss of the only available allele of *TP53*, clone 2 is marked in the plaice plot. Light blue – the color of clone 1, characterized by the initial variant in *TP53* – is chosen for coloring. Additionally, clone 3 (dark blue) features deficient *TBC1D4* (13q14.3 del + point mutation) and *UBA1* (X-chromosomal variant in a male patient) (plaice plots for both patients in data set 2 available in Additional file 1, Figures S22, S23).

For patient UPN06 in data set 3, clonal evolution cannot be reconstructed uniquely (Figure 4C). The patient features two variants affecting *TP53*: a point mutation (p.Val272Met) and a derivative chromosome 17 (der(17)t(13;17)(q21;p12)). Data does not allow for deciphering, which of the two variants developed first. Branched dependent (version 1) as well as linear (version 2) evolution can be reconstructed. In addition to the difference in clonal evolution model, the effect on *TP53* differs considerably: in version 1, plaice plots show *TP53* deficiency in 22-25% of the cells. In version 2, on the contrary, all cells contain at least one healthy copy of *TP53* throughout the entire period of follow-up (plaice plots for all patients in data set 3 available in Additional file 1, Figures S24-S34).

Relapsing patients in data set 4 (patients 1 to 5) are – different from non-relapse patients – characterized by $\geq$ 2 variants affecting *TP53*. Analysis and visualization with plaice plots shows that patients 2, 3 and 5 are characterized by deficient *TP53* in a majority of cells (Figure 4D). The stem line of patient 1 is characterized by a point mutation in *TP53* (p.Arg248Gln) and an overlapping CNV. The duplication affects the mutated allele of *TP53*, however, a ratio of 1:2 for healthy:mutated remains. Thus, no clone is marked in the lower plaice plot. For patient 4, a point mutation in *TP53* (p.Arg248Gln) is detected in the second to last clone. A CNV below detection thresholds is assumed to be additionally present. However, data does not allow to decide on whether it is a deletion or duplication. Thus, it is unclear whether a healthy copy of *TP53* remains (plaice plots for all patients in data set 4 available in Additional file 1, Figures S35-S45).

Discussion

clevRvis is an R/Bioconductor package, providing innovative visualization techniques for clonal evolution. The optimized, highly customizable implementation of established visualization approaches (shark plots, dolphin plots) is complemented by a unique allele-aware representation of clonal evolution, allowing for analysis of bi-allelic events at a glance: plaice plots. In addition, the tool contains fully automatic algorithms for time point interpolation and therapy effect estimation, phylogeny-aware color-coding, exploring alternative trees as well as a graphical user interface for intuitive usage not just by computer scientists, but also biologists and physicians.

To our knowledge, only two alternative approaches for visualizing clonal evolution exist: fishplot [13] and timescape [14]. With respect to functionalities, our novel approach unites all options of currently available tools and provides a wide set of additional features. Analyzing four publically available data sets, it can be observed that plots generated with clevRvis allow for an improved visualization of clonal evolution, outperforming both fishplot and timescape. Furthermore, new insights into disease course can be gained and reasons explored for therapy failure and relapse.

As regards suitable input data, the analysis of clonal evolution faces a major challenge: commonly, the number of tumor samples

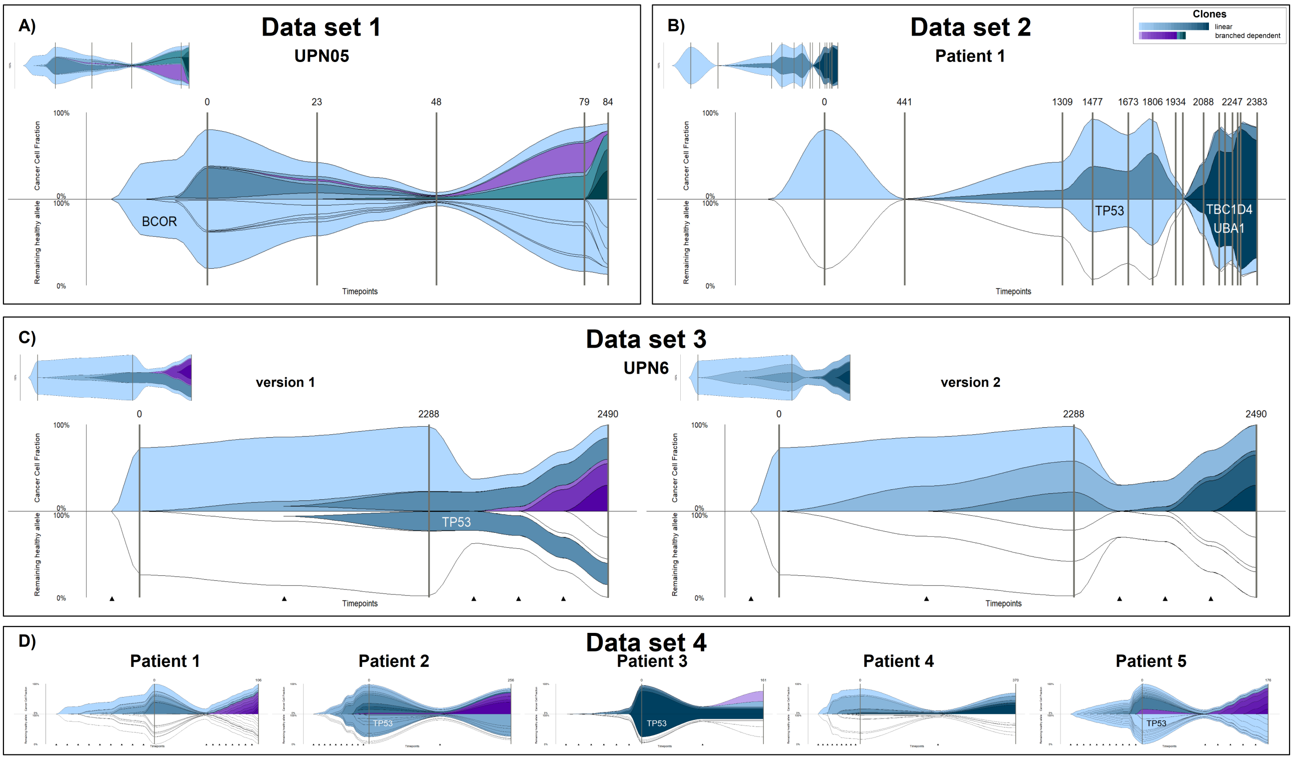

Figure 4. Visualization of clonal evolution using plaice plots vs dolphin plots. A) Data set 1, UPN05: A hemizygous variant affects *BCOR*. B) Data set 2, patient 1: A bi-allelic event causes *TP53* deficiency in clone 2. Clone 3 is characterized by additional *TBC1D4* and *UBA1* deficiency. C) Data set 3, UPN06 (enabled therapy effect estimation): A double-hit event affects *TP53* (der(17)t(13;17)(q21;p12) + point mutation). Clonal evolution cannot be reconstructed uniquely. Version 1 assumes branched dependent evolution, leading to *TP53* deficiency in one clone. Version 2 two assumes linear clonal evolution, leaving at least one healthy allele of *TP53* in each clone. D) Data set 4, patients 1 to 5: Different bi-allelic events lead to *TP53* deficiency in patients 2, 3 and 5. Note: for all plots, time point interpolation is enabled to improve visualization of newly developing clones prior to the first measured time point.

available heavily depends on the tumor itself. For non-solid tumors, bone marrow or peripheral blood are commonly analyzed (e.g. [6], [8]). Taking into account a patient's burden, performing regular bone marrow biopsies is ethically difficult to justify, especially towards the end of a therapy that is expected to lead to remission. For solid tumors, e.g. brain tumors, collecting samples after therapy is practically impossible as long as no relapse is observed. Thus, valid approaches estimating the development of a tumor and its response to therapy are of high relevance.

The two algorithms for time point interpolation and therapy effect estimation, inspired by the general idea outlined by Reutter et al. [8], mark a central element of clevRvis. However, the assumptions, on which these algorithms are based, can be discussed.

Interpolating initial development of a tumor towards the first measured time point t_1, we focus on the difference in CCFs (ccf'). If ccf' of clone A (=stemline) is 20% at t_1, we assume that it is also 20% at every interpolated initial time point. It is, of course, possible that clone A temporarily reaches values > 20% and is – towards t_1 – pushed away by clone B, resulting in a decrease of ccf'. On the contrary, it is also possible that clone A expands only slowly and values < 20% are observed prior to t_1. As we could not find evidence for one of the two scenarios being generally more likely, we decided – as a compromise – to focus only on ccf' at t_1, which may result in an underestimation in some cases and an overestimation in other cases.

In the absence of therapy, it appears sensible to assume that the overall tumor load never decreases. Interpolating development of a tumor between two measured time points with no therapy being applied, we therefore assume linear development of CCFs. While focusing on the difference in CCFs would be a valid alternative approach for clones showing an increase in ccf', it can lead to a violation of our main assumption in case of decreasing ccf' (new, quickly expanding clones pushing away existing clones). As the overall tumor load is not expected to decrease at any (interpolated) time point, we decided to stick to linear development for all clones. New clones are assumed to develop successively, based on their nested level.

In the presence of therapy, we assume that every observable decrease in CCF is related to therapy. Focusing again on the difference in CCFs, the minimum ccf' of the measured time points prior (t_i) and after (t_{i+1}) estimated therapy effect is considered. If a decrease in ccf' can be observed, we assume that it is caused by therapy. If an increase is observed, we assume that the clone is resistant to therapy and was able to expand after the end of therapy. Similar to interpolation of the initial development, it is possible that this approach overestimates therapy effect in some cases, while underestimating it in others. We consider our approach a compromise, approximating true development in a majority of cases.

Due to limited data being available, e.g. data set 4, we could not proof validity of our algorithms for all patients. However, evaluating two public data sets, we could show that our algorithms indeed provide valid solutions for interpolating time points and estimating therapy effect. Partly, they even revealed new insights into tumor development (data set 2, patient 2; Figure 3D vs 3E). While our analysis also showed that the extreme development of a clone, e.g. a considerable increase followed by subsequent decrease, in between two measured time points cannot be approximated, it remains questionable whether any algorithm would be capable of predicting this unexpected behaviour in the lack of sufficient data.

In addition to approximating a tumor's development, the analysis of clonal evolution on allele-level represents another central aspect of clevRvis. While bi-allelic events are considered of high relevance, their analysis commonly requires tedious manual inspection of the variants characterizing each clone. Considering CNVs, genetic expert knowledge is often required to decipher partly complex karyotypes. Additionally, it may be necessary to consider raw

sequencing data. While specific clones featuring bi-allelic events could also be highlighted in a common fish plot, our newly developed plaice plots provide a unique option to 1) easily convey information on bi-allelic events and 2) link this information to characteristic clones by suitable color-coding. As shown in Figure 4B (data set 2, patient 1), this does not necessarily refer to the same clone. It may of course be argued that it is still necessary to manually define the clones to color in the lower plaice plot once. Subsequently, however, this information can be evaluated at a glance by physicians, biologists and even computer scientists.

Compared to dolphin plots, it may be considered a disadvantage that the actual clonal evolution is only displayed in the upper half of a plaice plot, making complex clonal evolution patterns potentially difficult to see. However, real examples of complex clonal evolution (e.g. data set 1, UPN10: 8 clones, dependent and independent branches, Additional file 1, Figure S20C vs S20D; data set 4, patient 5: 17 clones, linear development, Additional file 1, Figure S39D vs S39E) show that all clones can still be distinguished clearly. Simulated data, considering even more complex clonal evolution (100 clones and 100 time points; Additional file 1, section 2.5), show that all plot types implemented in clevRvis – including plaice plots – allow for visualization of a high number of clones as well as a high number of time points.

Conclusion

clevRvis provides an extensive set of visualization techniques for clonal evolution. Exceeding currently available approaches, clevRvis allows for approximating of a tumor's development in between measured time points as well as analyzing bi-allelic events. Our future work will include an extension of the clevRvis package, including approaches for considering multiple spatial locations per time point, as well as visualizing changes in gene expression and methlyation along common clonal evolution, based on a tumor's mutational profile.

Availability of source code and requirements

- Project name: clevRvis
- Project home page: https://github.com/sandmanns/clevRvis
- Bioconductor: https://bioconductor.org/packages/clevRvis
- Operating system(s): Platform independent
- Programming language: R
- Other requirements: none
- License: LGPL-3.0
- bio.tools ID: biotools:clevRvis
- SciCrunch ID: RRID:SCR_023154

Availability of supporting data and materials

An archival copy of the code and supporting data is available via the GigaScience repository, GigaDB [21]. Further material supporting the results of this article are available in Additional file 1.

Additional file 1

SupplementaryInformation.pdf: information on supplementary methods and results.

Declarations

List of abbreviations

aCGH: array-CGH; AML: acute myeloid leukemia; BL: Burkitt lymphoma; CCF: cancer cell fraction; CLL: chronic lymphatic leukemia; CNV: copy number variant; indels: insertions/deletions; FCR: fludarabin, cyclophosphamid, rituximab; FISH: fluorescence *in situ* hybridization; IPSS-R: Revised International Prognostic Scoring System; MDS: myelodysplastic syndromes; MM: multiple myeloma; MPN: myeloid neoplasia; NGS: next-generation sequencing; R-ISS: Revised International Staging System; SV: structural variant; tNGS: targeted next-generation sequencing; VAF: variant allele frequency; WES: whole-exome sequencing.

Ethical Approval

All patient material was collected and analyzed in accordance with the relevant ethical guidelines and regulations. Informed consent was obtained from all subjects.

Consent for publication

Not applicable

Competing Interests

The authors declare that they have no competing interests.

Funding

This research was supported by the Open Access Publication Fund of the University of Muenster. There was no additional external funding received. The funding body had no role in the design of the study, collection, analysis, and interpretation of data, decision to publish, or in writing the manuscript.

Author's Contributions

S.S. conceptualized the project, developed the algorithm, supervised and supported implementation of the algorithm, performed data analyses, and wrote the manuscript. C.I. implemented the algorithm. J.V. supervised the project and reviewed the manuscript. All authors read, revised, and approved the final version of the manuscript.

Acknowledgements

Not applicable

References

1. Palumbo A, Avet-Loiseau H, Oliva S, Lokhorst HM, Goldschmidt H, Rosinol L, et al. Revised International Staging System for Multiple Myeloma: A Report From International Myeloma Working Group. J Clin Oncol 2015;33:2863–2869.
2. Rajkumar SV. Multiple Myeloma: 2020 Update on Diagnosis, Risk-Stratification and Management. Am J Hematol 2020;95:548–567.
3. Greenberg PL, Tuechler H, Schanz J, Sanz G, Garcia-Manero G, Solé F, et al. Revised international prognostic scoring system for myelodysplastic syndromes. Blood 2012;120:2454–2465.
4. Malcovati L, Crouch S, de Graaf AO, Sandmann S, Tobiasson M, Kosmider O, et al. Mutation Profiles Identify Distinct Clusters of Lower Risk Myelodysplastic Syndromes with Unique

Clinical and Biological Features and Clinical Endpoints. Blood 136(Supplement 1) 2020;p. 2454–2465.
5. Nowell PC. The Clonal Evolution of Tumor Cell Populations. Science 1976;194:23–28.
6. da Silva-Coelho P, Kroeze LI, Yoshida K, Koorenhof-Scheele TN, Knops R, van de Locht LT, et al. Clonal evolution in myelodysplastic syndromes. Nat Commun 2017;8:15099.
7. Sandmann S, Behrens YL, Davenport C, Thol F, Heuser M, Dörfel D, et al. Clonal Evolution at First Sight: A Combined Visualization of Diverse Diagnostic Methods Improves Understanding of Leukemic Progression. Front Oncol 2022;12:888114.
8. Reutter K, Sandmann S, Rohde J, Müller S, Wöste M, Khanam T, et al. Reconstructing clonal evolution in relapsed and non-relapsed Burkitt lymphoma. Leukemia 2021;35:639–643.
9. Davies A, Gao R, Navin N. Tumor evolution: Linear, branching, neutral or punctuated? Biochim Biophys Acta Rev Cancer 2017;1867:151–161.
10. Sandmann S, Karsch K, Bartel P, Exeler R, Brix TJ, Mai EK, et al. The Role of Clonal Evolution on Progression, Blood Parameters, and Response to Therapy in Multiple Myeloma. Front Oncol 2022;12:919278.
11. Krzywinski M. Visualizing Clonal Evolution in Cancer. Mol Cell 2016;62:652–656.
12. González-Rincón J, Gómez S, Martinez N, Troulé K, Perales-Patón J, Derdak S, et al. Clonal dynamics monitoring during clinical evolution in chronic lymphocytic leukaemia. Sci Rep 2019;9:975.
13. Miller CA, McMichael J, Dang HX, Maher CA, Ding L, Ley TJ, et al. Visualizing tumor evolution with the fishplot package for R. BMC Genomics 2016;17:880.
14. Smith M. timescape: Patient Clonal Timescapes. R package version 1.20.0; 2022.
15. Deshwar AG, Vembu S, Yung CK, Jang GH, Stein L, Morris Q. PhyloWGS: reconstructing subclonal composition and evolution from whole-genome sequencing of tumors. Genome Biol 2015;16:35.
16. Jiang Y, Qiu Y, Minn AJ, Zhang NR. Assessing intratumor heterogeneity and tracking longitudinal and spatial clonal evolutionary history by next-generation sequencing. Proc Natl Acad Sci U S A 2016;113:E5528–E5537.
17. Strino F, Parisi F, Micsinai M, Kluger Y. TrAp: a tree approach for fingerprinting subclonal tumor composition. Nucleic Acids Res 2013;41:e165.
18. Zhu W, Kuziora M, Creasy T, Lai Z, Morehouse C, Guo X, et al. BubbleTree: an intuitive visualization to elucidate tumoral aneuploidy and clonality using next generation sequencing data. Nucleic Acids Res 2016;44:e38.
19. Bao L, Pu M, Messer K. AbsCN-seq: a statistical method to estimate tumor purity, ploidy and absolute copy numbers from next-generation sequencing data. Bioinformatics 2014;30:1056–1063.
20. Smith M. mapscape: mapscape. R package version 1.20.0; 2022.
21. Sandmann S, Inserte C, Varghese J. Supporting data for "clevRvis: Visualization Techniques for Clonal Evolution". GigaScience Database; 2023, http://dx.doi.org/10.5524/102371.

LaTeX - Other (clo, cls, sty etc.)

Click here to access/download

Supplementary Material

oup-contemporary.cls

LaTeX - Other (clo, cls, sty etc.)

Click here to access/download

Supplementary Material

vancouver-authoryear.bst

LaTeX - Figure (eps, ps, etc.)

Click here to access/download
Supplementary Material
oup.pdf

LaTeX - Figure (eps, ps, etc.)

Click here to access/download
Supplementary Material
gigascience-logo.pdf

Figure 1

Click here to access/download
Supplementary Material
Figure1.png

Figure 2

Click here to access/download
Supplementary Material
Figure2.png

Figure 3

Click here to access/download
Supplementary Material
Figure3.png

Figure 4

Click here to access/download
Supplementary Material
Figure4.png

Click here to access/download
Supplementary Material
Additional File 1.pdf

LaTeX - Other (clo, cls, sty etc.)

Click here to access/download
Supplementary Material
main.bbl

LaTeX - Main Document (TeX file)

Click here to access/download
Supplementary Material
main.tex
