# Supplementary material for: clevRvis: visualization techniques for clonal evolution
Source: Gigascience. 2023 Apr 11;12:giad020. doi: 10.1093/gigascience/giad020 (PMC10087014; doi:10.1093/gigascience/giad020)
Supplement: giad020_Supplemental_File [file giad020_supplemental_file.pdf]

Additional file 1: Supplementary Information

clevRvis: Visualization Techniques for Clonal Evolution

Contents

1 Additional methods **3**
1.1 Data set 1 – MDS . . . 3
1.2 Data set 2 – CLL . . . 3
1.3 Data set 3 – MPN . . . 3
1.4 Data set 4 – BL . . . 3
1.5 clevRvis . . . 4
1.6 Using time point interpolation to implement skewed events . . . 8
1.7 Recommended color coding for plaice plots . . . 10

2 Additional results **12**
2.1 Data set 1 – MDS . . . 12
2.2 Data set 2 – CLL . . . 23
2.3 Data set 3 – MPN . . . 25
2.4 Data set 4 – BL . . . 36
2.5 Visualizing complex cases of clonal evolution with clevRvis . . . 47

1 Additional methods

1.1 Data set 1 – MDS

For data set 1 – MDS – clonal evolution was analyzed, based on the variants reported by da Silva-Coelho *et al.* [1]. For every single nucleotide variant (SNV), insertion and deletion (indel), the authors report detailed information on the number of reads with the reference, the number of reads with the alternative, the overall coverage, the variant allele frequency (VAF) and the assigned clone. For every copy number variant (CNV), the authors provide information on the number of mutated vs non-mutated cells. Information is available for all measured time points.

For each clone, we estimated the cancer cell fraction (CCF) by averaging over all variants belonging to the corresponding clone. Parental relations were referred from the figures published by da Silva-Coelho *et al.* [1].

The input we used for analysis with clevRvis is summed up in Additional file 2 (Table_S1.tsv).

1.2 Data set 2 – CLL

For data set 2 – CLL – clonal evolution was analyzed, based on the variants reported by González-Rincón *et al.* [2]. For every SNV and indel, the authors report detailed information on the number of reads with the alternative, the overall coverage and the VAF. For the four CNVs analyzed (11q22.3 del, 13q14.3 del, 17p13.1 del and c-myc), the authors provide information on the percentage of affected cells. Information is available for all measured time points.

For a subset of variants, the authors provide information on the assigned clusters (figures in [2]). Evaluating information on coverage and VAF, we reconstructed assignment of the remaining variants. For each clone, we estimated the CCF by averaging over all variants belonging to the corresponding clone. Parental relations were referred from the figures published by González-Rincón *et al.* [2].

The input we used for analysis with clevRvis is summed up in Additional file 3 (Table_S2.tsv).

1.3 Data set 3 – MPN

For data set 3 – MPN – clonal evolution was analyzed, based on the variants reported by Sandmann *et al.* [3]. For every SNV and indel, detailed information on the VAF, the calculated CCF and the adjusted CCF assumed for the reconstructed model of clonal evolution is available. For every CNV, information on the number of mutated vs non-mutated cells is provided. Information is available for all measured time points.

For all variants, information on the assigned clusters and parental relations can be referred from the figures published by Sandmann *et al.* [3].

The input we used for analysis with clevRvis is summed up in Additional file 4 (Table_S3.tsv).

1.4 Data set 4 – BL

For data set 4 – BL – clonal evolution was analyzed, based on the variants reported by Reutter *et al.* [4]. For every SNV and indel, the authors report detailed information on the number of reads with the reference, the number of reads with the alternative, the overall coverage, the variant allele frequency (VAF) and the assigned clone. For every CNV, the authors provide information on the estimated CCF based on SNP-arrays and whole-exome sequencing data. Information is available for all measured time points.

For each clone, we estimated the CCF by averaging over all variants belonging to the corresponding clone. Parental relations are available with the publication [4].

The input we used for analysis with clevRvis is summed up in Additional file 5 (Table_S4.tsv).

1.5 clevRvis

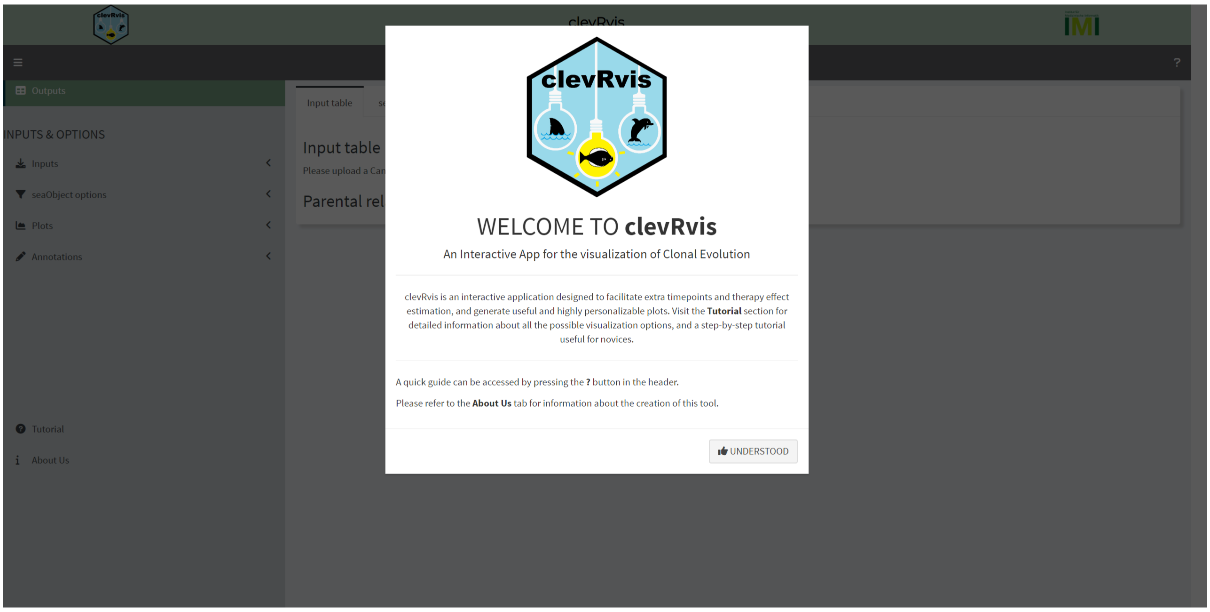

Figure S1: Start screen for the analysis with clevRvis.

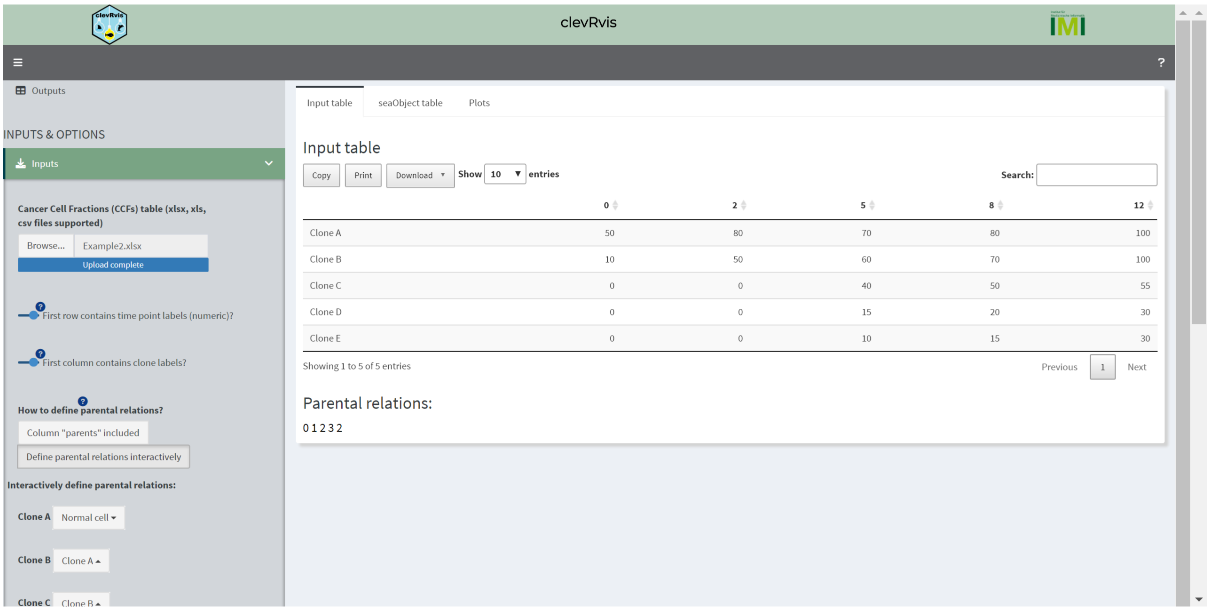

Figure S2: Defining input for the analysis with clevRvis. A table containing the cancer cell fractions characterizing every clone at every time point may be uploaded. Subsequently, parental relations have to be defined interactively.

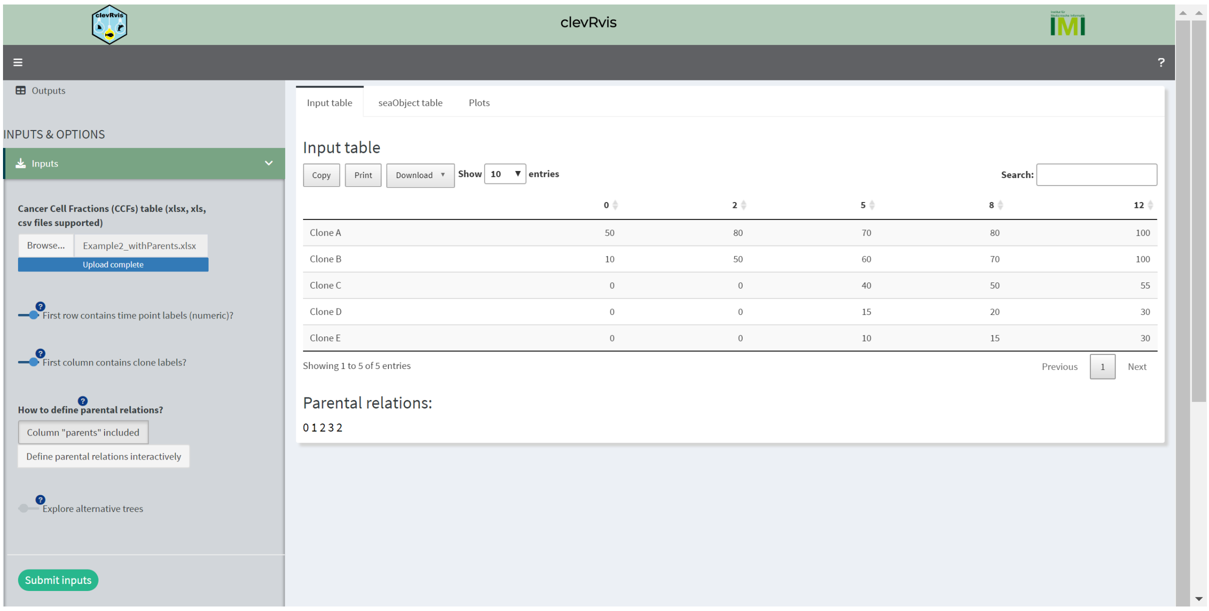

Figure S3: Defining input for the analysis with clevRvis. Alternatively, a table containing the cancer cell fractions characterizing every clone at every time point as well as parental information may be uploaded.

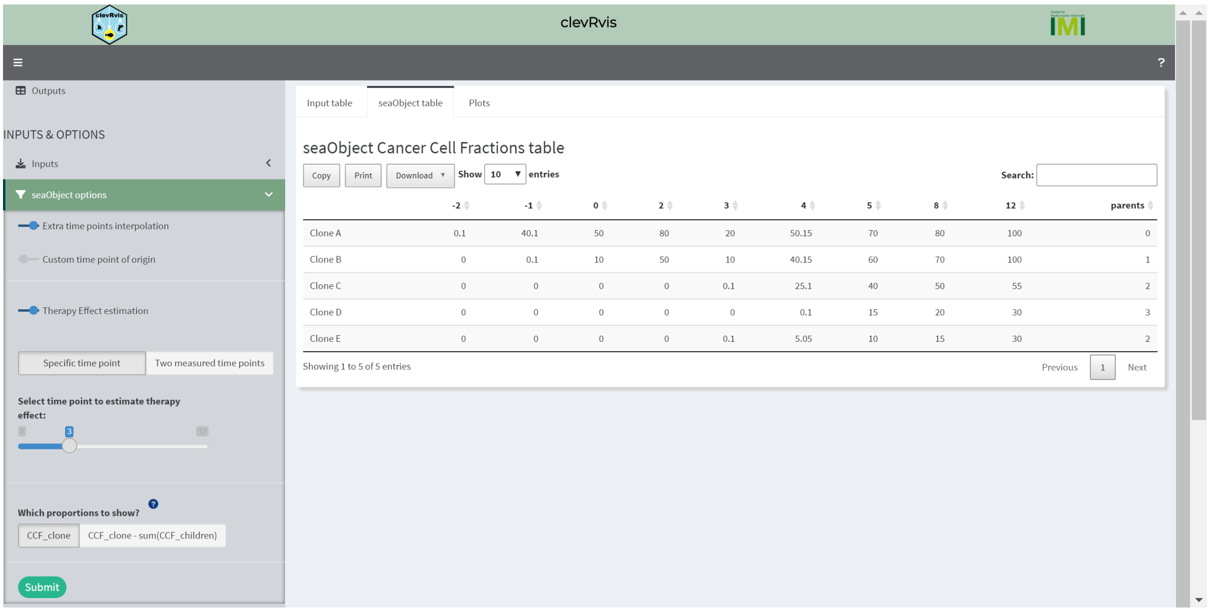

Figure S4: Defining the seaObject for the analysis with clevRvis. Optionally, additional time points may be interpolated, therapy effect may be estimated. The seaObject table contains information on the cancer cell fractions of all clones, at all measured and estimated time points. Additionally, information on parental relations is included in the table.

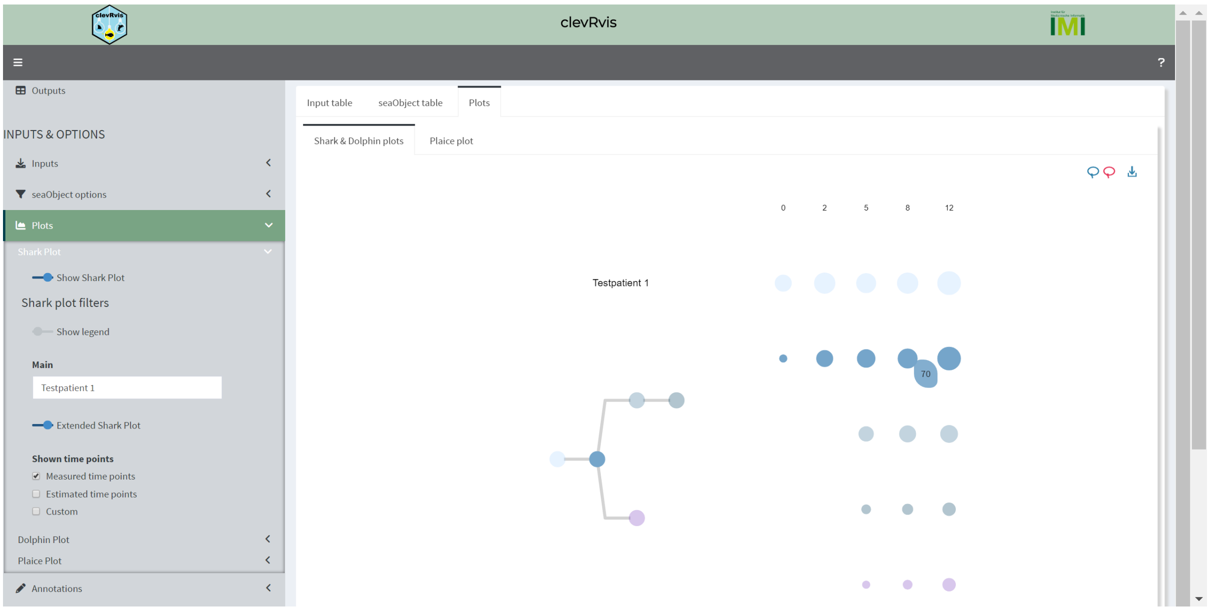

Figure S5: Customized interactive shark plot generated with clevRvis. When hovering over a clone in the graph on the left side, the clone's label is displayed. Additionally, all points on the right side, corresponding to the cancer cell fractions (measured and if selected also estimated), are highlighted. When hovering over a point on the right side, the precise cancer cell fraction is displayed. Additionally, the clone in the graph on the left side is highlighted. If additional dolphin plots are displayed, the corresponding clone is also highlighted in the dolphin plot.

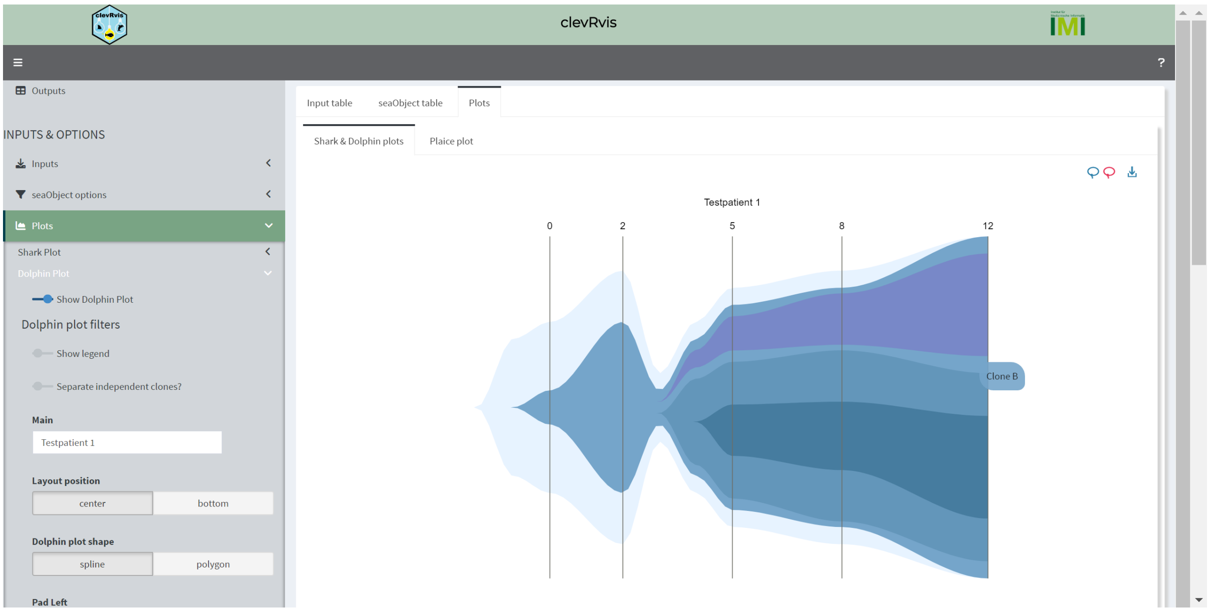

Figure S6: Customized interactive dolphin plot generated with clevRvis. When hovering over a clone, the clone's label is displayed and the CCF development highlighted. If additional shark plots are displayed, the corresponding clone is also highlighted in the shark plot.

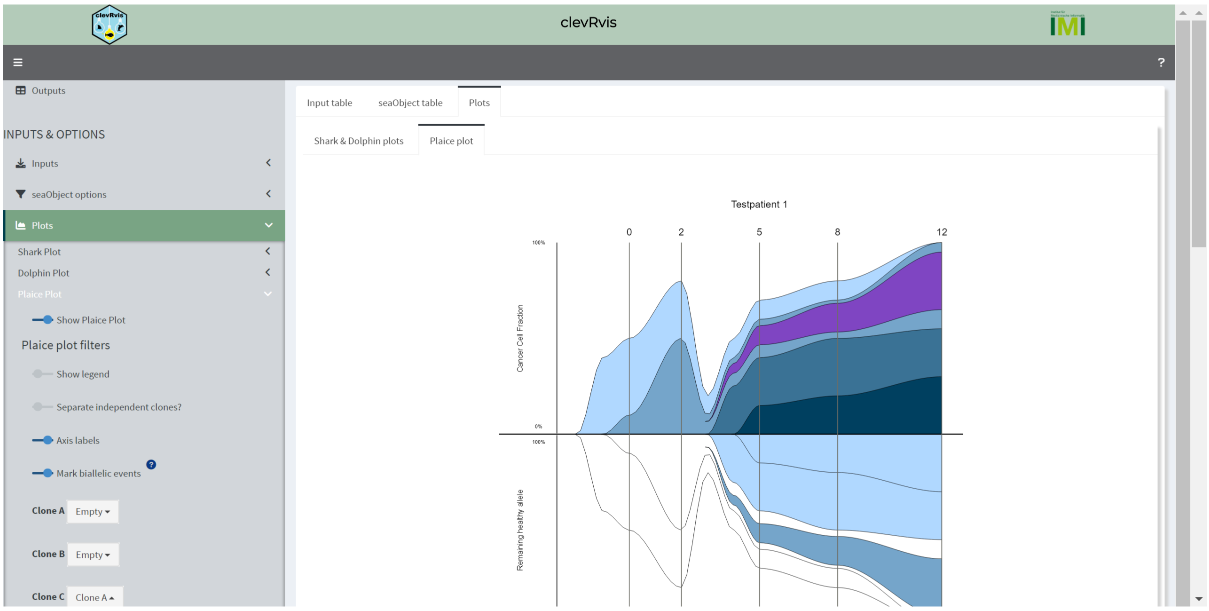

Figure S7: Customized interactive plaice plot generated with clevRvis. When hovering over a clone, the clone's label is displayed and the CCF development highlighted.

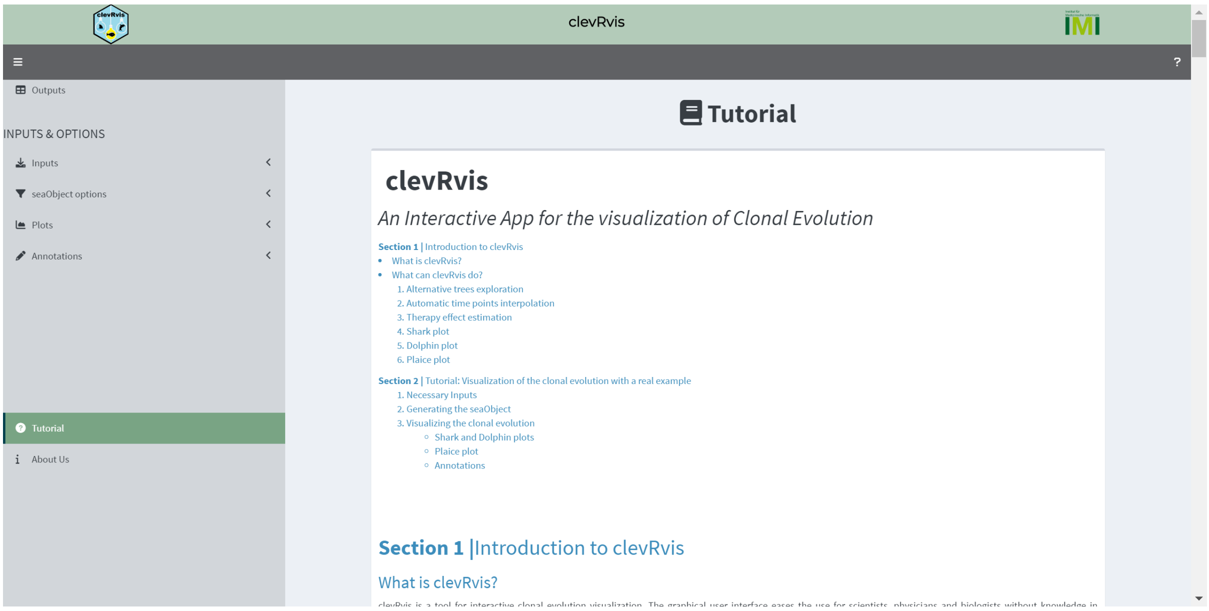

Figure S8: Tutorial describing the use of clevRvis.

1.6 Using time point interpolation to implement skewed events

clevRvis provides an algorithm for fully automatic time point interpolation. By default, all interpolated time points are evenly distributed. An example is given below:

```
timepoints <- c(0,50,100)
parents <- c(0,1,1,3,0,5,6)
fracTable <- matrix(c(20,10,0,0,0,0,0,
                      40,20,15,0,30,10,0,
                      50,25,15,10,40,20,15),
                    ncol = length(timepoints))
seaObject <- createSeaObject(fracTable, parents, timepoints,
                             timepointInterpolation = TRUE)
```

The resulting seaObject is characterized by 3 measured time points and 3 additional interpolated time points. Information on the CCFs at all measured and interpolated time points is available in the slot "fracTable" and can be inspected via `fracTable(seaObject)`:

```
> fracTable(seaObject)
  -50   -25     0    25    50  100
1 0.1  10.1  20.0 30.00  40.0   50
2 0.0   0.1  10.0 15.00  20.0   25
3 0.0   0.0   0.1  7.55  15.0   15
4 0.0   0.0   0.0  0.00   0.1   10
5 0.0   0.0   0.1 15.05  30.0   40
6 0.0   0.0   0.0  0.10  10.0   20
7 0.0   0.0   0.0  0.00   0.1   15
```

The argument "originTimepoint" – available for createSeaObject() – allows a customized definition of the time point, at which the first clone emerges (here default: -50). The remaining interpolated time points, -25 and 25, are evenly distributed and cannot be changed via standard input arguments. However, skewed events can still be visualized using clevRvis. To do so, the slot "timepoints" has to be accessed:

```
> timepoints(seaObject)
[1] -50 -25   0  25  50 100
```

Changing the content of the slot "timepoints" will directly affect the visualized clonal evolution. In the example, clone 6 emerges at interpolated time point 25. If, however, there is evidence that the clone developed later in the considered period between measured time points 0 and 50, e.g. at time point 40, the slot "timepoints" can be changed via

```
timepoints(seaObject)[4] <- 40
```

Dolphin plots, generated from the default and the modified seaObject, using

```
dolphinPlot(seaObject, showLegend = TRUE,
            vlines = timepoints(seaObject),
            vlab = timepoints(seaObject), vlabSize = 7,
            markMeasuredTimepoints = timepoints)
```

are visualized in Figure S9. It can be observed that development of clone 6 is, by default, visualized at time point 25 (Figure S9A). However, by manipulating the "timepoint" slot, a skewed event is generated, moving development of the clone to time point 40 (Figure S9B).

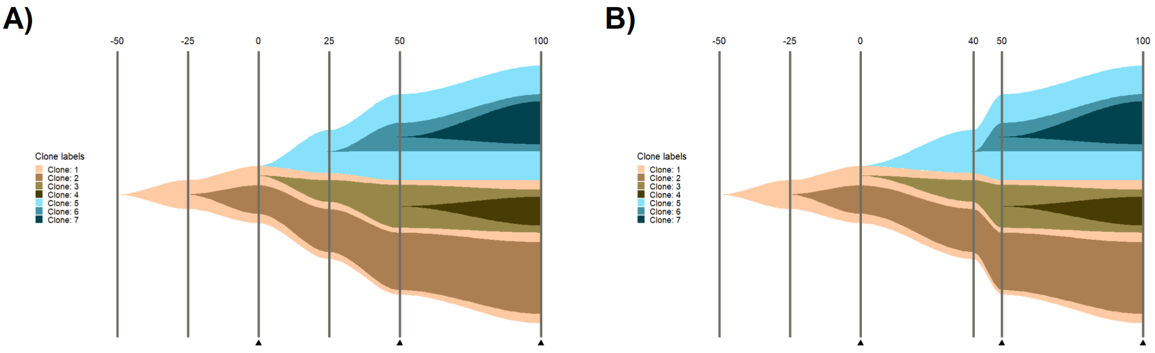

Figure S9: Visualization of clonal evolution in an exemplary simulated patient. A) clevRvis using dolphin plots, enabling time point interpolation. By default, interpolated time points at -50, -25 and 25 are added. B) clevRvis using dolphin plots, enabling time point interpolation. Interpolated time point 25 was manually changed to 40 to visualize a skewed event (development of clone 6).

Of note, the algorithm on time point interpolation has been implemented to generate an optimum visualization of clonal evolution. Changing the default interpolated time points is only encouraged in case of highly experienced users or strong evidence for the presence of skewed events.

1.7 Recommended color coding for plaice plots

Plaice plots provide an option for visualizing clonal evolution on an allelic-level. The upper half of the plot (= upper plaice) shows a common dolphin plot in bottom visualization. The lower plaice shows the percentage of healthy alleles. While clevRvis performs automatic phylogeny-aware color coding for the upper plaice, the lower plaice is – by default - not colored. A user has to define colors for clones, characterized by bi-allelic events, manually. We recommend the following rules for color coding of the lower plaice:

- If a clone contains at least one copy of a healthy allele for all genes, the corresponding clone should not be colored (see Figure S10A).
- If a clone lacks a copy of a healthy allele for at least one gene, the corresponding clone should be colored.
  - If clone 1 carries a point mutation in *TP53* and clone 2 carries an additional deletion of 17p (affecting the remaining healthy allele), clone 2 in the lower plaice should be colored in the same hue as clone 1 in the upper plaice. Clone 1 is originally characterized by a mutation in *TP53*. Thus, color coding indicates that the double-hit event affected *TP53* (see Figure S10B).
  - If clone 1 carries a deletion of 17p and clone 2 carries an additional point mutation in *TP53* (affecting the remaining healthy allele), clone 2 in the lower plaice should be colored in the same hue as clone 2 in the upper plaice. Clone 2 is originally characterized by a mutation in *TP53*. Thus, color coding indicates that the double-hit event affected *TP53* (see Figure S10C).
  - If clone 2 carries two point mutations in *TP53* affecting different alleles, clone 2 in the lower plaice should be colored in the same hue as clone 2 in the upper plaice. Thus, color coding indicates that the double-hit event affected *TP53* (see Figure S10D).
  - If clone 2 carries a variant affecting the X chromosome of a male subject, leading to a loss of the only available healthy allele, clone 2 in the lower plaice should be colored in the same hue as clone 2 in the upper plaice. Thus, color coding indicates presence of a hemizygous variant affecting a gene on the X chromosome.

The color coding rules we propose do not only allow to mark clones affected by bi-allelic events, but – in addition – also link the events to characteristic clones: Clone 2 is in case of Figure S10B, S10C and S10D affected by a bi-allelic event (*TP53* deficiency). However, once it is linked to clone 1 (Figure S10B) and twice to clone 2 (Figure S10C and S10D). As the bi-allelic event only affects *TP53* and not the whole p-arm of chromosome 17, clone 2 in the lower plaice is always linked to the clone in the upper plaice that is originally characterized by a *TP53* mutation.

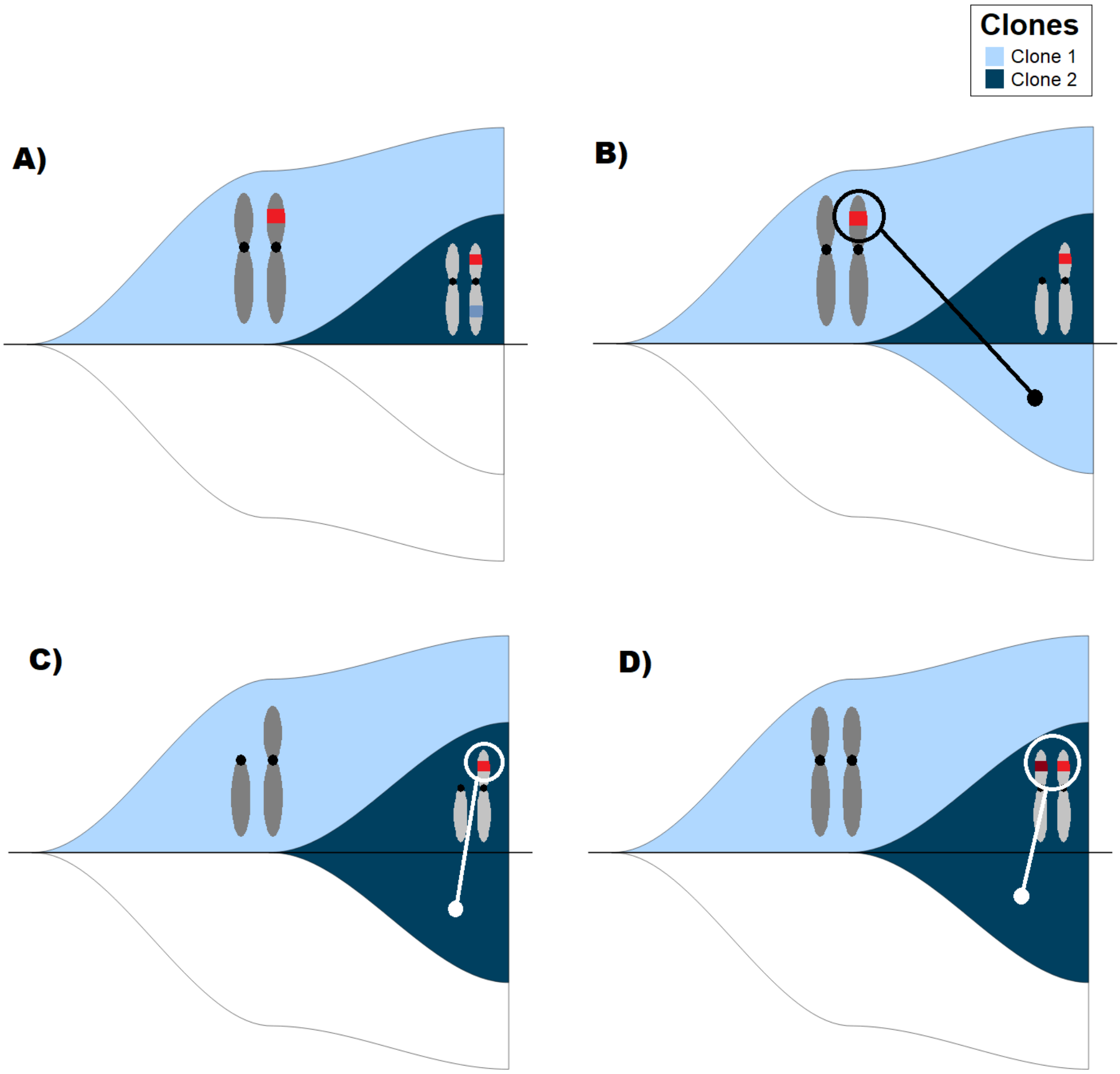

Figure S10: Recommended color coding for plaice plots considering chromosome 17 (red mark indicating mutation in *TP53*). A) No clone in the lower plaice is colored as one copy of a healthy allele remains for all genes. B) Clone 2 in the lower plaice is colored in the same hue as clone 1 in the upper plaice, which is characterized by mutated *TP53*. C) Clone 2 in the lower plaice is colored in the same hue as clone 2 in the upper plaice, which is characterized by mutated *TP53*. D) Clone 2 in the lower plaice is colored in the same hue as clone 2 in the upper plaice, which is characterized by two mutations in *TP53*.

2 Additional results

2.1 Data set 1 – MDS

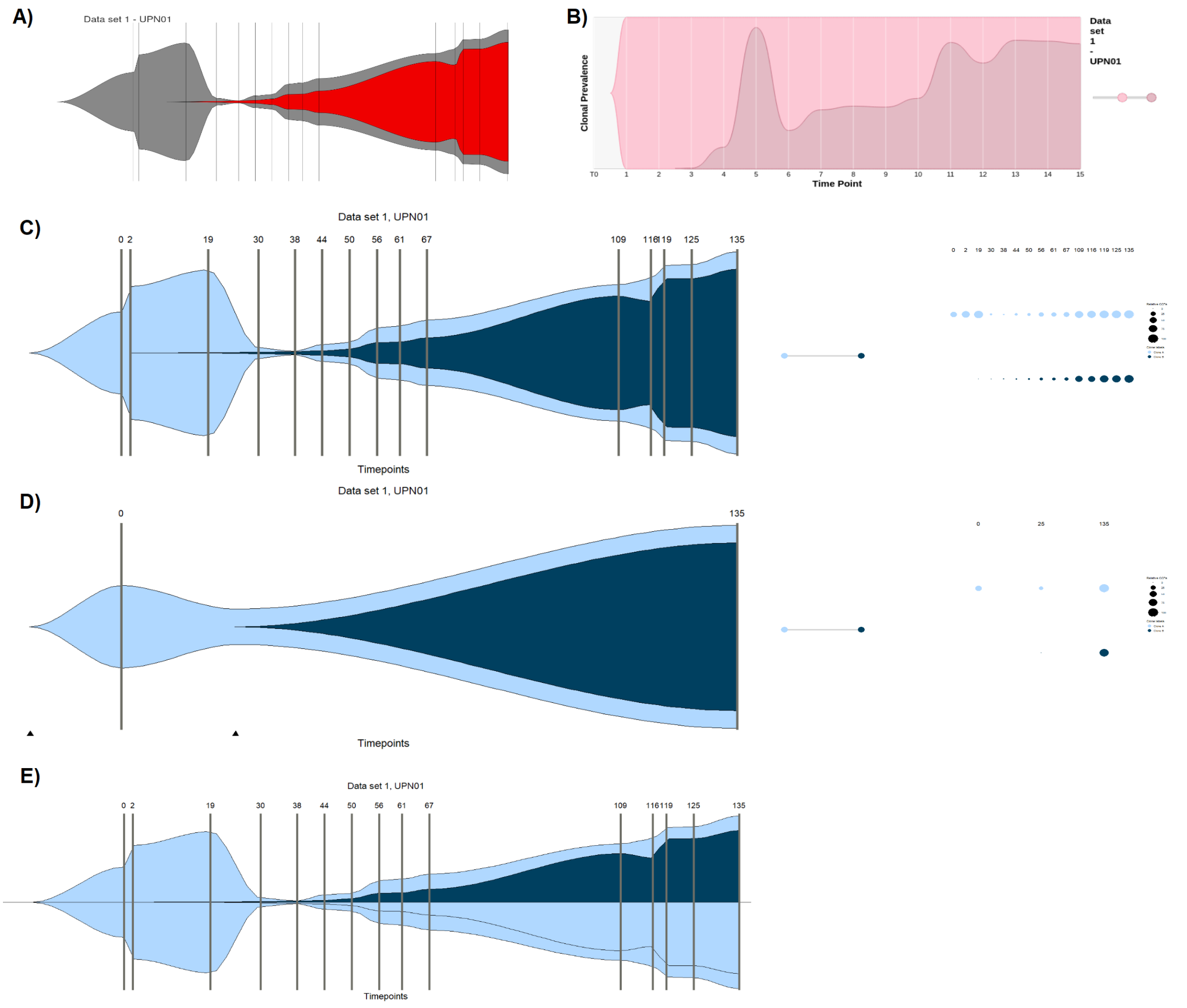

Figure S11: Visualization of clonal evolution of UPN01 in data set 1. A) Fishplot. B) Timescape. C) clevRvis using dolphin plots and extended shark plots, enabling time point interpolation for improved visualization of newly developing clones. D) clevRvis using dolphin plots and extended shark plots, enabling time point interpolation and therapy effect estimation, analyzing only the first and last measured time point. E) clevRvis using plaice plots, enabling time point interpolation. A bi-allelic event affecting *RAD50* (del(5q) + point mutation) is marked.

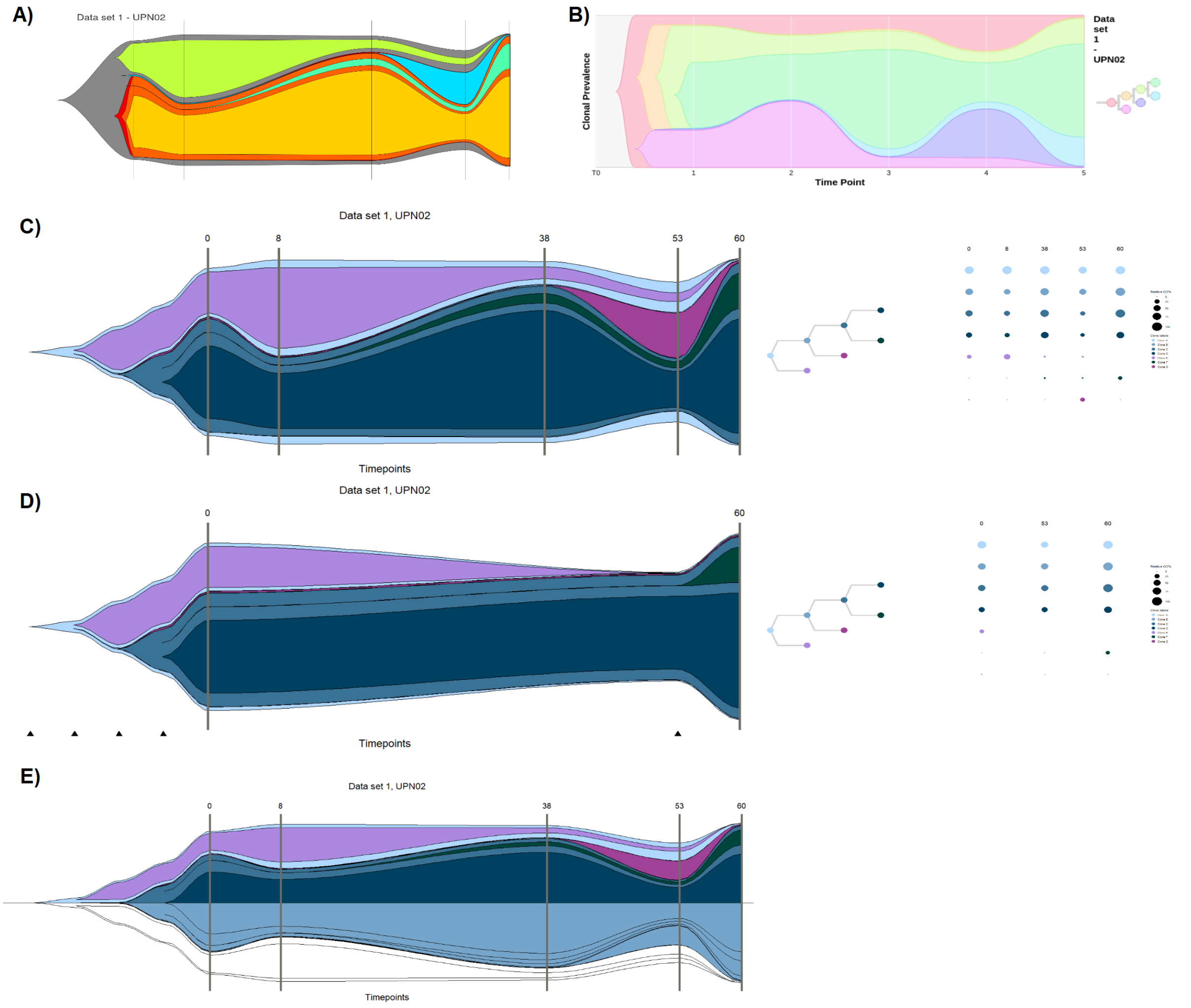

Figure S12: Visualization of clonal evolution of UPN02 in data set 1. A) Fishplot. B) Timescape. C) clevRvis using dolphin plots and extended shark plots, enabling time point interpolation for improved visualization of newly developing clones. D) clevRvis using dolphin plots and extended shark plots, enabling time point interpolation and therapy effect estimation, analyzing only the first and last measured time point. E) clevRvis using plaice plots, enabling time point interpolation. Two bi-allelic events affecting *ODZ2* (2 point mutations) and *TET2* (LOH(4q) + point mutation) are marked.

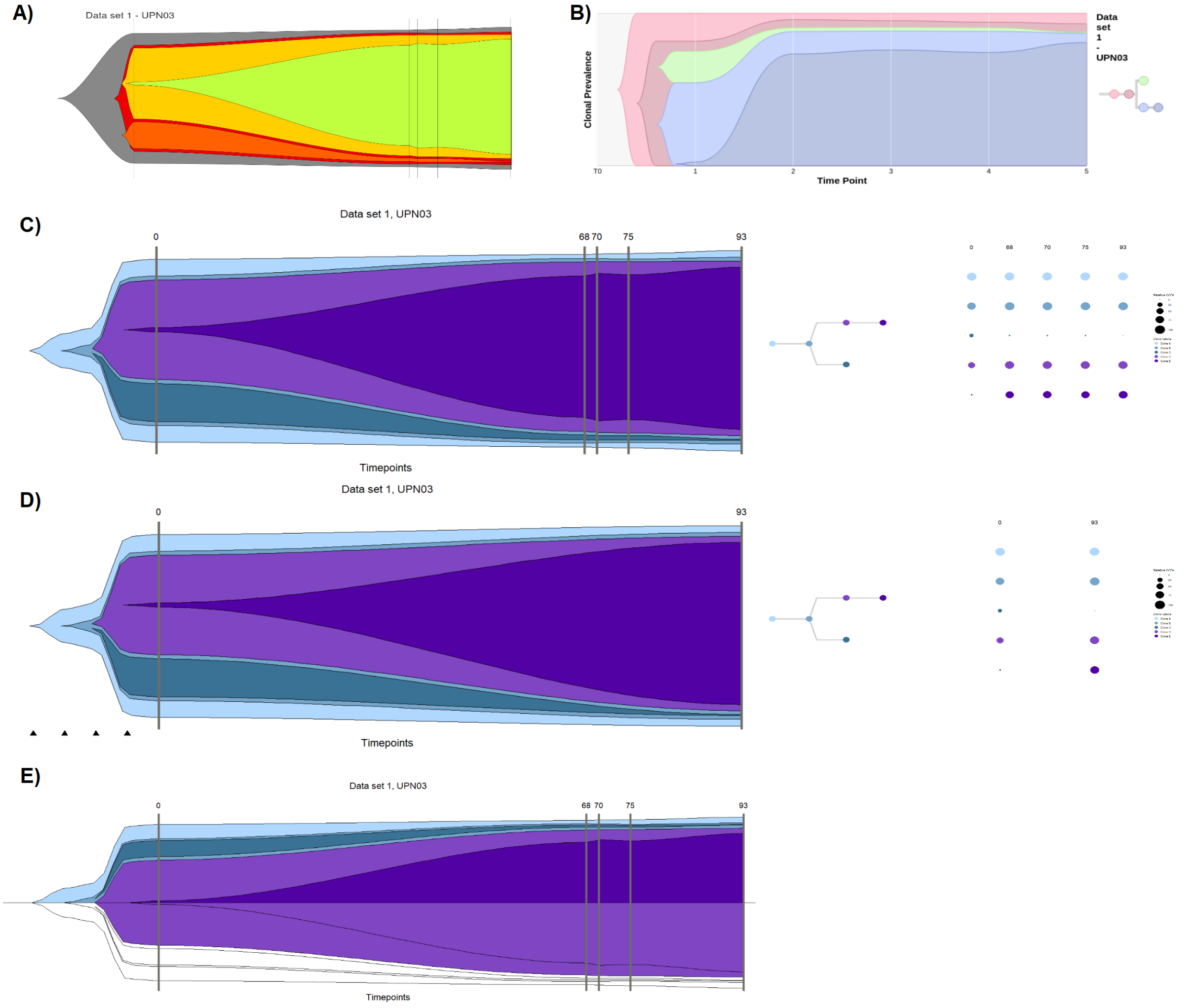

Figure S13: Visualization of clonal evolution of UPN03 in data set 1. A) Fishplot. B) Timescape. C) clevRvis using dolphin plots and extended shark plots, enabling time point interpolation for improved visualization of newly developing clones. D) clevRvis using dolphin plots and extended shark plots, enabling time point interpolation, analyzing only the first and last measured time point. E) clevRvis using plaice plots, enabling time point interpolation. A bi-allelic event affecting *ZNF609* (2 point mutations) is marked.

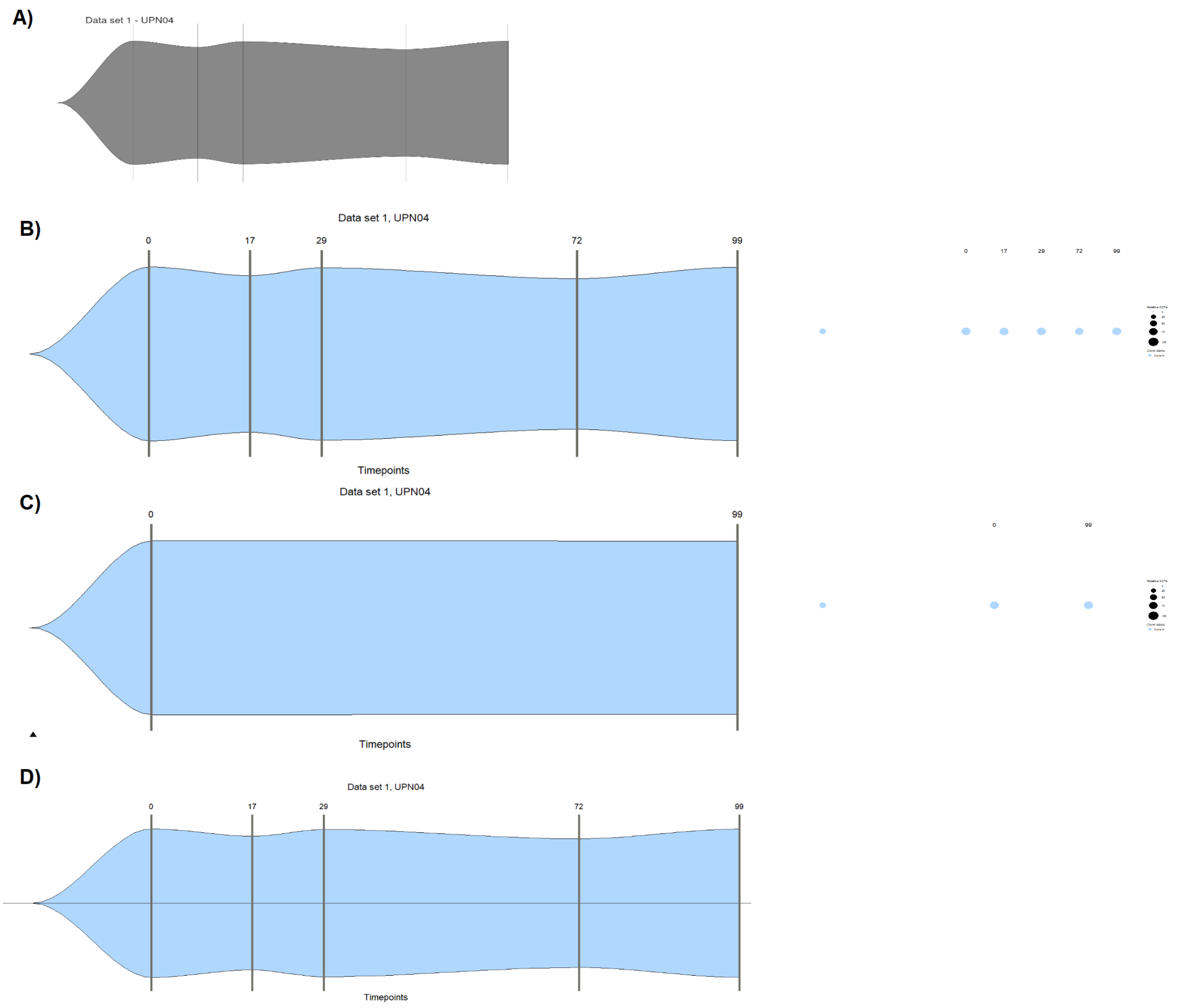

Figure S14: Visualization of clonal evolution of UPN04 in data set 1. A) Fishplot. B) clevRvis using dolphin plots and extended shark plots, enabling time point interpolation for improved visualization of newly developing clones. C) clevRvis using dolphin plots and extended shark plots, enabling time point interpolation, analyzing only the first and last measured time point. D) clevRvis using plaice plots, enabling time point interpolation. A bi-allelic event affecting *TET2* (2 point mutations) and a hemizygous variant affecting *ZRSR2* are marked. Note: Development of a single clone cannot be visualized using timescape.

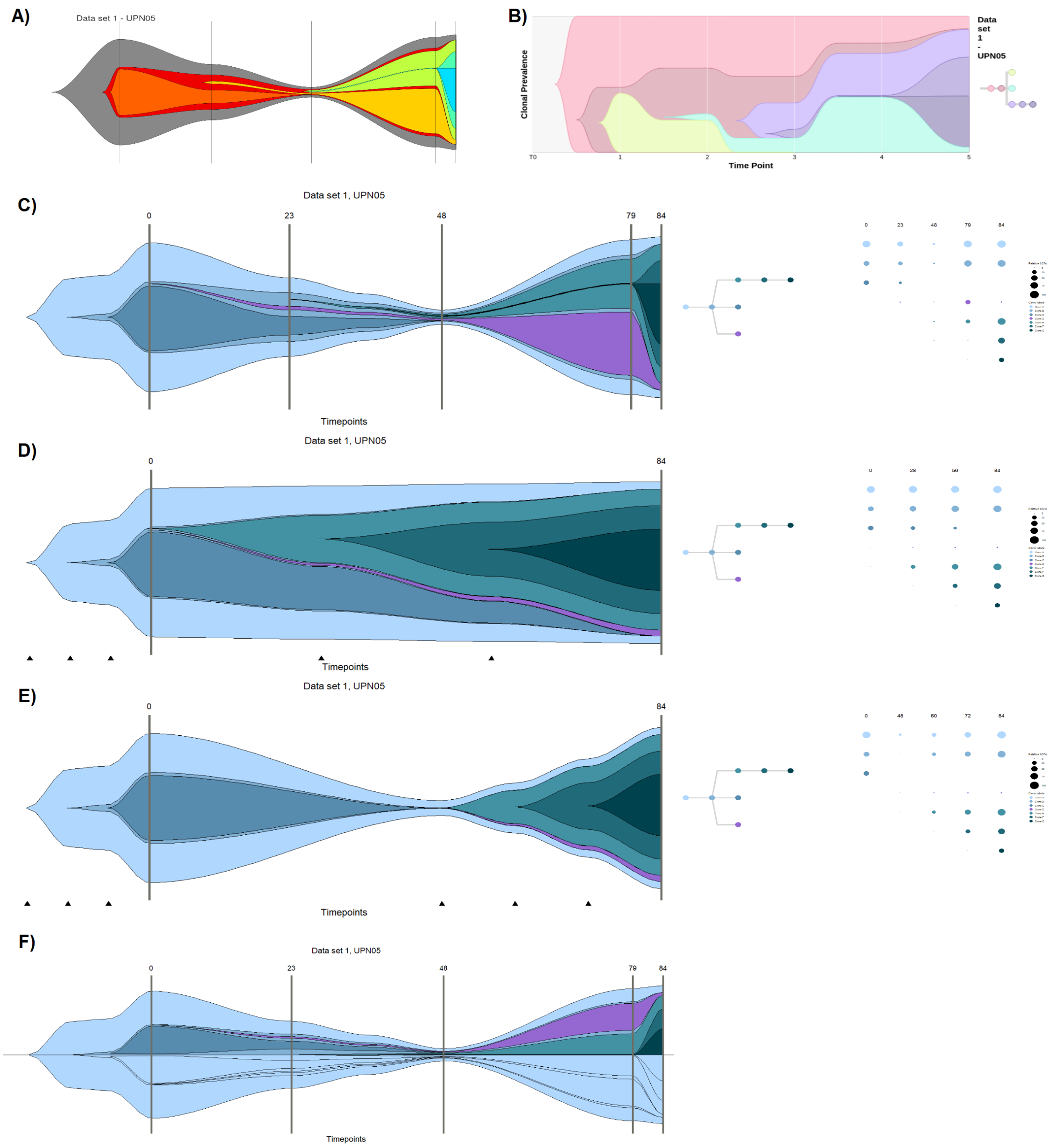

Figure S15: Visualization of clonal evolution of UPN05 in data set 1. A) Fishplot. B) Timescape. C) clevRvis using dolphin plots and extended shark plots, enabling time point interpolation for improved visualization of newly developing clones. D) clevRvis using dolphin plots and extended shark plots, enabling time point interpolation, analyzing only the first and last measured time point. E) clevRvis using dolphin plots and extended shark plots, enabling time point interpolation and therapy effect estimation, analyzing only the first and last measured time point. F) clevRvis using plaice plots, enabling time point interpolation and therapy effect estimation. A hemizygous variant affecting *BCOR* is marked. Note: Patient UPN05 is reported to have only received supportive care. The figure published by da Silva-Coelho [1] matches version D). However, reconstructing the CCF of each clone based on the precise variant calling information published by da Silva-Coelho [1] results in version C), indicating therapy effect.

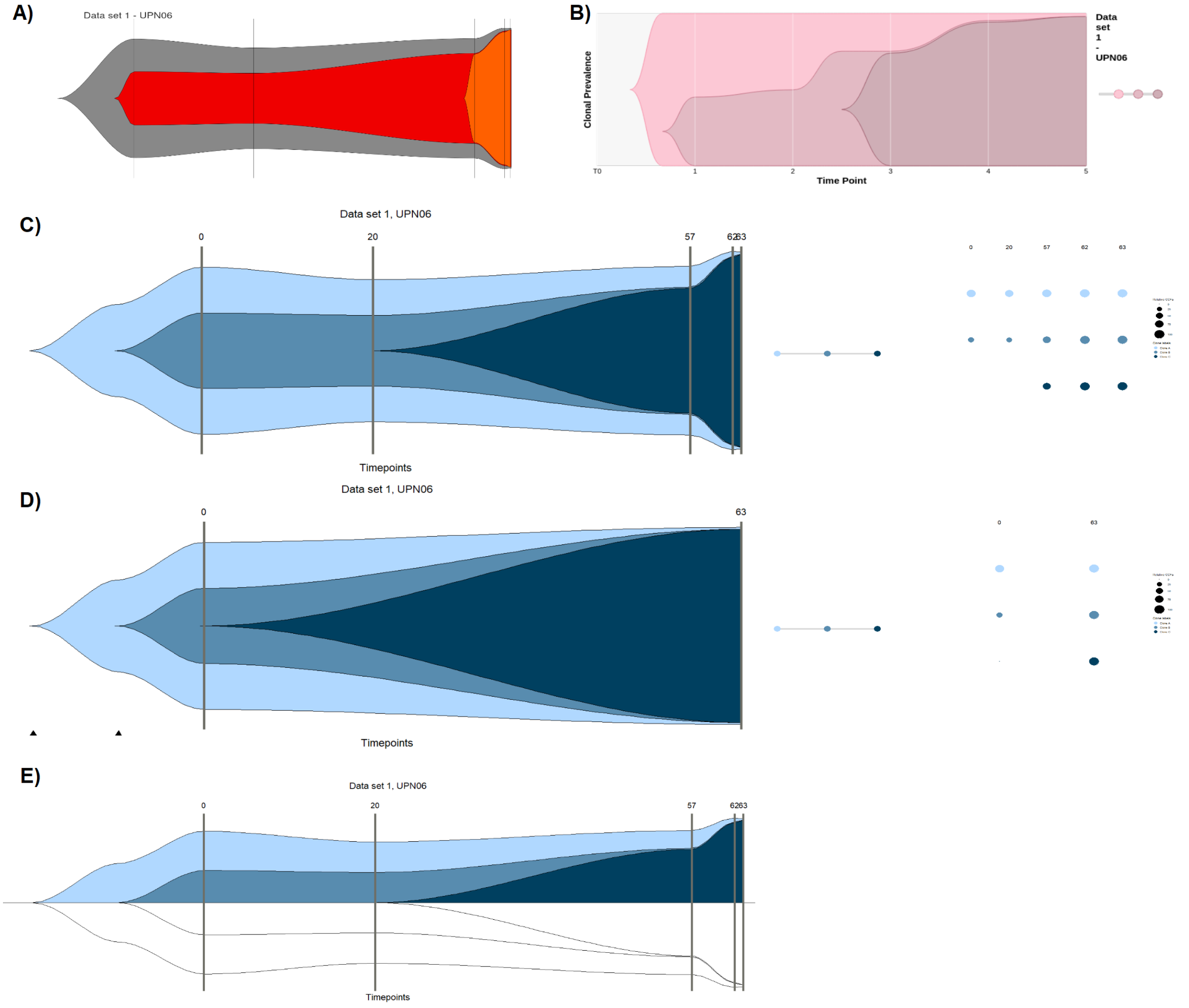

Figure S16: Visualization of clonal evolution of UPN06 in data set 1. A) Fishplot. B) Timescape. C) clevRvis using dolphin plots and extended shark plots, enabling time point interpolation for improved visualization of newly developing clones. D) clevRvis using dolphin plots and extended shark plots, enabling time point interpolation, analyzing only the first and last measured time point. E) clevRvis using plaice plots, enabling time point interpolation. A point mutation in *RUNX1* is overlapping trisomy 21, a point mutation in *UBR5* is overlapping trisomy 8. At least one healthy allele remains for both genes. Thus, no clone is marked.

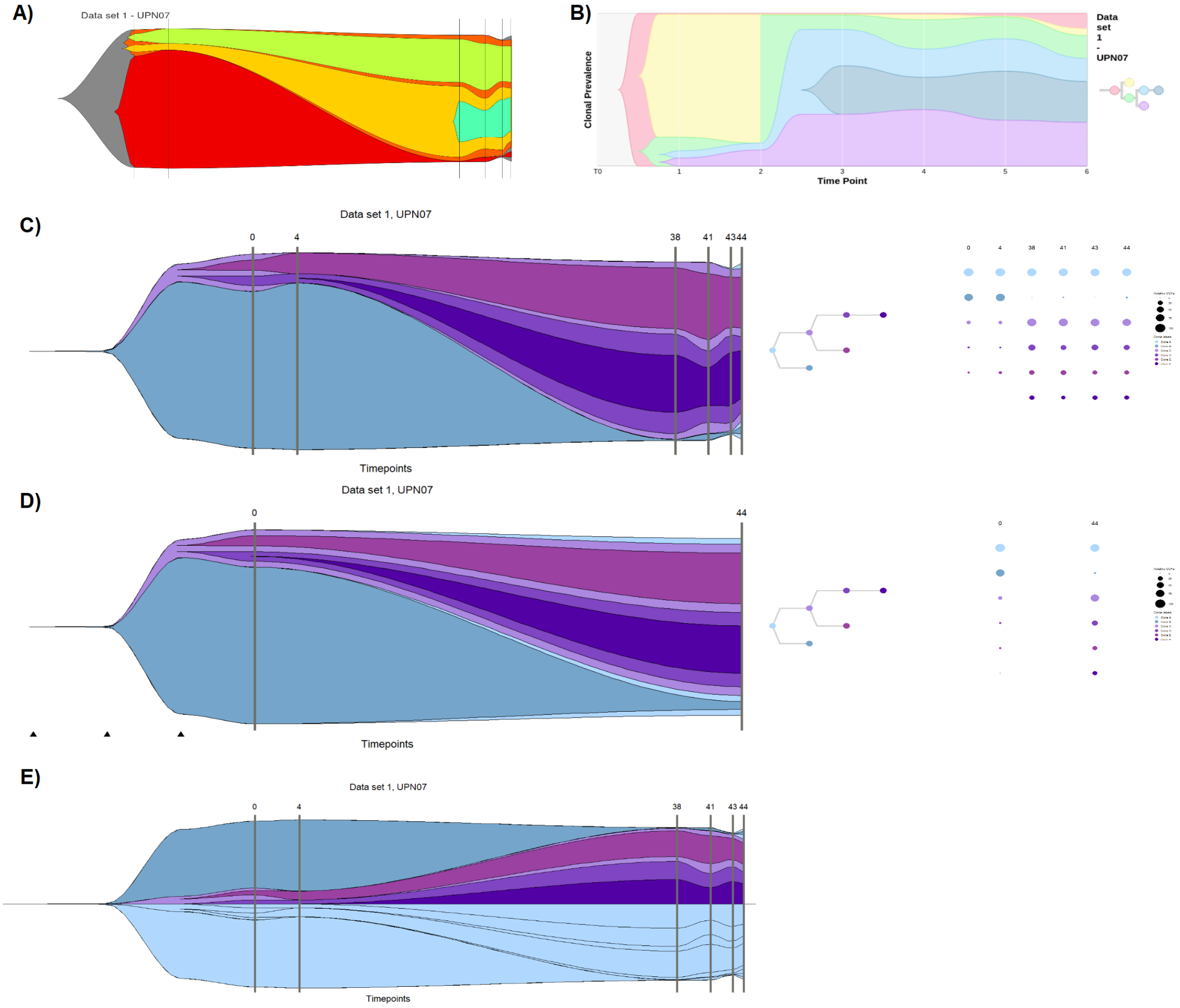

Figure S17: Visualization of clonal evolution of UPN07 in data set 1. A) Fishplot. B) Timescape. C) clevRvis using dolphin plots and extended shark plots, enabling time point interpolation for improved visualization of newly developing clones. D) clevRvis using dolphin plots and extended shark plots, enabling time point interpolation, analyzing only the first and last measured time point. E) clevRvis using plaice plots, enabling time point interpolation. A biallelic event affecting *TET2* (2 point mutations) and a hemizygous variant affecting *ZRSR2* are marked.

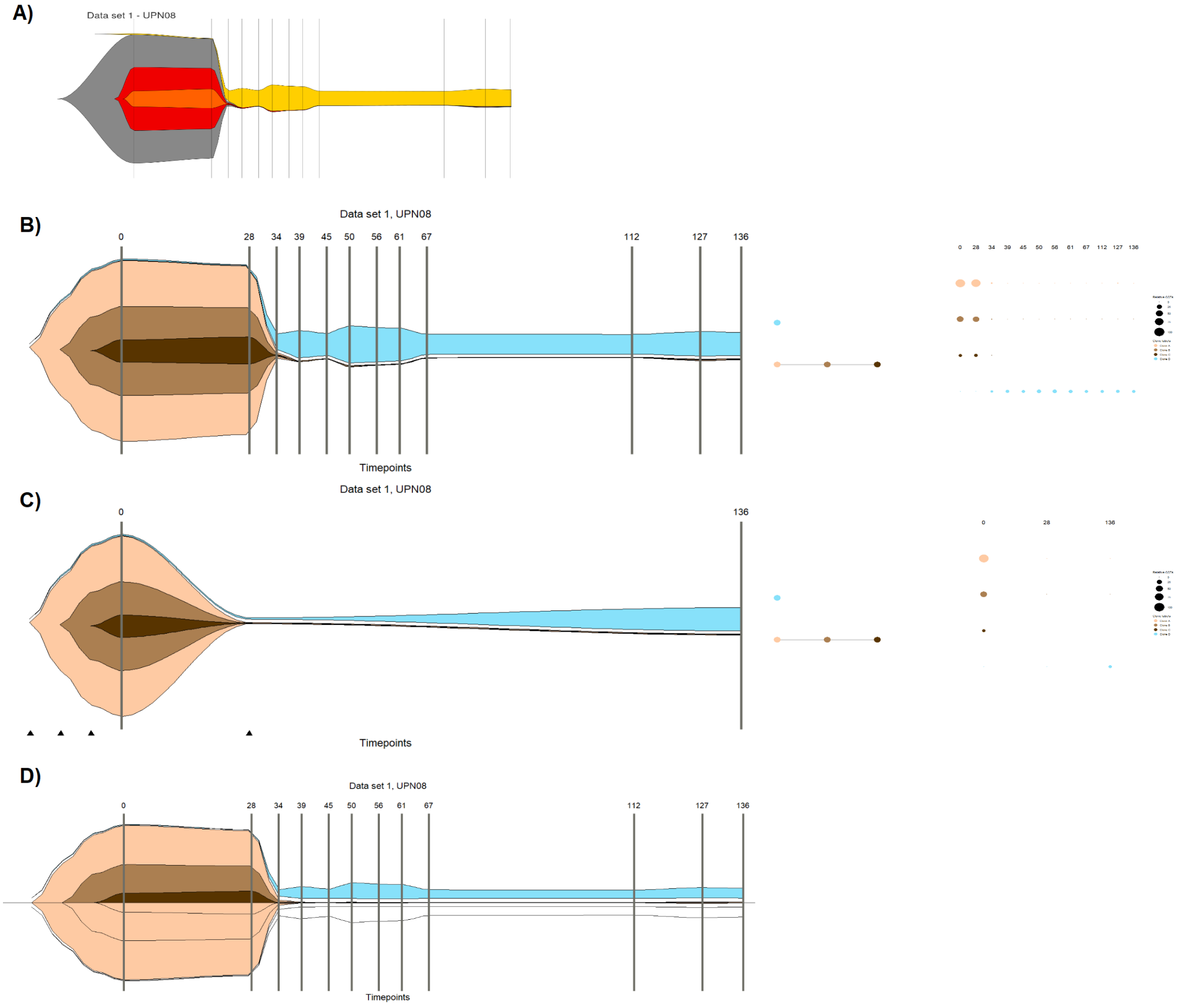

Figure S18: Visualization of clonal evolution of UPN08 in data set 1. A) Fishplot. B) clevRvis using dolphin plots and extended shark plots, enabling time point interpolation for improved visualization of newly developing clones. C) clevRvis using dolphin plots and extended shark plots, enabling time point interpolation and therapy effect estimation, analyzing only the first and last measured time point. D) clevRvis using plaice plots, enabling time point interpolation and therapy effect estimation. A bi-allelic event affecting *CSNK1A1* (del(5q) + point mutation) is marked. Note: Independent evolution cannot be visualized using timescape.

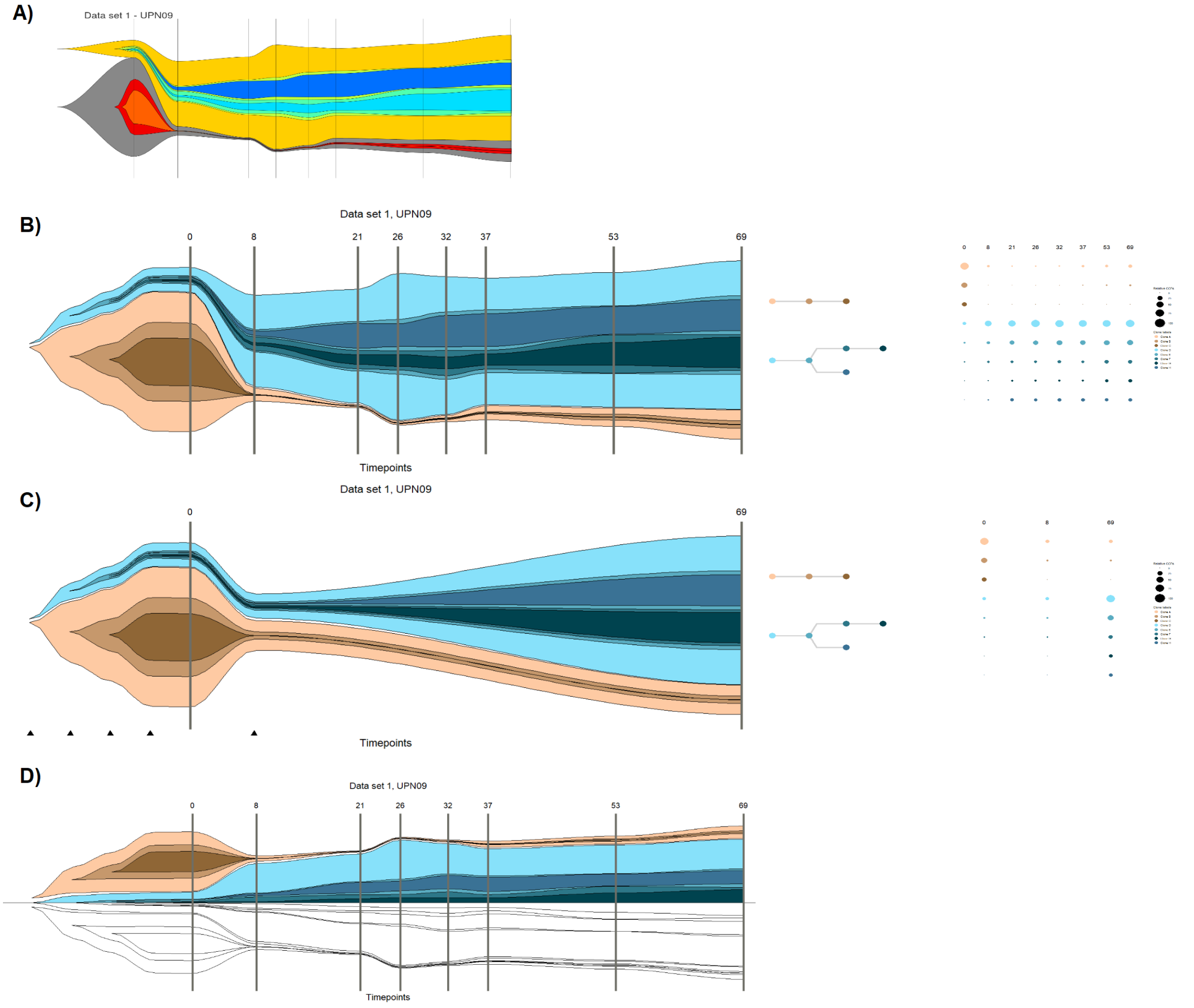

Figure S19: Visualization of clonal evolution of UPN09 in data set 1. A) Fishplot. B) clevRvis using dolphin plots and extended shark plots, enabling time point interpolation for improved visualization of newly developing clones. C) clevRvis using dolphin plots and extended shark plots, enabling time point interpolation and therapy effect estimation, analyzing only the first and last measured time point. D) clevRvis using plaice plots, enabling time point interpolation and therapy effect estimation. As no bi-allelic events are present, no clone is marked. Note: Independent evolution cannot be visualized using timescape.

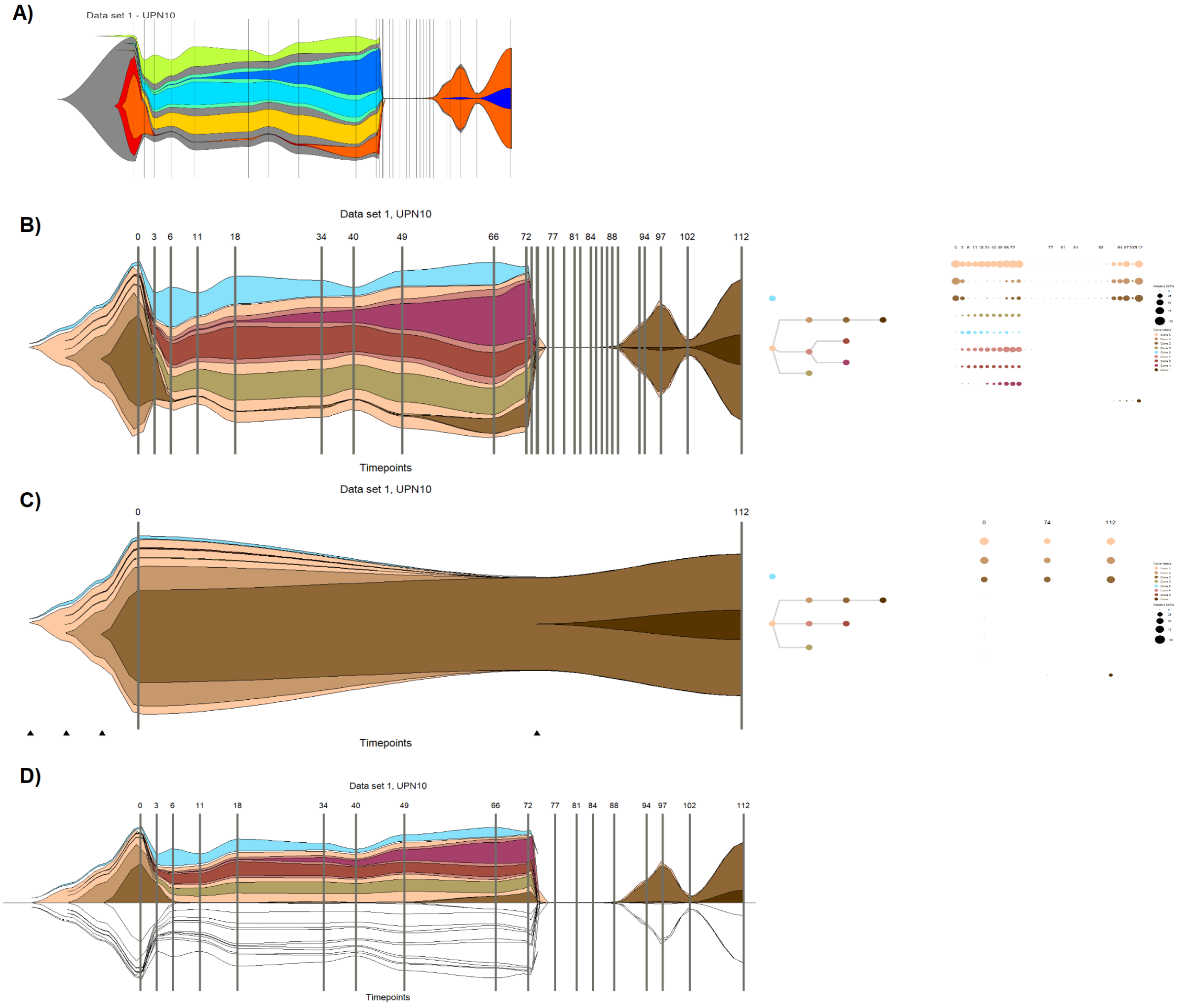

Figure S20: Visualization of clonal evolution of UPN10 in data set 1. A) Fishplot. B) clevRvis using dolphin plots and extended shark plots, enabling time point interpolation for improved visualization of newly developing clones. C) clevRvis using dolphin plots and extended shark plots, enabling time point interpolation and therapy effect estimation, analyzing only the first and last measured time point. D) clevRvis using plaice plots, enabling time point interpolation and therapy effect estimation. As no bi-allelic events are present, no clone is marked. Note: Independent evolution cannot be visualized using timescape.

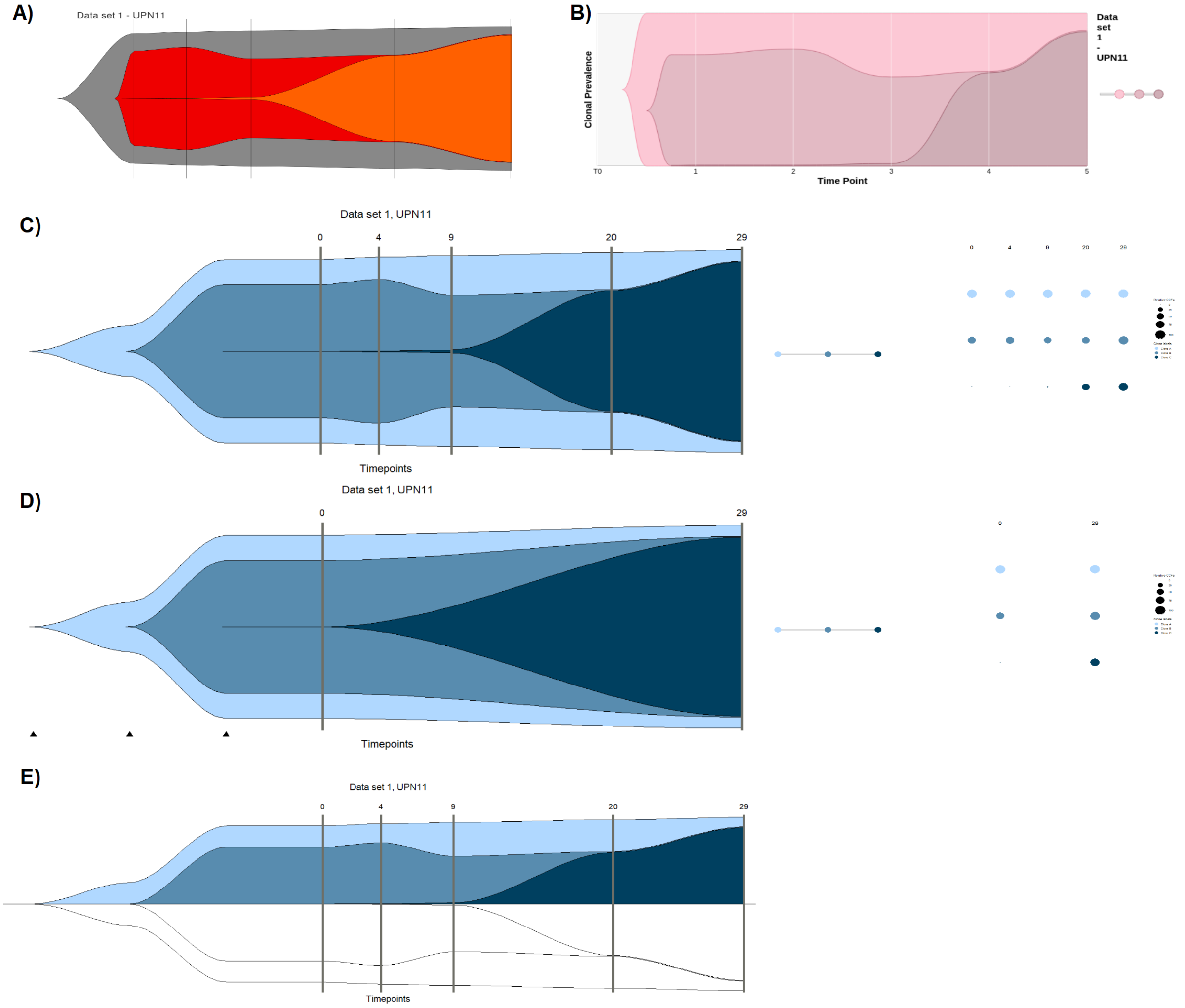

Figure S21: Visualization of clonal evolution of UPN11 in data set 1. A) Fishplot. B) Timescape. C) clevRvis using dolphin plots and extended shark plots, enabling time point interpolation for improved visualization of newly developing clones. D) clevRvis using dolphin plots and extended shark plots, enabling time point interpolation, analyzing only the first and last measured time point. E) clevRvis using plaice plots, enabling time point interpolation. A point mutation in *ARHGAP36* is overlapping dup(21q). As at least one healthy allele remains in each clone, no clone is marked.

2.2 Data set 2 – CLL

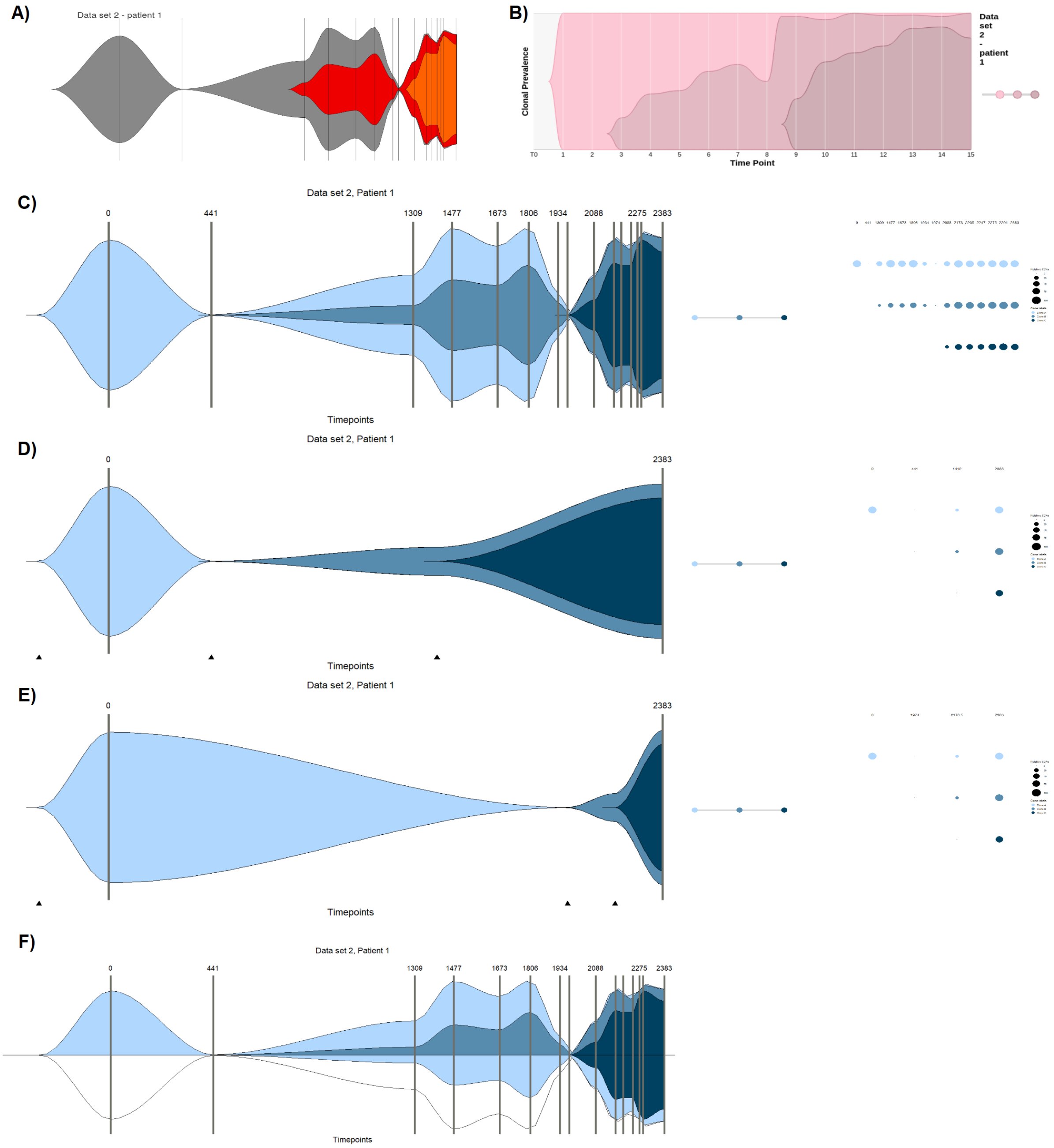

Figure S22: Visualization of clonal evolution of patient 1 in data set 2. A) Fishplot. B) Timescape. C) clevRvis using dolphin plots and extended shark plots, enabling time point interpolation for improved visualization of newly developing clones. D) clevRvis using dolphin plots and extended shark plots, enabling time point and first therapy effect estimation, analyzing only the first and last measured time point. E) clevRvis using dolphin plots and extended shark plots, enabling time point and second therapy effect estimation, analyzing only the first and last measured time point. F) clevRvis using plaice plots, enabling time point interpolation. Bi-allelic events affecting *TP53* (17p13.1 del + point mutations; light blue), *TBC1D4* (13q14.3 del + point mutation; dark blue) and a hemizygous variant on *UBA1* (point mutation; dark blue) are marked.

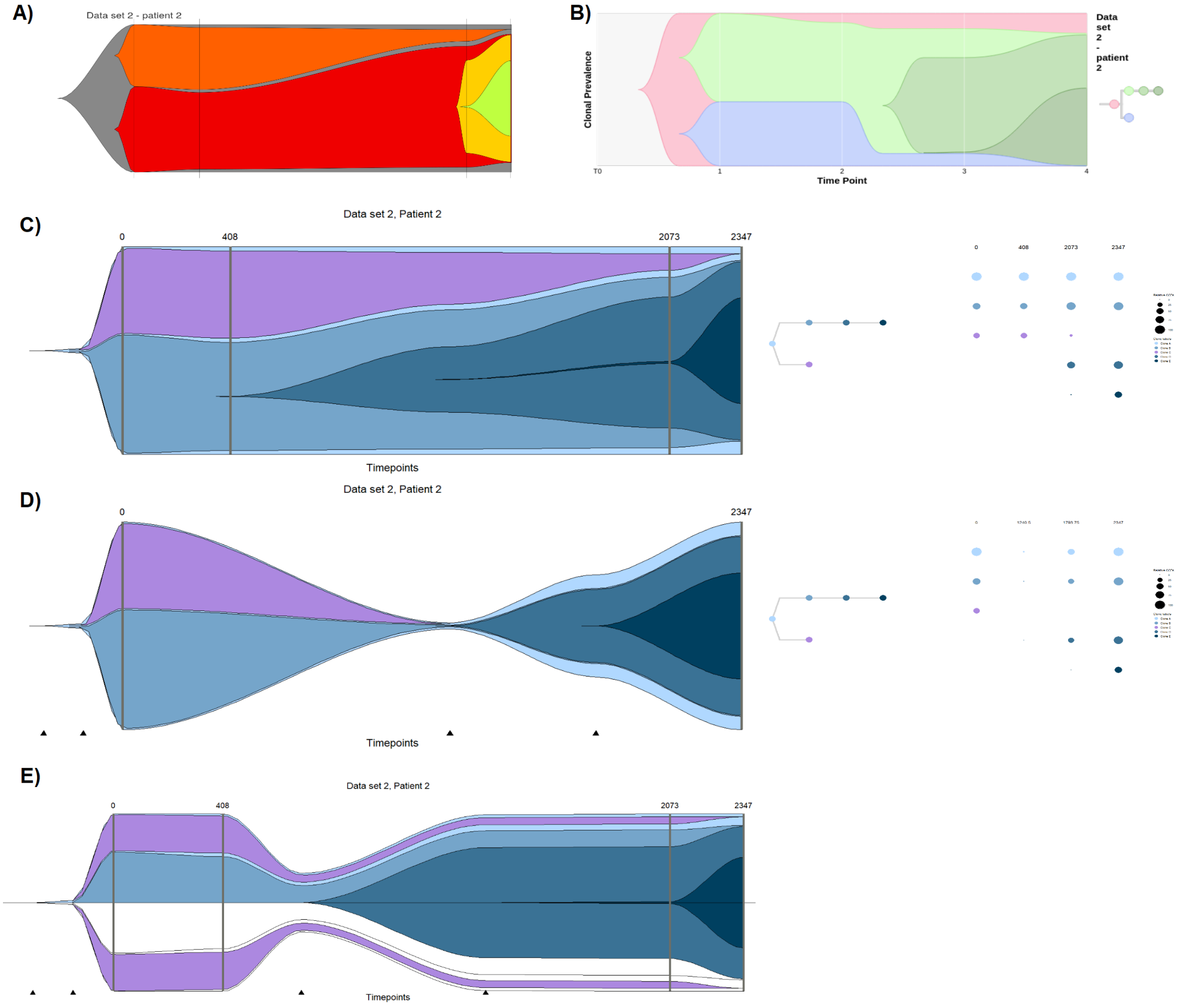

Figure S23: Visualization of clonal evolution of patient 2 in data set 2. A) Fishplot. B) Timescape. C) clevRvis using dolphin plots and extended shark plots, enabling time point interpolation for improved visualization of newly developing clones. D) clevRvis using dolphin plots and extended shark plots, enabling time point interpolation and therapy effect estimation, analyzing only the first and last measured time point. E) clevRvis using plaice plots, enabling time point interpolation and therapy effect estimation. Bi-allelic events affecting *TP53* (17p13.1 del + point mutations; intermediate blue) and *GPS2* (17p13.1 del + point mutations; dark blue) are marked. Additionally, hemizygous variants on *MED12* (purple), *ZCCHC16* (intermediate blue) and *DDX3X* (dark blue) are marked.

2.3 Data set 3 – MPN

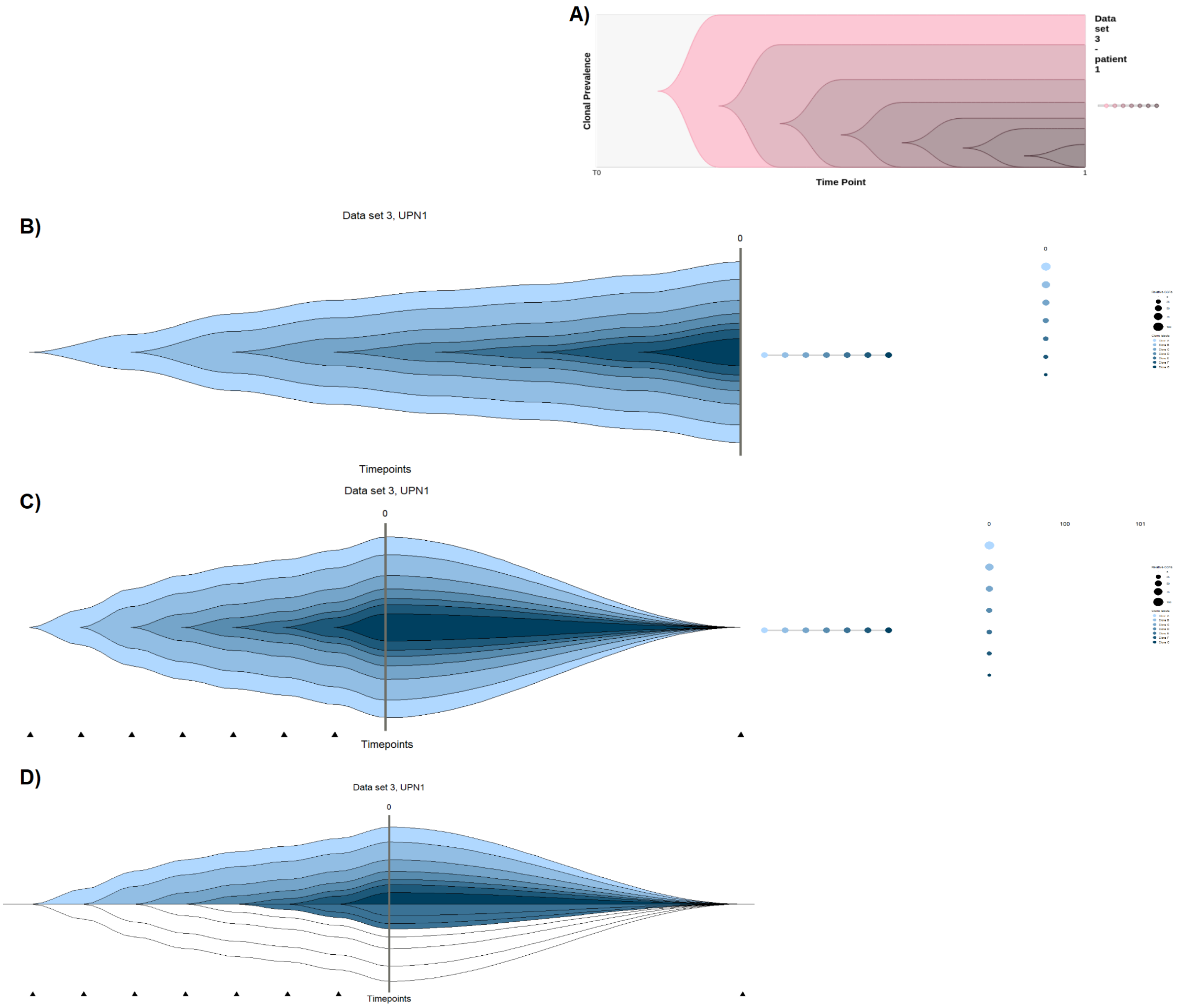

Figure S24: Visualization of clonal evolution of UPN1 in data set 3. A) Timescape. B) clevRvis using dolphin plots and extended shark plots, enabling time point interpolation for improved visualization of newly developing clones. C) clevRvis using dolphin plots and extended shark plots, enabling time point interpolation and therapy effect estimation. D) clevRvis using plaice plots, enabling time point interpolation and therapy effect estimation. A bi-allelic event affecting *TP53* (deletion chromosome 17 + point mutation in *TP53*) is marked. Note: A single time point cannot be visualized using fishplot.

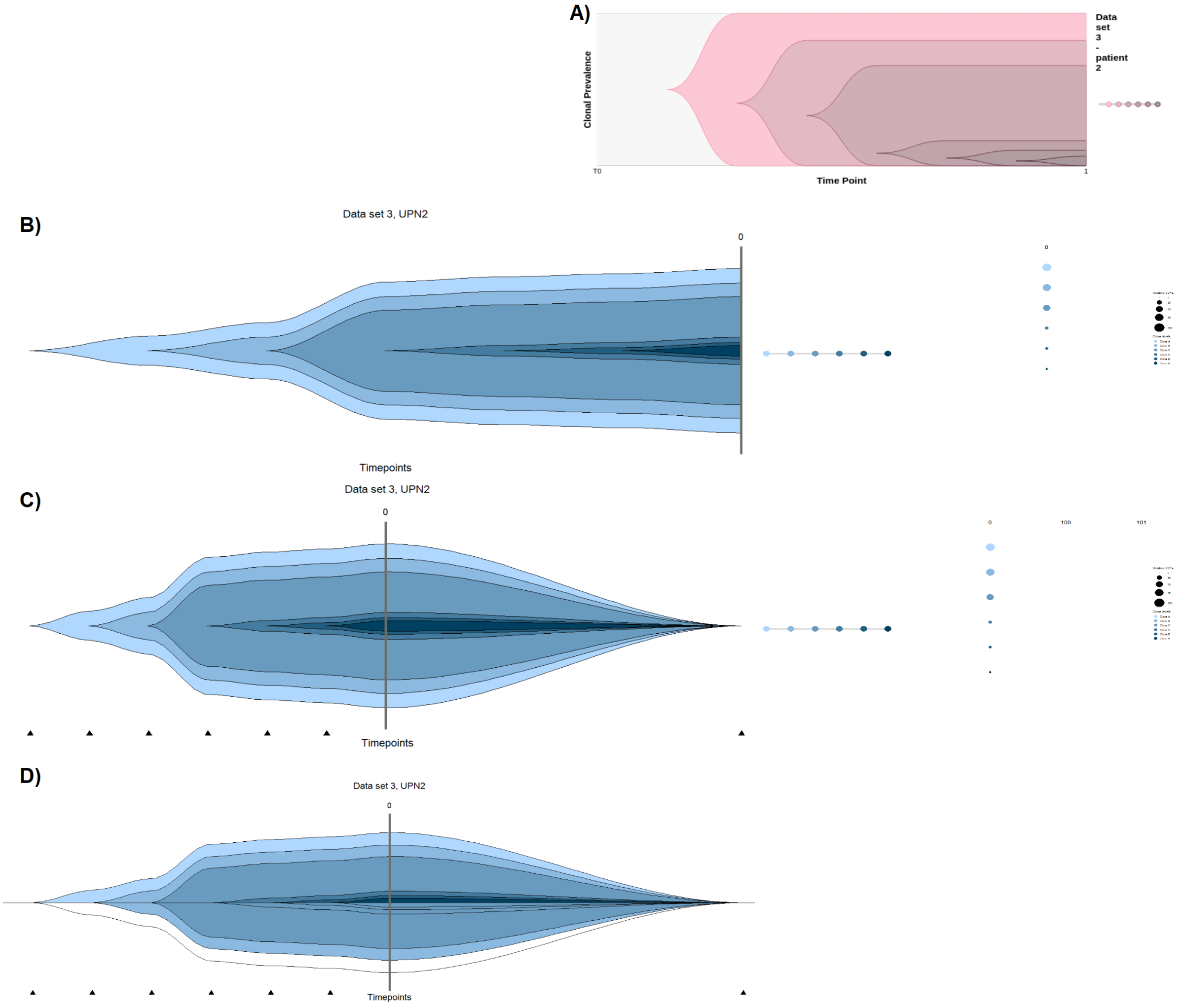

Figure S25: Visualization of clonal evolution of UPN2 in data set 3. A) Timescape. B) clevRvis using dolphin plots and extended shark plots, enabling time point interpolation for improved visualization of newly developing clones. C) clevRvis using dolphin plots and extended shark plots, enabling time point interpolation and therapy effect estimation. D) clevRvis using plaice plots, enabling time point interpolation and therapy effect estimation. Hemizygous variants affecting *BCOR* and *STAG2*, leading to a loss of the only available healthy allele, are marked. Note: A single time point cannot be visualized using fishplot.

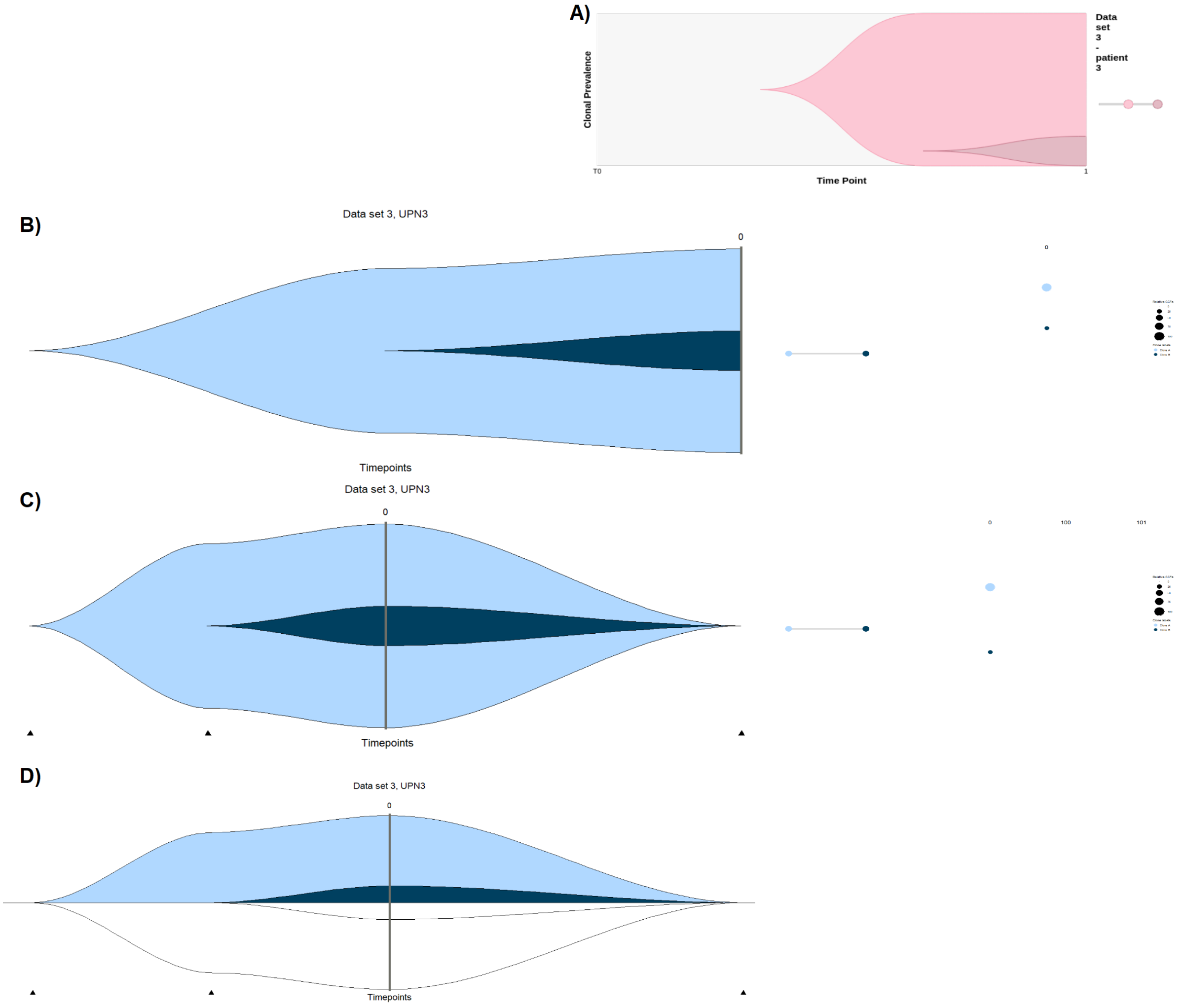

Figure S26: Visualization of clonal evolution of UPN3 in data set 3. A) Timescape. B) clevRvis using dolphin plots and extended shark plots, enabling time point interpolation for improved visualization of newly developing clones. C) clevRvis using dolphin plots and extended shark plots, enabling time point interpolation and therapy effect estimation. D) clevRvis using plaice plots, enabling time point interpolation. As all clones feature at least one healthy allele of every gene, no clone is marked. Note: A single time point cannot be visualized using fishplot.

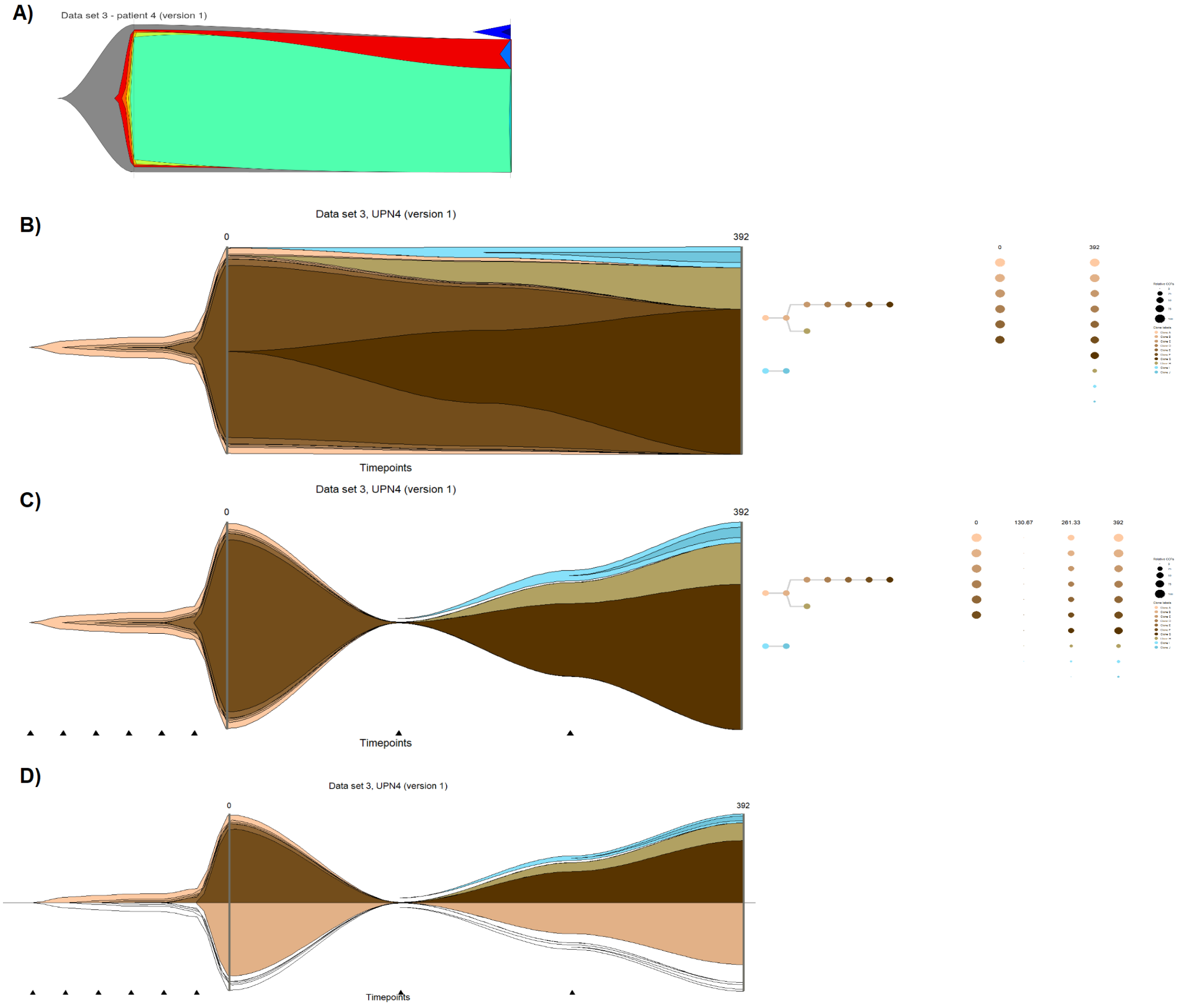

Figure S27: Visualization of clonal evolution of UPN4 (version 1) in data set 3. A) Fishplot. B) clevRvis using dolphin plots and extended shark plots, enabling time point interpolation for improved visualization of newly developing clones. C) clevRvis using dolphin plots and extended shark plots, enabling time point interpolation and therapy effect estimation. D) clevRvis using plaice plots, enabling time point interpolation and therapy effect estimation. A bi-allelic event affecting *TET2* (2 point mutations) is marked. Note: Independent evolution cannot be visualized using timescape.

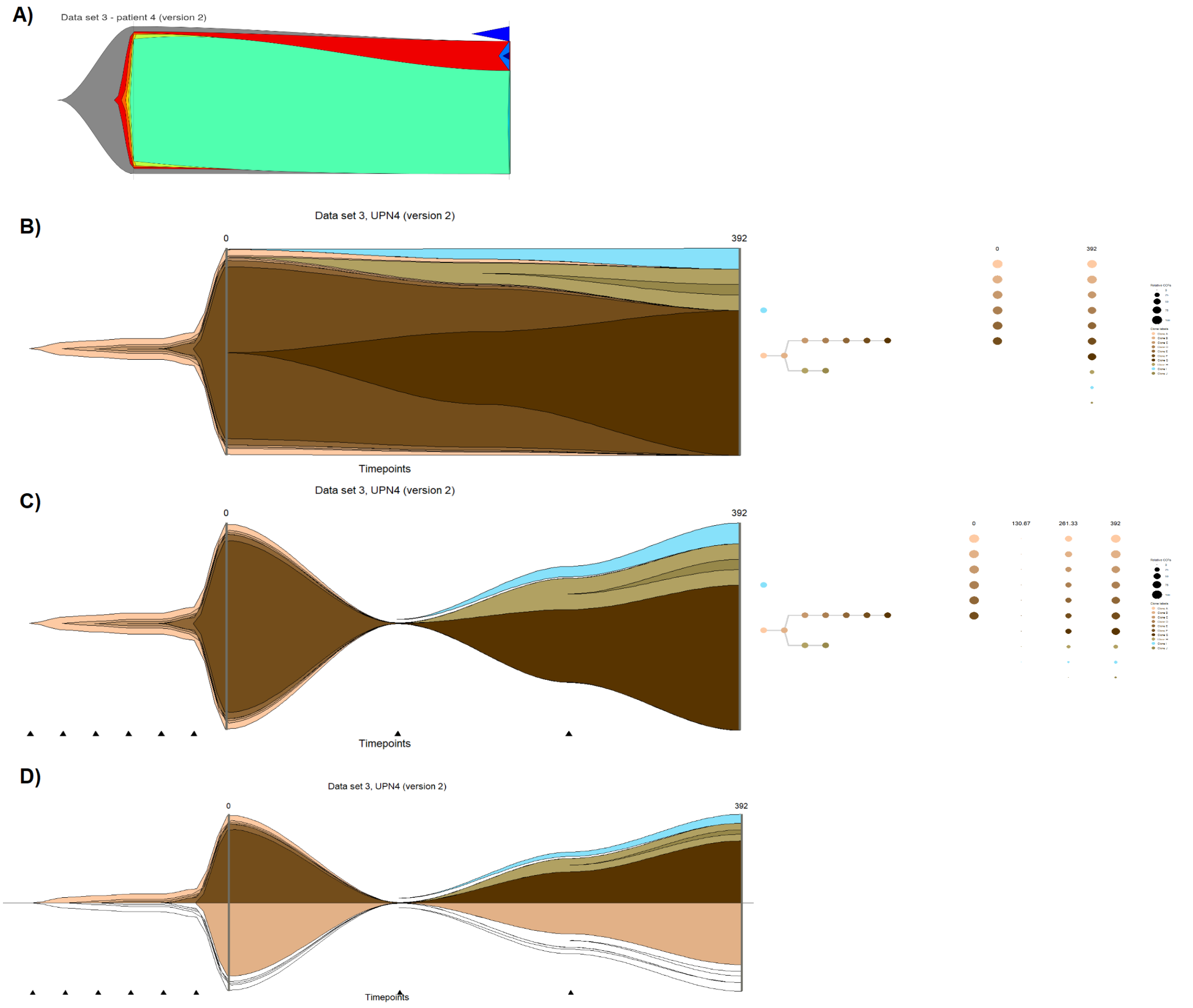

Figure S28: Visualization of clonal evolution of UPN4 (version 2) in data set 3. A) Fishplot. B) clevRvis using dolphin plots and extended shark plots, enabling time point interpolation for improved visualization of newly developing clones. C) clevRvis using dolphin plots and extended shark plots, enabling time point interpolation and therapy effect estimation. D) clevRvis using plaice plots, enabling time point interpolation and therapy effect estimation. A bi-allelic event affecting *TET2* (2 point mutations) is marked. Note: Independent evolution cannot be visualized using timescape.

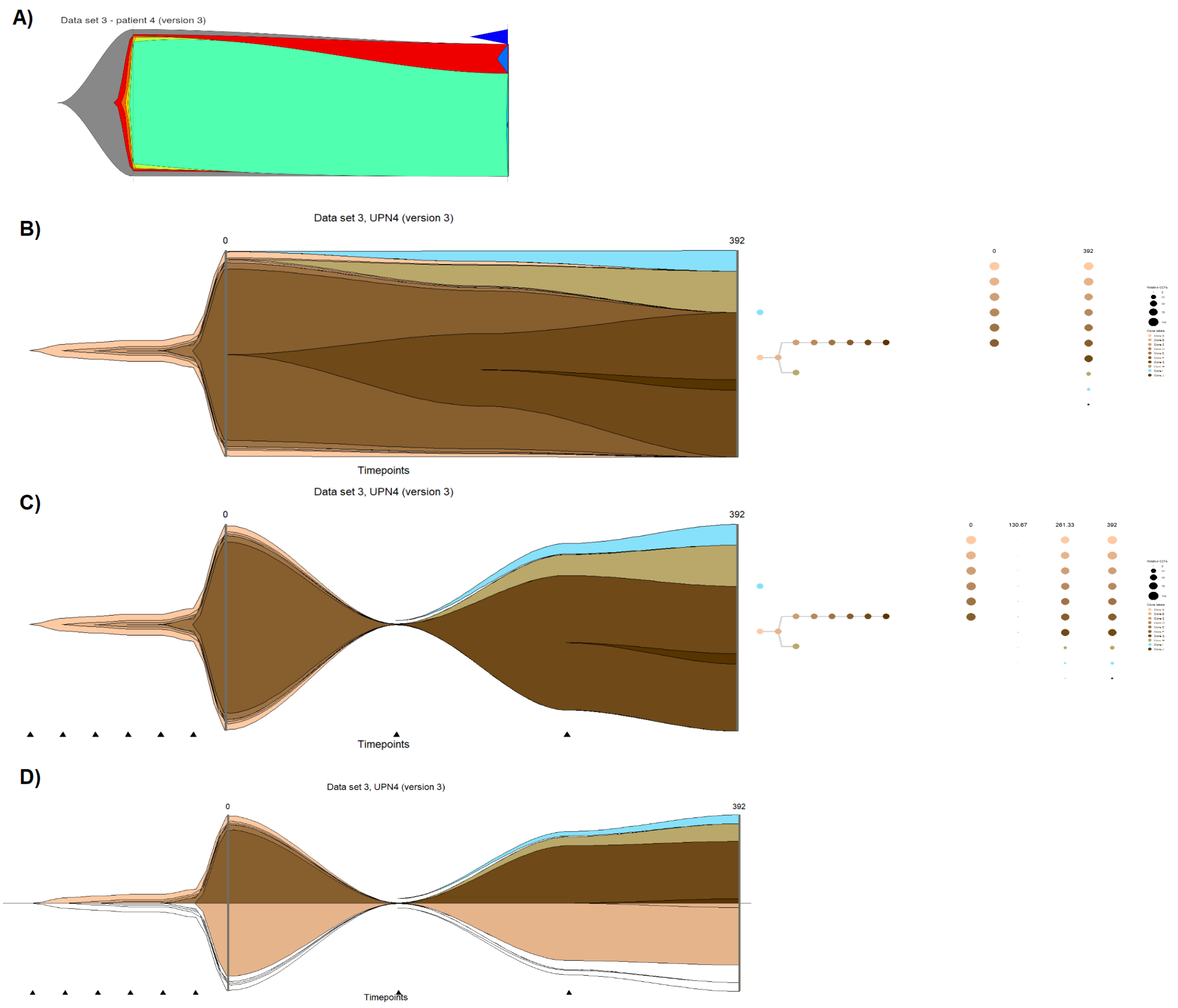

Figure S29: Visualization of clonal evolution of UPN4 (version 3) in data set 3. A) Fishplot. B) clevRvis using dolphin plots and extended shark plots, enabling time point interpolation for improved visualization of newly developing clones. C) clevRvis using dolphin plots and extended shark plots, enabling time point interpolation and therapy effect estimation. D) clevRvis using plaice plots, enabling time point interpolation and therapy effect estimation. A bi-allelic event affecting *TET2* (2 point mutations) is marked. Note: Independent evolution cannot be visualized using timescape.

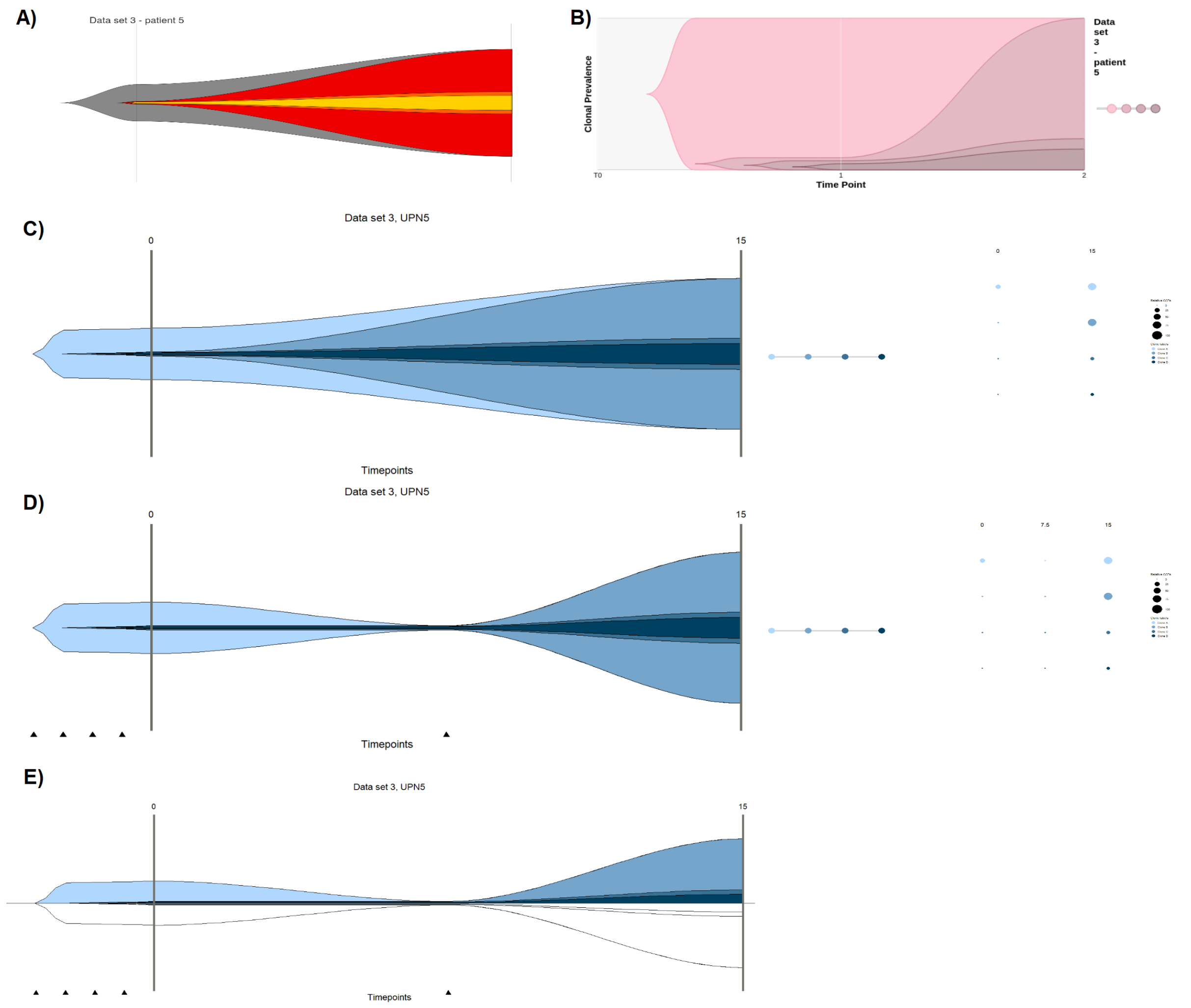

Figure S30: Visualization of clonal evolution of UPN5 in data set 3. A) Fishplot. B) Timescape. C) clevRvis using dolphin plots and extended shark plots, enabling time point interpolation for improved visualization of newly developing clones. D) clevRvis using dolphin plots and extended shark plots, enabling time point interpolation and therapy effect estimation. E) clevRvis using plaice plots, enabling time point interpolation and therapy effect estimation. As all clones feature at least one healthy allele of every gene, no clone is marked.

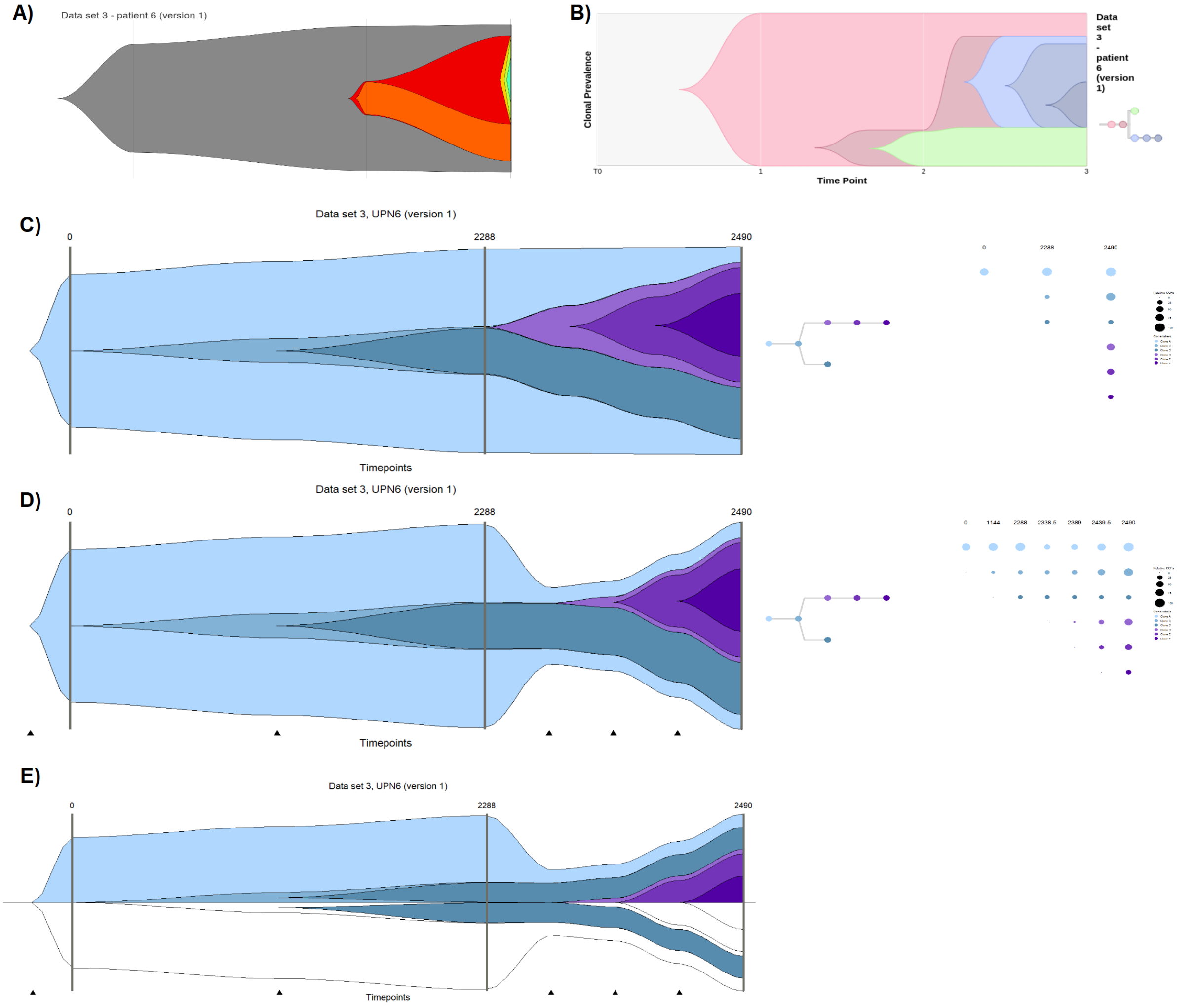

Figure S31: Visualization of clonal evolution of UPN6 (version 1) in data set 3. A) Fishplot. B) Timescape. C) clevRvis using dolphin plots and extended shark plots, enabling time point interpolation for improved visualization of newly developing clones. D) clevRvis using dolphin plots and extended shark plots, enabling time point interpolation and therapy effect estimation. E) clevRvis using plaice plots, enabling time point interpolation and therapy effect estimation. A bi-allelic event affecting *TP53* (derivative chromosome 17 der(17)t(13;17)(q21;p12) + point mutation in *TP53*) is marked.

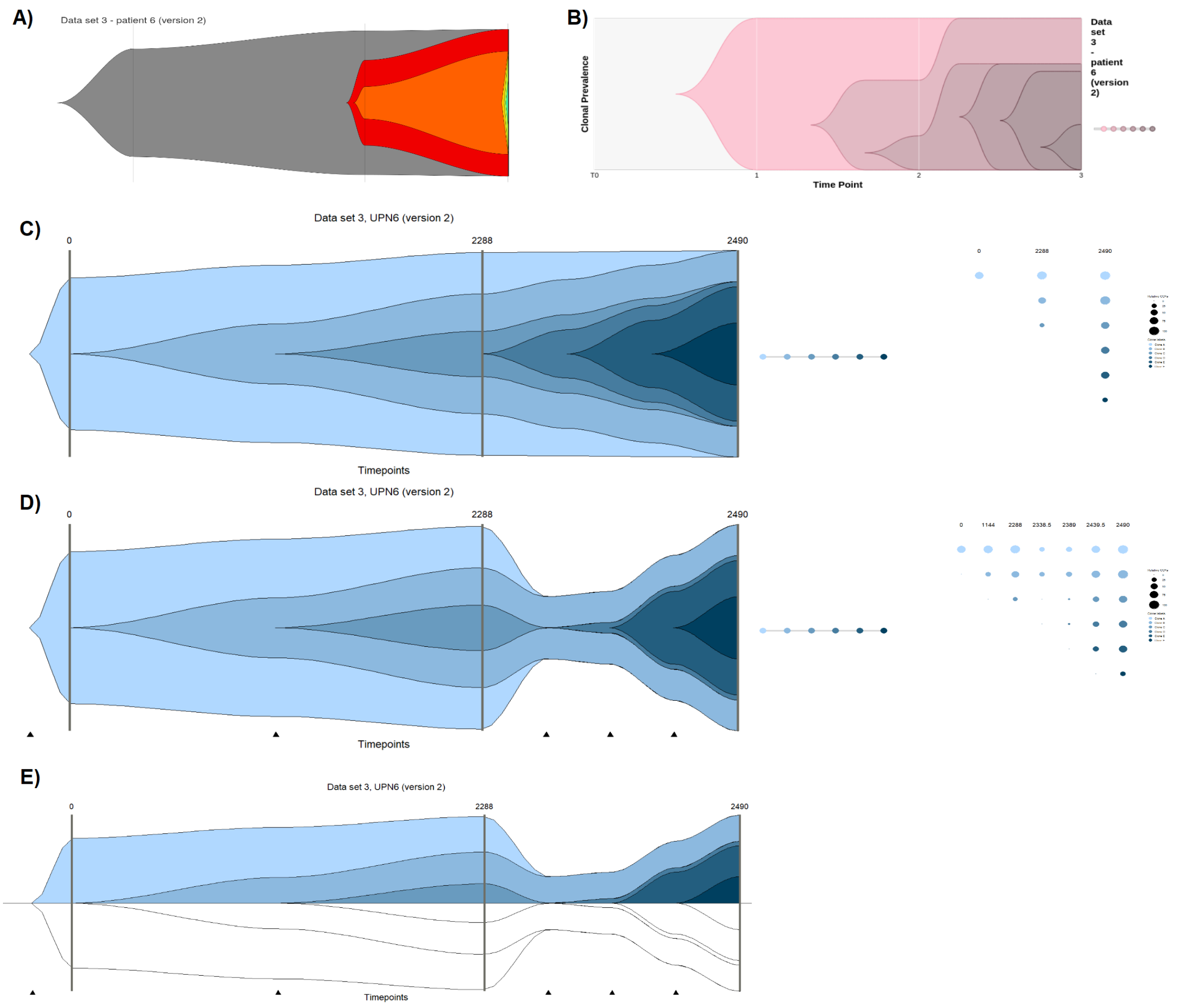

Figure S32: Visualization of clonal evolution of UPN6 (version 2) in data set 3. A) Fishplot. B) Timescape. C) clevRvis using dolphin plots and extended shark plots, enabling time point interpolation for improved visualization of newly developing clones. D) clevRvis using dolphin plots and extended shark plots, enabling time point interpolation and therapy effect estimation. E) clevRvis using plaice plots, enabling time point interpolation and therapy effect estimation. It is assumed that der(17)t(13;17)(q21;p12) affects the allele already affected by a point mutation in *TP53*. Thus, a healthy copy of the gene remains and no clone is marked.

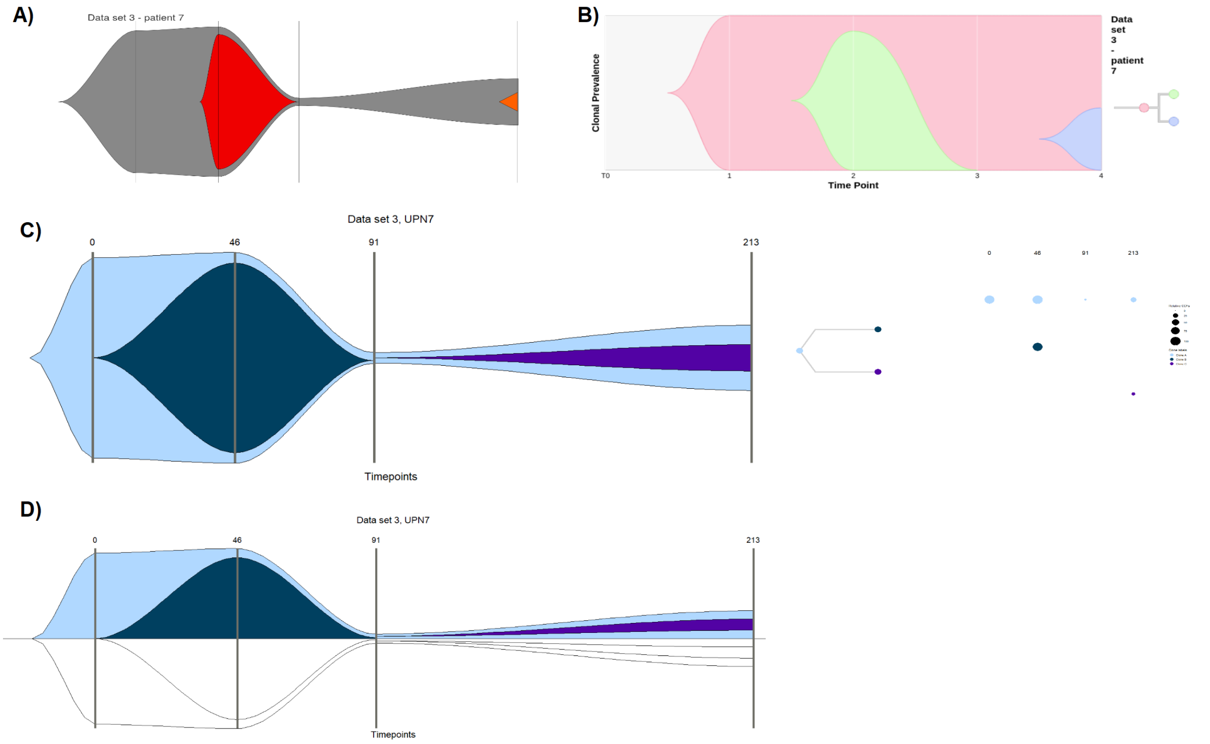

Figure S33: Visualization of clonal evolution of UPN7 in data set 3. A) Fishplot. B) Timescape. C) clevRvis using dolphin plots and extended shark plots, enabling time point interpolation for improved visualization of newly developing clones. D) clevRvis using plaice plots, enabling time point interpolation. As all clones feature at least one healthy allele of every gene, no clone is marked.

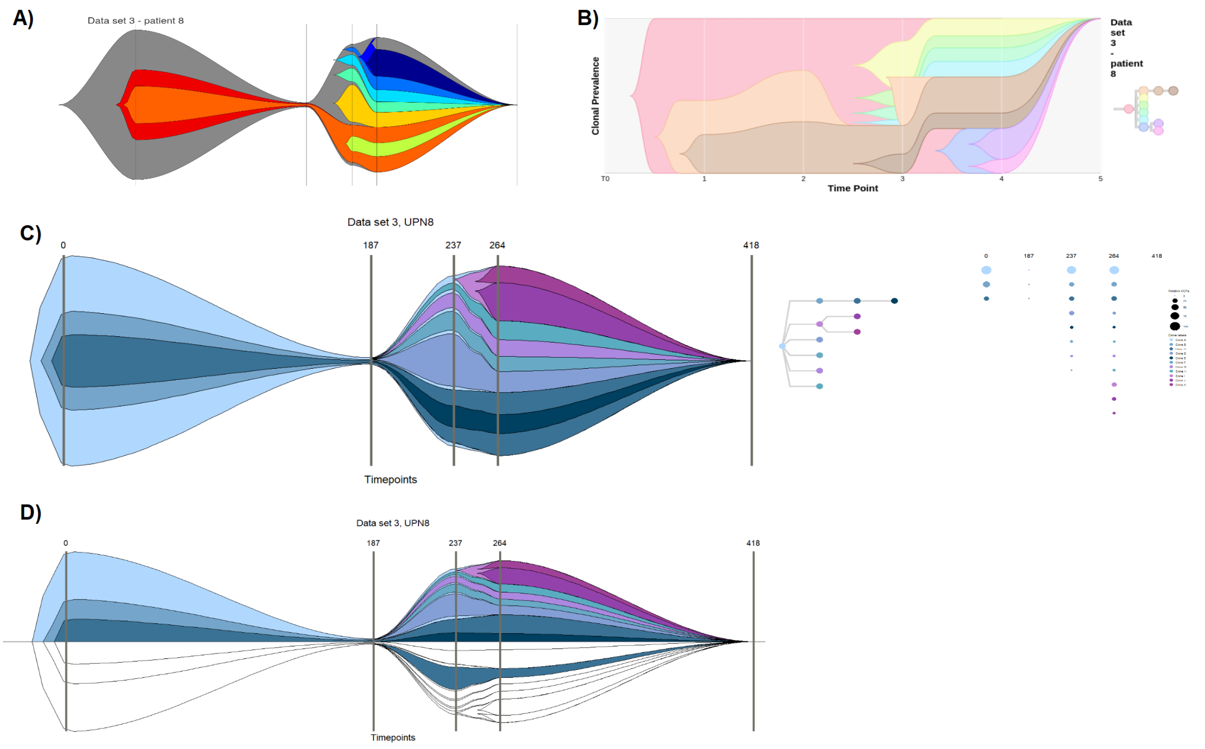

Figure S34: Visualization of clonal evolution of UPN8 in data set 3. A) Fishplot. B) Timescape. C) clevRvis using dolphin plots and extended shark plots, enabling time point interpolation for improved visualization of newly developing clones. D) clevRvis using plaice plots, enabling time point interpolation and therapy effect estimation. A bi-allelic event affecting *NRAS* (2 point mutations) is marked.

2.4 Data set 4 – BL

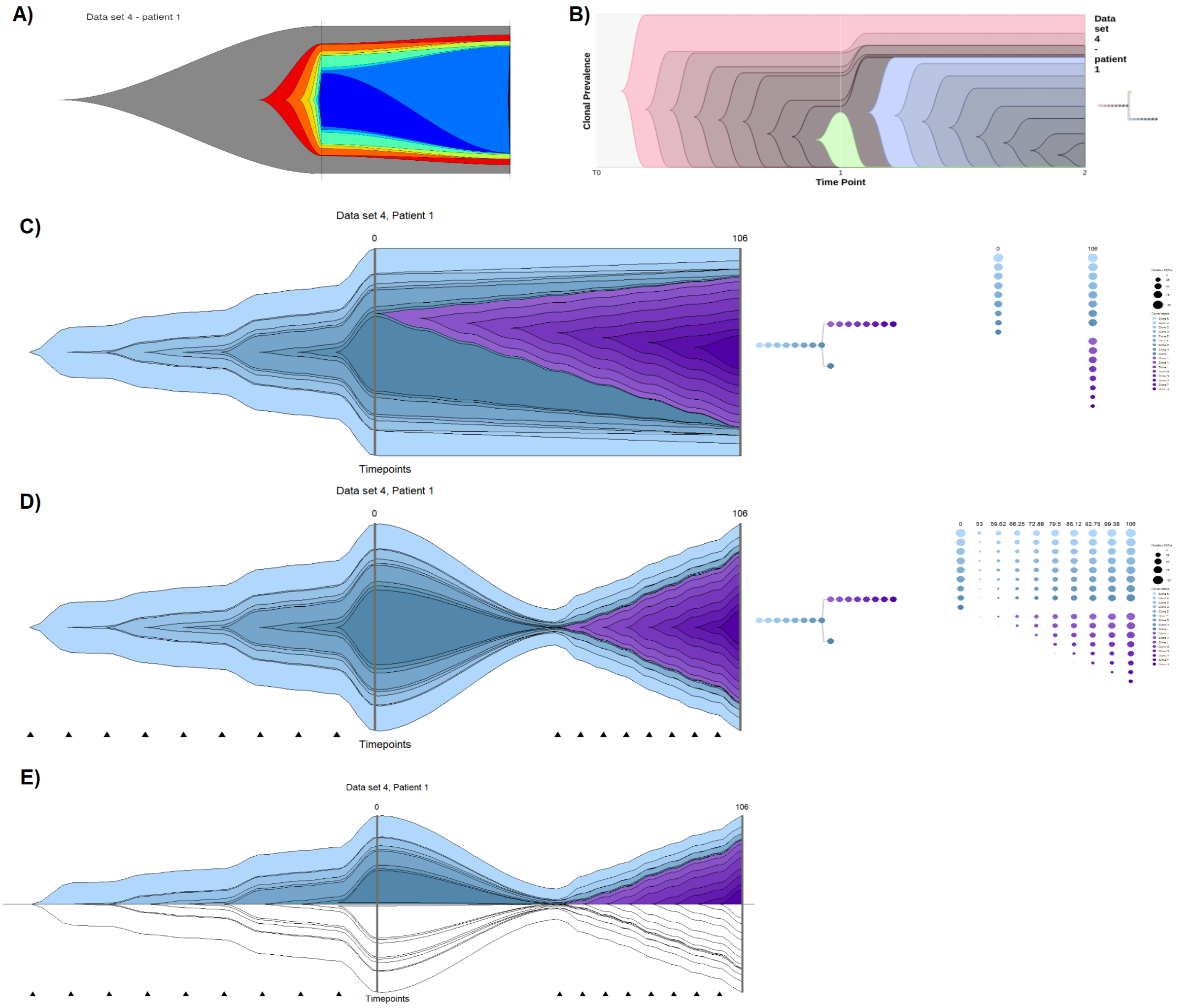

Figure S35: Visualization of clonal evolution of patient 1 in data set 4. A) Fishplot. B) Timescape. C) clevRvis using dolphin plots and extended shark plots, enabling time point interpolation for improved visualization of newly developing clones. D) clevRvis using dolphin plots and extended shark plots, enabling time point interpolation and therapy effect estimation. E) clevRvis using plaice plots (only focusing on *TP53*), enabling time point interpolation and therapy effect estimation. Two variants affect *TP53* (point mutation + duplication). However, one healthy copy of the gene remains. Thus, no clone is marked.

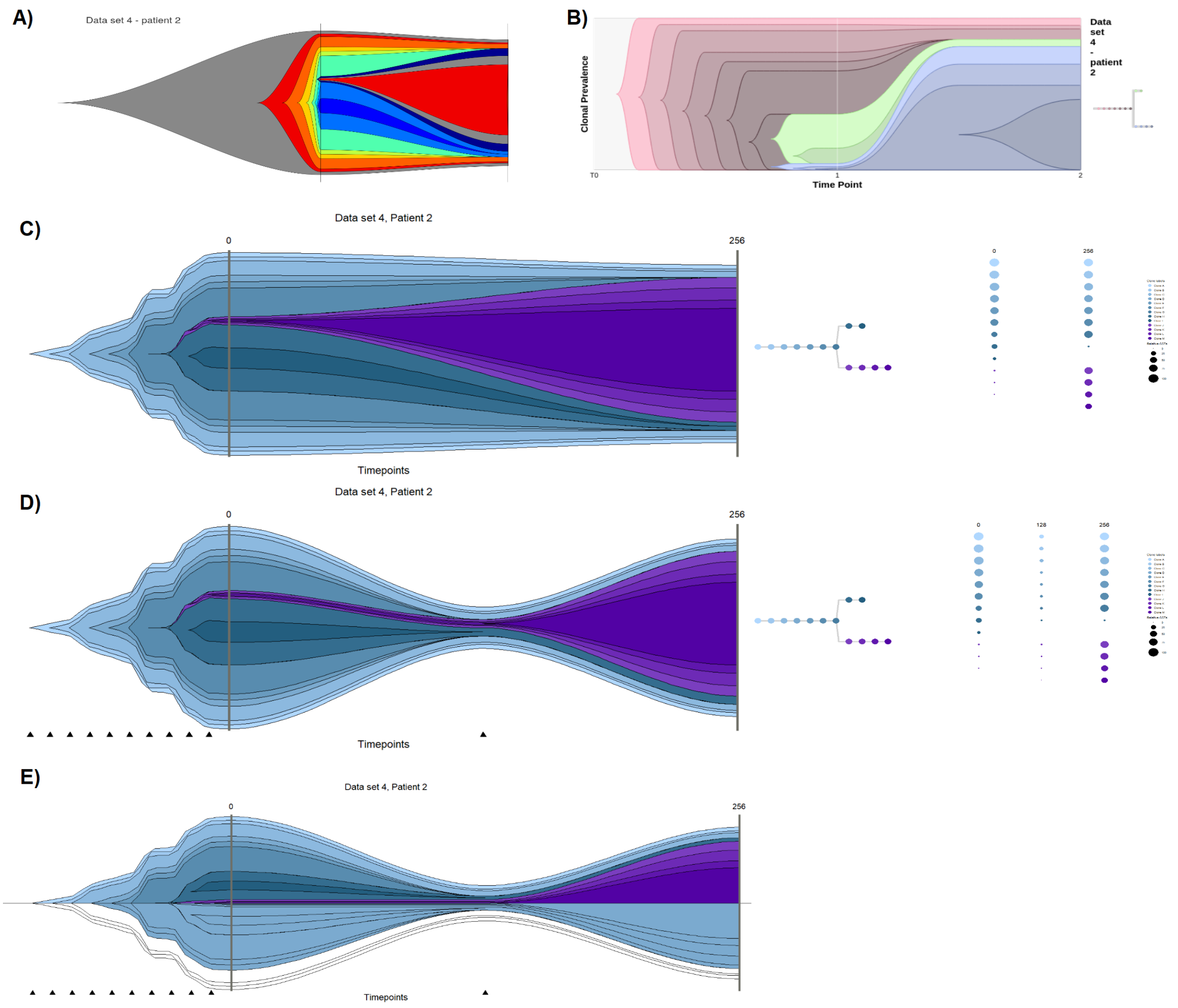

Figure S36: Visualization of clonal evolution of patient 2 in data set 4. A) Fishplot. B) Timescape. C) clevRvis using dolphin plots and extended shark plots, enabling time point interpolation for improved visualization of newly developing clones. D) clevRvis using dolphin plots and extended shark plots, enabling time point interpolation and therapy effect estimation. E) clevRvis using plaice plots (only focusing on *TP53*), enabling time point interpolation and therapy effect estimation. A bi-allelic event affecting *TP53* (2 point mutations) is marked.

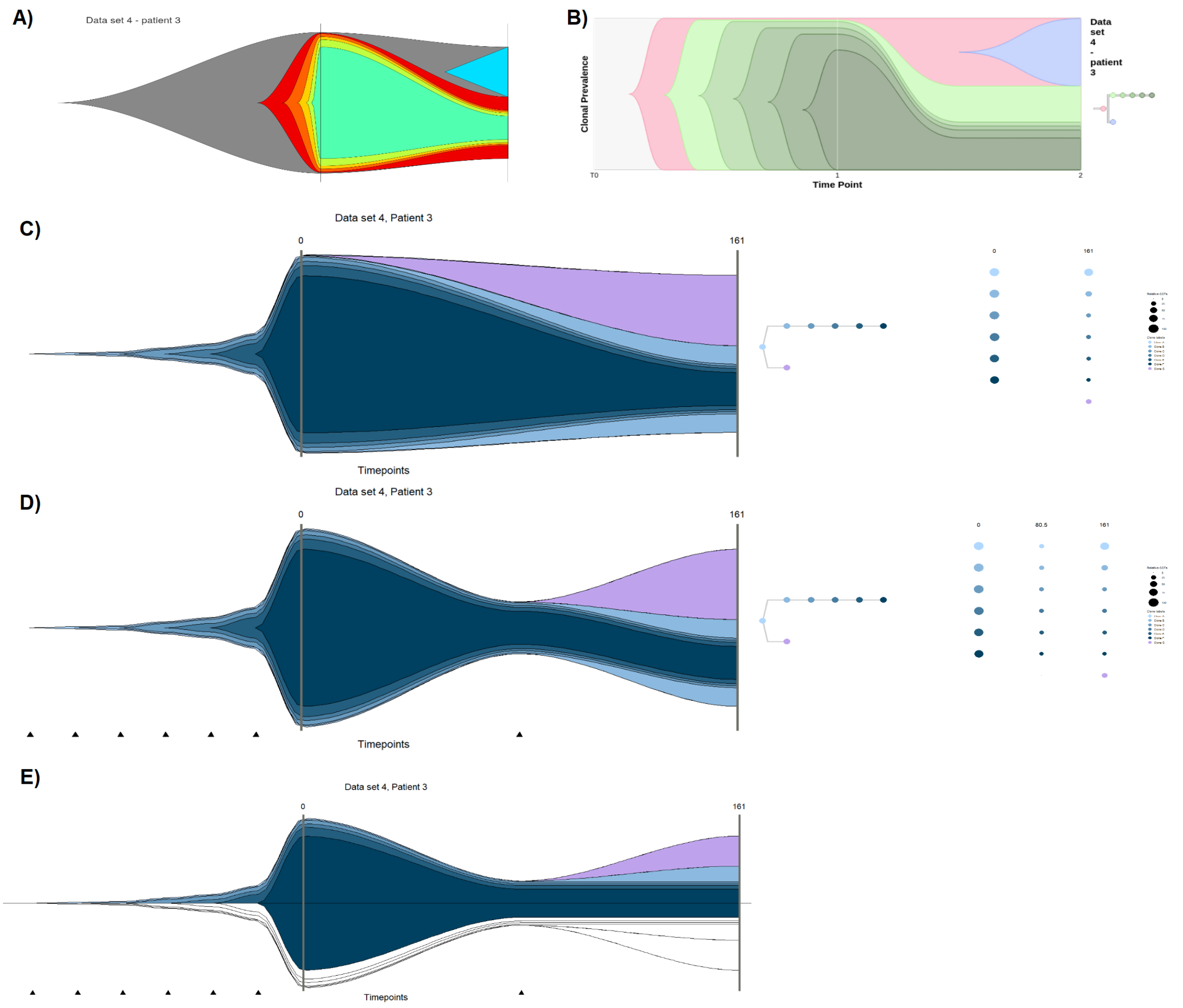

Figure S37: Visualization of clonal evolution of patient 3 in data set 4. A) Fishplot. B) Timescape. C) clevRvis using dolphin plots and extended shark plots, enabling time point interpolation for improved visualization of newly developing clones. D) clevRvis using dolphin plots and extended shark plots, enabling time point interpolation and therapy effect estimation. E) clevRvis using plaice plots (only focusing on *TP53*), enabling time point interpolation and therapy effect estimation. A bi-allelic event affecting *TP53* (loss of heterozygosity + point mutation in *TP53*) is marked.

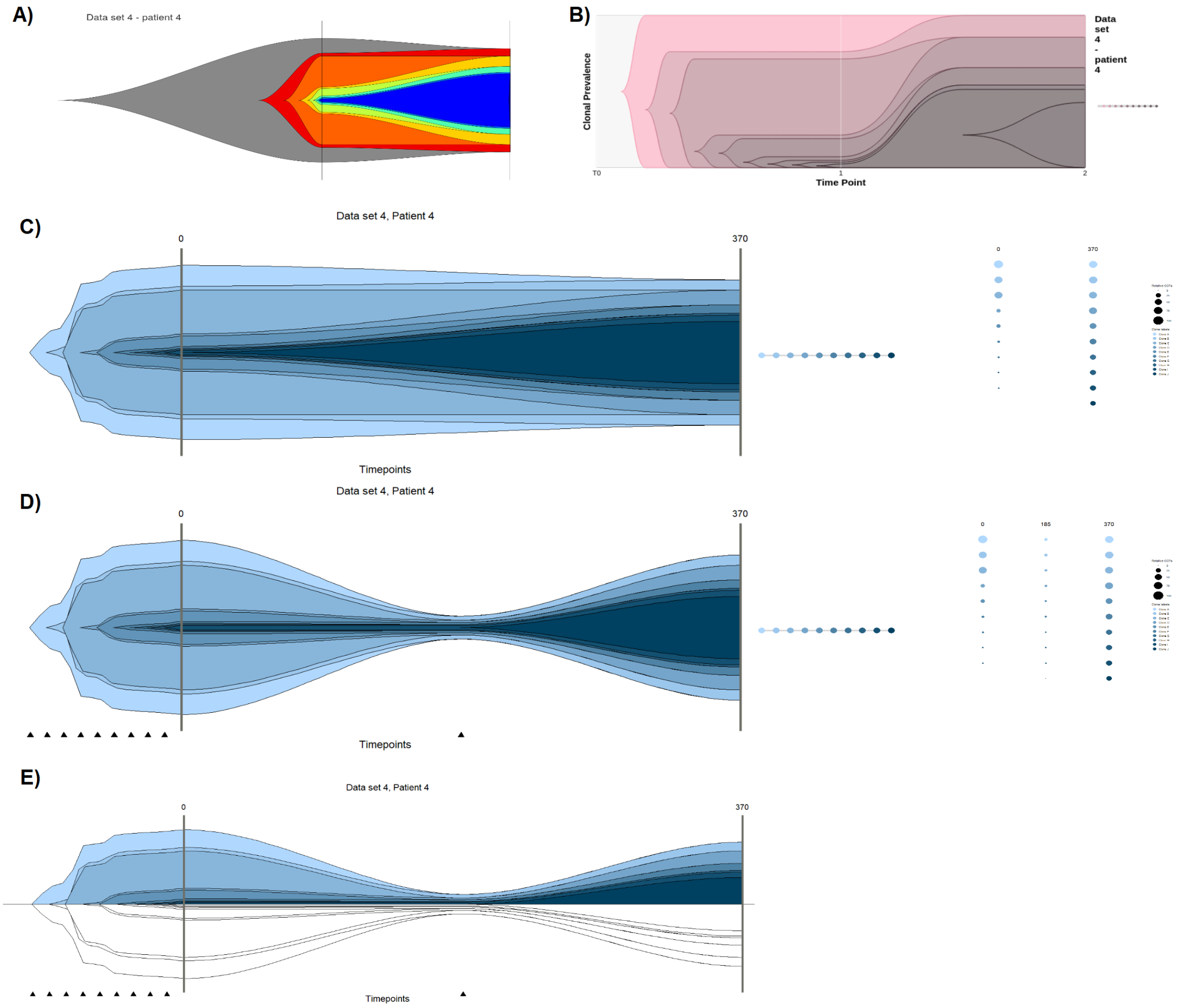

Figure S38: Visualization of clonal evolution of patient 4 in data set 4. A) Fishplot. B) Timescape. C) clevRvis using dolphin plots and extended shark plots, enabling time point interpolation for improved visualization of newly developing clones. D) clevRvis using dolphin plots and extended shark plots, enabling time point interpolation and therapy effect estimation. E) clevRvis using plaice plots (only focusing on *TP53*), enabling time point interpolation and therapy effect estimation. A point mutation affects *TP53*. Additionally, a CNV below detection threshold is assumed to be present. However, as it is unclear whether it is a deletion or duplication, no clone is marked.

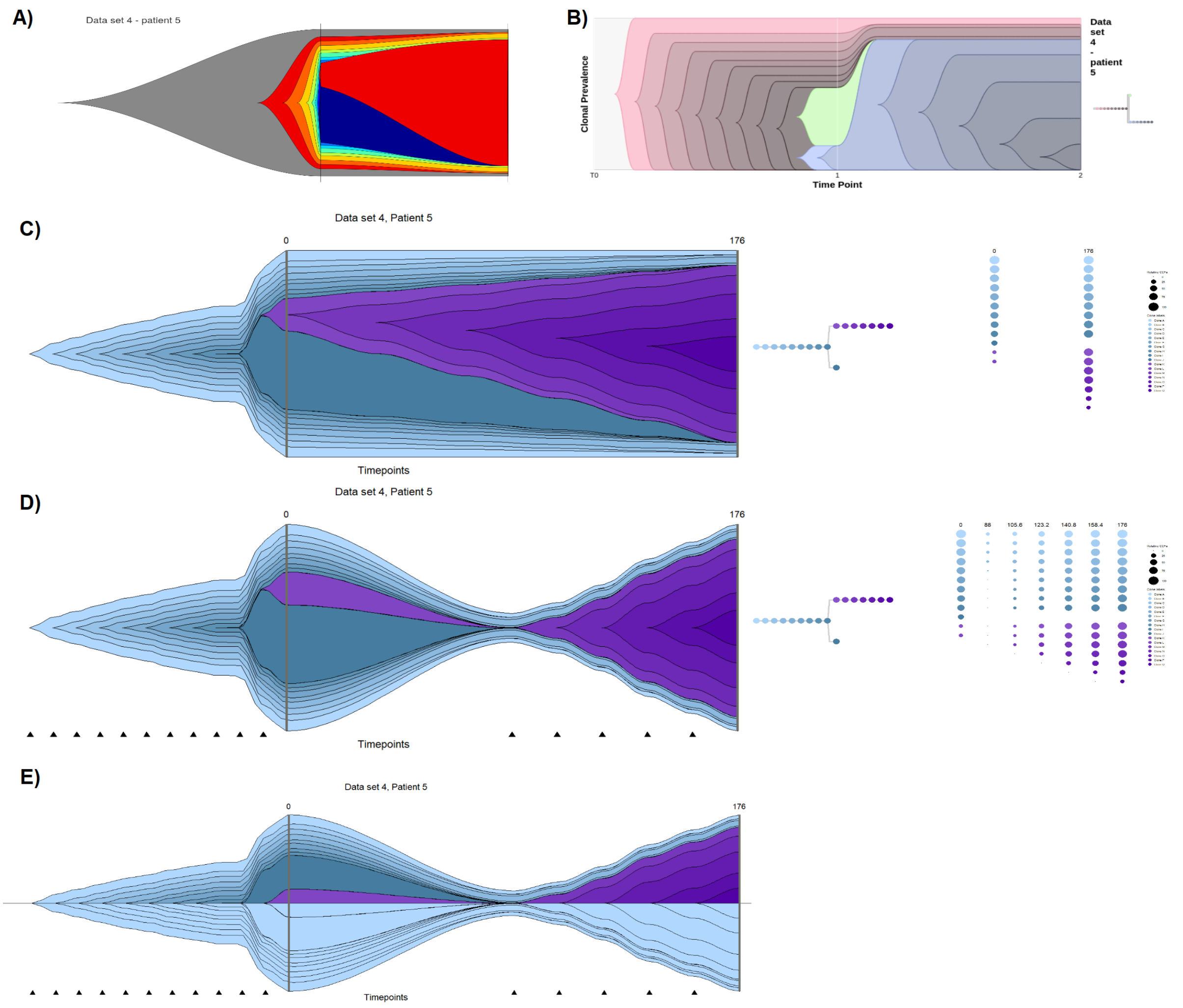

Figure S39: Visualization of clonal evolution of patient 5 in data set 4. A) Fishplot. B) Timescape. C) clevRvis using dolphin plots and extended shark plots, enabling time point interpolation for improved visualization of newly developing clones. D) clevRvis using dolphin plots and extended shark plots, enabling time point interpolation and therapy effect estimation. E) clevRvis using plaice plots (only focusing on *TP53*), enabling time point interpolation and therapy effect estimation. A bi-allelic event affecting *TP53* (loss of heterozygosity + point mutation in *TP53*) is marked.

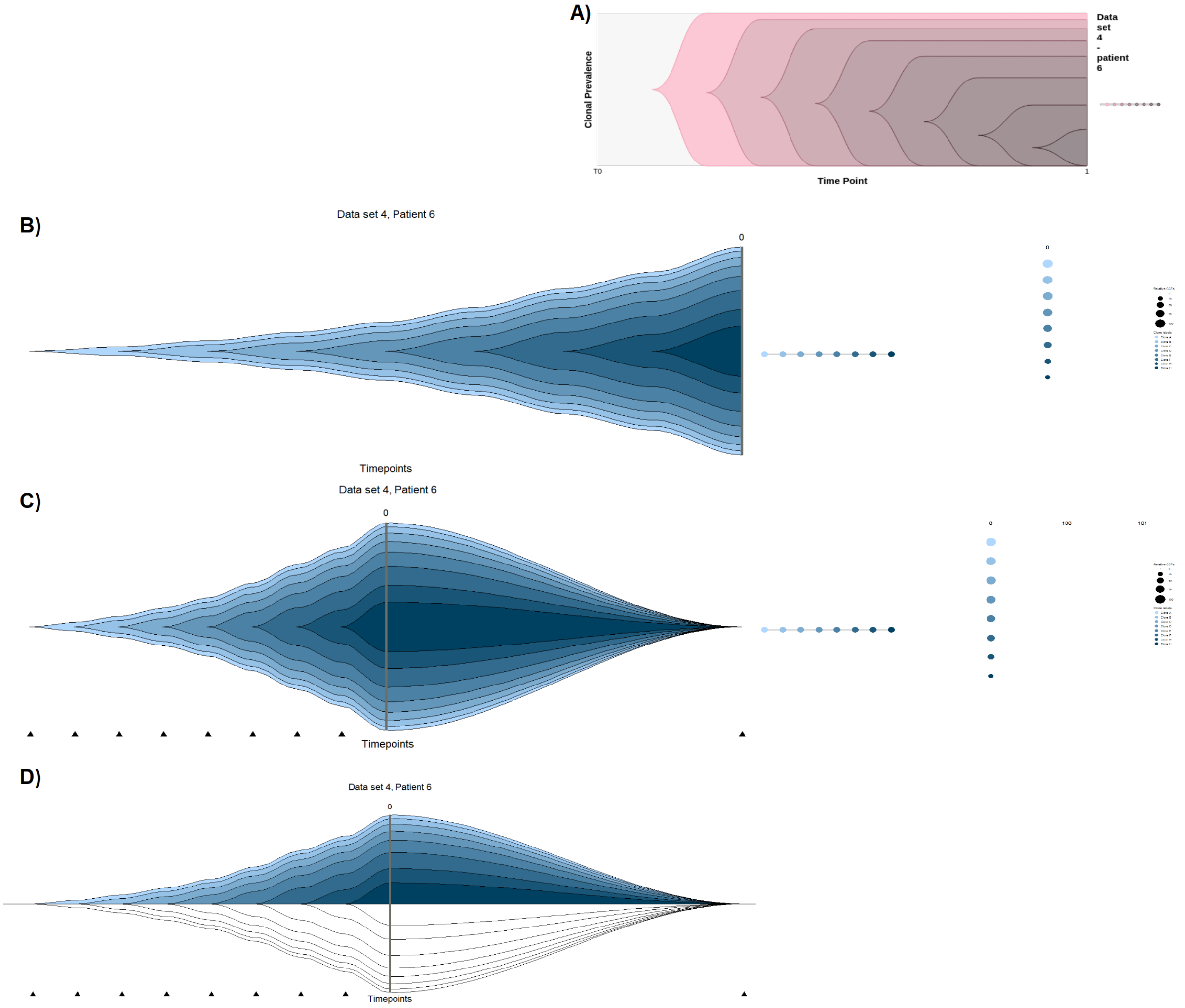

Figure S40: Visualization of clonal evolution of patient 6 in data set 4. A) Timescape. B) clevRvis using dolphin plots and extended shark plots, enabling time point interpolation for improved visualization of newly developing clones. C) clevRvis using dolphin plots and extended shark plots, enabling time point interpolation and therapy effect estimation. D) clevRvis using plaice plots (only focusing on *TP53*), enabling time point interpolation and therapy effect estimation. As all clones feature at least one healthy allele of *TP53*, no clone is marked. Note: A single time point cannot be visualized using fishplot.

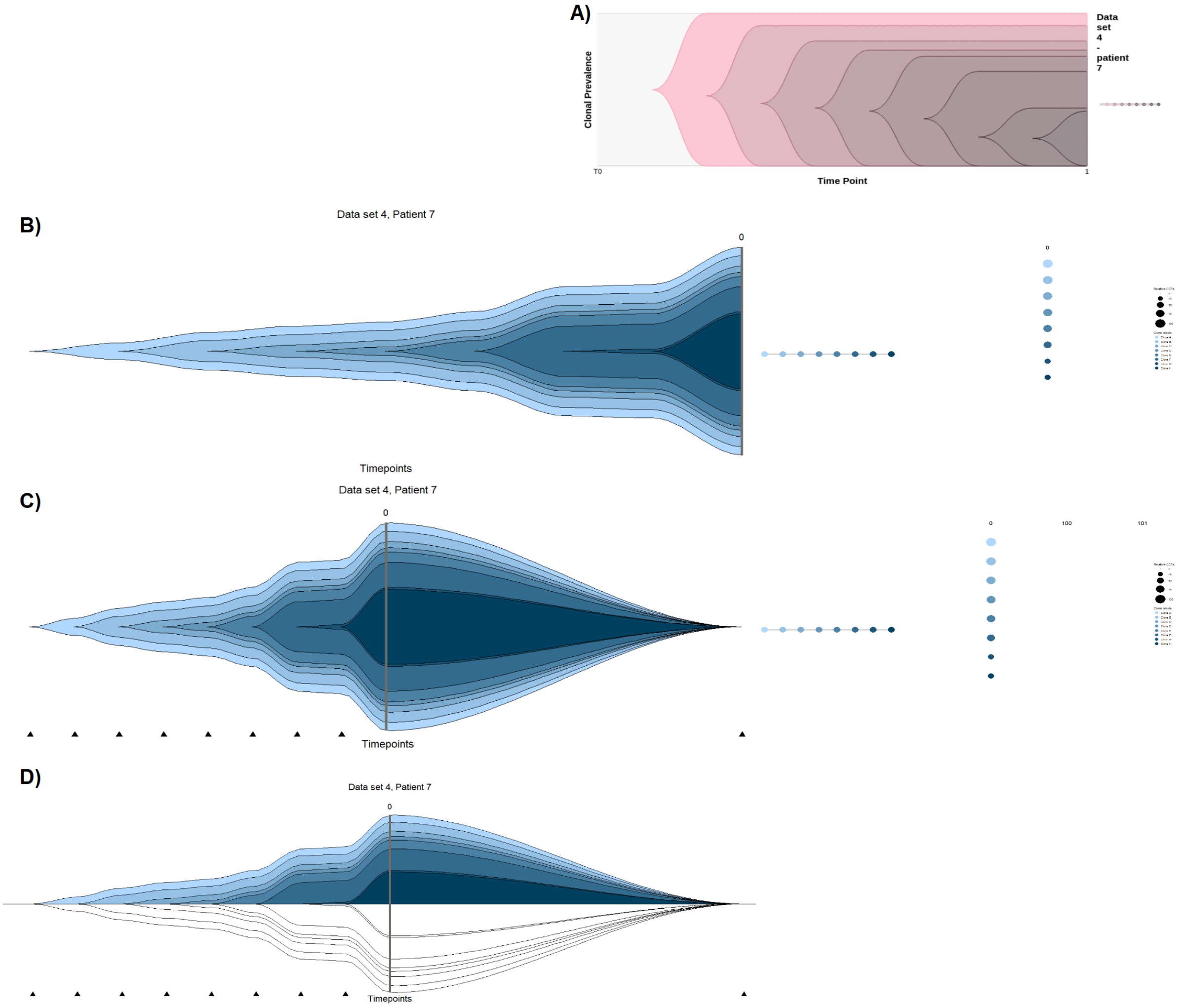

Figure S41: Visualization of clonal evolution of patient 7 in data set 4. A) Timescape. B) clevRvis using dolphin plots and extended shark plots, enabling time point interpolation for improved visualization of newly developing clones. C) clevRvis using dolphin plots and extended shark plots, enabling time point interpolation and therapy effect estimation. D) clevRvis using plaice plots (only focusing on *TP53*), enabling time point interpolation and therapy effect estimation. As all clones feature at least one healthy allele of *TP53*, no clone is marked. Note: A single time point cannot be visualized using fishplot.

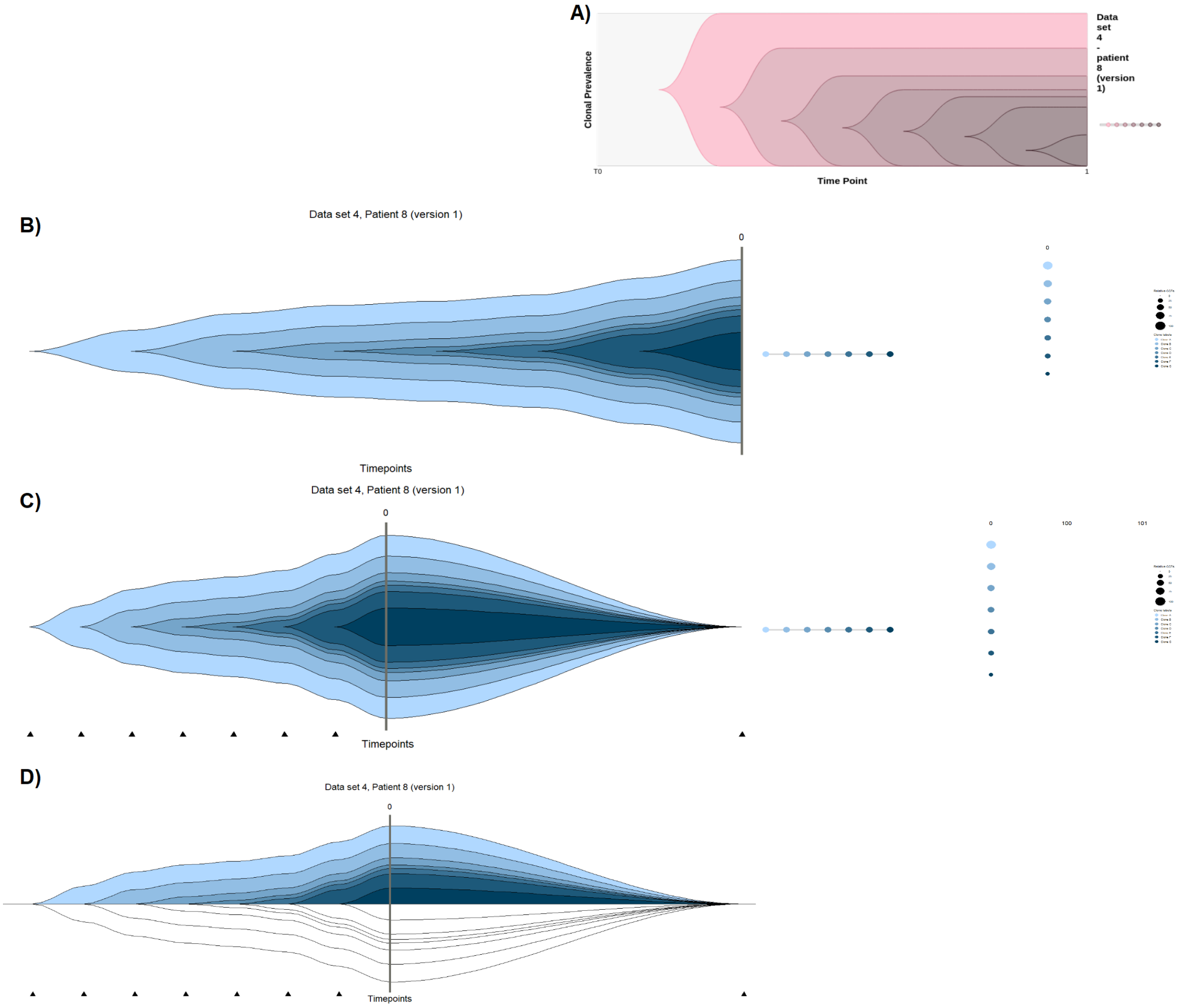

Figure S42: Visualization of clonal evolution of patient 8 (version 1) in data set 4. A) Timescape. B) clevRvis using dolphin plots and extended shark plots, enabling time point interpolation for improved visualization of newly developing clones. C) clevRvis using dolphin plots and extended shark plots, enabling time point interpolation and therapy effect estimation. D) clevRvis using plaice plots (only focusing on *TP53*), enabling time point interpolation and therapy effect estimation. As all clones feature at least one healthy allele of *TP53*, no clone is marked. Note: A single time point cannot be visualized using fishplot.

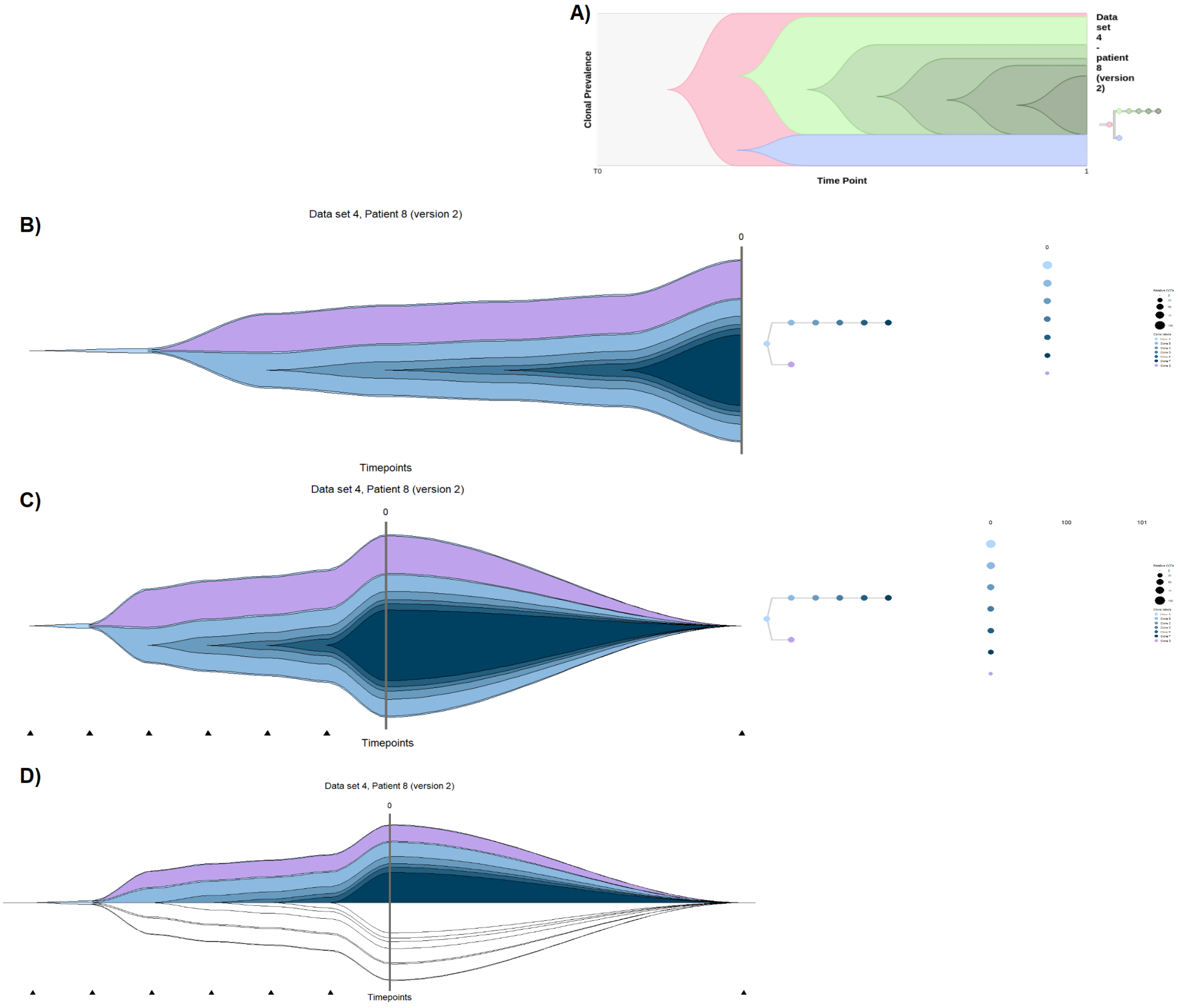

Figure S43: Visualization of clonal evolution of patient 8 (version 2) in data set 4. A) Timescape. B) clevRvis using dolphin plots and extended shark plots, enabling time point interpolation for improved visualization of newly developing clones. C) clevRvis using dolphin plots and extended shark plots, enabling time point interpolation and therapy effect estimation. D) clevRvis using plaice plots (only focusing on *TP53*), enabling time point interpolation and therapy effect estimation. As all clones feature at least one healthy allele of *TP53*, no clone is marked. Note: A single time point cannot be visualized using fishplot.

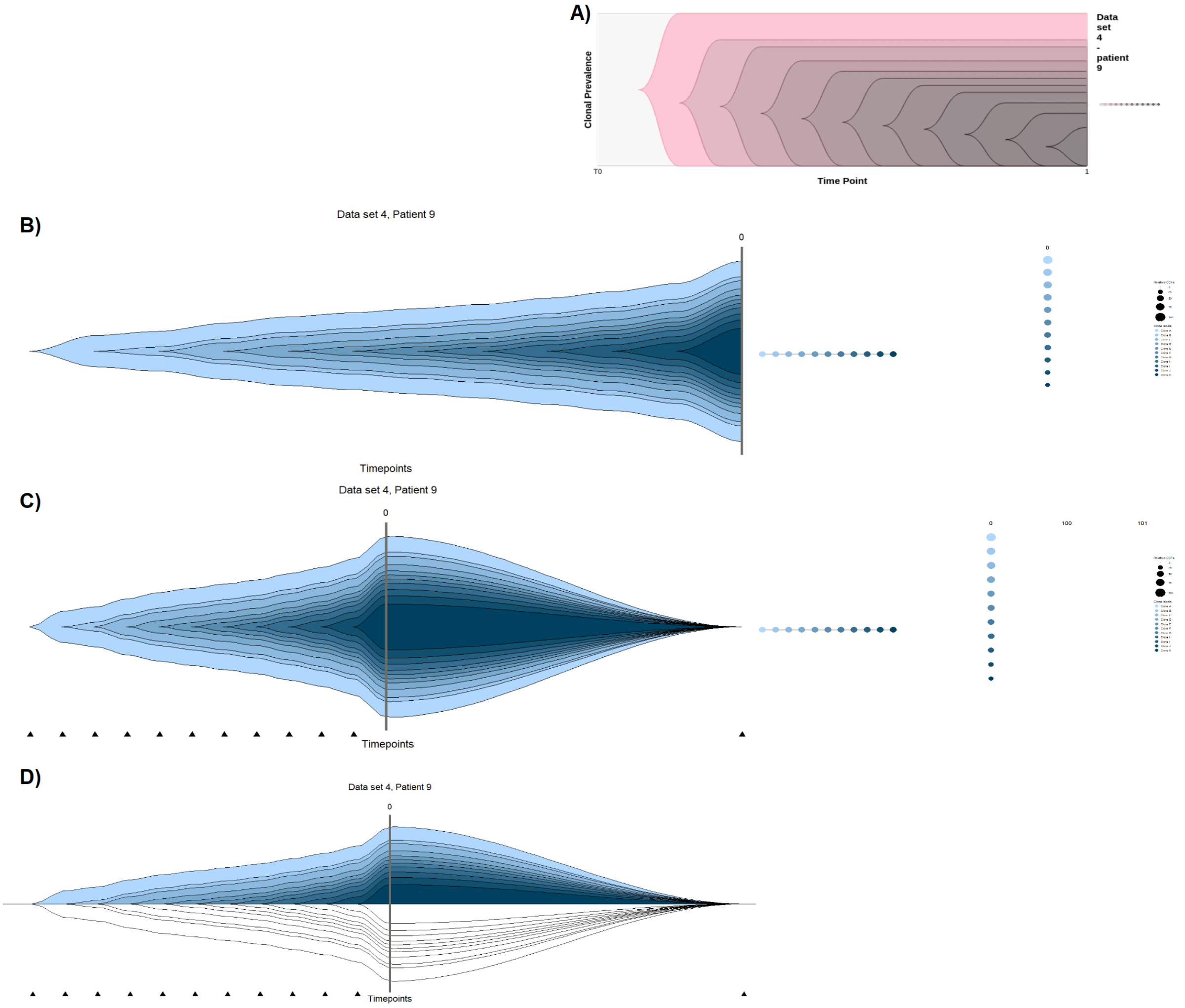

Figure S44: Visualization of clonal evolution of patient 9 in data set 4. A) Timescape. B) clevRvis using dolphin plots and extended shark plots, enabling time point interpolation for improved visualization of newly developing clones. C) clevRvis using dolphin plots and extended shark plots, enabling time point interpolation and therapy effect estimation. D) clevRvis using plaice plots (only focusing on *TP53*), enabling time point interpolation and therapy effect estimation. As all clones feature at least one healthy allele of *TP53*, no clone is marked. Note: A single time point cannot be visualized using fishplot.

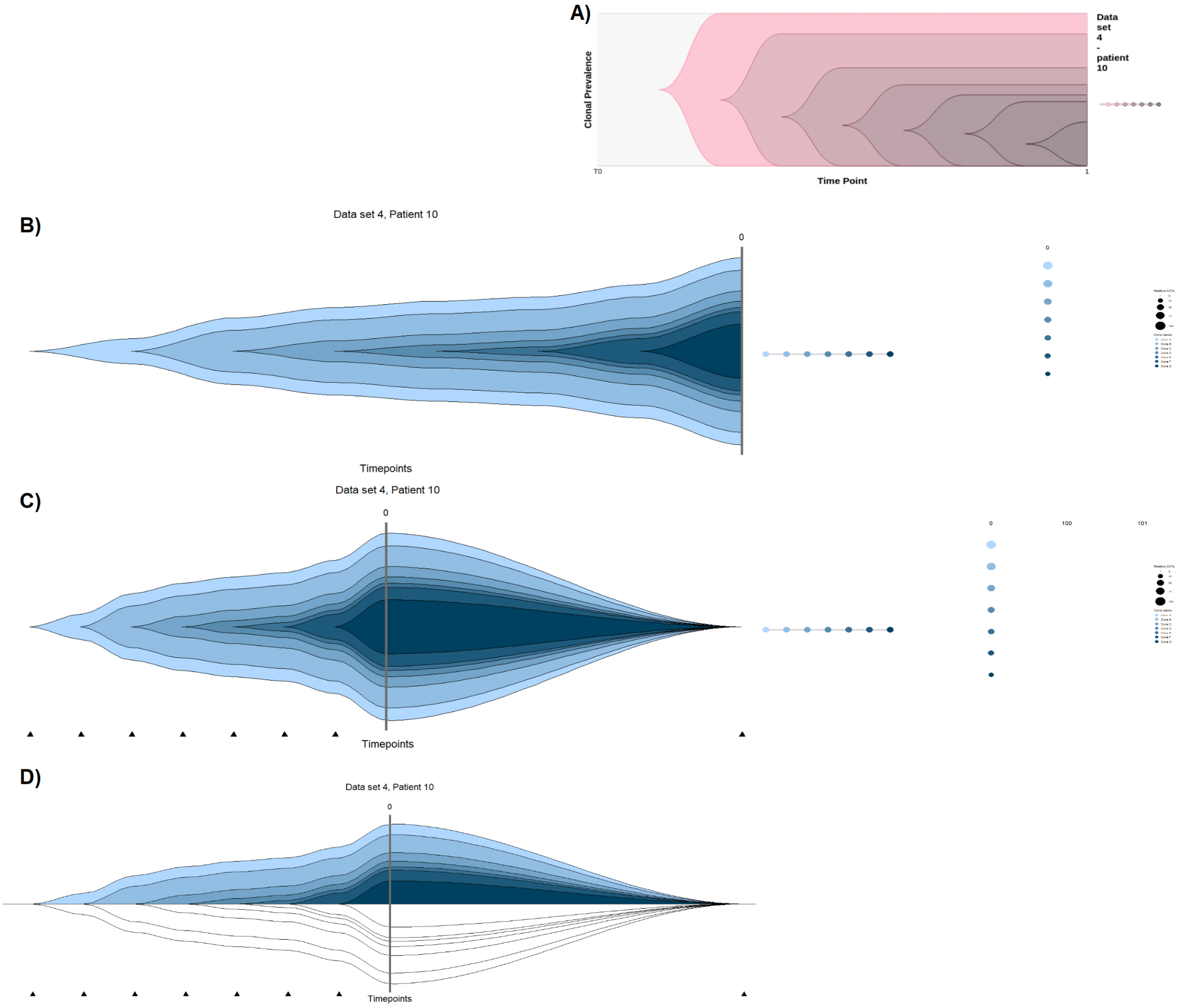

Figure S45: Visualization of clonal evolution of patient 10 in data set 4. A) Timescape. B) clevRvis using dolphin plots and extended shark plots, enabling time point interpolation for improved visualization of newly developing clones. C) clevRvis using dolphin plots and extended shark plots, enabling time point interpolation and therapy effect estimation. D) clevRvis using plaice plots (only focusing on *TP53*), enabling time point interpolation and therapy effect estimation. As all clones feature at least one healthy allele of *TP53*, no clone is marked. Note: A single time point cannot be visualized using fishplot.

2.5 Visualizing complex cases of clonal evolution with clevRvis

In clevRvis, we implemented an approach for automatic phylogeny-aware color coding. A maximum of 25 independent clones, developing from normal cells, and an unlimited number of related clones is supported. To evaluate clevRvis' ability to visualize highly complex cases of clonal evolution, we consider two simulated patients.

Simulated patient 1 is characterized by 100 clones. The clones are organized in 6 independent and 5 dependent branches. Within each branch, additional linear clonal evolution can be observed. Exemplary visualization with clevRvis is provided in Figure S46.

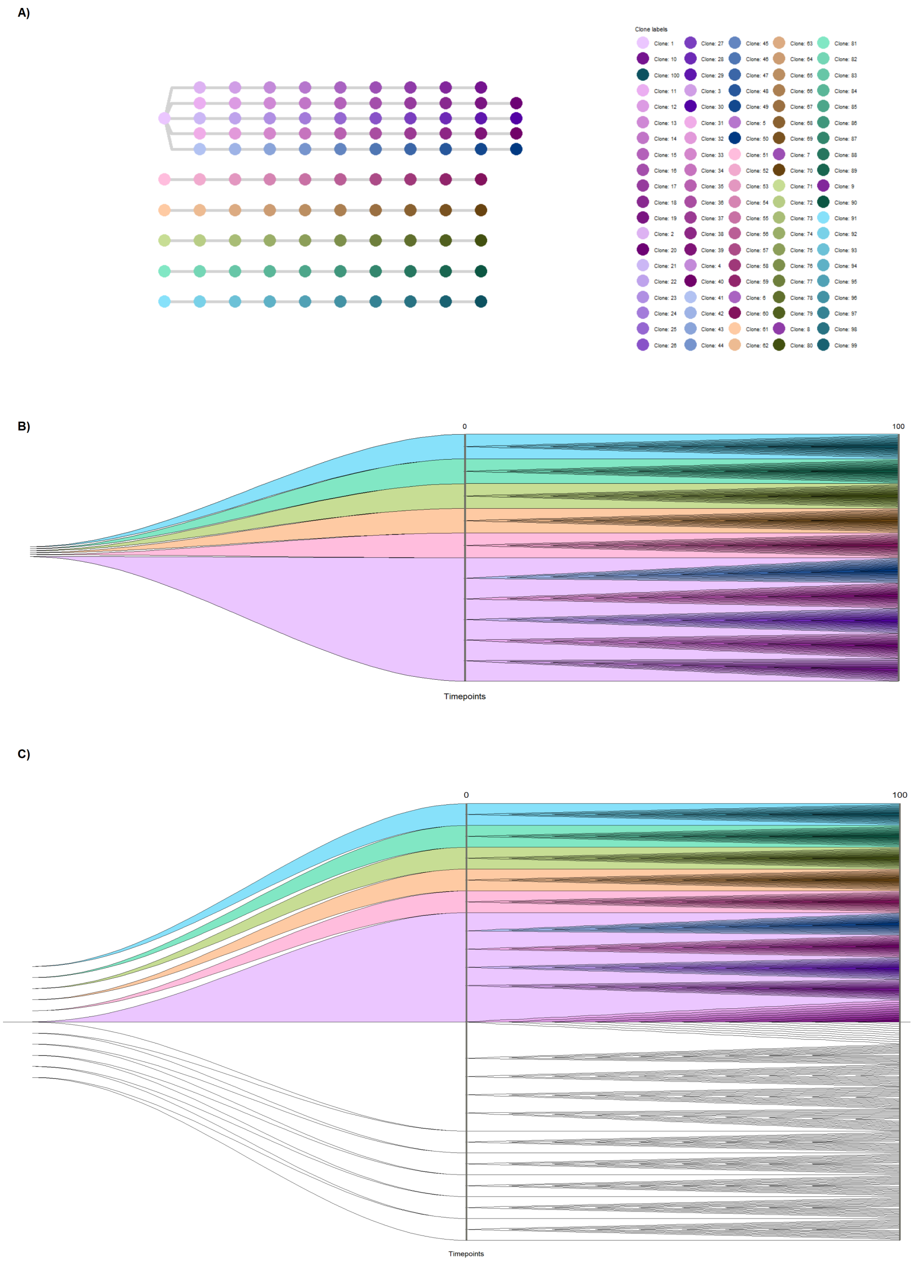

Figure S46: Visualization of clonal evolution of an exemplary case with 100 clones. A) clevRvis using shark plots. B) clevRvis using dolphin plots, enabling time point interpolation for improved visualization of newly developing clones. C) clevRvis using plaice plots, enabling time point interpolation.

It can be observed that clevRvis, including automatic phylogeny-aware color coding, supports visualization of data characterized by a high number of clones. All main plot types – shark, dolphin and plaice – can be used. Of note, differentiating between different clones according to their color is partly difficult. This is especially true for branched dependent evolution. However, defining 100 different colors is a challenge by itself, which inevitably leads to similar colors.

Simulated patient 2 is characterized by 100 time points. Linear clonal evolution of 5 clones can be observed. Exemplary visualization with clevRvis is provided in Figure S47.

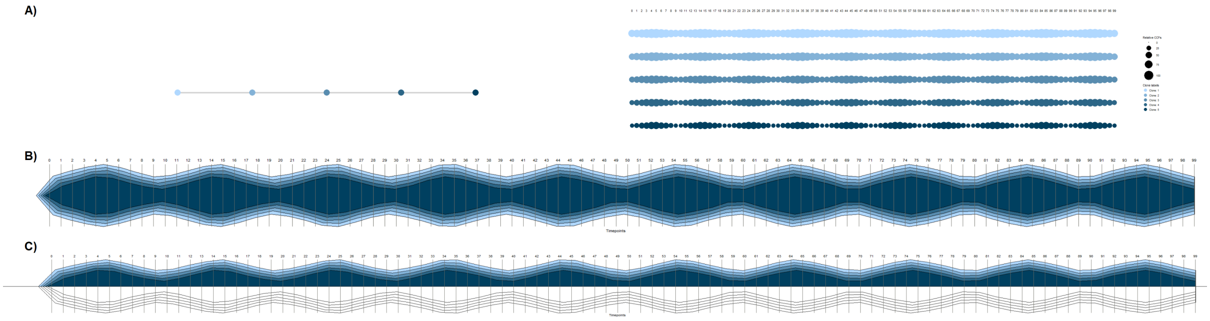

Figure S47: Visualization of clonal evolution of an exemplary case with 100 time points. A) clevRvis using extended shark plots. B) clevRvis using dolphin plots, enabling time point interpolation for improved visualization of newly developing clones. C) clevRvis using plaice plots, enabling time point interpolation.

It can be observed that clevRvis also supports visualization of data characterized by a high number of time points. All main plot types – shark, dolphin and plaice – can be used.

For both cases, high number of clones and high number of time points, we recommend exporting the plots with high resolution to optimize visualization.

References

[1] da Silva-Coelho P, Kroeze LI, Yoshida K *et al.* Clonal evolution in myelodysplastic syndromes. *Nat Commun.* 2017;**8**:15099.

[2] González-Rincón J, Gómez S, Martinez N *et al.* Clonal dynamics monitoring during clinical evolution in chronic lymphocytic leukaemia. *Sci Rep.* 2019;**9**:975.

[3] Sandmann S, Behrens YL, Davenport C *et al.* Clonal Evolution at First Sight: A Combined Visualization of Diverse Diagnostic Methods Improves Understanding of Leukemic Progression. *Front Oncol.* 2022;**12**:888114.

[4] Reutter K, Sandmann S, Rohde J *et al.* Reconstructing clonal evolution in relapsed and non-relapsed Burkitt lymphomaa. *Leukemia.* 2021;**35**:639-643.
