# Supplementary material for: clevRvis: visualization techniques for clonal evolution
Source: Gigascience. 2023 Apr 11;12:giad020. doi: 10.1093/gigascience/giad020 (PMC10087014; doi:10.1093/gigascience/giad020)
Supplement: giad020_Response_to_Reviewer_Comments_Original_Submission [file giad020_response_to_reviewer_comments_original_submission.pdf]

Author's Response To Reviewer Comments

Close

Editor:

1) Please include a link to an RCRAN/bioconductor version of the tool in the revised paper.

Changes: clevRvis was successfully submitted to Bioconductor and accepted as a package (https://bioconductor.org/packages/clevRvis). The clevRvis-package is currently available at Bioconductor's development-branch and is expected to be included in the Bioconductor 3.17 Release in April/May 2023. The link was included in the manuscript.

2) Reviewer #1 also has found some issues with the code which will need to be looked into carefully.

Changes: We carefully revised our code and examples. We added additional exemplary code. Furthermore, we extended our documentation on how to execute the examples, differentiating between examples for the “function-version” of clevRvis and the “shiny-version”.

3) In addition, please register any new software application in the bio.tools and SciCrunch.org databases to receive RRID (Research Resource Identification Initiative ID) and biotoolsID identifiers, and include these in your manuscript. Computational workflows should be registered in workflowhub.eu and the DOIs cited in the relevant places in the manuscript. These will facilitate tracking, reproducibility and re-use of your tool.

Changes: We registered clevRvis at bio.tools and SciCrunch.org, and added information on the biotools ID and the SciCrunch RRID to our manuscript.

We had a detailed look at workflowhub.eu and currently available workflows. To the best of our knowledge, only comprehensive workflows – partly using one or more R packages – are currently available at this website. R packages that stand alone, like clevRvis, we could not identify. Therefore, we opted against uploading clevRvis to workflowhub.eu for the moment.

Reviewer #1:

1) I do wonder if replacing "timepoint estimation" with "timepoint interpolation" might make things a little more clear. This tool isn't somehow gaining insight into non-represented intermediate timepoints, it's just evenly spacing events/changes throughout the missing time. That's certainly a fine (and very useful) approach for visualization purposes, but I initially assumed it was doing something more complicated! To be clear, I am not arguing that this has to be changed in the manuscript, but it's something to consider.

Changes: We changed “time point estimation” to “time point interpolation” – in the manuscript, the documentation of clevRvis and the functions.

2) I also think that at some future point (also not necessary for this manuscript), it might be nice to include options to the timepoint "estimation" that would allow events to be skewed left or right, if the user has belief that mutations were acquired early or late in the period between timepoints.

Changes: We added a detailed description and exemplary code to the supplement, describing how clevRvis'

time point interpolation can be used to visualize skewed events (Additional File 1, section 1.6 “Using time point interpolation to implement skewed events”).

3) It took a while to wrap my head around the coloring of the plaice plots (e.g. Figure 3b). The match in color between the TP53 deletion (in clone 2) and the TP53 mutation (in clone 1) is a bit confusing at first. (why is clone 2's shape colored with clone 1's color?) While I understand now, and don't have a specific suggestion for how to improve it, it's something to ponder.

Changes: We added the section “Recommended color coding for plaice plots” to the supplement (Additional File 1, section 1.7), suggesting rules for coloring the “lower plaice” and providing a visual example with additional explanation.

4) With the version that I pulled (commit 83508c3 from Sep 14), man pages seemed to be missing, such that typing `?sharkPlot` or `?createSeaObject` didn't display the expected help text. That'd be a useful addition.

Changes: clevRvis is now available as a Bioconductor package (https://bioconductor.org/packages/clevRvis). Manual pages for all functions should now be accessible, typing e.g. `?sharkPlot` or `?createSeaObject'.

5) I tried to convert the example frac.table and parents info from github into an example csv and load it into the shiny app, but was unable to get it to run.

Changes: We carefully revised our code and examples. We added additional exemplary code. Furthermore, we extended our documentation on how to execute the examples, differentiating between examples for the “function-version” of clevRvis and the “shiny-version”. Additionally, we added an error message if a user provides non-numeric time points as input.

We are very sorry for the problems and the confusion that arose from the exemplary data we provided. The exemplary code available in the vignette (parents, fracTable etc.) was intended exclusively for use of the “function-version” of clevRvis. Of course, the fracTable we provide may be converted to e.g. a CSV file and taken as input for clevRvis. However, the reason why the error message “NAs are not allowed in subscripted assignments" appeared is as follows: by default, clevRvis assums that the first row contains information on the time points (if this option is de-selected, the first row is assumed to contain CCFs of a clone). However, these time points are required to be numeric so that the subsequent algorithms for time point interpolation and therapy effect estimation do work. By changing the CSV file to

```
"","1","2","3"
"1",20,40,50
"2",10,20,25
"3",0,15,15
"4",0,0,10
"5",0,30,40
"6",0,10,20
"7",0,0,15
```

or – alternatively – leaving out the first line and selecting “Use default time points? (1,2…)”, everything should work as expected.
We added a code example to our vignette (section clevRvisShiny() > Examples > Input – without parental relations and Input – with parental relations) on how to generate CSV files for use in clevRvisShiny() based on a fracTable matrix, a timepoints vector and optionally a parents vector.
We completely agree with the reviewer that this could have caused several support tickets in the future. Therefore, we added an automatic check to our “shiny-version” on whether the first row contains numeric values and – if this is not the case – a meaningful error message appears.

6) Outside of the GUI, when I ran `dolphinPlot(seaObject)` with the example data from github, I got output, but also a warning. These appear non-fatal, but it would be nice to suppress them or generate a more informative error if is something is actually wrong here.

Changes: The warnings are non-fatal. We had a look at all warnings reported by clevRvis and removed the reasons – wherever it was possible – or included suppressWarnings() otherwise.

Reviewer #2:

1) I am wondering why authors did not format the tool as an R package, while a list of R script? For unexperienced users, it might be difficult to directly use the R scripts. I saw the documentation of functions is already in vignettes/clevR-vis.html, so with the package "devtools", I think it should be very easy to turn it into an R package.

Changes: clevRvis was successfully submitted to Bioconductor and accepted as a package (https://bioconductor.org/packages/clevRvis). The clevRvis-package is currently available at Bioconductor's development-branch and is expected to be included in the Bioconductor 3.17 Release in April/May 2023.

2) The shiny app looks great. I think it can also be integrated in the package. I tried to use the shiny app with the two example files provided (example1.xls and example2.xls). I can see the files are imported, but no plot is shown. I guess it is because the example files are too simple. I would suggest authors to provide a complete example file, also in the shiny app, to add a new button "load example" in the "Inputs" tab, so that users can know the format of the input file and fast try the shiny app.

Changes: We carefully revised our code and examples. Especially, we extended our documentation on how to execute the examples using the "shiny-version" of clevRvis. Furthermore, messages were added to the "seaObject table"-tab [Please upload a Cancer Cell Fractions table first ("Inputs" → "Submit inputs"). Then, configure the seaObject ("seaObject options" → "Submit") to visualize the seaObject Cancer Cell Fractions table.] and the "Plots"-tab [Please configure the seaObject first ("seaObject options" → "Submit"). Then, select the plots to visualize ("Plots" → switches for Shark, Dolphin and Plaice Plots)] if prerequisites for generating plots are not yet given.

We are very sorry for the inconveniences the reviewer experienced when testing clevRvis. Both files Example1.xlsx and Example2.xlsx should contain fully functional examples for the analysis with clevRvis. From what the reviewer describes, it could be that the exemplary files were successfully imported, but no seaObject was generated afterwards. When uploading an input file, it is automatically displayed as a datatable in the input tab on the right. Subsequently, a user has to define the parental relations (information available as a table at the end of the vignette; update now: parental relations can also be uploaded directly, using one of the new, exemplary files Example1_withParents.xlsx and Example2_withParents. xlsx that are provided along with the package). As soon as information on parental relations has been provided, a user has to click "Submit inputs" to execute the validity check. If a success message is displayed, one may move on to the "seaObject options". Even if "Extra time point interpolation" and "Therapy effect estimation" are not enabled, a user has to click "Submit" to generate a seaObject table (shown in the tab "seaObject table" on the right). Then, finally, plots can be generated and inspected in the "Plots"-tab.

3) In the shiny app, I saw the relations of parent-child clones should be manually selected. Maybe authors can allow to upload the relations as a second table file?

Changes: The upload options have been extended. Optionally, a user may now upload a CCF table (clones x time points) additionally containing a "parents" column with information on the parental relations.

Information is automatically imported. Still, a CCF table not containing any parent information can be uploaded and the relations defined manually.

4) If authors plan to submit the R package to CRAN or Bioconductor, "clevR-vis" might need to be changed to e.g. "clevRvis" or maybe "clever"? Hyphen is not allowed in the package name on these two repositories.

Changes: We changed the name of our algorithm and the R package to clevRvis.

5) Last, in Figure 2 and 3, the legends are missing. Maybe add a global legend for all panels?

Changes: We added global legends to Figures 2 and 3 (now: Figures 3 and 4).

Reviewer #3:

1) Comparison against timescape and fishplot: Differences between clevR-vis and the two previous methods (timescape and fishplot) should be illustrated using real data. Also, the differences between a fishplot and dolphin plot are not clear to me. As far as I can tell, the visualization is identical, barring small diferences in how clones are ordered and the use of colormaps. clevR-vis does include interpolation, which might lead to smoother fishplots. I encourage the authors to better illustrate the differences.

Changes: We revised our manuscript and added more information on the comparison of clevRvis vs fishplot and timescape, especially focusing on additional features only provided by clevRvis. We added Figure 2 to the Results section, highlighting differences in the fish plots generated by the three approaches, considering 3 exemplary patients (real data). Evaluation of real data from all 31 patients is available in Additional File 1 (Figures S11-S45).

For the visualization of clonal evolution, we consider fish plots being the most established solution, as they unite information on phylogeny, CCFs and time course in a single elegant plot. By our approach, clevRvis, we aimed at providing an extensive set of visualization techniques for clonal evolution. Therefore, clevRvis also contains an implementation of fish plots. We decided to call them "dolphin plots" as we liked to stick to the "sea"-naming and – in comparison to the rather raw graph (=shark) plots – they appeared to us a more "friendly" visualization, which lead us to the name "dolphin plots".
The reviewer is thoroughly right in noticing that fish plots, as implemented in the fishplot or timescape package, and dolphin plots contain the same basis. However, we aimed at optimizing the visualization (e.g. by automatic phylogeny-aware color coding, showing the correct starting point for all clones, single time point and single clone visualization being supported, providing an option to switch between centered and bottom layout, providing interactive plots and interactive annotation). Furthermore, clevRvis contains several unique features, e.g. shark plots and extended shark plots, plaice plots, algorithms for time point interpolation and therapy effect estimation, a graphical user interface and – now added – an option to explore alternative trees.
We hope to show that clevRvis offers an optimized version of the established visualization of clonal evolution, as well as several novel visualization techniques and features for advanced analysis of clonal evolution.

2) Better description of methods: There are three steps that need to be described more carefully in the Methods part of the paper: (i) estimation of initial CCFs, (ii) estimation of additional timepoints and CCF interpolation, (iii) estimation of tumor load. For step (i) no description in Methods is given. It is also not clear how many additional timepoints are added. For (iii), it is not clear how tumor load is calculated for previous and subsequent timepoints prior and after treatment. Pseudocode is not an alternative to a precise textual description.

Changes: We revised the description of our Algorithm 3 "time point interpolation", adding further details.

Especially, information was added on how the number of interpolated time points is defined. Furthermore, we added a detailed description of Algorithm 4 “therapy effect estimation”. Of note, as the functionality of clevRvis was extended, Algorithm 2 “exploring alternative trees” was added to the Methods section (pseudocode + detailed description).

3) Usefulness of plaice plots: The paper motivates the use of plaice plots to visualize bi-allelic events. An example of such an event is two hits of TP53, where one allelic copy is deleted and the other copy undergoes a truncating mutation. I am not convinced that such events warrant a new type of visualization. A disadvantage of plaice plots is that one loses half the visualization real estate due to mirroring along the x-axis. The more general feature seems to be highlighting/emphasizing clones that contain a pre-specified set of mutations. This can be accomplished in the original fishplot using for instance edge color.

Changes: We carefully revised our manuscript and added a discussion on the potential difficulty to see complex clonal evolution patterns in plaice plots vs dolphin plots. Furthermore, we outline the added value of plaice plots compared to marking clones affected by bi-allelic events in common fish plots. Plaice plots provide the unique additional option to link bi-allelic events to characteristic clones by suitable color-coding. We added the section “Recommended color coding for plaice plots” to the supplement (Additional File 1, section 1.7), suggesting rules for coloring the “lower plaice” and providing a visual example with additional explanation.

4) Lack of interactivity: A missed opportunity is that the visualizations are mostly static. Interactivity would greatly improve the tool. For instance, filtering based on mutations, listing clone proportions (ccf(parent) - sum ccfs of children), exploring alternative tree solutions.

Changes: Interactivity of clevRvis was extended: For the input, now, a user has the option to explore alternative tree solutions (available in the shiny-app + as individual new function exploreTrees()). For the seaObject, now, a user may easily switch between visualization of “CCF_clone” vs “CCF_clone – sum(CCF_children)”. For the plots, clevRvis still offers the option of interactive plots (available in the shiny-app + in the individual plot functions).

We would like to thank the reviewer for his very valuable ideas of increasing clevRvis' level of interactivity! For now, we opted against including an option to filter based on mutations. clevRvis does – in its current version – solely work on information characterizing clones. We assume that a user initially performs clustering of mutations to determine clones and their CCFs using other established approaches. However, we hope to extend our “clevR” software suite in near future, developing e.g. “clevRclust” for this analysis step. For the commonly subsequent analysis step – reconstructing clonal evolution trees, which also includes exploring alternative trees – we extended our clevRvis package. Taking information on the available time points and CCFs of each clone at each time point as input, our novel exploreTrees()-function investigates possible, alternative parental relations and reports all valid solutions, passing clevRvis' validity check. Results are available in a drop-down menu (shiny-version) or as a list (function-version). Subsequently, a user may select any of these parental relations for visualization with clevRvis.

5) Examples in R package, bioconductor/cran: To facilitate reproduction and also ease of use, I encourage the authors to save the seaObjects corresponding to the real data figures as .Rdata files. Moreover, it might be good to include one or two examples in the library. Finally, I recommend publishing this on bioconductor/cran.

Changes: seaObjects for all real data figures are now available as file “data.Rdata” at Giga DB. clevRvis was successfully submitted to Bioconductor and accepted as a package (https://bioconductor.org/packages/clevRvis). The clevRvis-package is currently available at Bioconductor's development-branch and is expected to be included in the Bioconductor 3.17 Release in April/May 2023. Examples are included in the functions' manual pages as well as in the vignette. For the ”shiny-version” of clevRvis, we provide 4 exemplary xlsx-files (2 exemplary patients, each with and without information on parental relations included in the file). Furthermore, the vignette contains exemplary code to generate a

CSV file for the analysis with clevRvisShiny(), based the fracTable matrix, a time points vector and optionally a parents vector.

6) Another quality of life improvement is that the tree itself should be taken as input. Setting edges in the UI is cumbersome.

Changes: The upload options have been extended. Optionally, a user may now upload a CCF table (clones x time points) additionally containing a “parents” column with information on the parental relations. Information is automatically imported. Still, a CCF table not containing any parent information can be uploaded and the relations defined manually.

7) List limitations: Finally, I'd like to see a discussion of limitations. For instance, how many clones do you support? How many timepoints? How realistic is the timepoint estimation? What about multiple spatial locations per timepoint?

Changes: We added section 2.5 “Visualizing complex cases of clonal evolution” to Additional file 1, considering visualization of a simulated data set with 100 clones, and another set with 100 time points using clevRvis. The results are considered in our Discussion. Additionally, we revised our Discussion adding the limitation that, due to limited data being available, we could not check validity of our algorithms interpolating time points and estimating therapy effect for all patients. However, analysis of the two real data sets 1 and 2 showed that our algorithms generate realistic results. Considering multiple spatial locations per time point was added as future work to our Conclusions.

Close
