# Supplementary material for: clevRvis: visualization techniques for clonal evolution
Source: Gigascience. 2023 Apr 11;12:giad020. doi: 10.1093/gigascience/giad020 (PMC10087014; doi:10.1093/gigascience/giad020)
Supplement: giad020_Response_to_Reviewer_Comments_Revision_1 [file giad020_response_to_reviewer_comments_revision_1.pdf]

Author's Response To Reviewer Comments

Close

We added our ORCID iDs to the title page. The data availability section was modified as suggested, now citing our archived code and data available at GigaDB.

As pdf building from our tex-file went wrong in the EM, we uploaded the manuscript as pdf and added all files (incl figures, bibliography and style files) to re-build our manuscript as "supplemental material".

Close
