# Supplementary material for: clevRvis: visualization techniques for clonal evolution
Source: Gigascience. 2023 Apr 11;12:giad020. doi: 10.1093/gigascience/giad020 (PMC10087014; doi:10.1093/gigascience/giad020)
Supplement: giad020_Reviewer_1_Report_Original_Submission — Christopher Miller -- 10/25/2022 Reviewed [file giad020_reviewer_1_report_original_submission.pdf]

Reviewer Report

Title: clevRvis: Visualization Techniques for Clonal Evolution

Version: Original Submission **Date:** 10/25/2022

Reviewer name: Christopher Miller

Reviewer Comments to Author:

Overall, this is a nicely put-together R package that improves upon previous visualization tools for clonal evolution. It's novelty comes specifically from offering a graphical user interface, many improved options for plotting and annotation, sensible and informative default color schemes, and additional plot types that add insight. It undoubtedly moves the field forward and will find many eager users among folks who are studying clonal evolution.The manuscript is well written and I have only a few comments:- I agree with the arguments made about the timepoint and therapy estimation being valid (and very useful!) approaches to visualization. That said, I do wonder if replacing "timepoint estimation" with "timepoint interpolation" might make things a little more clear. This tool isn't somehow gaining insight into non-represented intermediate timepoints, it's just evenly spacing events/changes throughout the missing time. That's certainly a fine (and very useful) approach for visualization purposes, but I initially assumed it was doing something more complicated! To be clear, I am not arguing that this has to be changed in the manuscript, but it's something to consider.- I also think that at some future point (also not necessary for this manuscript), it might be nice to include options to the timepoint "estimation" that would allow events to be skewed left or right, if the user has belief that mutations were acquired early or late in the period between timepoints.- It took a while to wrap my head around the coloring of the plaice plots (e.g. Figure 3b). The match in color between the TP53 deletion (in clone 2) and the TP53 mutation (in clone 1) is a bit confusing at first. (why is clone 2's shape colored with clone 1's color?) While I understand now, and don't have a specific suggestion for how to improve it, it's something to ponder.After running the code, I have several additional comments:- With the version that I pulled (commit 83508c3 from Sep 14), man pages seemed to be missing, such that typing `?sharkPlot` or `?createSeaObject` didn't display the expected help text. That'd be a useful addition. In many cases, the code appears to have the required docs in the code:#' Create a seaObject#'#' @param fracTable A numeric matrix containing tumor fraction estimates for all clones at all timepoints#' @param parents An integer vector specifying parental relationships between clones. . .It's then relatively straightforward to use the the `devtools` package to convert these comments to the necessary man pages - (see "How do I Document My Functions?" here: https://tinyheero.github.io/jekyll/update/2015/07/26/making-your-first-R-package.html)- I tried to convert the example frac.table and parents info from github into an example csv and load it into the shiny app, but was unable to get it to run. Specifically this data:parents <- c(0,1,1,3,0,5,6)fracTable <- matrix(c(20,10,0,0,0,0,0,40,20,15,0,30,10,0,50,25,15,10,40,20,15),ncol = length(timepoints))I then converted it to a CSV that looks like:"","V1","V2","V3""1",20,40,50"2",10,20,25"3",0,15,15"4",0,0,10"5",0,30,40"6",0,10,20"7",0,0,15I then uploaded it with the same parental relationships as above. When doing so, I got a success message, and the table appeared to be sane. When I submitted from the "seaObject options" panel, I get a second

"Success" message. Then, when I then attempt to click on "seaObject Table", I get an error: "NAs are not allowed in subscripted assignments" and could not create any plots. It is possible that I did something wrong, but it also may be worth investigating. If a GUI is being offered to non-savvy users, more informative errors and data checking will probably save you a lot of support tickets!- I was able to successfully load the provided example xlsx files from github and produce plots that way, showing that the software does work in that case.- Outside of the GUI, when I ran `dolphinPlot(seaObject)` with the example data from github, I got output, but also a warning:Warning: stack imbalance in 'NextMethod', 92 then 93Warning messages:1: In regularize.values(x, y, ties, missing(ties)) :collapsing to unique 'x' values. . .These appear non-fatal, but it would be nice to suppress them or generate a more informative error if is something is actually wrong here.These are generally minor points, though, and as a whole, I think this is an excellent paper, a useful tool, and both should be well-received by the cancer genomics community

Level of Interest

Please indicate how interesting you found the manuscript: Choose an item.

Quality of Written English

Please indicate the quality of language in the manuscript: Choose an item.

Declaration of Competing Interests

Please complete a declaration of competing interests, considering the following questions:

- Have you in the past five years received reimbursements, fees, funding, or salary from an organisation that may in any way gain or lose financially from the publication of this manuscript, either now or in the future?
- Do you hold any stocks or shares in an organisation that may in any way gain or lose financially from the publication of this manuscript, either now or in the future?
- Do you hold or are you currently applying for any patents relating to the content of the manuscript?
- Have you received reimbursements, fees, funding, or salary from an organization that holds or has applied for patents relating to the content of the manuscript?
- Do you have any other financial competing interests?
- Do you have any non-financial competing interests in relation to this paper?

If you can answer no to all of the above, write 'I declare that I have no competing interests' below. If your reply is yes to any, please give details below.

I declare that I have no competing interests

I agree to the open peer review policy of the journal. I understand that my name will be included on my report to the authors and, if the manuscript is accepted for publication, my named report including any attachments I upload will be posted on the website along with the authors' responses. I agree for my report to be made available under an Open Access Creative Commons CC-BY license (http://creativecommons.org/licenses/by/4.0/). I understand that any comments which I do not wish to be included in my named report can be included as confidential comments to the editors, which will not be published.

Choose an item.

To further support our reviewers, we have joined with Publons, where you can gain additional credit to further highlight your hard work (see: https://publons.com/journal/530/gigascience). On publication of this paper, your review will be automatically added to Publons, you can then choose whether or not to claim your Publons credit. I understand this statement.

Yes Choose an item.
