# Supplementary material for: clevRvis: visualization techniques for clonal evolution
Source: Gigascience. 2023 Apr 11;12:giad020. doi: 10.1093/gigascience/giad020 (PMC10087014; doi:10.1093/gigascience/giad020)
Supplement: giad020_Reviewer_2_Report_Original_Submission — Zuguang Gu -- 11/3/2022 Reviewed [file giad020_reviewer_2_report_original_submission.pdf]

Reviewer Report

Title: clevRvis: Visualization Techniques for Clonal Evolution

Version: Original Submission **Date:** 11/3/2022

Reviewer name: Zuguang Gu

Reviewer Comments to Author:

In the manuscript entitled "clevR-vis: Visualization Techniques for Clonal Evolution", authors described a tool which provides nice visualization on tumor evolution. Authors performed comparisons to current tools with public datasets. Also, the supplementary file contains very comprehensive information. In general, the manuscript is well written. I am not an expert in the field of tumor evolution, so I only give comments on the implementation of the tool.I am wondering why authors did not format the tool as an R package, while a list of R script? For unexperienced users, it might be difficult to directly use the R scripts. I saw the documentation of functions is already in vignettes/clevR-vis.html, so with the package "devtools", I think it should be very easy to turn it into an R package.The shiny app looks great. I think it can also be integrated in the package. I tried to use the shiny app with the two example files provided (example1.xls and example2.xls). I can see the files are imported, but no plot is shown. I guess it is because the example files are too simple. I would suggest authors to provide a complete example file, also in the shiny app, to add a new button "load example" in the "Inputs" tab, so that users can know the format of the input file and fast try the shiny app.In the shiny app, I saw the relations of parent-child clones should be manually selected. Maybe authors can allow to upload the relations as a second table file?If authors plan to submit the R package to CRAN or Bioconductor, "clevR-vis" might need to be changed to e.g. "clevRvis" or maybe "clever"? Hyphen is not allowed in the package name on these two repositories.Last, in Figure 2 and 3, the legends are missing. Maybe add a global legend for all panels?

Level of Interest

Please indicate how interesting you found the manuscript: Choose an item.

Quality of Written English

Please indicate the quality of language in the manuscript: Choose an item.

Declaration of Competing Interests

Please complete a declaration of competing interests, considering the following questions:

- Have you in the past five years received reimbursements, fees, funding, or salary from an organisation that may in any way gain or lose financially from the publication of this manuscript, either now or in the future?

- Do you hold any stocks or shares in an organisation that may in any way gain or lose financially from the publication of this manuscript, either now or in the future?
- Do you hold or are you currently applying for any patents relating to the content of the manuscript?
- Have you received reimbursements, fees, funding, or salary from an organization that holds or has applied for patents relating to the content of the manuscript?
- Do you have any other financial competing interests?
- Do you have any non-financial competing interests in relation to this paper?

If you can answer no to all of the above, write 'I declare that I have no competing interests' below. If your reply is yes to any, please give details below.

I declare that I have no competing interests.

I agree to the open peer review policy of the journal. I understand that my name will be included on my report to the authors and, if the manuscript is accepted for publication, my named report including any attachments I upload will be posted on the website along with the authors' responses. I agree for my report to be made available under an Open Access Creative Commons CC-BY license (http://creativecommons.org/licenses/by/4.0/). I understand that any comments which I do not wish to be included in my named report can be included as confidential comments to the editors, which will not be published.

Choose an item.

To further support our reviewers, we have joined with Publons, where you can gain additional credit to further highlight your hard work (see: https://publons.com/journal/530/gigascience). On publication of this paper, your review will be automatically added to Publons, you can then choose whether or not to claim your Publons credit. I understand this statement.

Yes Choose an item.
