# Supplementary material for: clevRvis: visualization techniques for clonal evolution
Source: Gigascience. 2023 Apr 11;12:giad020. doi: 10.1093/gigascience/giad020 (PMC10087014; doi:10.1093/gigascience/giad020)
Supplement: giad020_Reviewer_3_Report_Original_Submission — Mohammed El-Kebir -- 11/8/2022 Reviewed [file giad020_reviewer_3_report_original_submission.pdf]

Reviewer Report

Title: clevRvis: Visualization Techniques for Clonal Evolution

Version: Original Submission **Date:** 11/8/2022

Reviewer name: Mohammed El-Kebir

Reviewer Comments to Author:

This paper introduces clevR-vis, an R package to visualize cancer phylogenies. This tool is implemented in R, and includes an implementation as well as extension of the previous fishplot. Briefly, the authors introduce (i) a shark plot (which is a simple tree along with a clone x timepoint matrix of CCFs), (ii) a dolphin plot, which as far as I can tell is a fishplot, and (iii) a plaice plot, which is a fishplot that is mirrored along the x-axis, showing "bi-allelic events". These are static visualizations. Moreover, the paper introduces several heuristics for CCF interpolation as well as estimation of tumor load. There is an R shiny user interface. I installed the tool -- the documentation is clear but the tool and paper can be improved. My comments are below.1. Comparison against timescape and fishplotDifferences between clevR-vis and the two previous methods (timescape and fishplot) should be illustrated using real data. Also, the differences between a fishplot and dolphin plot are not clear to me. As far as I can tell, the visualization is identical, barring small diferences in how clones are ordered and the use of colormaps. clevR-vis does include interpolation, which might lead to smoother fishplots. I encourage the authors to better illustrate the differences.2. Better description of methodsThere are three steps that need to be described more carefully in the Methods part of the paper: (i) estimation of initial CCFs, (ii) estimation of additional timepoints and CCF interpolation, (iii) estimation of tumor load. For step (i) no description in Methods is given. It is also not clear how many additional timepoints are added. For (iii), it is not clear how tumor load is calculated for previous and subsequent timepoints prior and after treatment. Pseudocode is not an alternative to a precise textual description.3. Usefulness of plaice plotsThe paper motivates the use of plaice plots to visualize bi-allelic events. An example of such an event is two hits of TP53, where one allelic copy is deleted and the other copy undergoes a truncating mutation. I am not convinced that such events warrant a new type of visualization. A disadvantage of plaice plots is that one loses half the visualization real estate due to mirroring along the x-axis. The more general feature seems to be highlighting/emphasizing clones that contain a pre-specified set of mutations. This can be accomplished in the original fishplot using for instance edge color.4. Lack of interactivityA missed opportunity is that the visualizations are mostly static. Interactivity would greatly improve the tool. For instance, filtering based on mutations, listing clone proportions (ccf(parent) - sum ccfs of children), exploring alternative tree solutions.5. Examples in R package, bioconductor/cranTo facilitate reproduction and also ease of use, I encourage the authors to save the seaObjects corresponding to the real data figures as .Rdata files. Moreover, it might be good to include one or two examples in the library. Finally, I recommend publishing this on bioconductor/cran.Another quality of life improvement is that the tree itself should be taken as input. Setting edges in the UI is cumbersome.6. List limitationsFinally, I'd like to see a discussion of limitations. For instance, how many clones do you

support? How many timepoints? How realistic is the timepoint estimation? What about multiple spatial locations per timepoint?

Level of Interest

Please indicate how interesting you found the manuscript: Choose an item.

Quality of Written English

Please indicate the quality of language in the manuscript: Choose an item.

Declaration of Competing Interests

Please complete a declaration of competing interests, considering the following questions:

- Have you in the past five years received reimbursements, fees, funding, or salary from an organisation that may in any way gain or lose financially from the publication of this manuscript, either now or in the future?
- Do you hold any stocks or shares in an organisation that may in any way gain or lose financially from the publication of this manuscript, either now or in the future?
- Do you hold or are you currently applying for any patents relating to the content of the manuscript?
- Have you received reimbursements, fees, funding, or salary from an organization that holds or has applied for patents relating to the content of the manuscript?
- Do you have any other financial competing interests?
- Do you have any non-financial competing interests in relation to this paper?

If you can answer no to all of the above, write 'I declare that I have no competing interests' below. If your reply is yes to any, please give details below.

None

I agree to the open peer review policy of the journal. I understand that my name will be included on my report to the authors and, if the manuscript is accepted for publication, my named report including any attachments I upload will be posted on the website along with the authors' responses. I agree for my report to be made available under an Open Access Creative Commons CC-BY license (http://creativecommons.org/licenses/by/4.0/). I understand that any comments which I do not wish to be included in my named report can be included as confidential comments to the editors, which will not be published.

Choose an item.

To further support our reviewers, we have joined with Publons, where you can gain additional credit to further highlight your hard work (see: https://publons.com/journal/530/gigascience). On publication of this paper, your review will be automatically added to Publons, you can then choose whether or not to claim your Publons credit. I understand this statement.

Yes Choose an item.
